# Models for the No-Observed-Effect Concentration (NOEC) and Maximal Half-Effective Concentration (EC50)

**DOI:** 10.3390/toxics12060425

**Published:** 2024-06-12

**Authors:** Nadia Iovine, Alla P. Toropova, Andrey A. Toropov, Alessandra Roncaglioni, Emilio Benfenati

**Affiliations:** Istituto di Ricerche Farmacologiche Mario Negri IRCCS, Via Mario Negri 2, 20156 Milan, Italy; nadia.iovine@marionegri.it (N.I.); alla.toropova@marionegri.it (A.P.T.); andrey.toropov@marionegri.it (A.A.T.); alessandra.roncaglioni@marionegri.it (A.R.)

**Keywords:** harlequin fly, swollen duckweed, QSAR, Monte Carlo method, index of ideality of correlation, correlation intensity index

## Abstract

Typical in silico models for ecotoxicology focus on a few endpoints, but there is a need to increase the diversity of these models. This study proposes models using the NOEC for the harlequin fly (*Chironomus riparius*) and EC50 for swollen duckweed (*Lemna gibba*) for the first time. The data were derived from the EFSA OpenFoodTox database. The models were based on the correlation weights of molecular features used to calculate the 2D descriptor in CORAL software. The Monte Carlo method was used to calculate the correlation weights of the algorithms. The determination coefficients of the best models for the external validation set were 0.74 (NOAEC) and 0.85 (EC50).

## 1. Introduction

The aquatic ecosystem has an important role for humans, animals, plants, and aquatic organisms. Water pollution resulting from humans as consumers and other anthropogenic activities such as agriculture and industry have negative impacts on aquatic organisms. Hazard and risk assessments are needed to investigate the adverse effects of chemicals on water life such as aquatic plants, fish, and algae [1,2,3].

Considering the amount of chemicals released into the environment, fast, accurate, and cost-effective assessments are required. Several organizations and regulations such as the United States Environmental Protection Agency (US EPA), REACH regulation (Registration, Evaluation, Authorization and Restriction of Chemicals) in Europe, the European Center for Validation of Alternative Methods (ECVAM) of the European Union, and the European Union Commission Scientific Committee on Toxicity, Ecotoxicity, and Environment (CSTEE) suggest and encourage an in silico approach for risk assessment [1]. Among the in silico approaches, the quantitative structure–activity relationship (QSAR) is a rapid and low-cost method to create models to test a large number of chemicals, reducing the number of time-consuming in vivo tests [4].

There are several assays to investigate aquatic ecotoxicity, and some are validated and standardized [5,6,7,8,9,10]. Swollen duckweed (*Lemna gibba*) has many attributes that make it useful for ecosystem health assessment [11,12,13]. The OECD Test Guidelines (TG) 221 are designed to assess the toxicity of substances to freshwater aquatic plants of the genus *Lemna* (duckweed). Plant cultures are exposed to different concentrations of the test substance for seven days to test the substance-related effects on vegetative growth. The effects are quantified by comparing growth in test solutions to the control and the concentration that causes x% inhibition of growth is determined (e.g., EC50).

The harlequin fly (*Chironomus riparius*) is used for ecotoxicological studies to assess acute and sub-lethal effects of chemicals in soil and water [14,15,16]. The OECD TG 218, 219, and 233 [5,6,8] are designed to assess the effects of prolonged exposure to chemicals on the lifecycle of the sediment-dwelling freshwater dipteran *Chironomus* sp. The instar chironomid larvae are placed in a sediment–water system together with the test substance. Chironomid emergence and development rates are measured. This test shows the concentration that caused x% reduction in emergence, larvae survival and growth (e.g., EC50), and/or the no-observed-effect concentration (NOEC). The OECD TG 233 [8], compared to the former, covers all the lifecycles of the first generation and the early part of the second generation, and effects are measured as the TG described previously.

The NOEC and EC50 are endpoints largely used in ecotoxicology to define the critical effects caused by long-term exposure (NOEC) [17] and acute exposure (EC50) [18] to substances for non-target organisms. In this paper, we collected NOEC and EC50 data from the EFSA OpenFoodTox database [19] and developed two QSAR models for the prediction of ecotoxicological endpoints: NOEC for the harlequin fly and EC50 for swollen duckweed. These models are based on the correlation weights of molecular features used to calculate the 2D descriptor in the CORAL software (http://www.insilico.eu/coral/ accessed on 11 June 2024). The Monte Carlo method has been used for many other models [20,21,22,23,24,25,26,27].

## 2. Materials and Methods

### 2.1. Data

Experimental NOEC for harlequin fly (*n* = 122) and EC50 swollen duckweed (*n* = 94) were collected from the EFSA OpenFoodTox database version 6 (https://zenodo.org/records/8120114, accessed on 16 April 2024). These values were converted into the logarithmic scale. The chemical substances with the experimental data for the two endpoints we studied are pesticides; for other families of substances, it is not common to perform this kind of experimental study. The datasets with the information on the compound structures and experimental values are reported in Table S1 (EC50 for swollen duckweed) and Table S2 (NOEC for harlequin fly). The folder “2D chemical structures”, available in Supplementary Materials, reports the 2D chemical structures of the compounds for both datasets.

### 2.2. Model

In this work, models of endpoints are regression relations of the following form [28,29]:

(1) $$Endpoint=C_{0}+C_{1}\times DCW\left(T;N\right)$$

The *DCW*(*T*, *N*) is the SMILES-based optimal descriptor, which is the sum of the so-called correlation weights. The numerical data on correlation weights were calculated with the Monte Carlo method. *T* and *N* are parameters of the Monte Carlo optimization procedure. *T* is the threshold to define active molecular features extracted from a simplified molecular input-line entry system (SMILES). *T* = 5 means that all molecular features present in the training set at least five times are active; otherwise, they are inactive. *N* is the number of epochs (iterations) of the Monte Carlo procedure. Starting from the same dataset, the substances were assigned to active training, passive training, calibration, or validation subsets. This splitting was repeated three times; thus, we developed three models for each endpoint.

### 2.3. Optimal SMILES-Based Descriptors

The descriptor used here was calculated as follows:

(2) $$DCW\left(T,N\right)=\sum CW(S_{k})+\sum CW({SS}_{k})$$

where *S_k_* is the SMILES atom, i.e., the undivided part of the SMILES. In other words, *S_k_* is a single symbol (‘C’, ‘O’, etc.) or a group of symbols that cannot be examined separately because they define the atom jointly (‘Cl’, ‘Br’, etc.). The *CW*(*x*) are the correlation weights of the corresponding molecular features.

### 2.4. Monte Carlo Optimization

The optimization of the correlation weights is based on the search for the maximum of the target function (*TF*) calculated as follows:

(3) $${TF}_{\;}=r_{AT}+r_{PT}-\left|r_{AT}-r_{PT}\right|\times 0.1+(IIC+CII)\times 0.3$$

where *IIC* is the index of the ideality of correlation. *CII* is the correlation intensity index. The application of *IIC* and *CII* improves the predictive potential of models (i.e., the statistical quality of the validation set) but to the detriment of values on the training set [30,31].

The use of *IIC* and *CII* is actually “statistical forcing”; the correlation weights are optimized to be more favorable for the calibration set than for the training sets. It is hoped (and often justified) that a favorable effect on the calibration set will be accompanied by a favorable effect on the external validation set. This way, the system prefers a general model rather than a model that is too close to the training set—in this last case, there is the risk of sticking to a model that is overfitting.

### 2.5. Validation of the Models

There are a number of conceptually different criteria for the predictive potential of QSAR models. Some of them are based on calculations with the training set only. Other criteria are calculated using an external validation set. In our case, the models were validated using the following criteria for the predictive potential:

- The determination coefficient (R^2^);
- The correlation ideality index (IIC) [30,31];
- The correlation intensity index (CII) [30,31];
- RMSE, which is the root mean squared error;
- MAE, which is the mean absolute error;
- F, which is the Fischer F ratio.

In this way, we calculated statistics suitable both for the internal and the eternal validation approaches.

## 3. Results

### 3.1. The New Models for the Environmental Protection

Our environment needs better protection. Unfortunately, this calls for a huge effort, to cover an incredibly large number of species. In silico models may be a valid help in this effort, but so far, most of the models relate to a few endpoints; typically, in silico models address fish, daphnia, and algae, partly because these are commonly requested by current regulation. Here, we introduce new models for other species, exploiting data from the OpenFoodTox database. In particular, we addressed the harlequin fly (*Chironomus riparius*) and swollen duckweed (*Lemna gibba*). Collections of experimental values from sound databases offer a powerful driver for the development of transparent and reproducible in silico models. Thus, this paper promotes this vision. Using the datasets from authoritative databases boosts the development of in silico models, and it is convenient to identify algorithms that can quickly process datasets and develop models. CORAL, the software applied in our study, is an example.

### 3.2. Models for NOEC for Harlequin Fly

Table 1 contains the main statistical characteristics of models for logNOEC for the harlequin fly. The statistical values are for each subset (active training, passive training, calibration, and validation), also considering the leave-one-out statistics, indicated as Q^2^. More details on the statistical parameters are given in Table S3 in Supplementary Materials. These models, for each of the three splits starting from the same overall dataset, are the following:

LogNOEC = −1.3840 (±0.1057) + 0.1873 (±0.0066) × DCW(5; 15)(4)

LogNOEC = −1.5626 (±0.0983) + 0.2218 (±0.0093) × DCW(5; 15)(5)

LogNOEC = −2.3440 (±0.0752) + 0.2848 (±0.0075) × DCW(5; 15)(6)

Table S4 in Supplementary Materials shows the experimental and calculated values and the applicability domain (AD) of logNOEC for the model obtained in the case of split 1.

### 3.3. Models for EC50 for Swollen Duckweed

Table 2 gives the statistical characteristics of models for logEC50. More details on the statistical parameters are given in Table S5 in Supplementary Materials. These models are the following, for the three splits:

LogEC50 = −1.1669 (±0.1576) + 0.1228 (±0.0100) × DCW(5; 15)(7)

LogEC50 = −1.8297 (±0.1470) + 0.4278 (±0.0189) × DCW(5; 15)(8)

LogEC50 = −1.2497 (±0.1670) + 0.1006 (±0.0079) × DCW(5; 15)(9)

Considering the results on the two endpoints, there is a similar situation. The results for the validation sets are better than those for the active and passive training sets. For instance, in the model for NOEC (Table 1), the *R*^2^ values for the validation sets are in the range of 0.72–0.75, while the *R*^2^ values for the training sets are in the range of 0.26–0.58. The situation is similar for the second endpoint, EC50 (Table 2). In this case, the *R*^2^ values for the training sets are in the range of 0.26–0.49, always lower than the *R*^2^ values of the validation sets, which are in the range of 0.77–0.85. This is due to the mechanism of the CORAL model which forces the optimization of the values of the calibration sets.

The situation is similar for *RMSE*. The ranges for training sets are 1.07–2.03 (NOEC) and 1.55–1.67 (EC50), while for calibration sets, the ranges are 0.449–0.968 (NOEC) and 0.726–0.631 (EC50). This is due to the influence of the factors *IIC* and *CII* on the Monte Carlo optimization method [20,27,30,31].

Table S6 in Supplementary Materials shows the experimental and calculated values and the applicability domain (AD) of EC50 for swollen duckweed for the model obtained in the case of split 1.

Compared with the common practice of splitting the set of substances into training and validation sets, our approach is more elaborate. We further split the set of substances to be used for the model development into three subsets, namely active training, passive training, and calibration, as described in the Materials and Methods section. In practice, the initial steps of model building, when active and passive training sets are used, are preliminary phases to identify the components of the models. The structure of the proposed model is decided in the calibration phase. Thus, the results on the calibration set are conceptually closer to those in the usual process where only one training set is used. For this reason, the values of the validation set should be compared only with the calibration set, which in fact are much more similar than those in the preliminary phases of the model using the active and passive training sets, which are related to a not fully developed model.

## 4. Discussion

Our models were developed using SMILES as the format to represent the chemical structures. This is one of the most commonly used formats for in silico models. There are several advantages associated with the use of this format. It is quite compact; for instance, the structure of 1-tert-butyl-3,5-dimethyl-2,4,6-trinitrobenzene requires 62 bytes when represented with the SMILES, 998 bytes using a connected table, and 2066 bytes using and MDL MOL file. This speeds up the calculation and makes the use of the developed models more convenient and faster. The SMILES format is somehow more readable, at least for expert users. The SMILES structures can be easily found on the internet, for instance, from PubChem (https://pubchem.ncbi.nlm.nih.gov/ accessed on 11 June 2024). There are disadvantages too. The SMILES format does not address the 3D structure, which is, however, not used in most in silico models for ecotoxicity. Indeed, 3D models require more complexity in handling the structure, which has to be optimized, and the benefits resulting from the improved description of the chemical structure are usually not appreciable due to the uncertainty of the experimental data. Furthermore, the SMILES format is not unique, even if we used the canonical SMILES. Different formats may be used, for instance, for the description of the nitro groups, and this has to be standardized before modeling.

The SMILES format can cover chirality. However, in our case, we did not use the chiral representation, since in our datasets, there was no information about the toxicity values specific to individual enantiomers. This is a common situation, and pure enantiomers are only used within specific sectors when the price of the product can afford the physical separation of less active enantiomers.

Beyond these general considerations regarding the use of SMILES for the representation of the substances, there is a particular advantage related to the use of the CORAL software. In CORAL, the SMILES is directly used by the software, without the need to calculate molecular descriptors starting from the 2D or 3D structure, which is typical in most in silico models. Indeed, the software processes directly the SMILES code through the identification of the sequence of characters. This represents an important simplification of the modeling scheme. This is convenient from the point of view of using and comparing models. The obtained results also allow us to conclude that the correlation ideality index can be a useful measure of the predictive potential of models.

**Figure 1:**
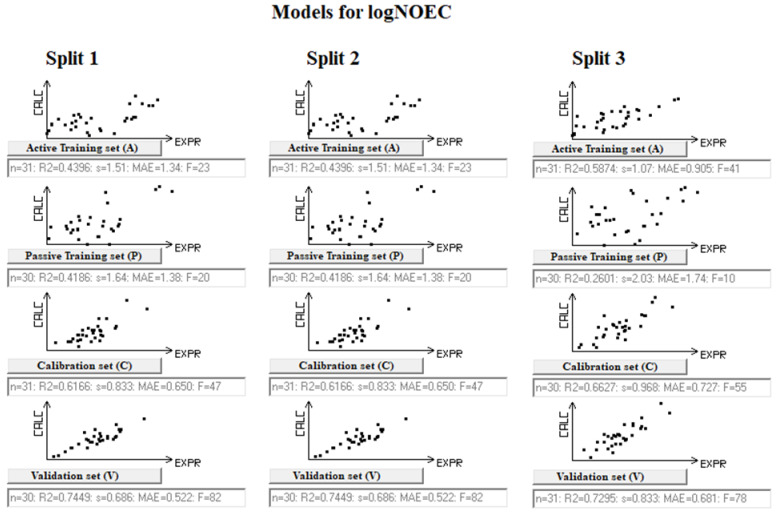
Splitting correlations for the active and passive training sets for logNOEC models for the harlequin fly. Black dots represent the substances in the subset.

**Figure 2:**
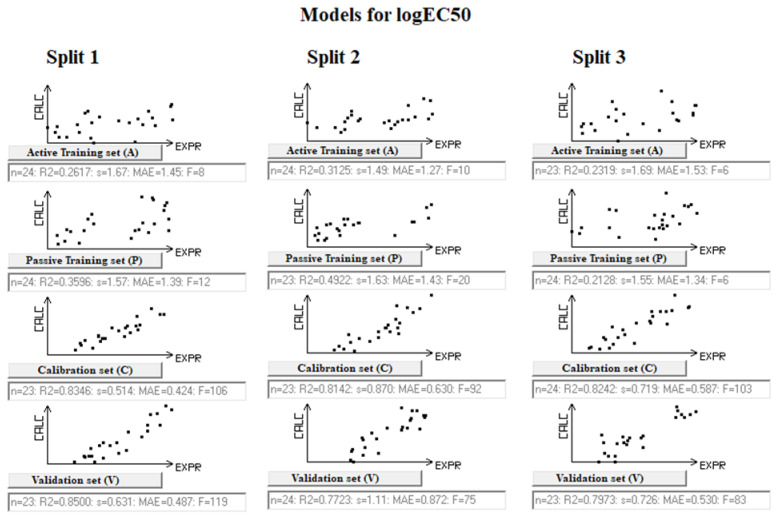
Splitting correlations for the active and passive training, calibration, and validation sets for logEC50 for swollen duckweed. Black dots represent the substances in the subset.

The Monte Carlo method involves the use of some random process. The figures (Figure 1 and Figure 2) representing the constructed models as well as their statistical quality (Table 1 and Table 2) show that, nevertheless, in both cases, both the determination coefficients and the mean square errors are reproduced during the mentioned random processes. Thus, the method under consideration can be considered useful from both practical and heuristic points of view.

This study was carried out to assess the feasibility of the approach used for modeling experimental data for two endpoints, which have not been studied so far. The only example we found in the literature refers to a model for swollen duckweed, using 13 substances in total; thus, it can be considered a preliminary study, without a proper validation of the results [32]. The paucity of existing models demonstrates the need to generate new models for these poorly represented endpoints. However, it should be noted that it is possible to expand the search space of this modeling system by assessing mechanistic interpretations and deeper analysis of the reproducibility of the results of the described random processes on large datasets. This is what is planned to be carried out in the near future.

## 5. Conclusions

We introduced new QSAR models for two endpoints of ecotoxicological interest for the first time. The data came from the EFSA database OpenFoodTox. This illustrates the easy way to exploit data that are more and more frequently available to protect our environment by better considering a larger set of species than those usually addressed. We used CORAL software to obtain models, which simply need the SMILES, without calculating molecular descriptors. We investigated different ways to optimize the results using new features of the CORAL software. The *IIC* and *CII* components of the target function are significant, since without these, the models are not satisfactory (their predictive potentials are low). These models will be implemented in the VEGAHUB website (www.vegahub.eu; accessed on 11 June 2024) for free use.

## Figures and Tables

**Table 1:** The statistical characteristics of model logNOEC for the harlequin fly. A, P, C, and V are active training, passive training, calibration, and validation sets.

Split	Set	*n*	R^2^ *	IIC	CII	Q^2^	RMSE	MAE	F
1	A	31	0.4396	0.5460	0.7386	0.3694	1.51	1.34	23
	P	30	0.4186	0.5630	0.7060	0.3122	1.64	1.38	20
	C	31	0.6166	0.7847	0.8329	0.5328	0.833	0.650	47
	V	30	0.7449	-	-	-	0.686	-	-
2	A	32	0.4427	0.6654	0.6927	0.3590	1.43	1.26	24
	P	31	0.4611	0.6557	0.7281	0.3753	1.41	1.28	25
	C	30	0.8037	0.8963	0.8684	0.7825	0.449	0.360	115
	V	29	0.7234	-	-	-	0.563	-	-
3	A	31	0.5874	0.7185	0.7339	0.5350	1.07	0.905	41
	P	30	0.2601	0.4773	0.7201	0.1510	2.03	1.74	10
	C	30	0.6627	0.8140	0.7829	0.6116	0.968	0.727	55
	V	31	0.7295	-	-	-	0.833	-	-

* Please see Section 2.3 for the description of the statistical parameters.

**Table 2:** The statistical characteristics on model logEC50. A, P, C, and V are active training, passive training, calibration, and validation sets.

Split	Set	*n*	R^2^ *	IIC	CII	Q^2^	RMSE	MAE	F
1	A	24	0.2617	0.4329	0.7068	0.1210	1.67	1.45	8
	P	24	0.3596	0.5931	0.7166	0.2660	1.57	1.39	12
	C	23	0.8346	0.9131	0.9073	0.7980	0.514	0.424	106
	V	23	0.8500	-	-	-	0.631	-	-
2	A	24	0.3125	0.4730	0.7386	0.2229	1.49	1.27	10
	P	23	0.4922	0.4969	0.7320	0.4104	1.63	1.43	20
	C	23	0.8142	0.9020	0.8767	0.7793	0.870	0.630	92
	V	24	0.7723	-	-	-	1.113	-	-
3	A	23	0.2319	0.4414	0.7751	0.0977	1.69	1.53	6
	P	24	0.2128	0.2435	0.7330	0.0633	1.55	1.34	6
	C	24	0.8242	0.9078	0.9021	0.7888	0.719	0.587	103
	V	23	0.7973	-	-	-	0.726	-	-

* Please see Section 2.3 for the description of the statistical parameters.

## Data Availability

Data regarding the models are available in Supplementary Materials.

## Supplementary Materials

The following supporting information can be downloaded at: https://www.mdpi.com/article/10.3390/toxics12060425/s1, Table S1: information on the compounds (ID, CAS number, name, and SMILES) and the experimental values used for the EC50 model of swollen duckweed. Table S2: information on the compounds (ID, CAS number, name, and SMILES) and the experimental values used for the NOEC model of the harlequin fly. Table S3: the statistical details on the model for NOEC for harlequin fly (*Chirinomus riparius*). Table S4: the experimental and calculated values and the applicability domain of NOEC for the harlequin fly (*Chirinomus riparius*) for the model obtained in the case of split 1. Table S5: the statistical details on the model for EC50 for swollen duckweed (*Lemna gibba*). Table S6: the experimental and calculated values and the applicability domain of EC50 for swollen duckweed (*Lemna gibba*) for the model obtained in the case of split 1. The folder “2D chemical structures” contains the 2D chemical structures of the compounds used for the development of the models. Each pdf file has the name of the compound ID.
