# Supplementary figures and images for: Models for the No-Observed-Effect Concentration (NOEC) and Maximal Half-Effective Concentration (EC50) (part 2 of 2)
Source: Toxics. 2024 Jun 12;12(6):425. doi: 10.3390/toxics12060425 (PMC11209108; doi:10.3390/toxics12060425)

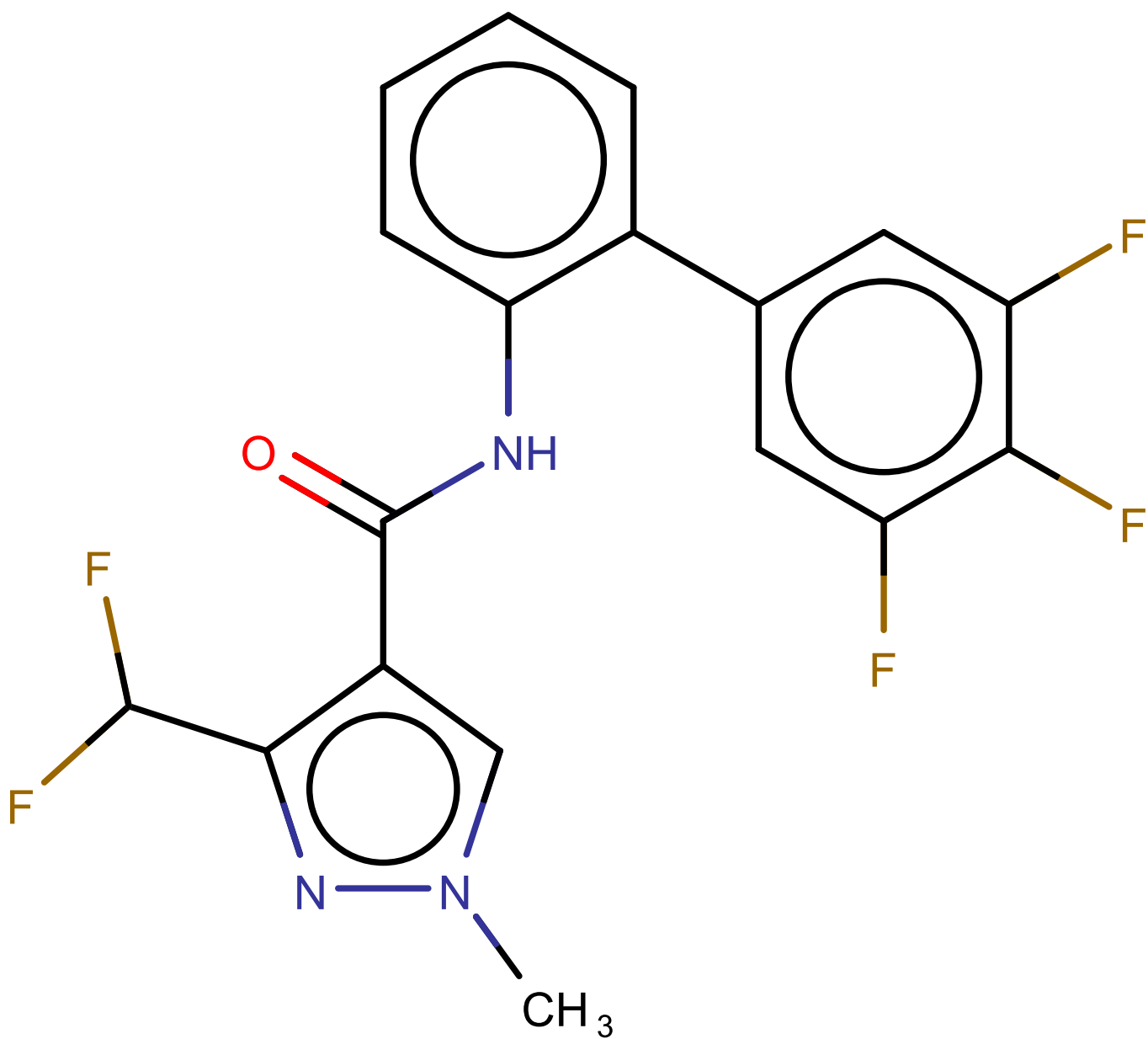

Supplement: Supplementary file 1 [file toxics-12-00425-s001.zip › Supplementary Materials/2D chemical structures/2043.pdf]

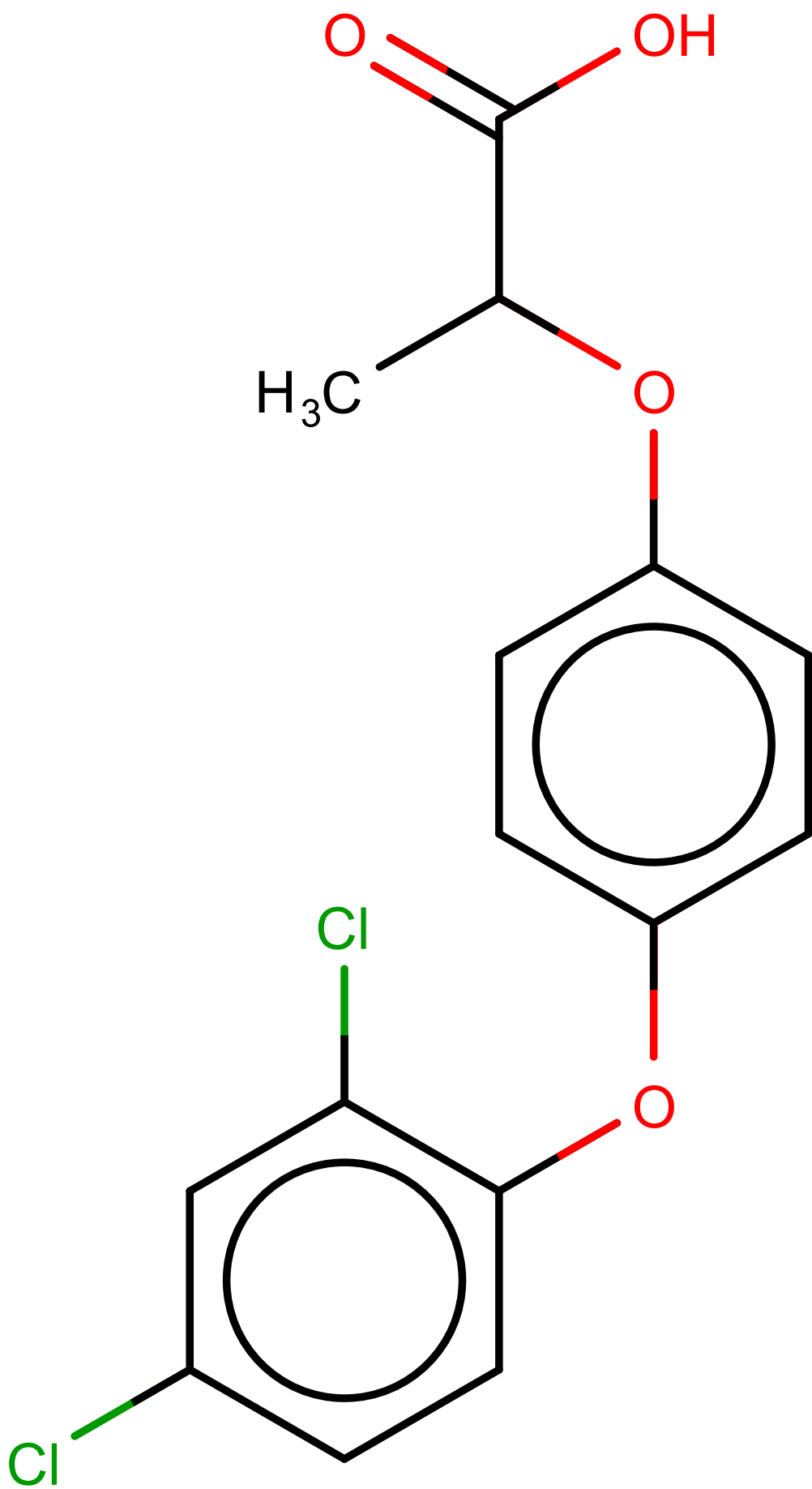

Supplement: Supplementary file 1 [file toxics-12-00425-s001.zip › Supplementary Materials/2D chemical structures/2109.pdf]

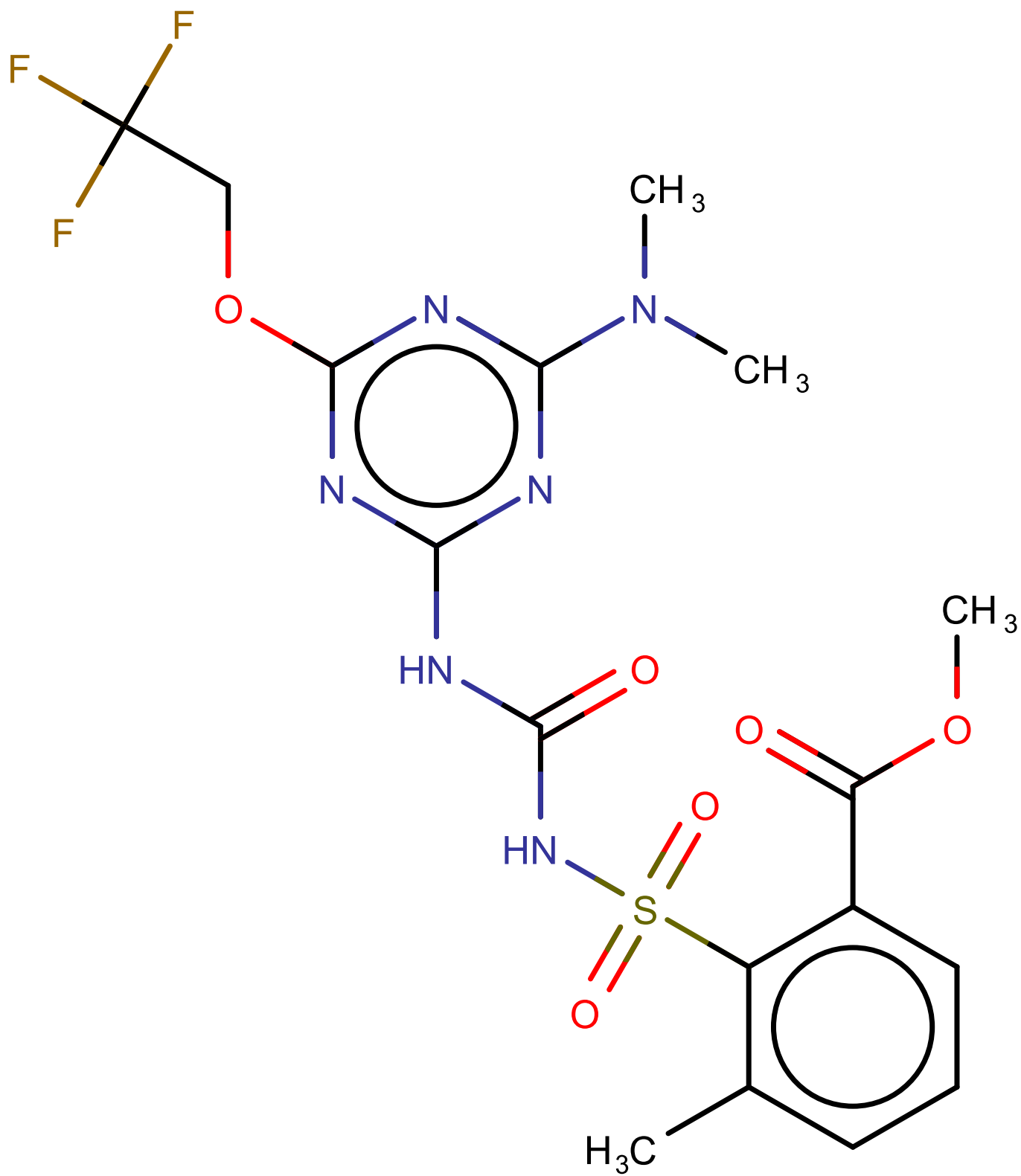

Supplement: Supplementary file 1 [file toxics-12-00425-s001.zip › Supplementary Materials/2D chemical structures/2123.pdf]

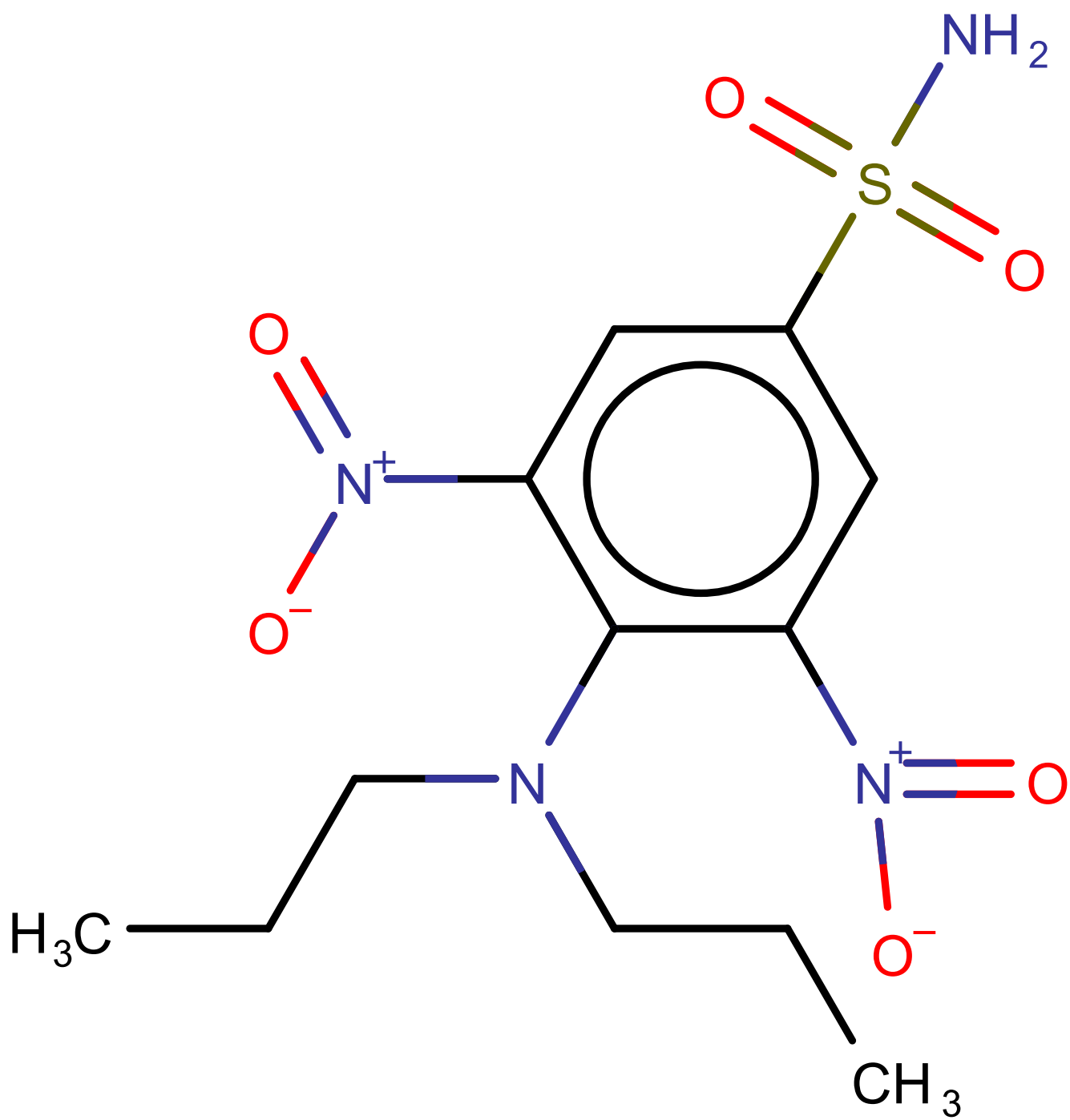

Supplement: Supplementary file 1 [file toxics-12-00425-s001.zip › Supplementary Materials/2D chemical structures/2148.pdf]

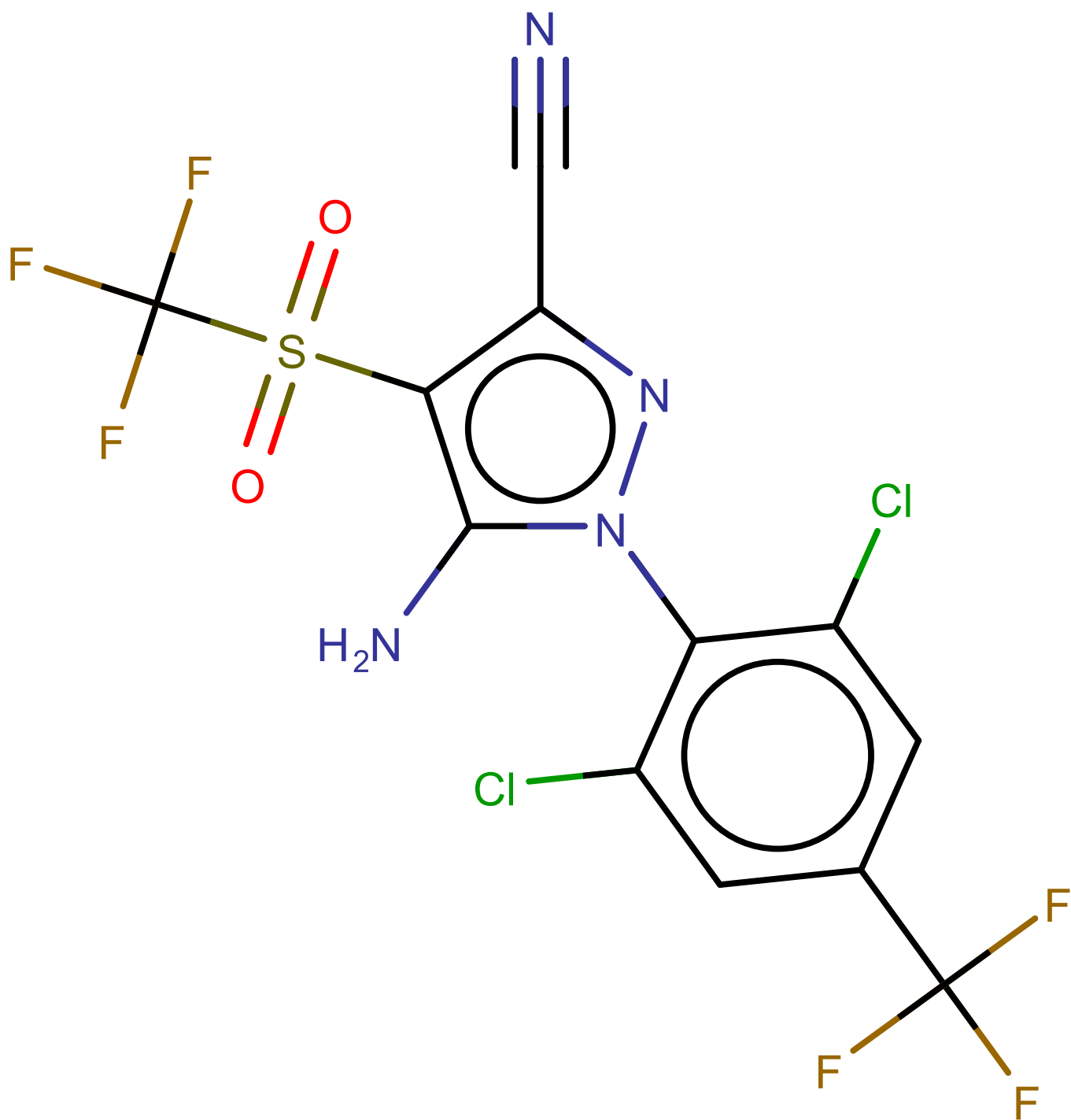

Supplement: Supplementary file 1 [file toxics-12-00425-s001.zip › Supplementary Materials/2D chemical structures/2212.pdf]

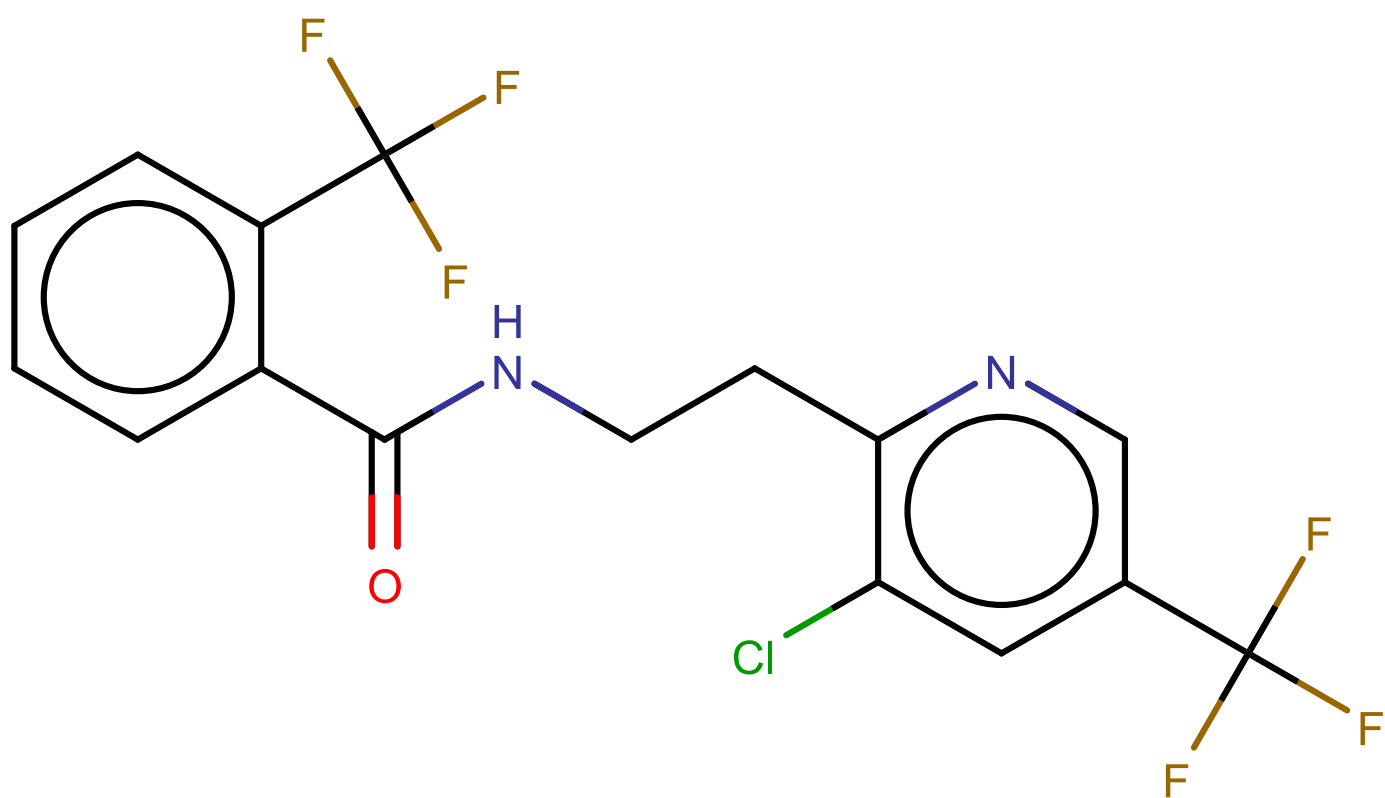

Supplement: Supplementary file 1 [file toxics-12-00425-s001.zip › Supplementary Materials/2D chemical structures/2408.pdf]

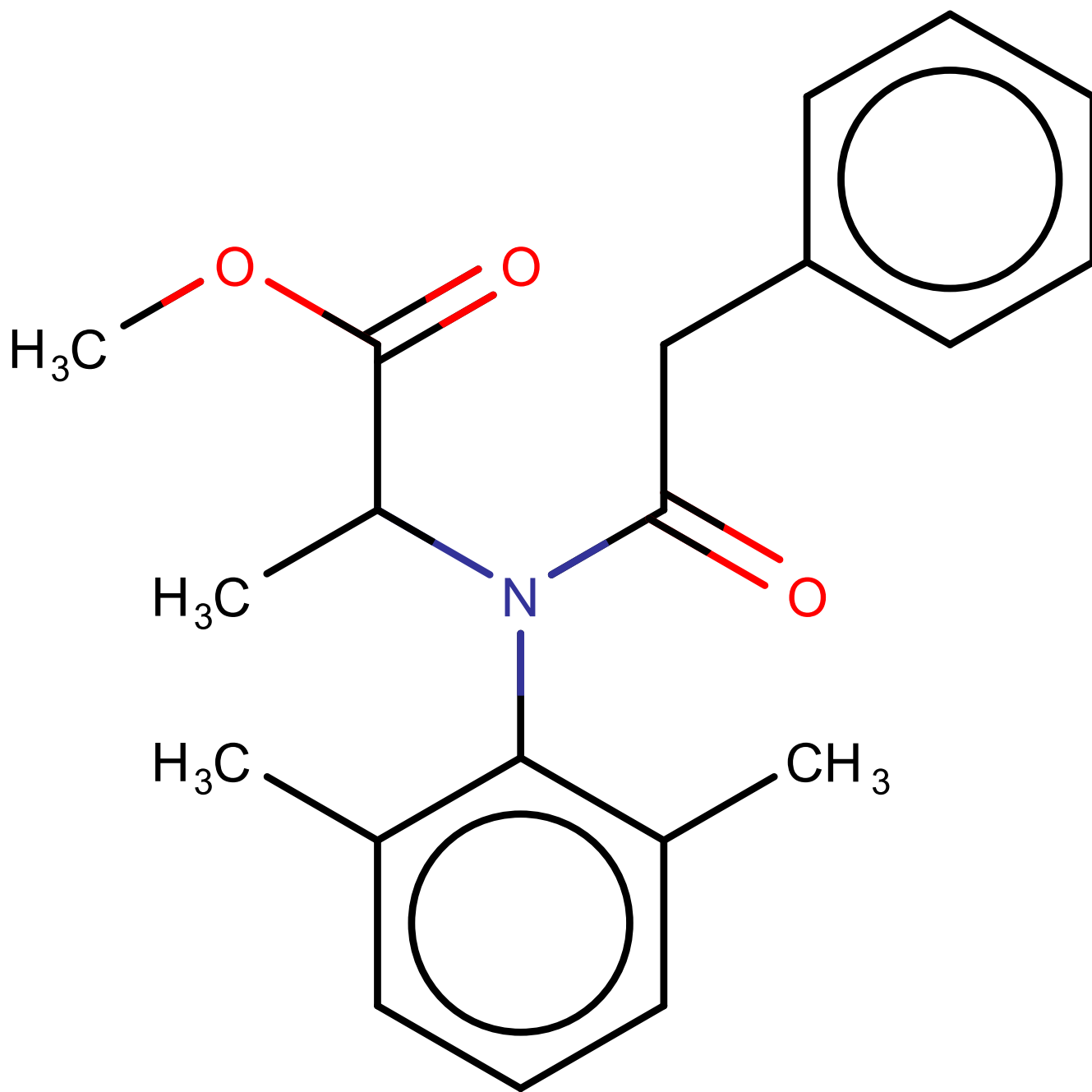

Supplement: Supplementary file 1 [file toxics-12-00425-s001.zip › Supplementary Materials/2D chemical structures/2983.pdf]

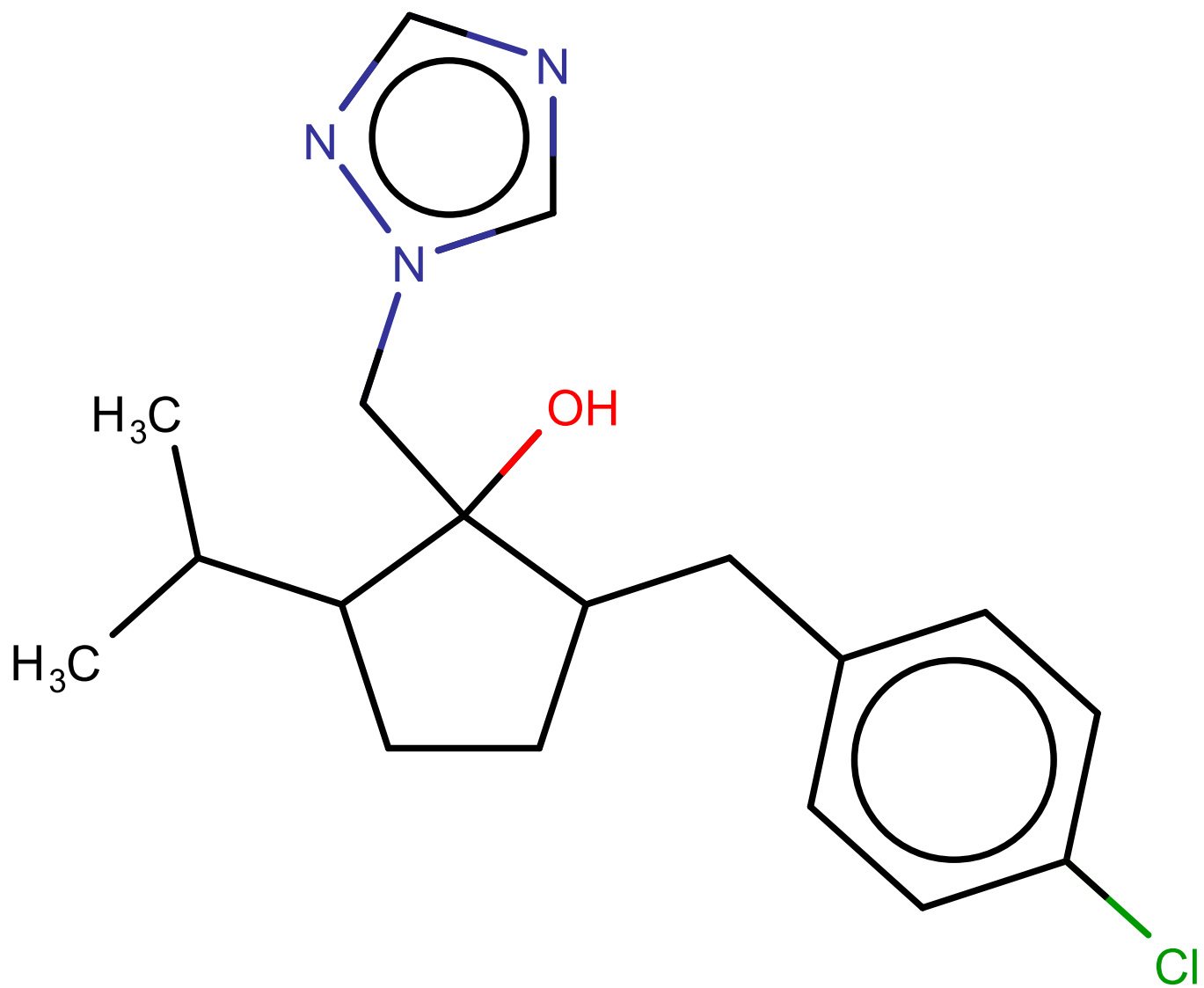

Supplement: Supplementary file 1 [file toxics-12-00425-s001.zip › Supplementary Materials/2D chemical structures/2994.pdf]

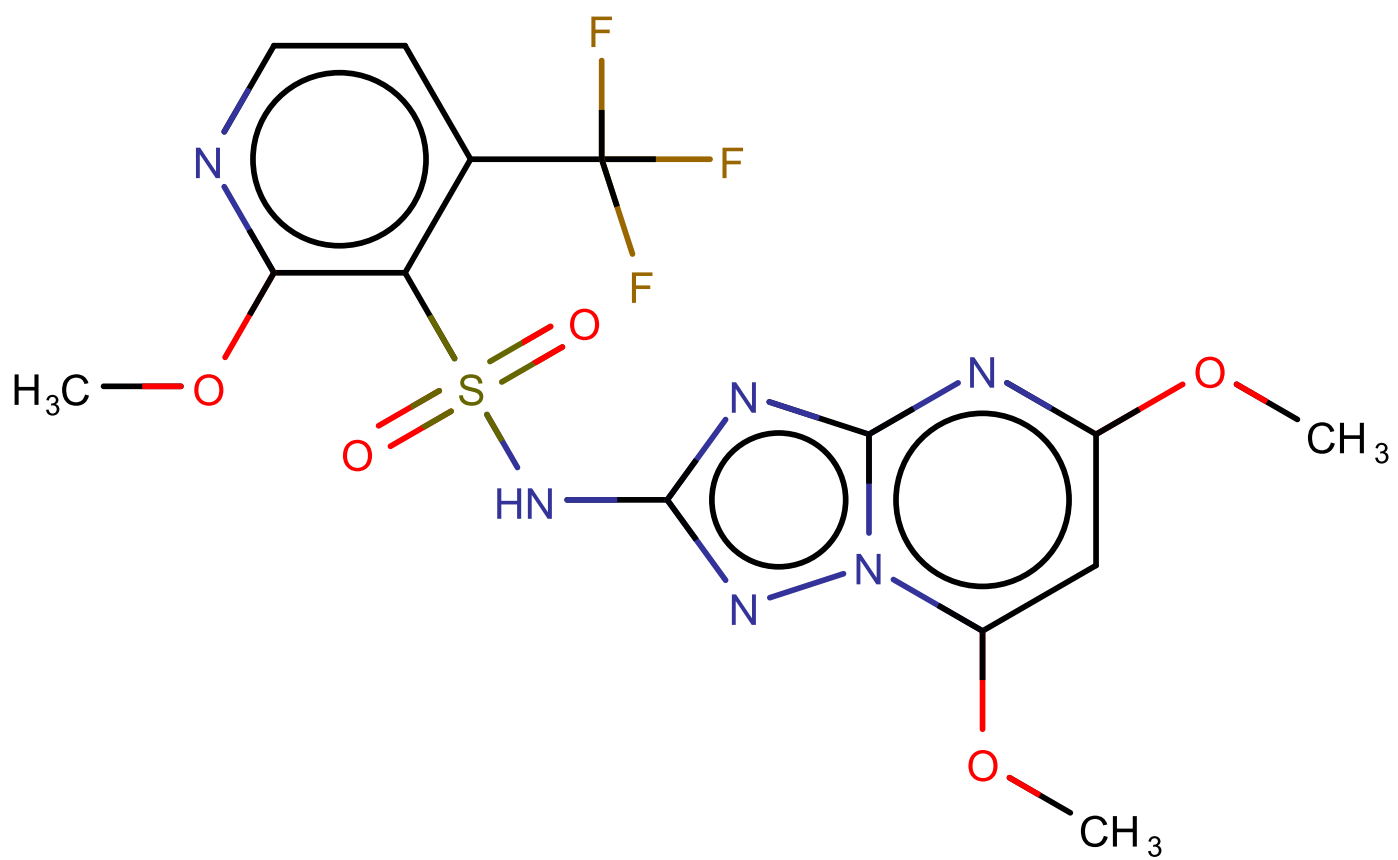

Supplement: Supplementary file 1 [file toxics-12-00425-s001.zip › Supplementary Materials/2D chemical structures/2995.pdf]

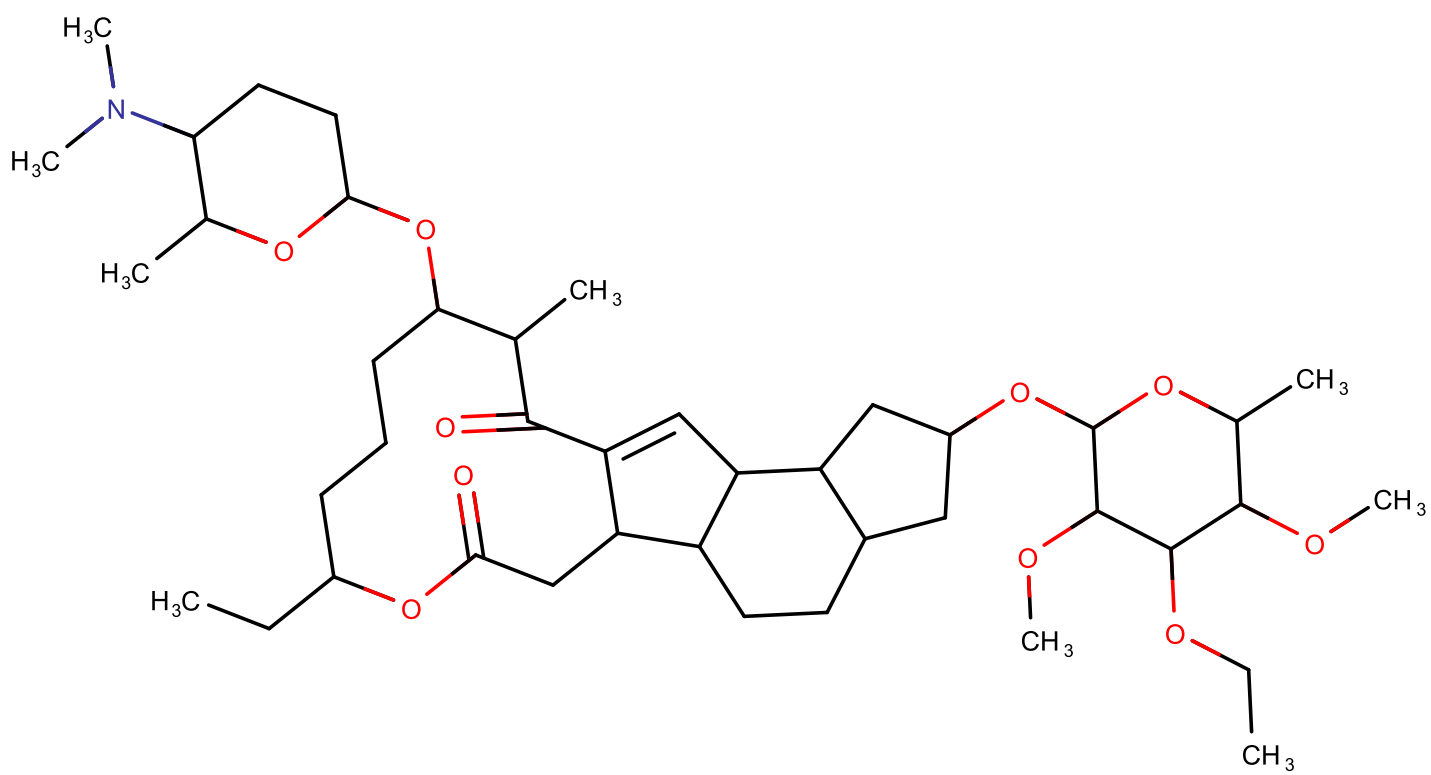

Supplement: Supplementary file 1 [file toxics-12-00425-s001.zip › Supplementary Materials/2D chemical structures/3007.pdf]

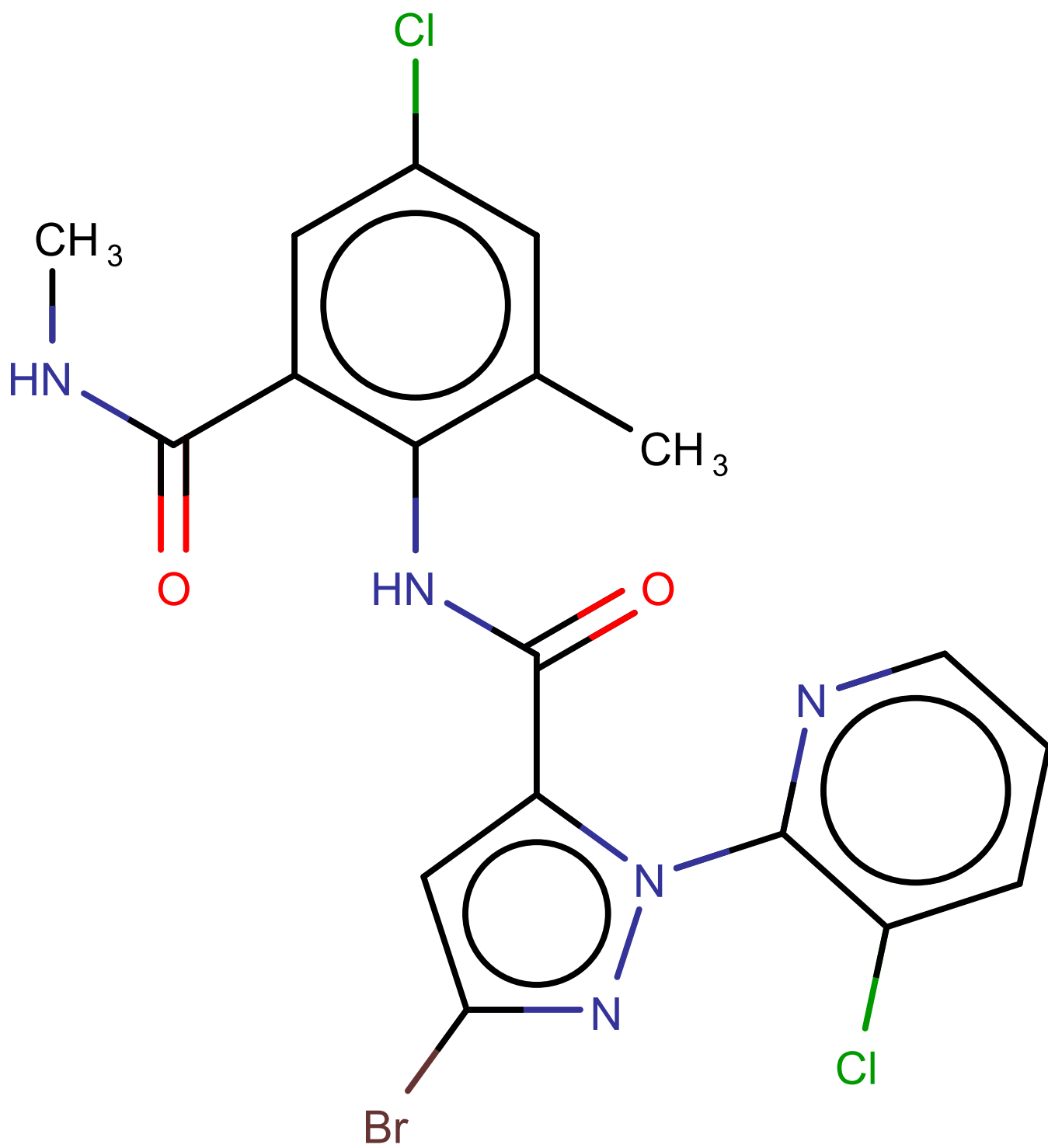

Supplement: Supplementary file 1 [file toxics-12-00425-s001.zip › Supplementary Materials/2D chemical structures/4219.pdf]

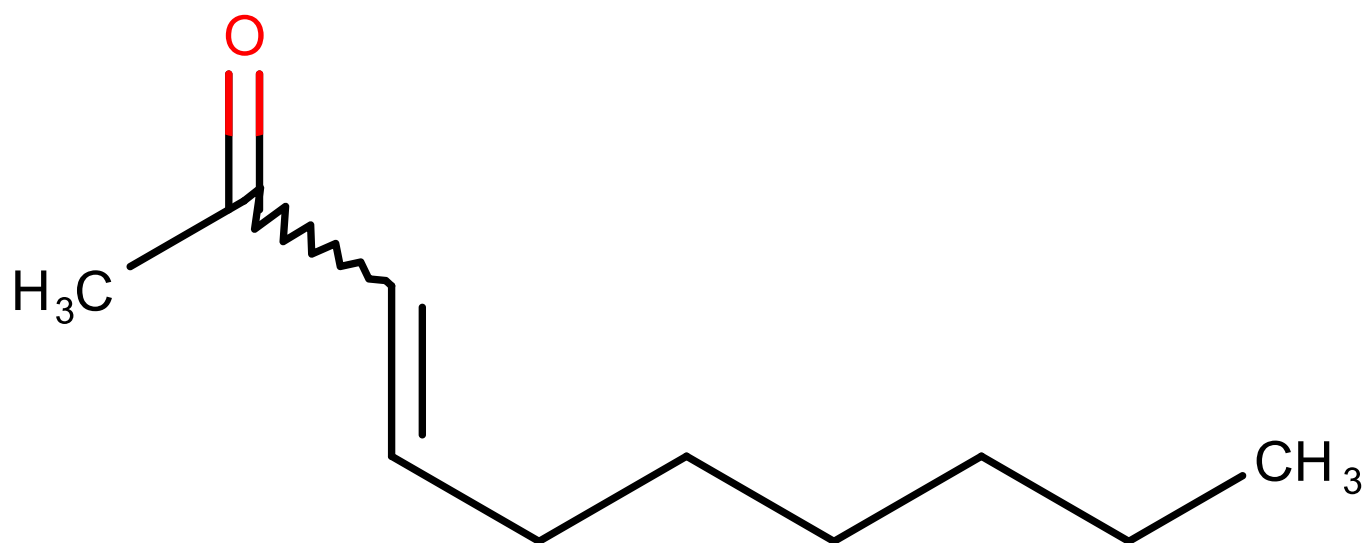

Supplement: Supplementary file 1 [file toxics-12-00425-s001.zip › Supplementary Materials/2D chemical structures/4374.pdf]

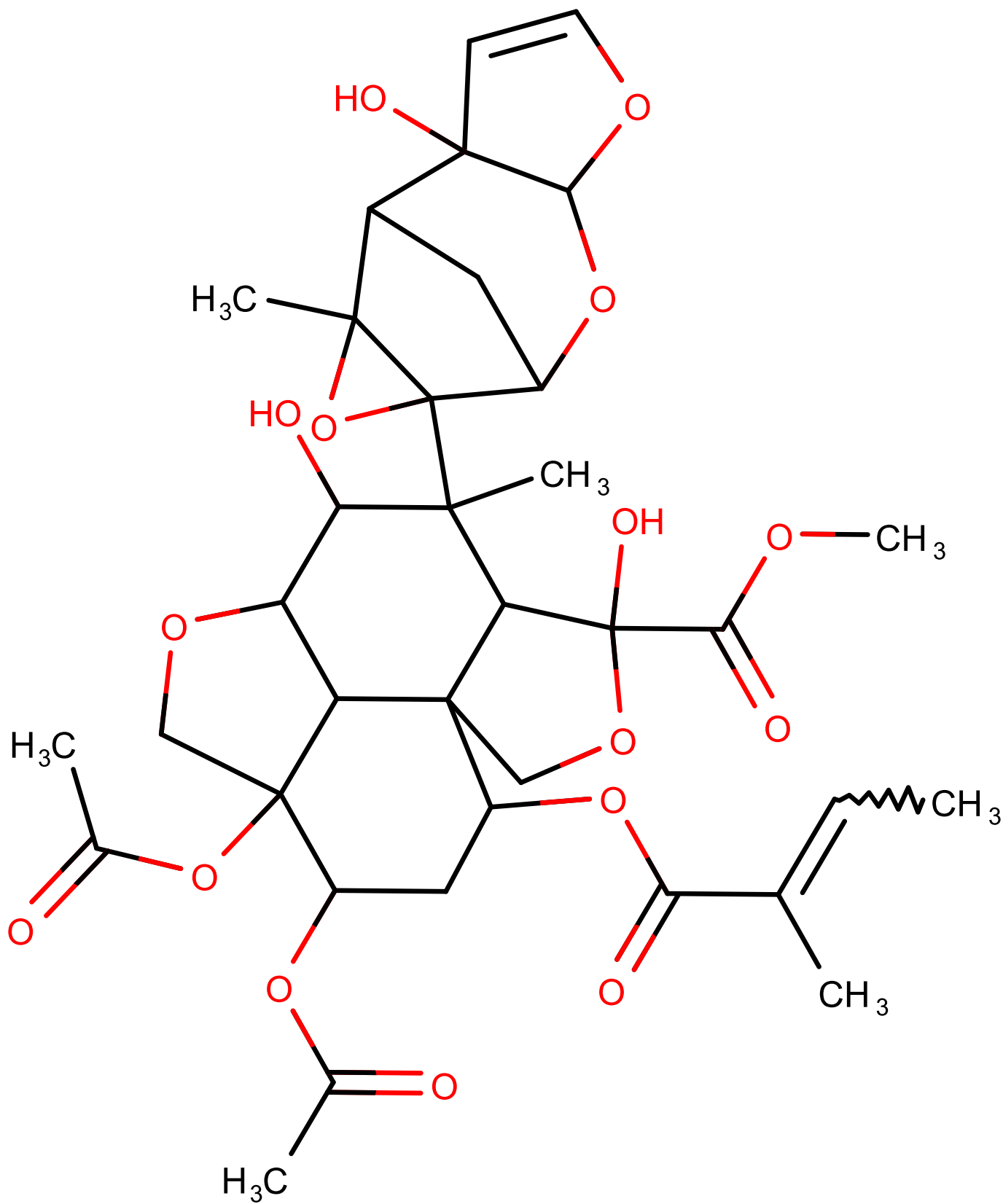

Supplement: Supplementary file 1 [file toxics-12-00425-s001.zip › Supplementary Materials/2D chemical structures/4474.pdf]

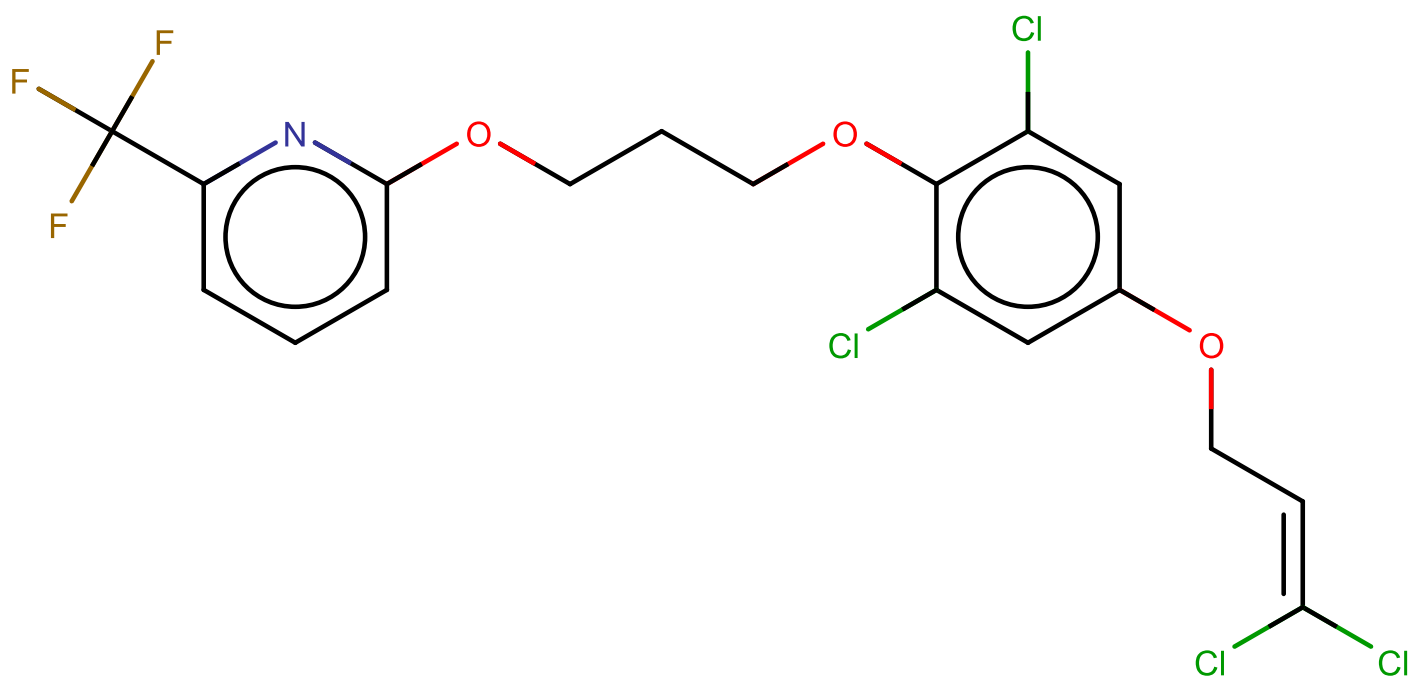

Supplement: Supplementary file 1 [file toxics-12-00425-s001.zip › Supplementary Materials/2D chemical structures/4495.pdf]

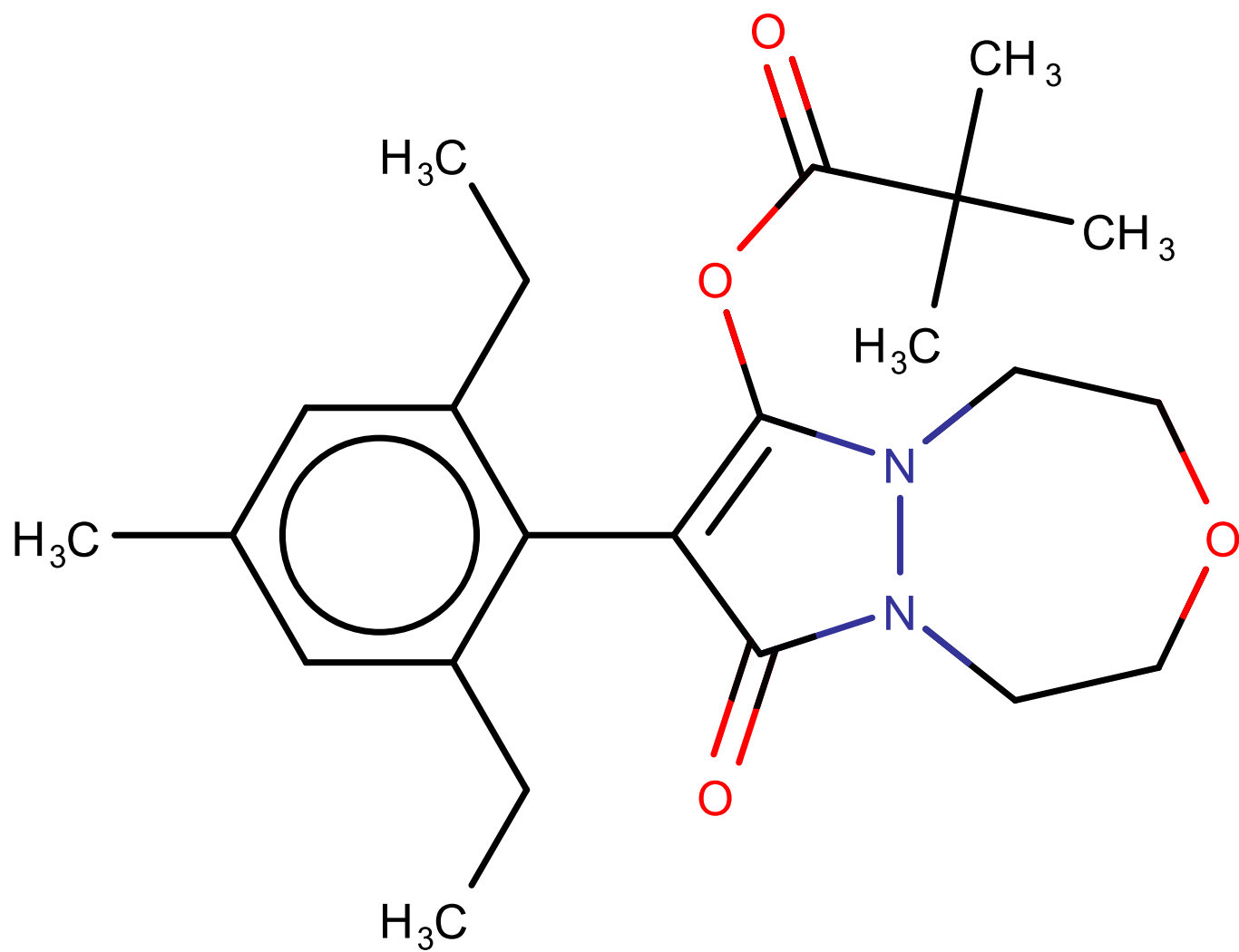

Supplement: Supplementary file 1 [file toxics-12-00425-s001.zip › Supplementary Materials/2D chemical structures/4496.pdf]

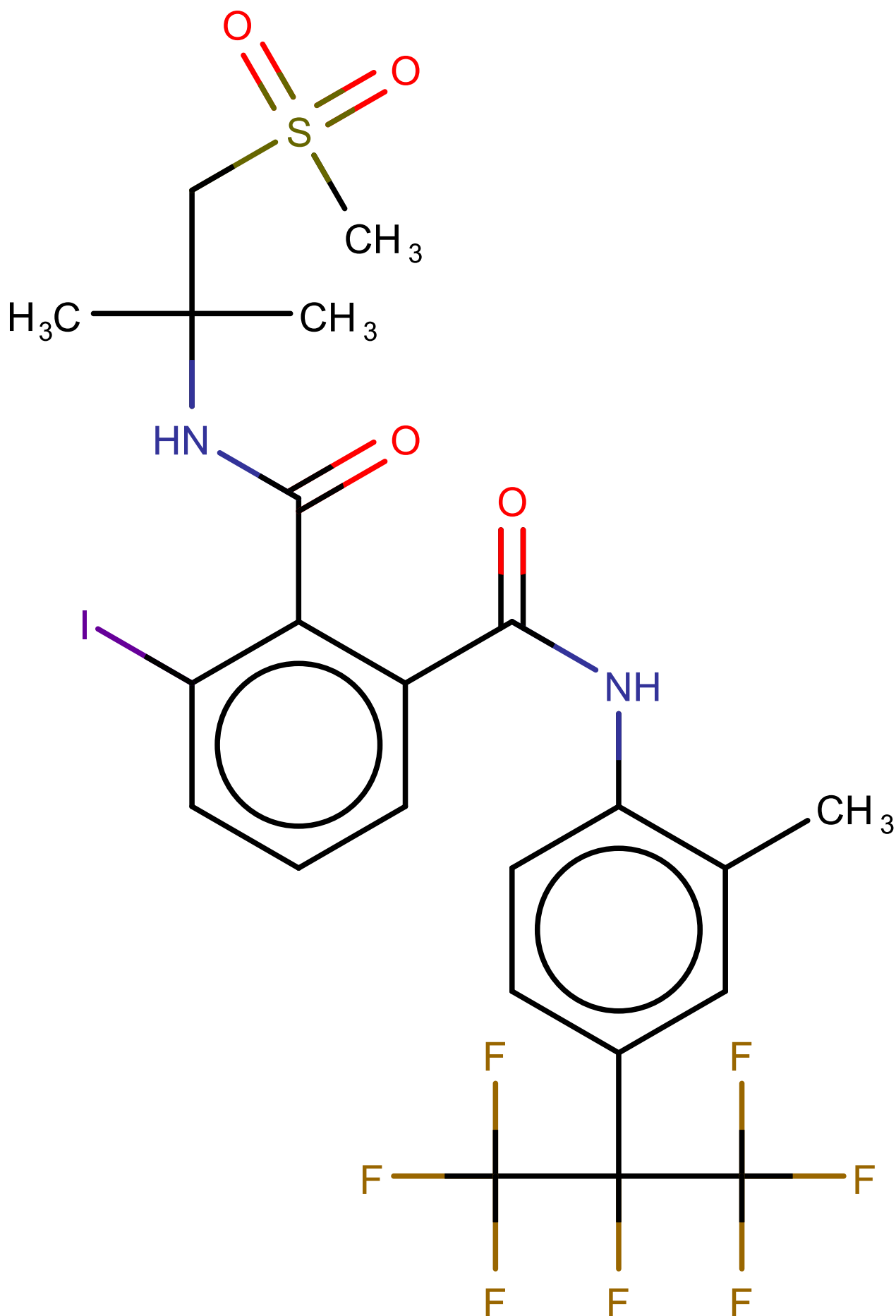

Supplement: Supplementary file 1 [file toxics-12-00425-s001.zip › Supplementary Materials/2D chemical structures/4497.pdf]

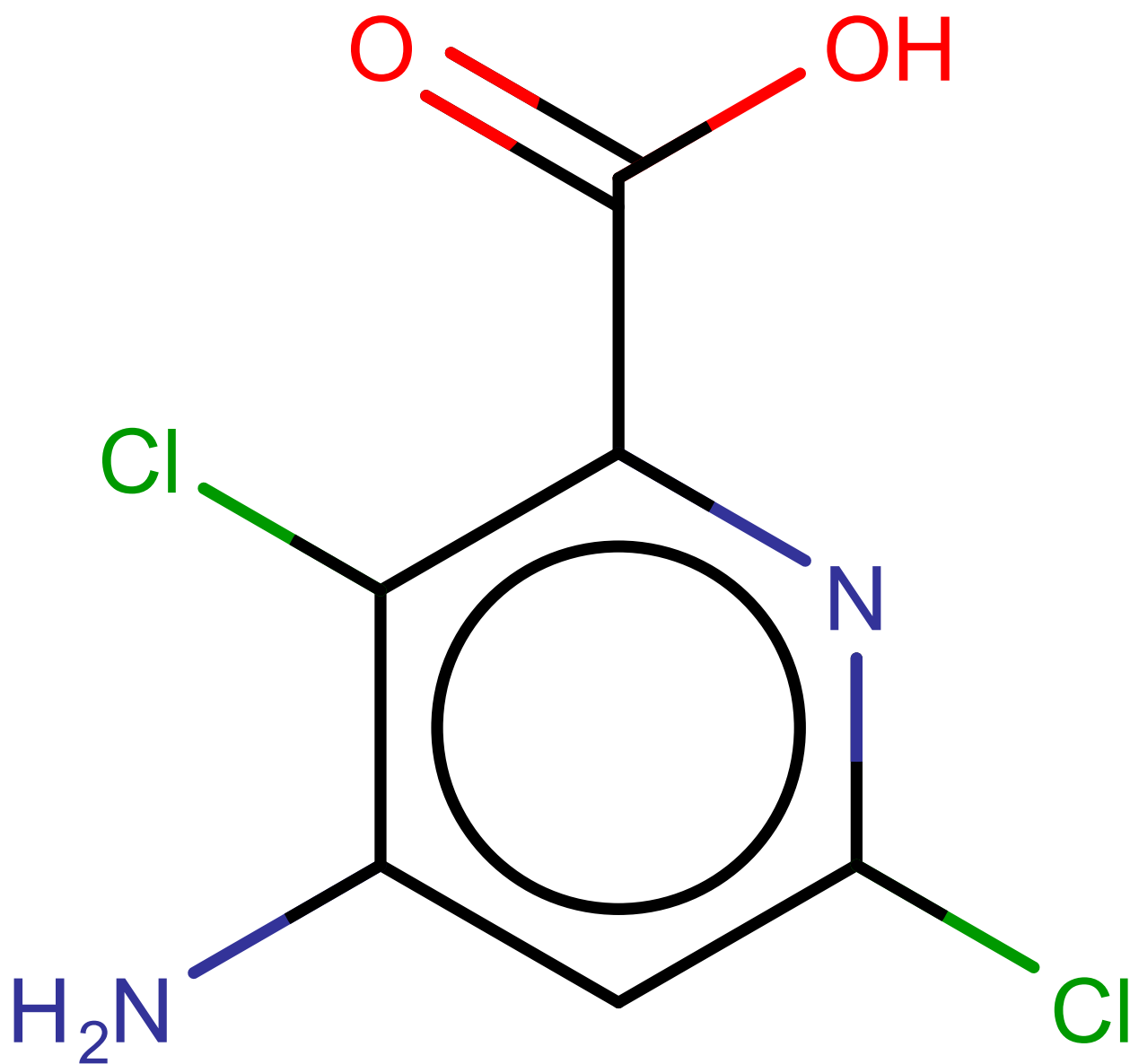

Supplement: Supplementary file 1 [file toxics-12-00425-s001.zip › Supplementary Materials/2D chemical structures/4551.pdf]

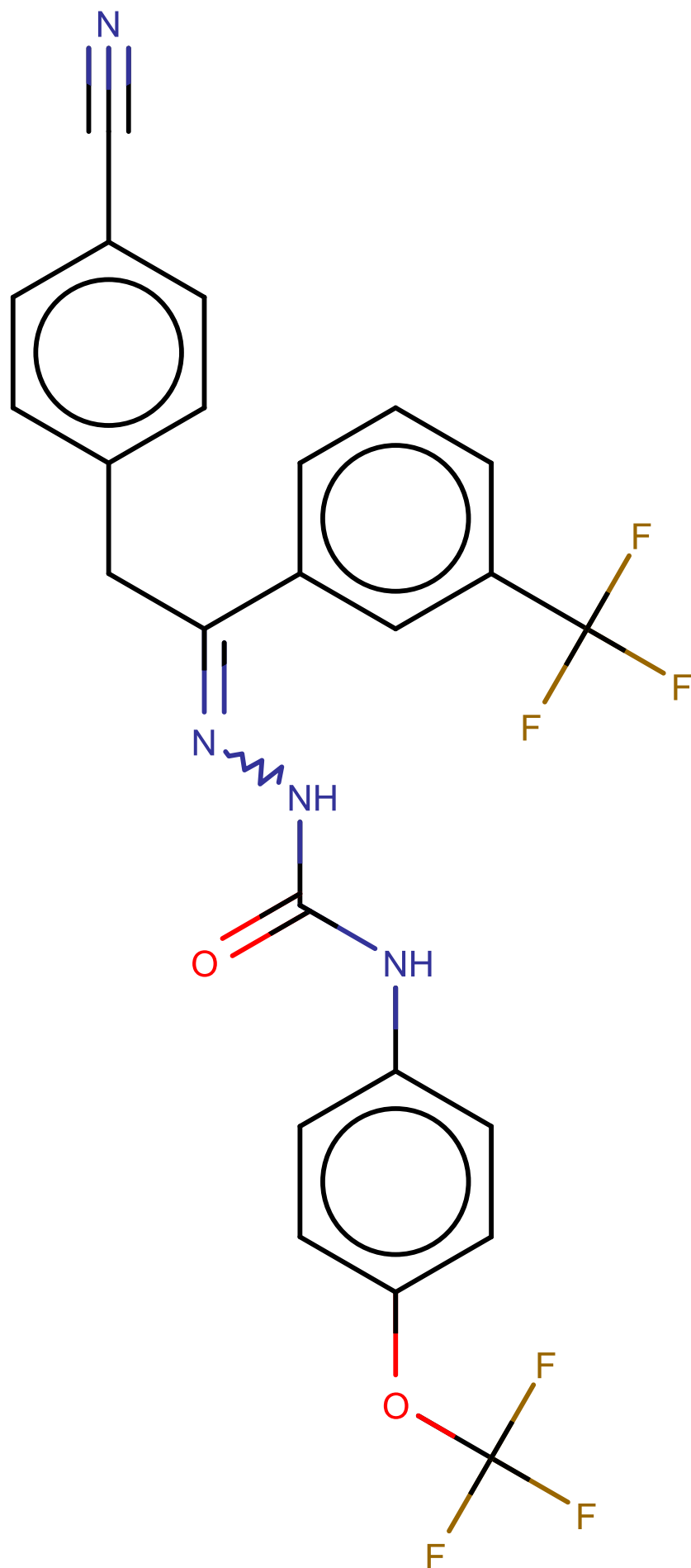

Supplement: Supplementary file 1 [file toxics-12-00425-s001.zip › Supplementary Materials/2D chemical structures/4552.pdf]

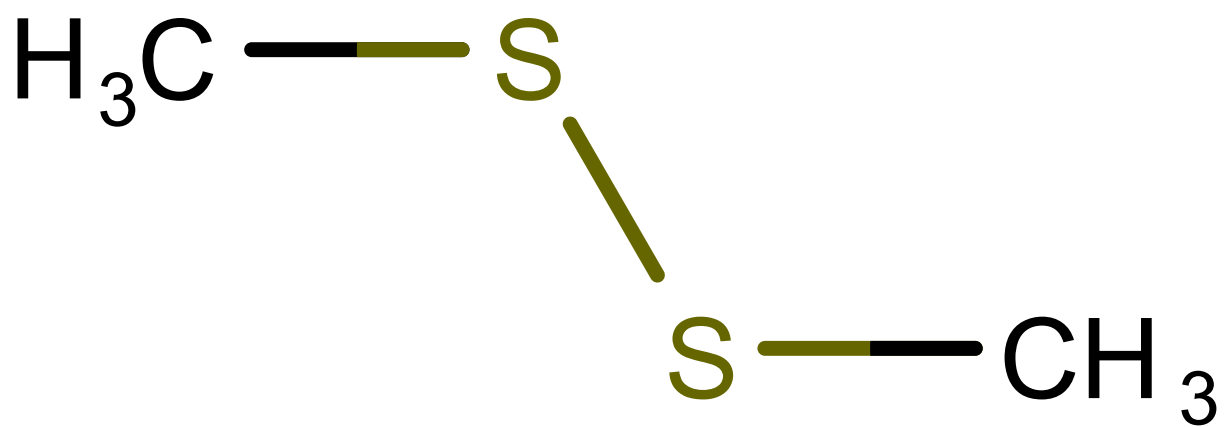

Supplement: Supplementary file 1 [file toxics-12-00425-s001.zip › Supplementary Materials/2D chemical structures/4631.pdf]

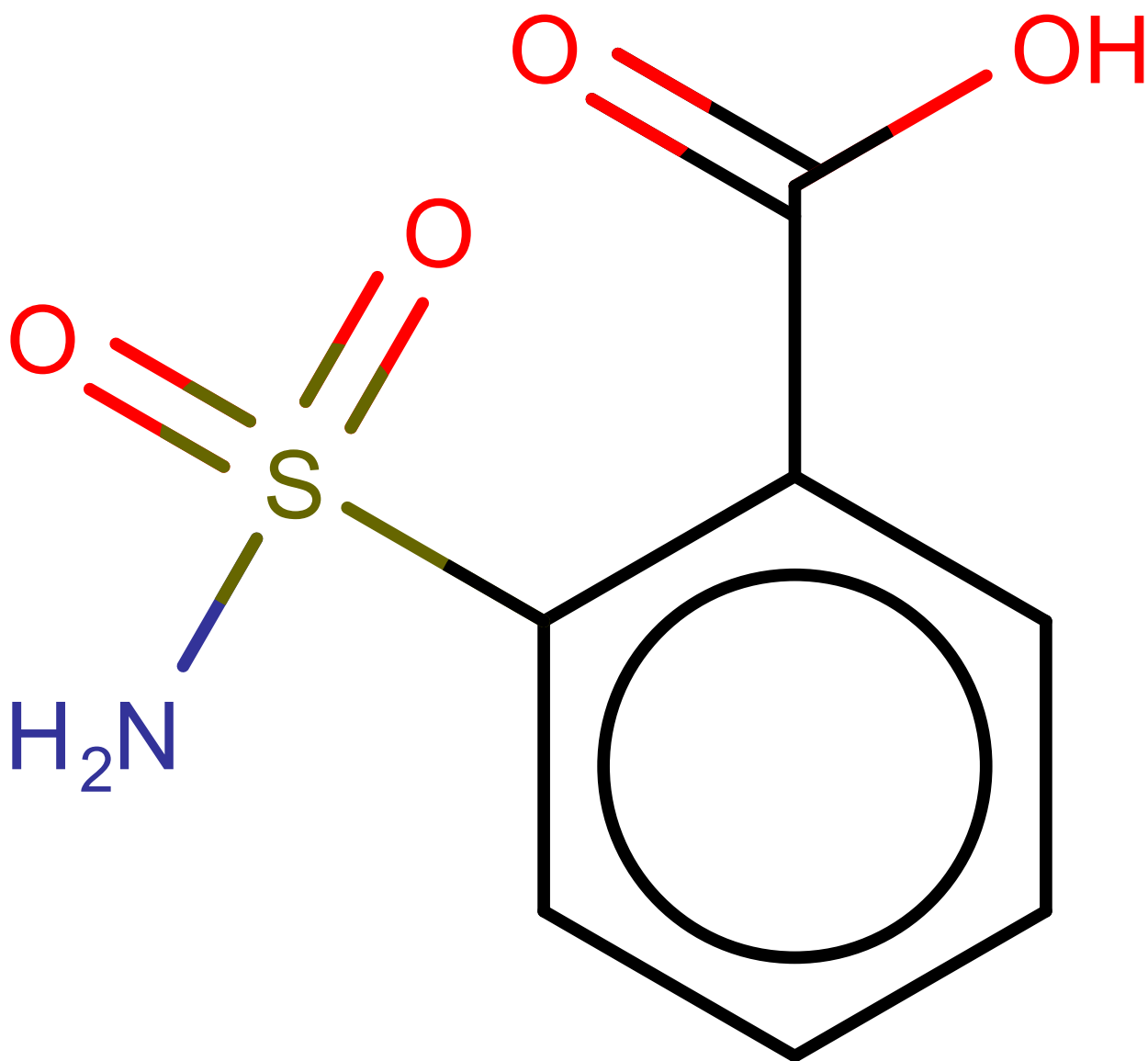

Supplement: Supplementary file 1 [file toxics-12-00425-s001.zip › Supplementary Materials/2D chemical structures/4697.pdf]

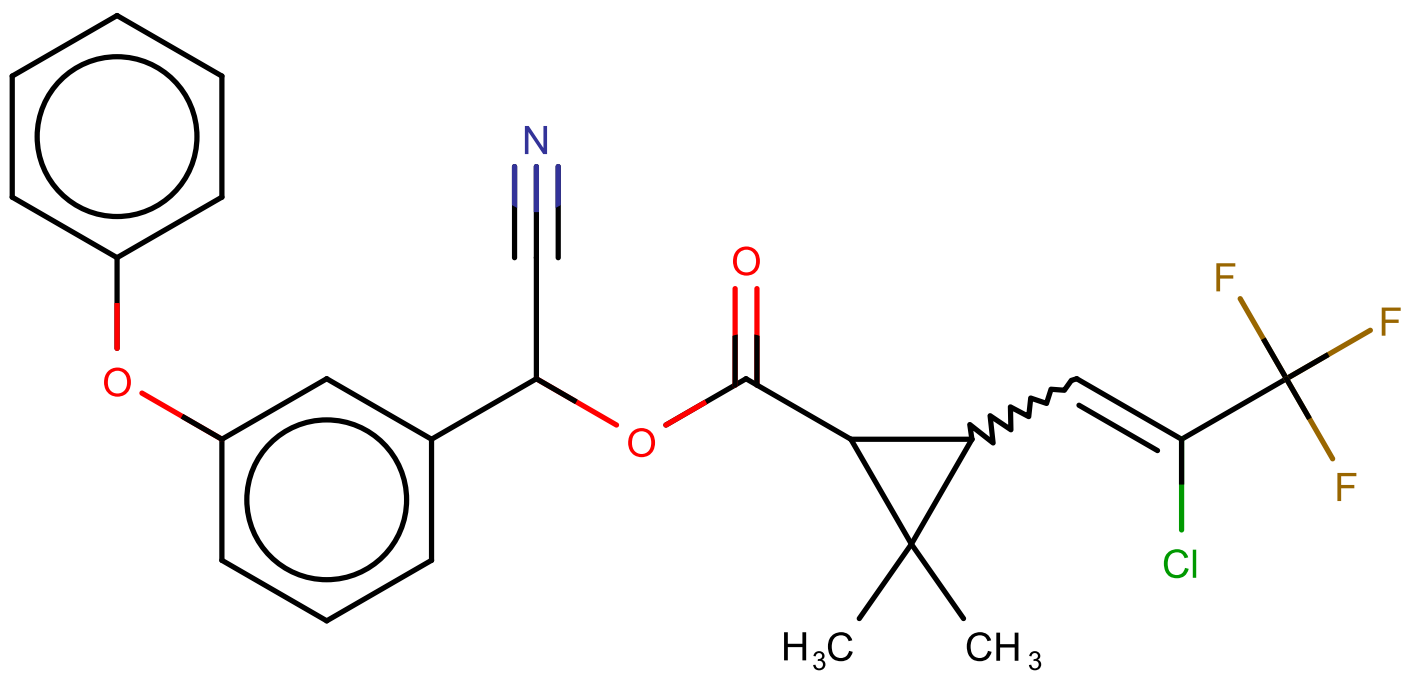

Supplement: Supplementary file 1 [file toxics-12-00425-s001.zip › Supplementary Materials/2D chemical structures/4727.pdf]

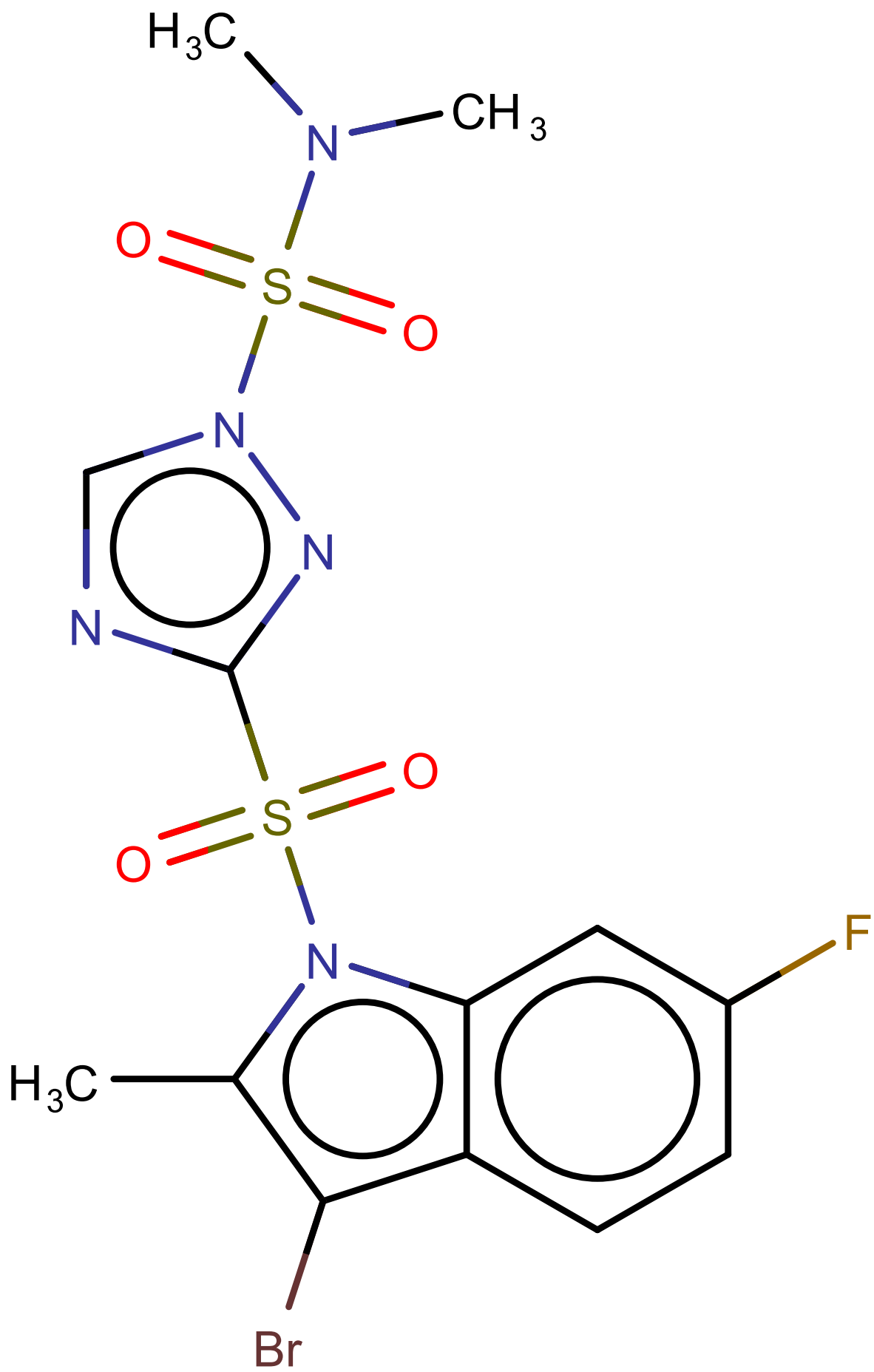

Supplement: Supplementary file 1 [file toxics-12-00425-s001.zip › Supplementary Materials/2D chemical structures/5100.pdf]

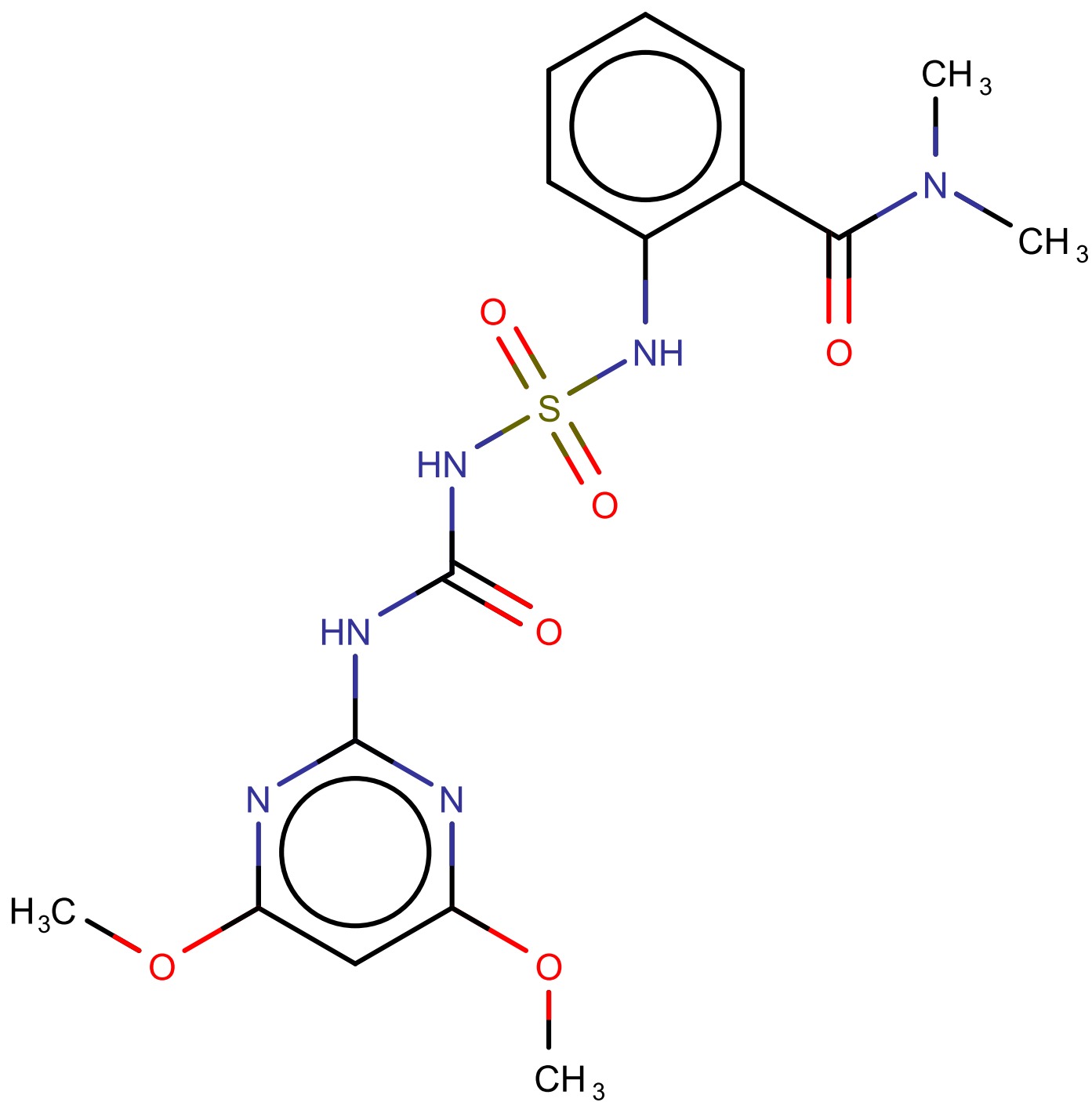

Supplement: Supplementary file 1 [file toxics-12-00425-s001.zip › Supplementary Materials/2D chemical structures/5113.pdf]

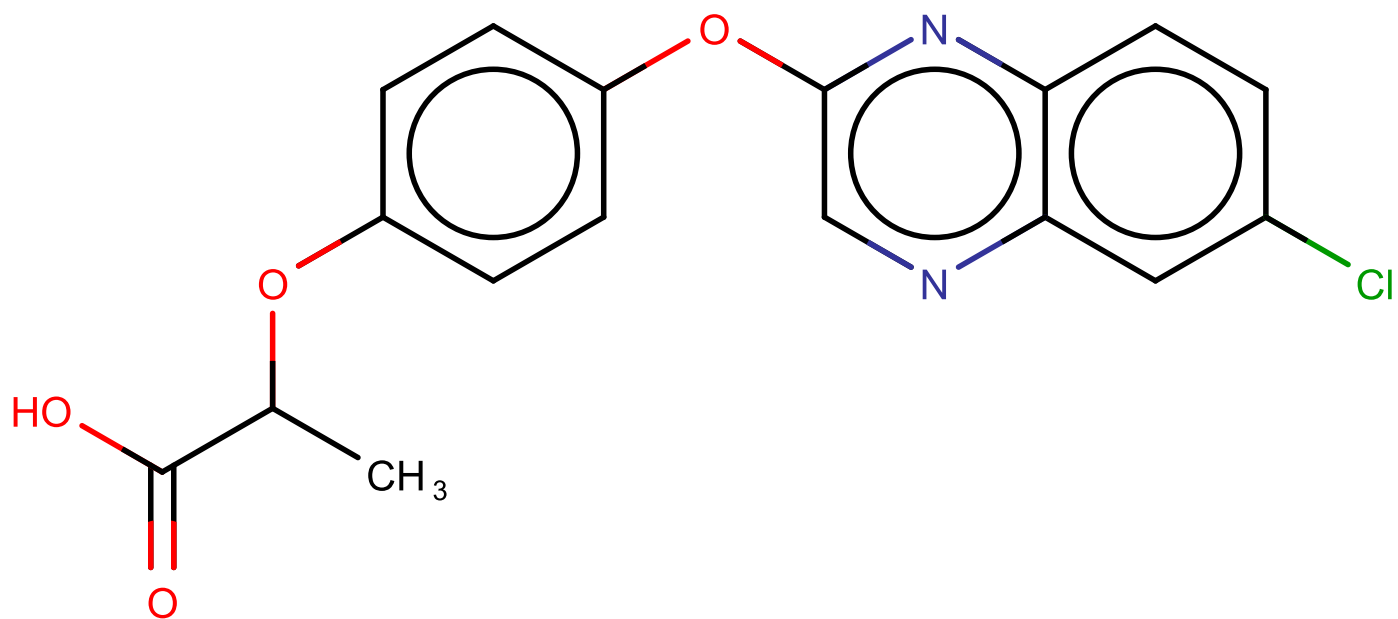

Supplement: Supplementary file 1 [file toxics-12-00425-s001.zip › Supplementary Materials/2D chemical structures/5228.pdf]

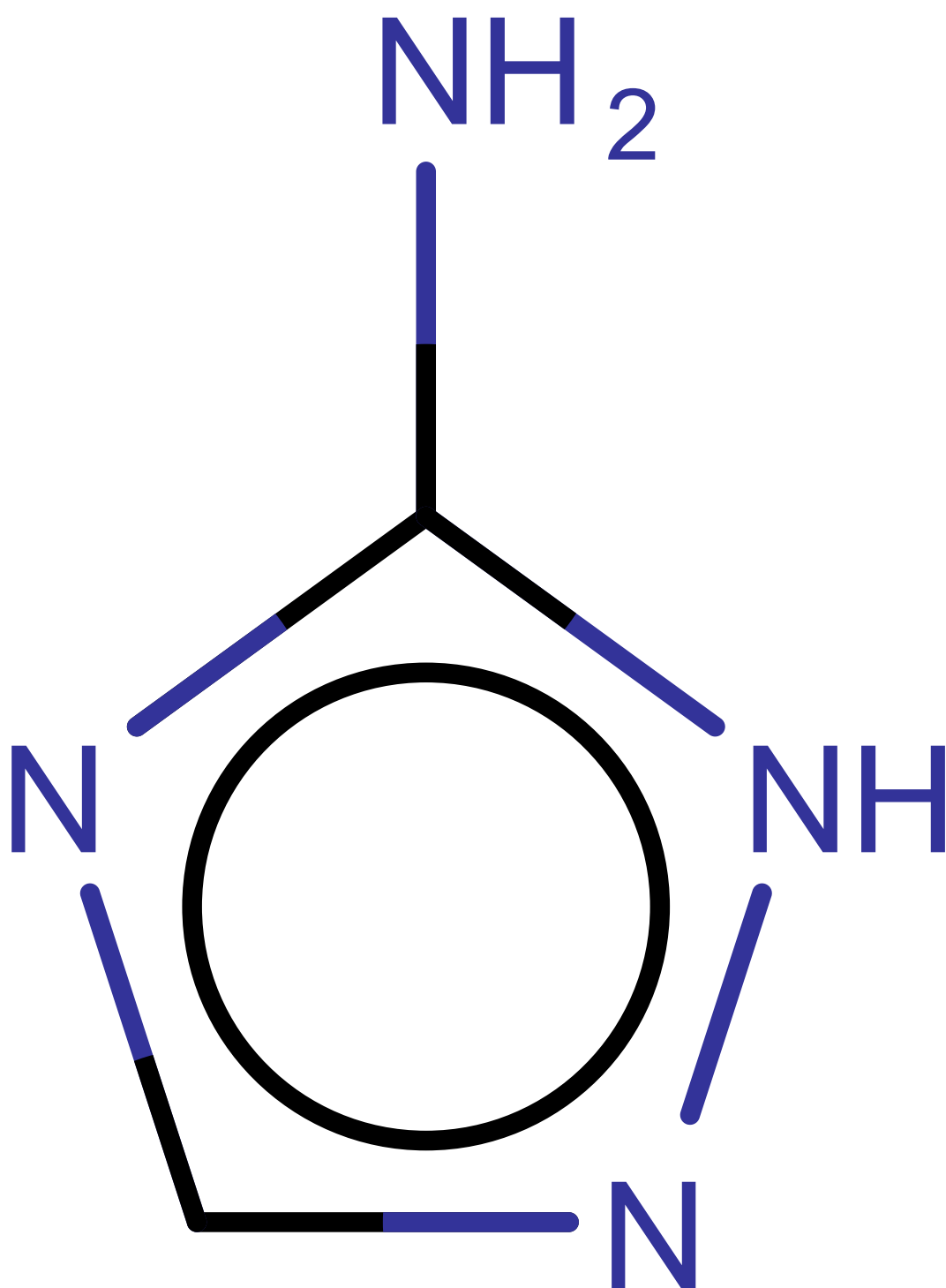

Supplement: Supplementary file 1 [file toxics-12-00425-s001.zip › Supplementary Materials/2D chemical structures/5328.pdf]

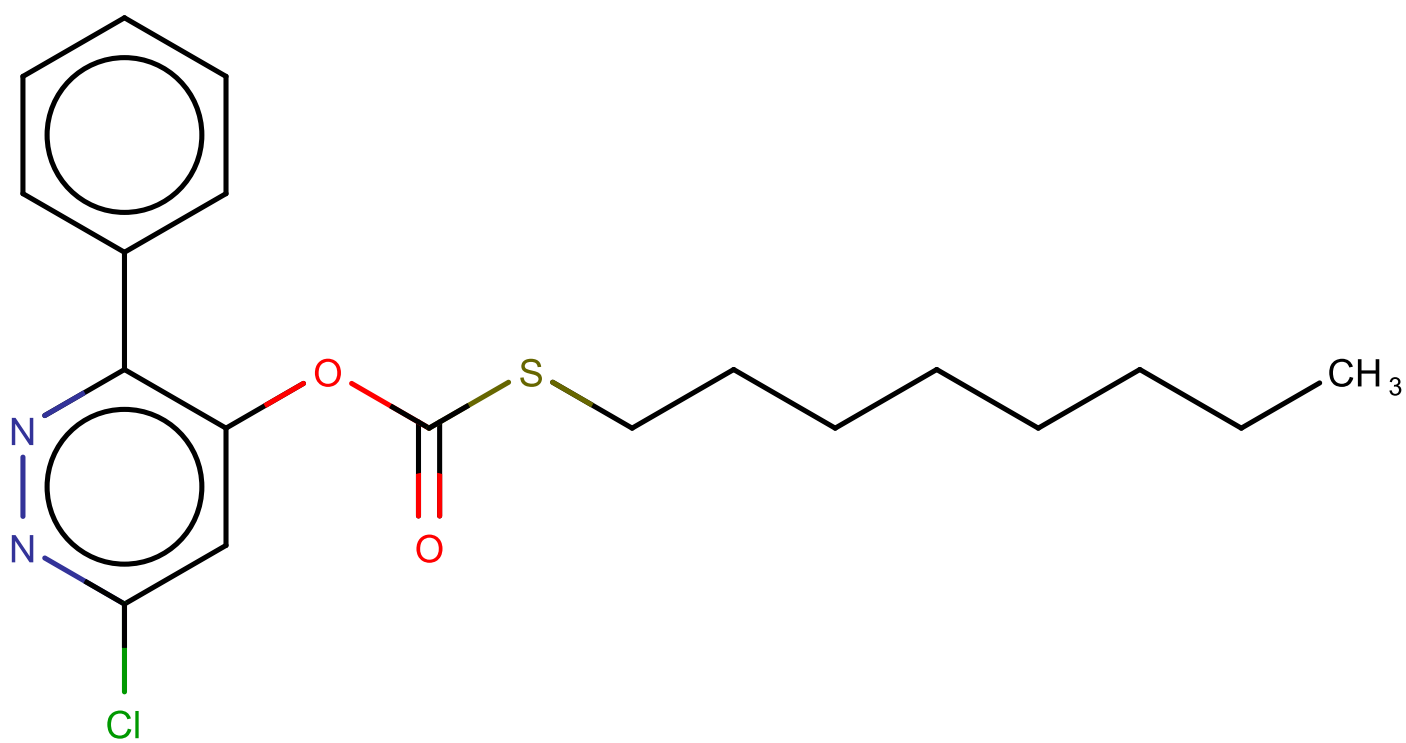

Supplement: Supplementary file 1 [file toxics-12-00425-s001.zip › Supplementary Materials/2D chemical structures/5357.pdf]

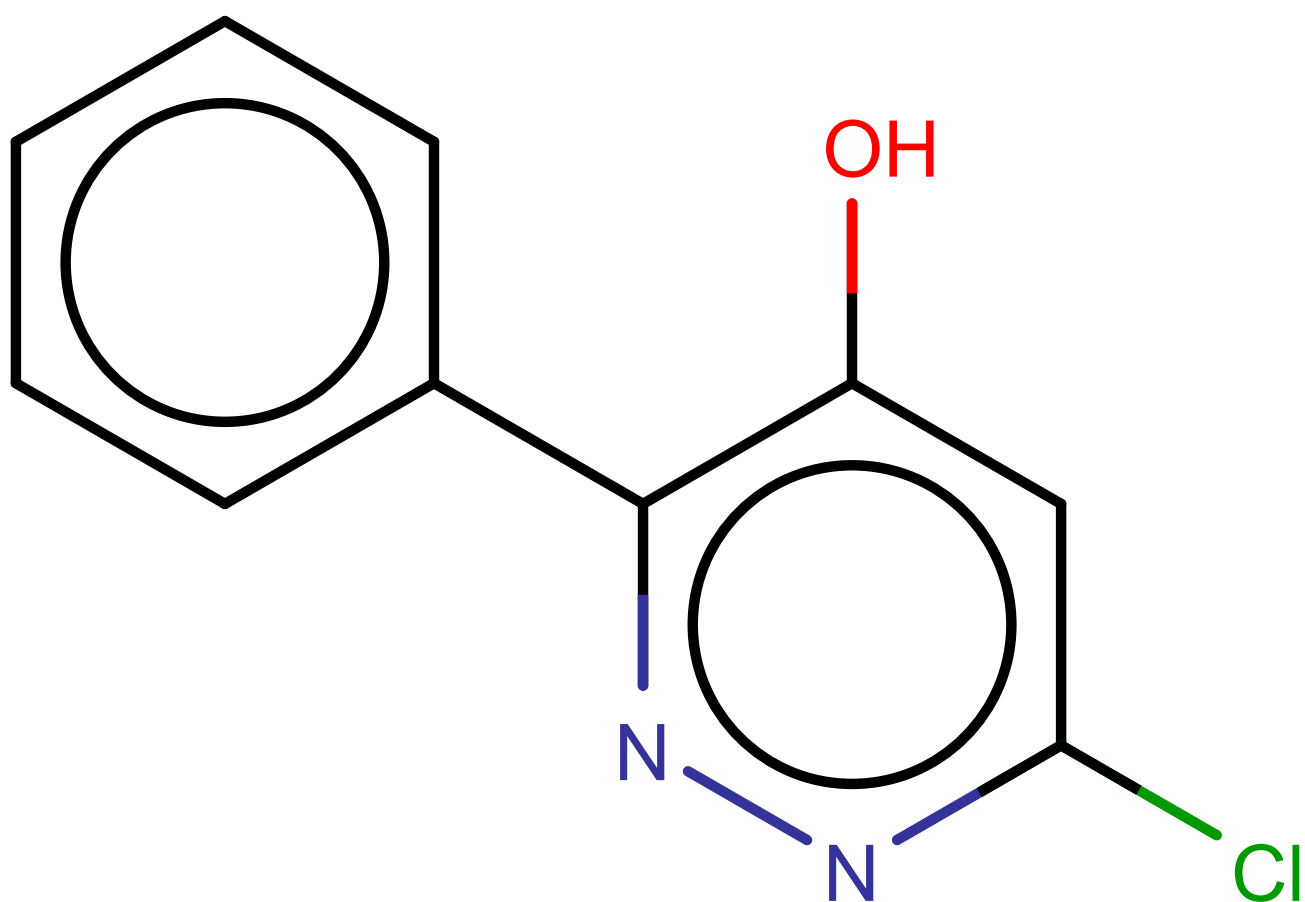

Supplement: Supplementary file 1 [file toxics-12-00425-s001.zip › Supplementary Materials/2D chemical structures/5358.pdf]

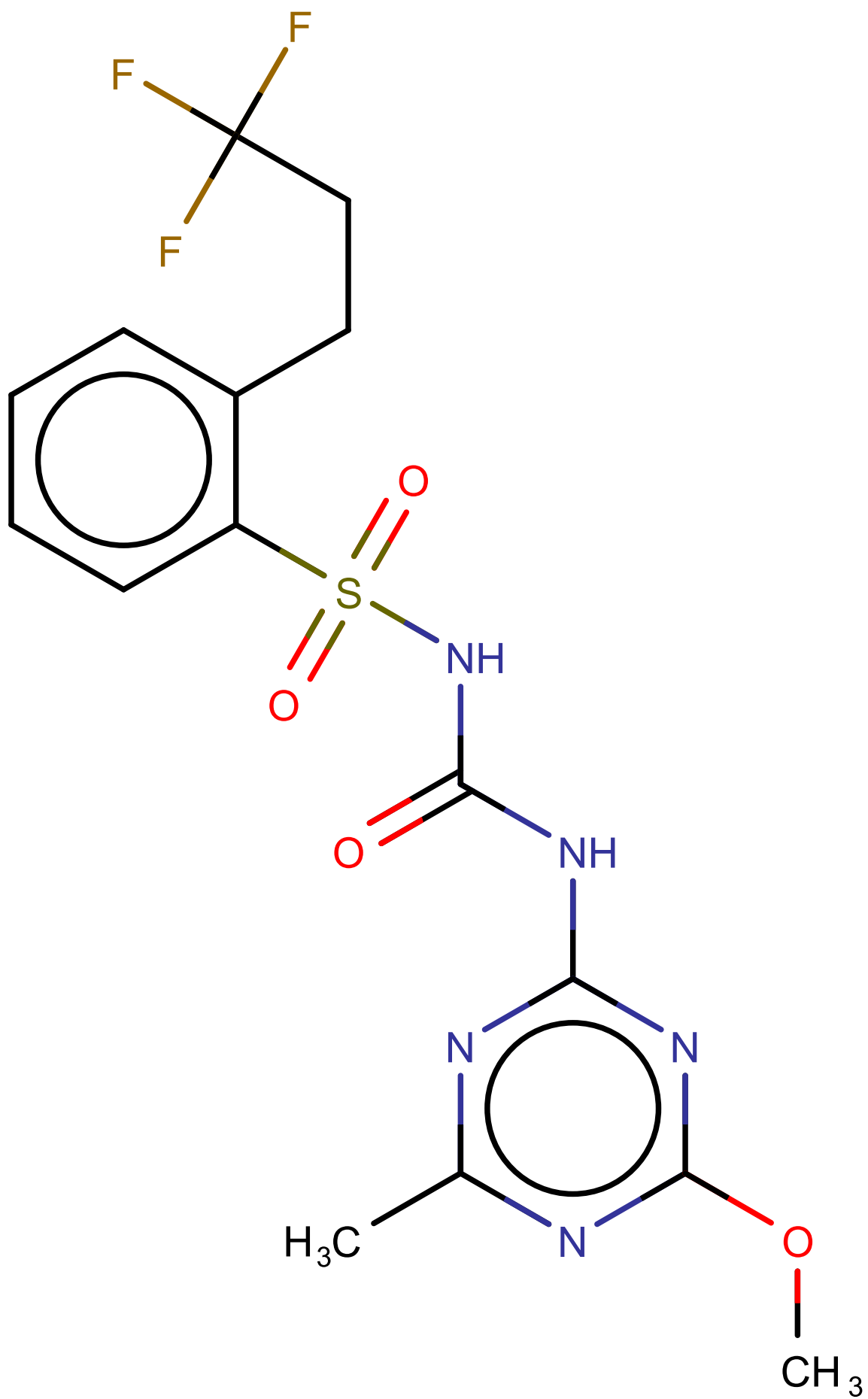

Supplement: Supplementary file 1 [file toxics-12-00425-s001.zip › Supplementary Materials/2D chemical structures/5391.pdf]

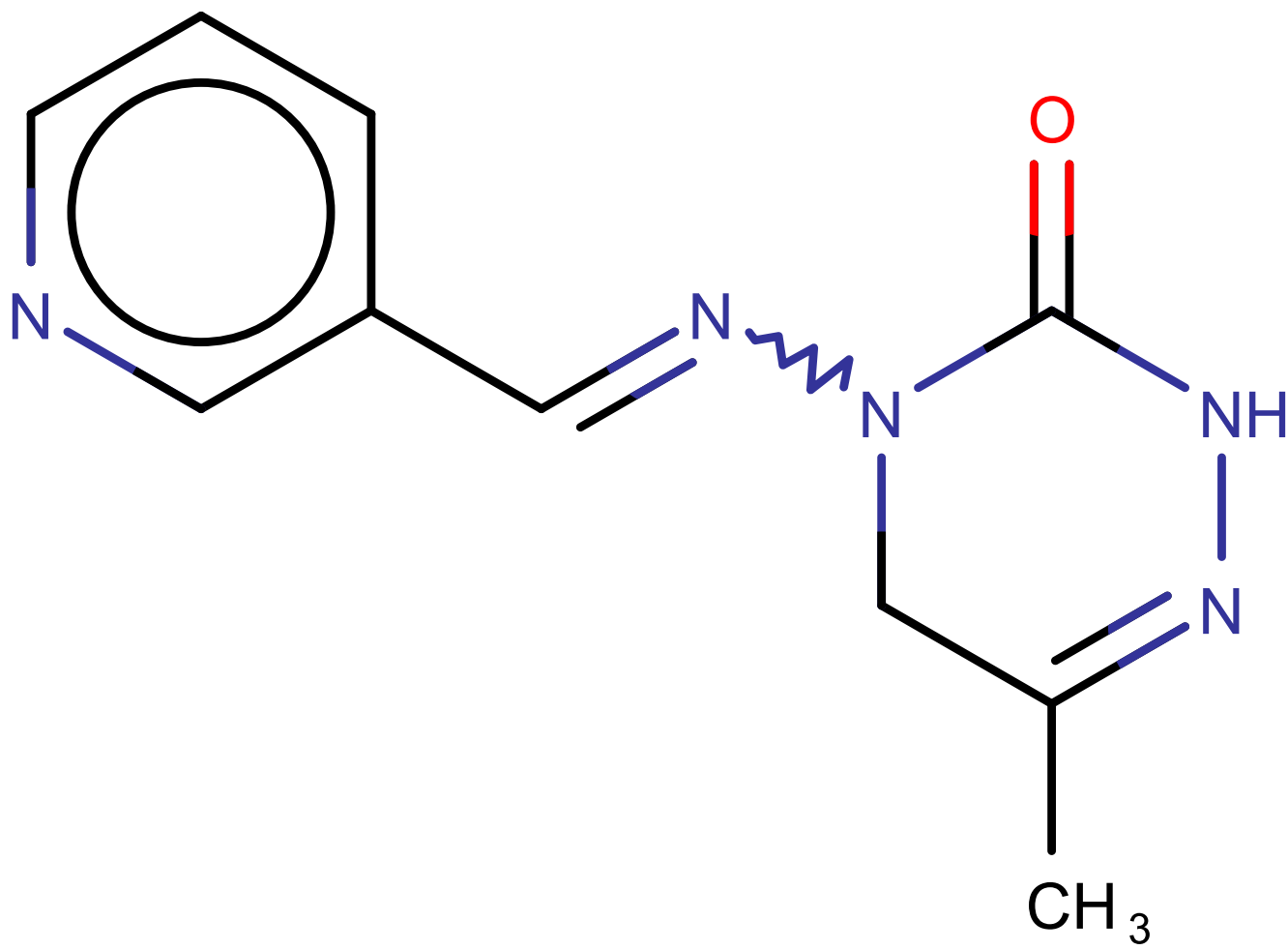

Supplement: Supplementary file 1 [file toxics-12-00425-s001.zip › Supplementary Materials/2D chemical structures/5393.pdf]

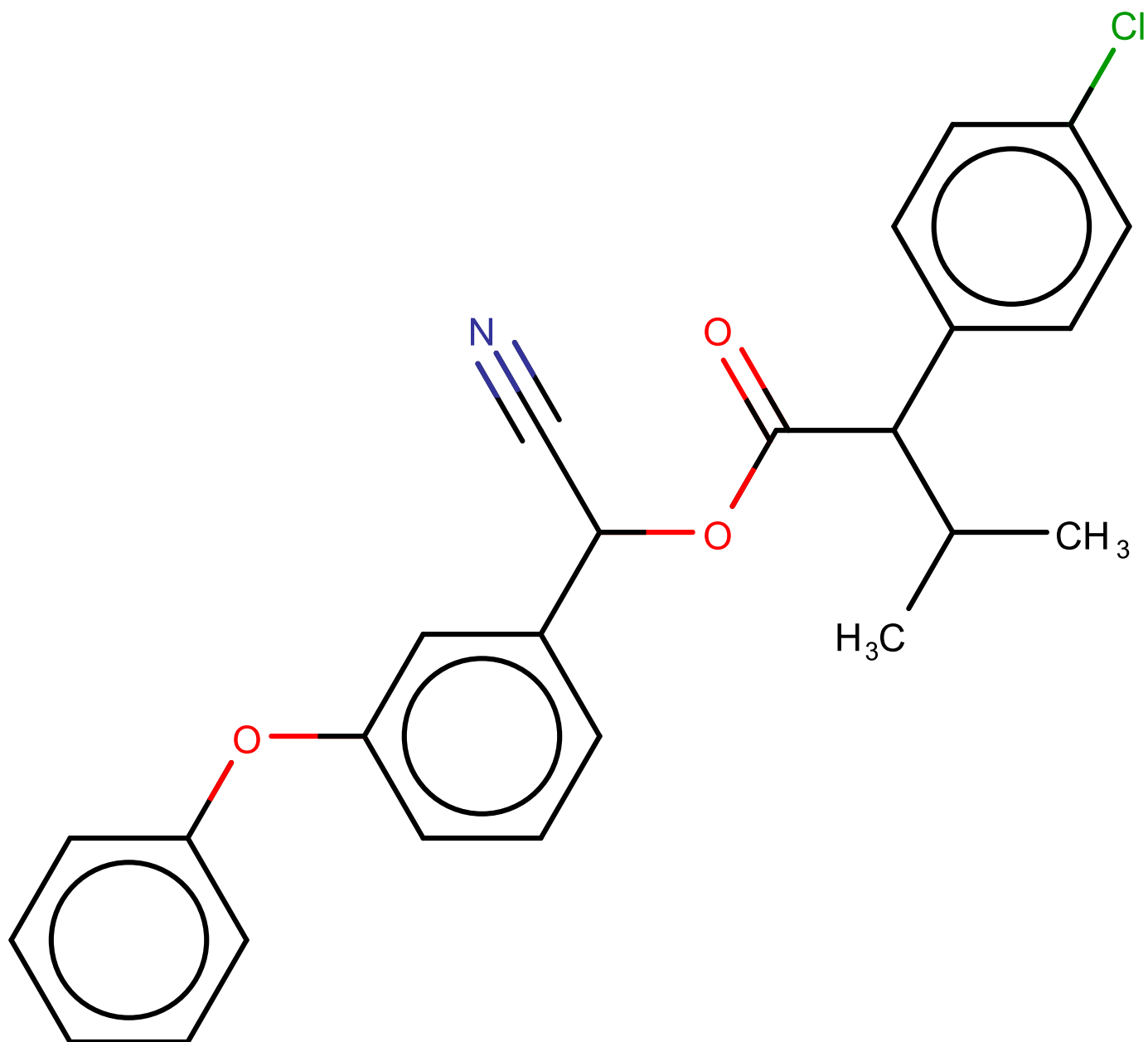

Supplement: Supplementary file 1 [file toxics-12-00425-s001.zip › Supplementary Materials/2D chemical structures/5432.pdf]

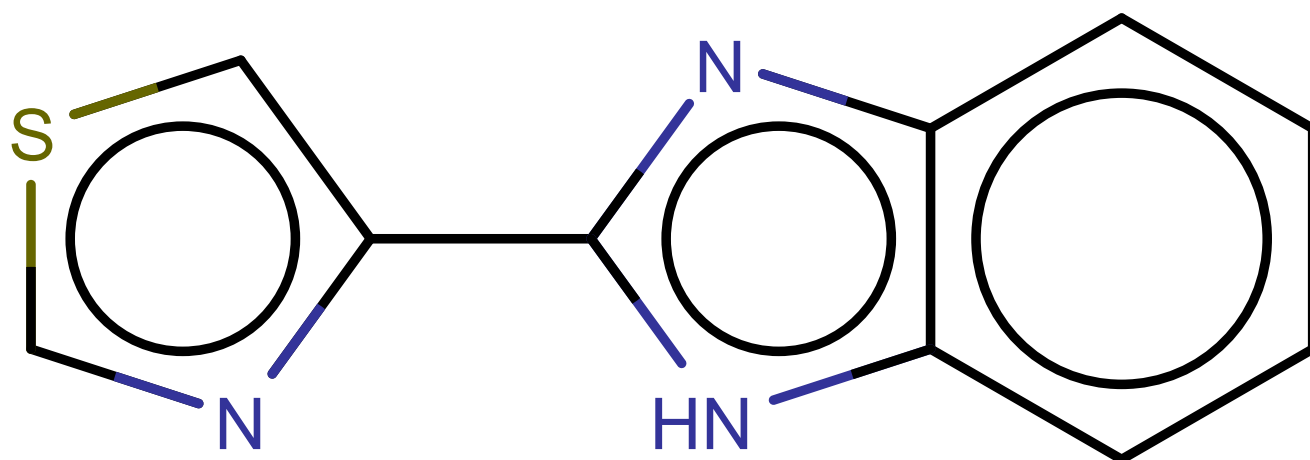

Supplement: Supplementary file 1 [file toxics-12-00425-s001.zip › Supplementary Materials/2D chemical structures/5439.pdf]

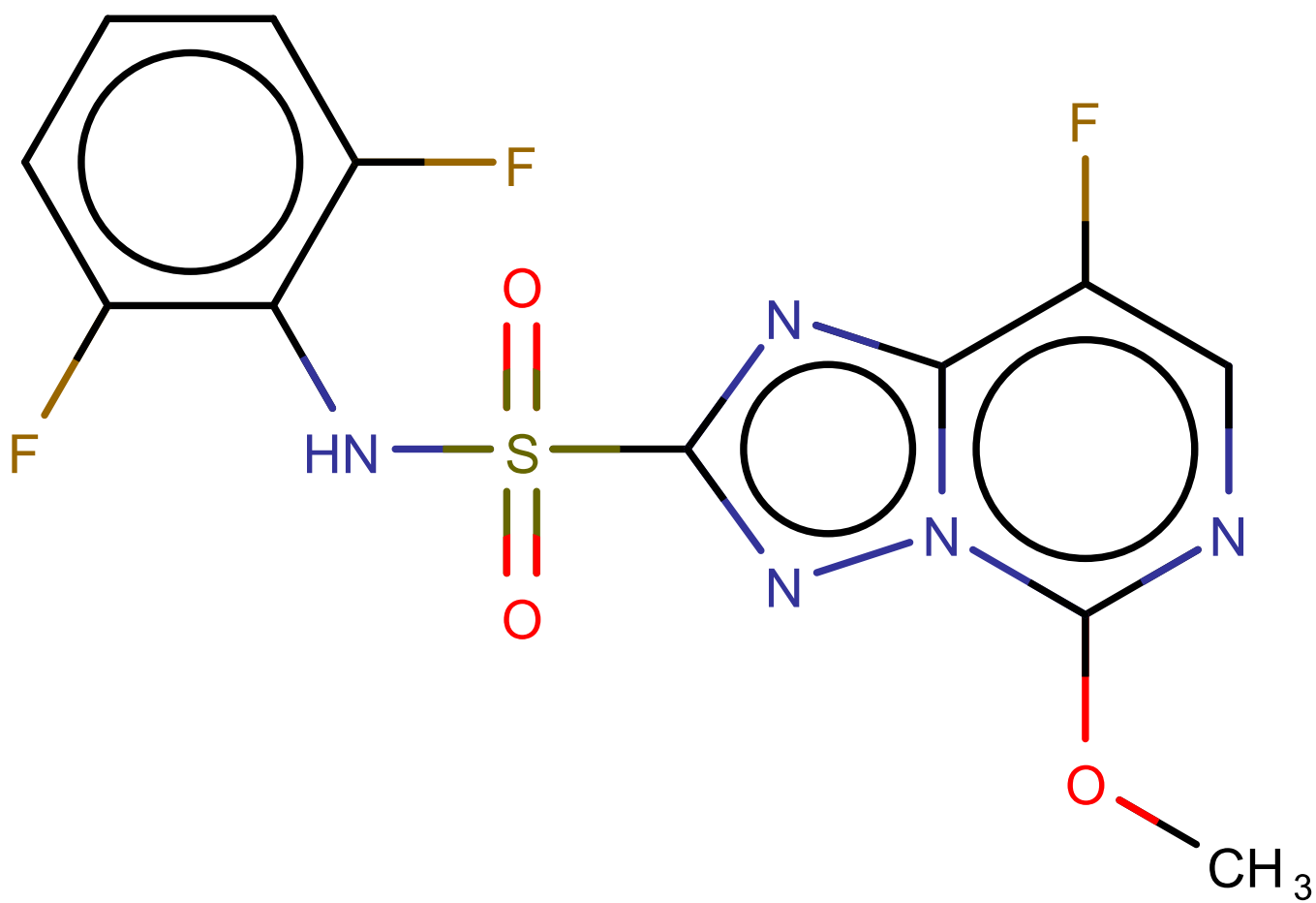

Supplement: Supplementary file 1 [file toxics-12-00425-s001.zip › Supplementary Materials/2D chemical structures/5474.pdf]

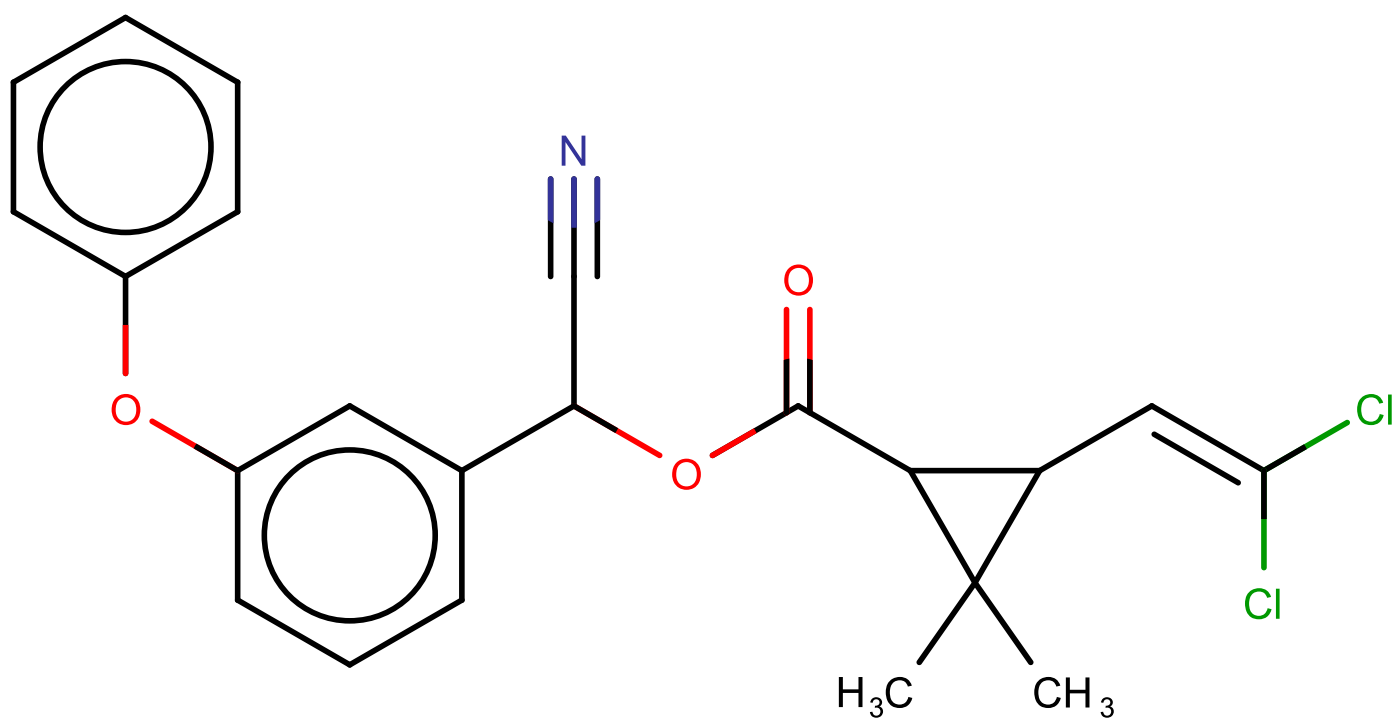

Supplement: Supplementary file 1 [file toxics-12-00425-s001.zip › Supplementary Materials/2D chemical structures/5493.pdf]

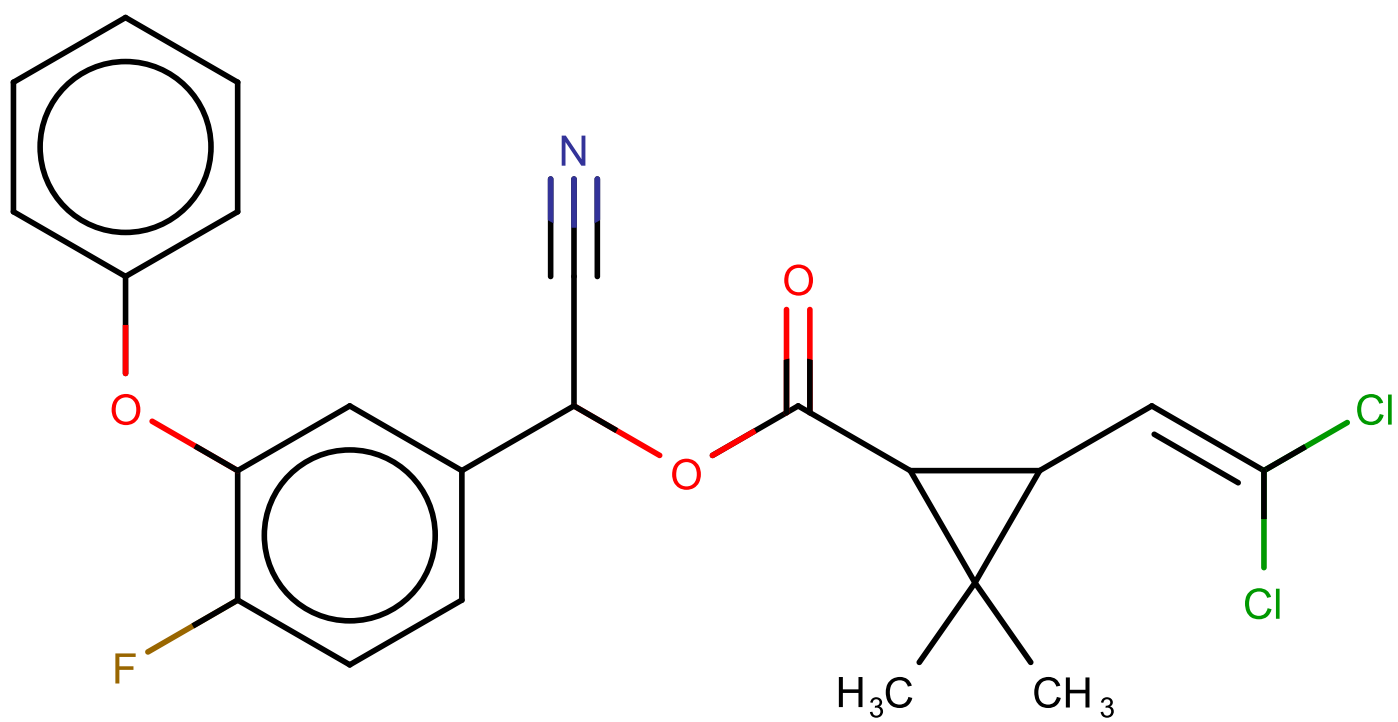

Supplement: Supplementary file 1 [file toxics-12-00425-s001.zip › Supplementary Materials/2D chemical structures/5494.pdf]

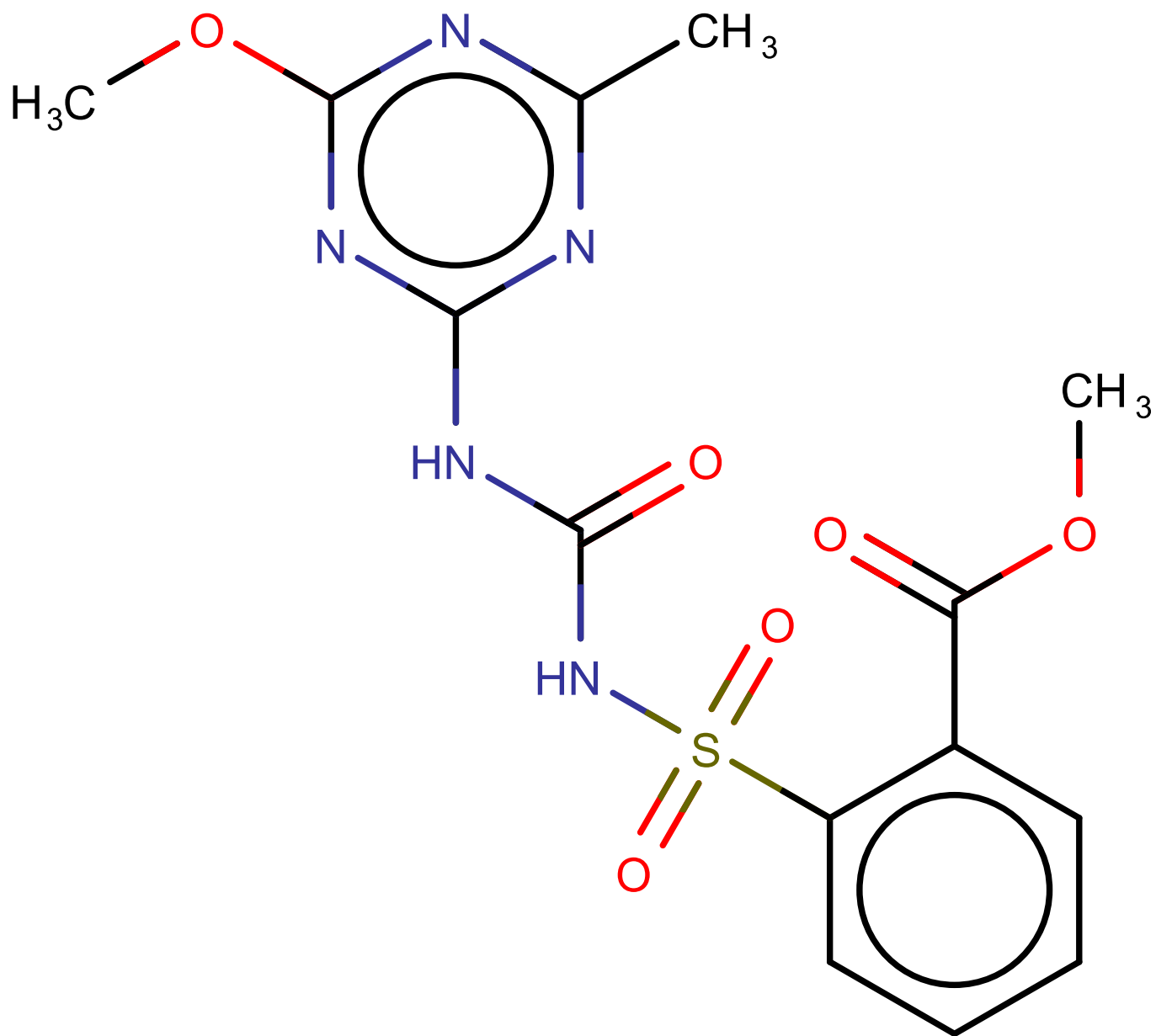

Supplement: Supplementary file 1 [file toxics-12-00425-s001.zip › Supplementary Materials/2D chemical structures/5496.pdf]

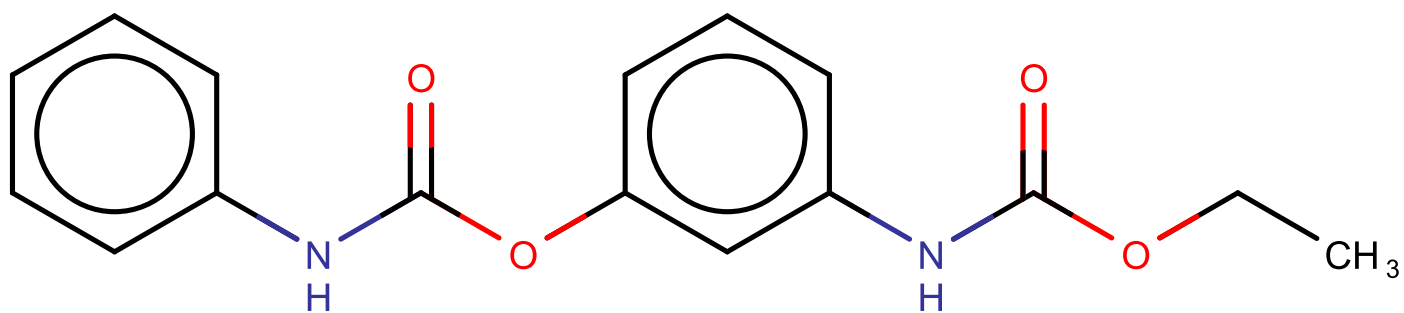

Supplement: Supplementary file 1 [file toxics-12-00425-s001.zip › Supplementary Materials/2D chemical structures/5501.pdf]

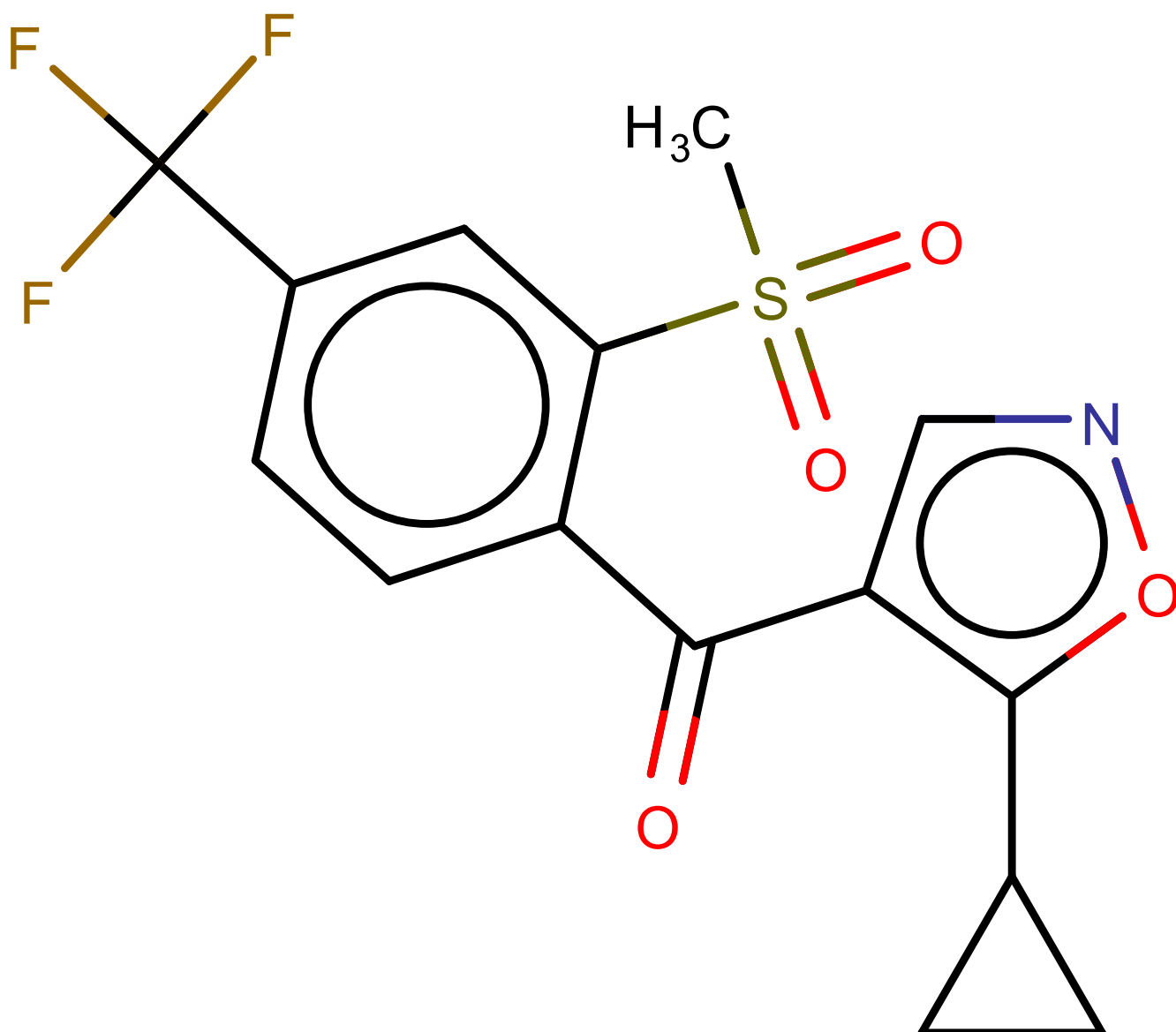

Supplement: Supplementary file 1 [file toxics-12-00425-s001.zip › Supplementary Materials/2D chemical structures/5503.pdf]

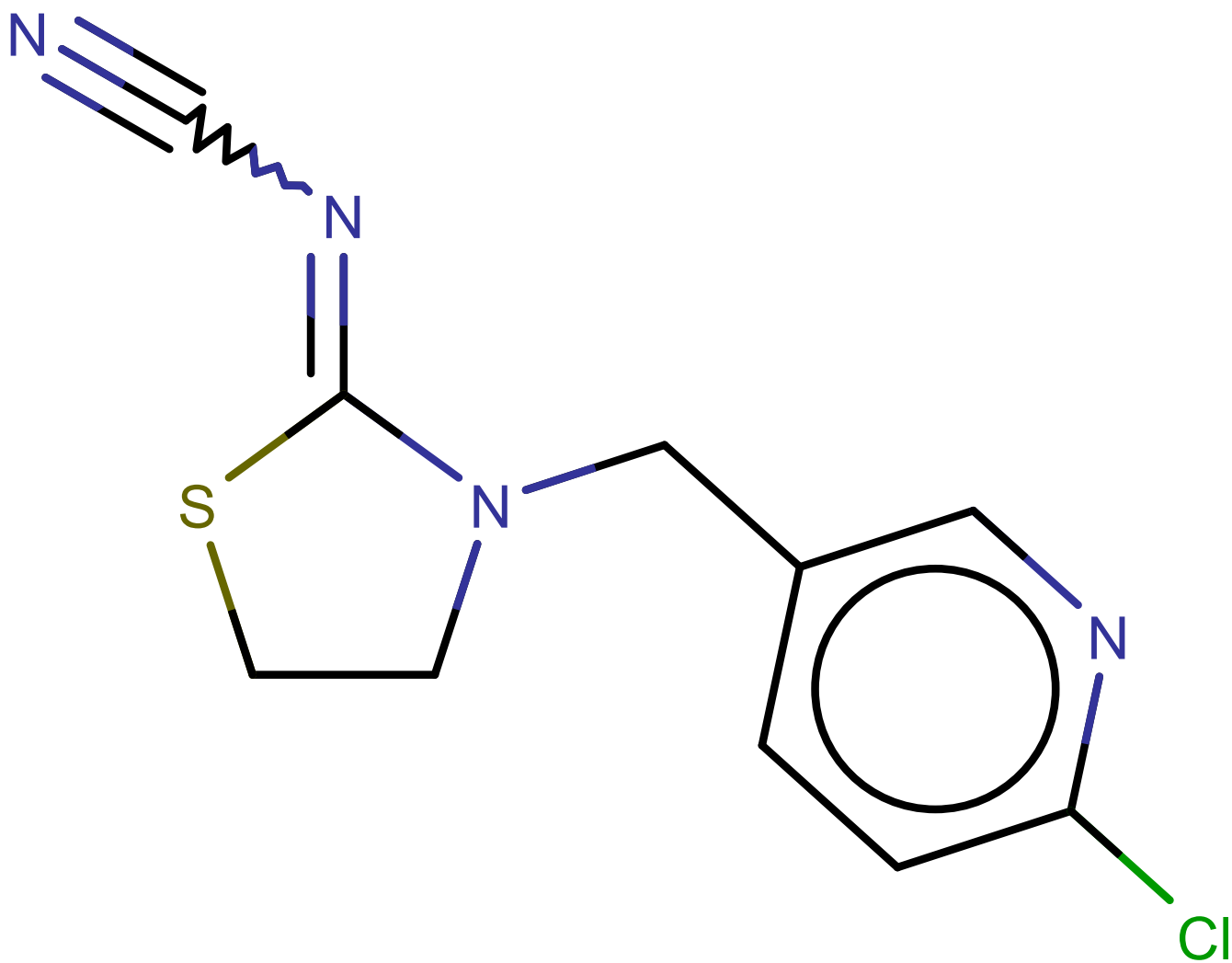

Supplement: Supplementary file 1 [file toxics-12-00425-s001.zip › Supplementary Materials/2D chemical structures/5509.pdf]

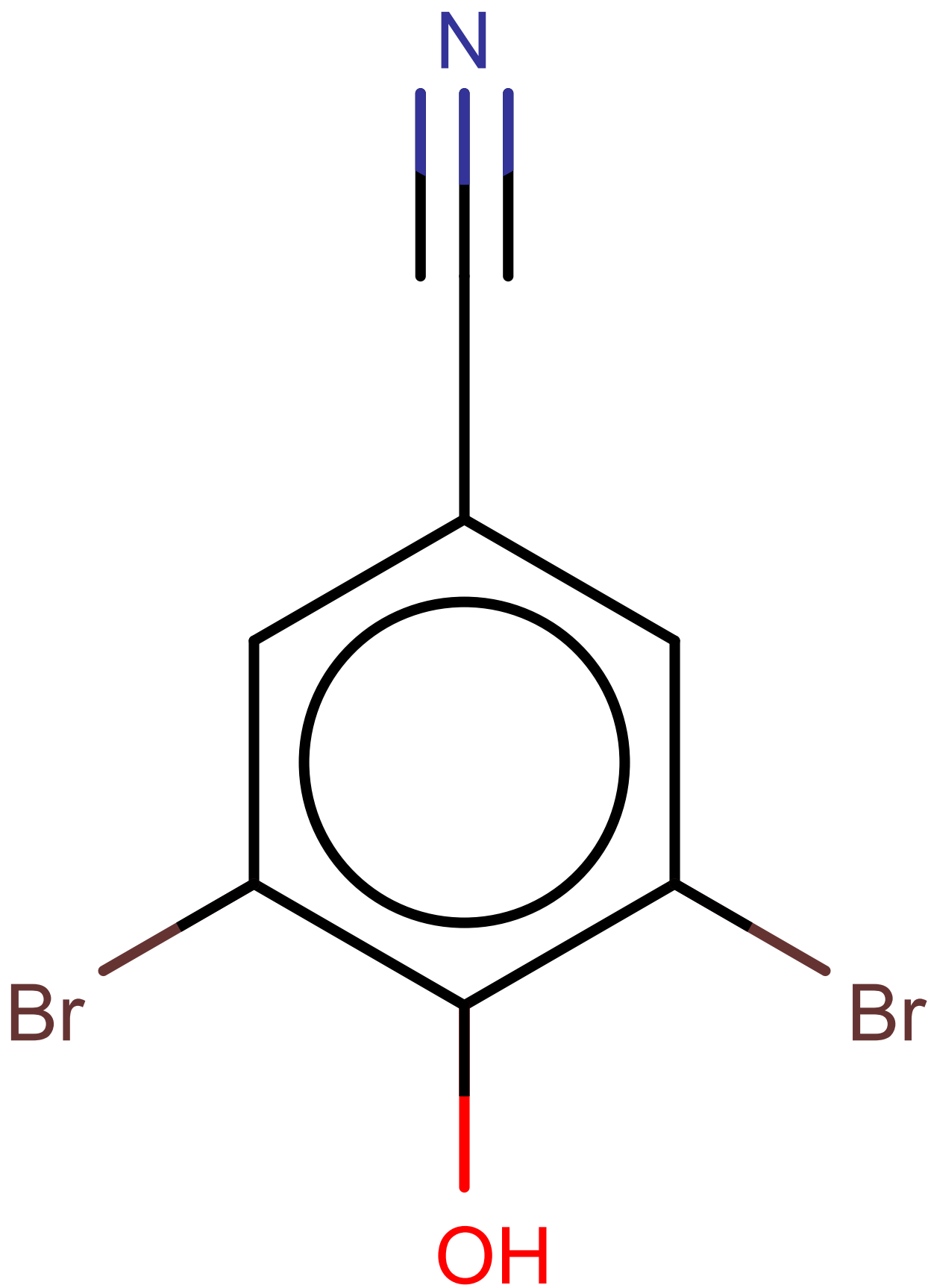

Supplement: Supplementary file 1 [file toxics-12-00425-s001.zip › Supplementary Materials/2D chemical structures/5515.pdf]

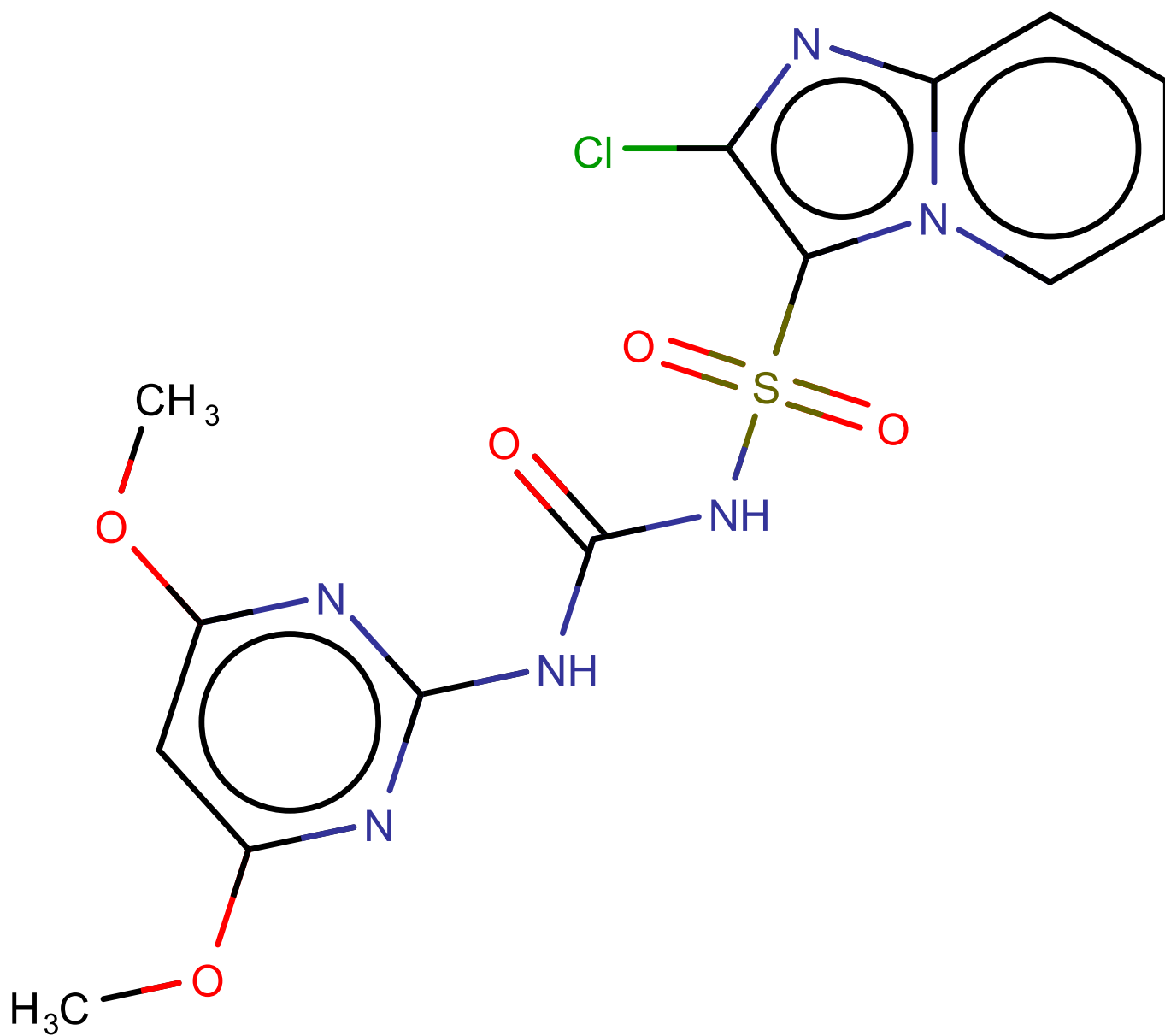

Supplement: Supplementary file 1 [file toxics-12-00425-s001.zip › Supplementary Materials/2D chemical structures/5518.pdf]

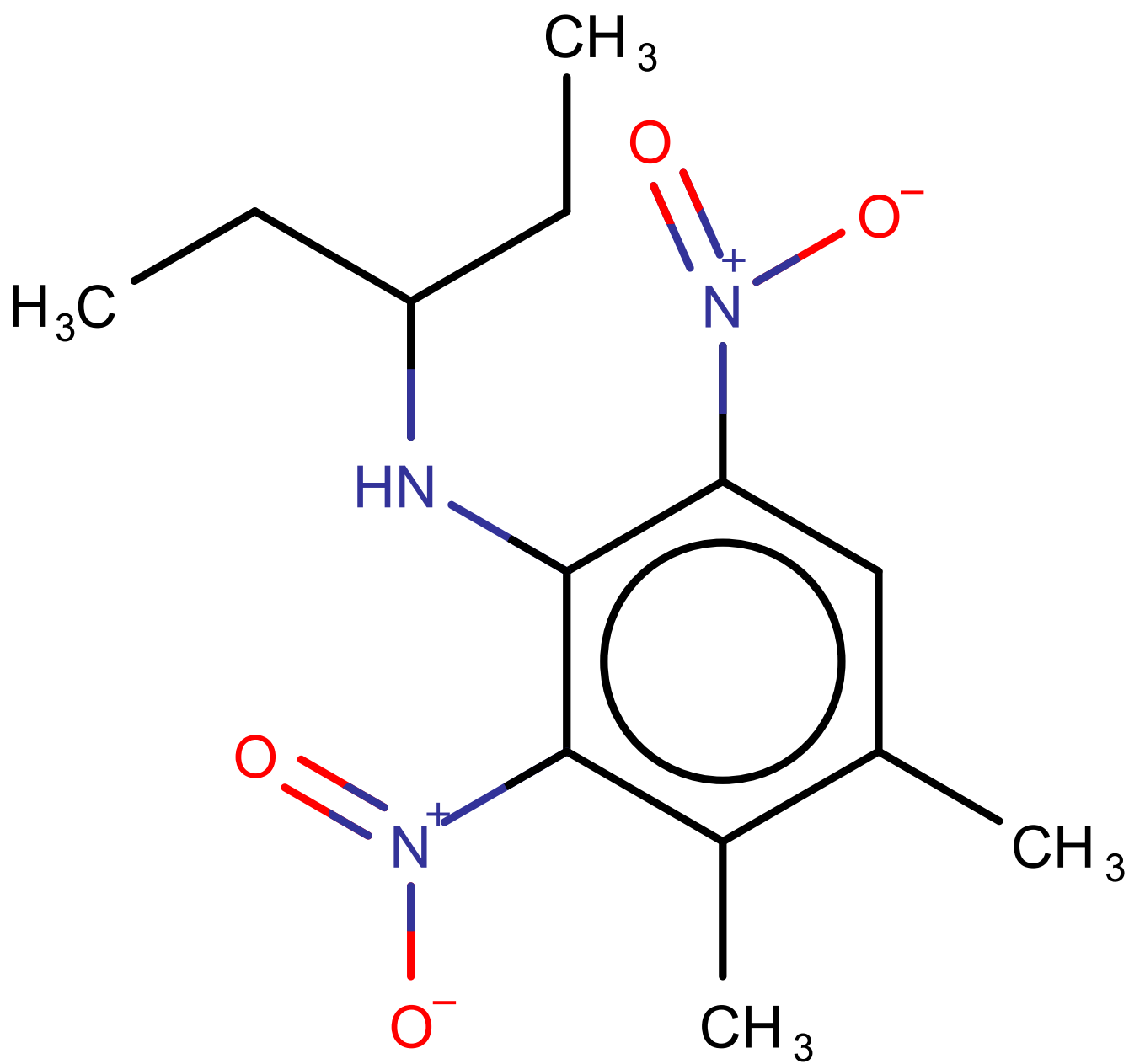

Supplement: Supplementary file 1 [file toxics-12-00425-s001.zip › Supplementary Materials/2D chemical structures/5522.pdf]

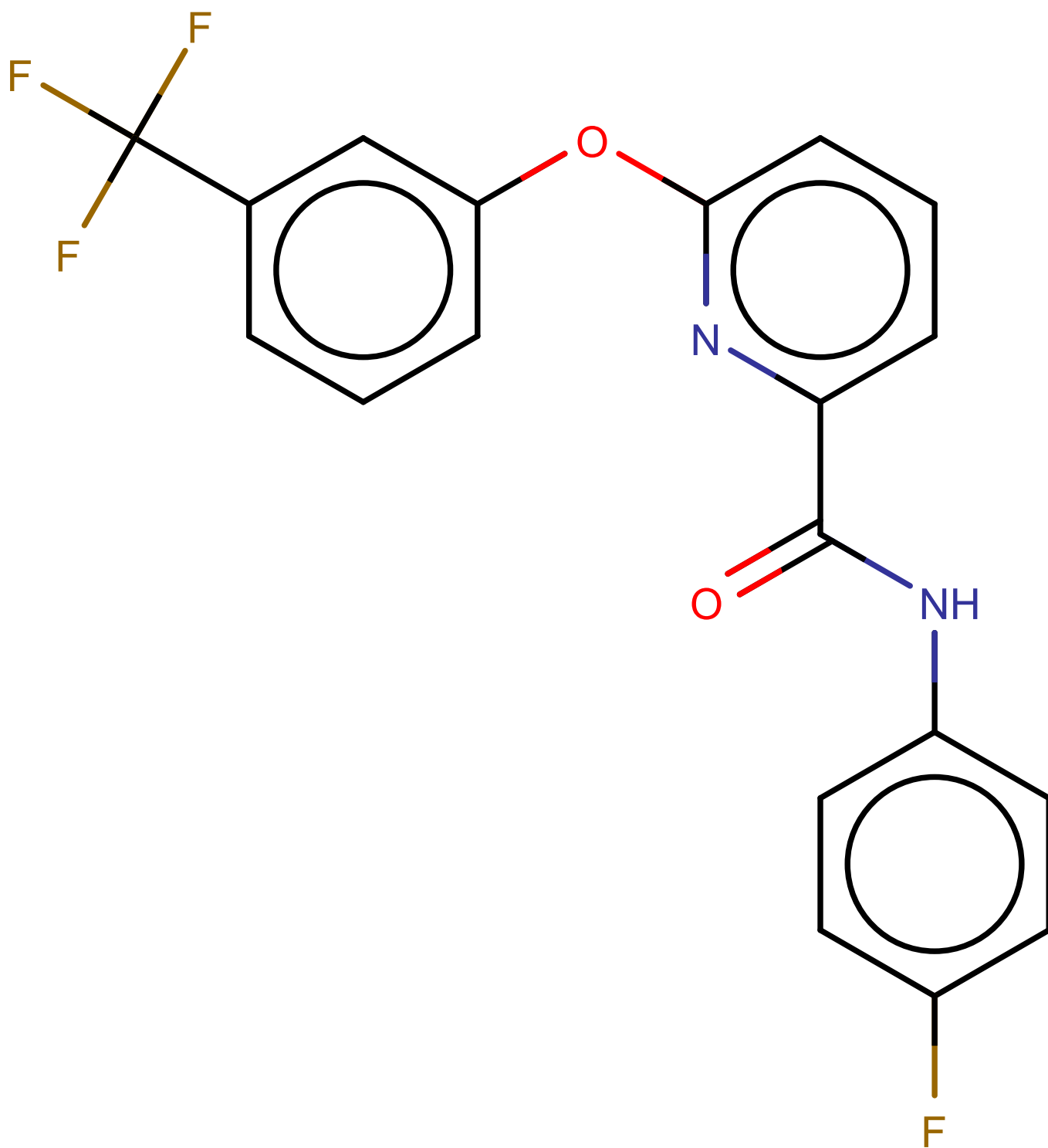

Supplement: Supplementary file 1 [file toxics-12-00425-s001.zip › Supplementary Materials/2D chemical structures/5524.pdf]

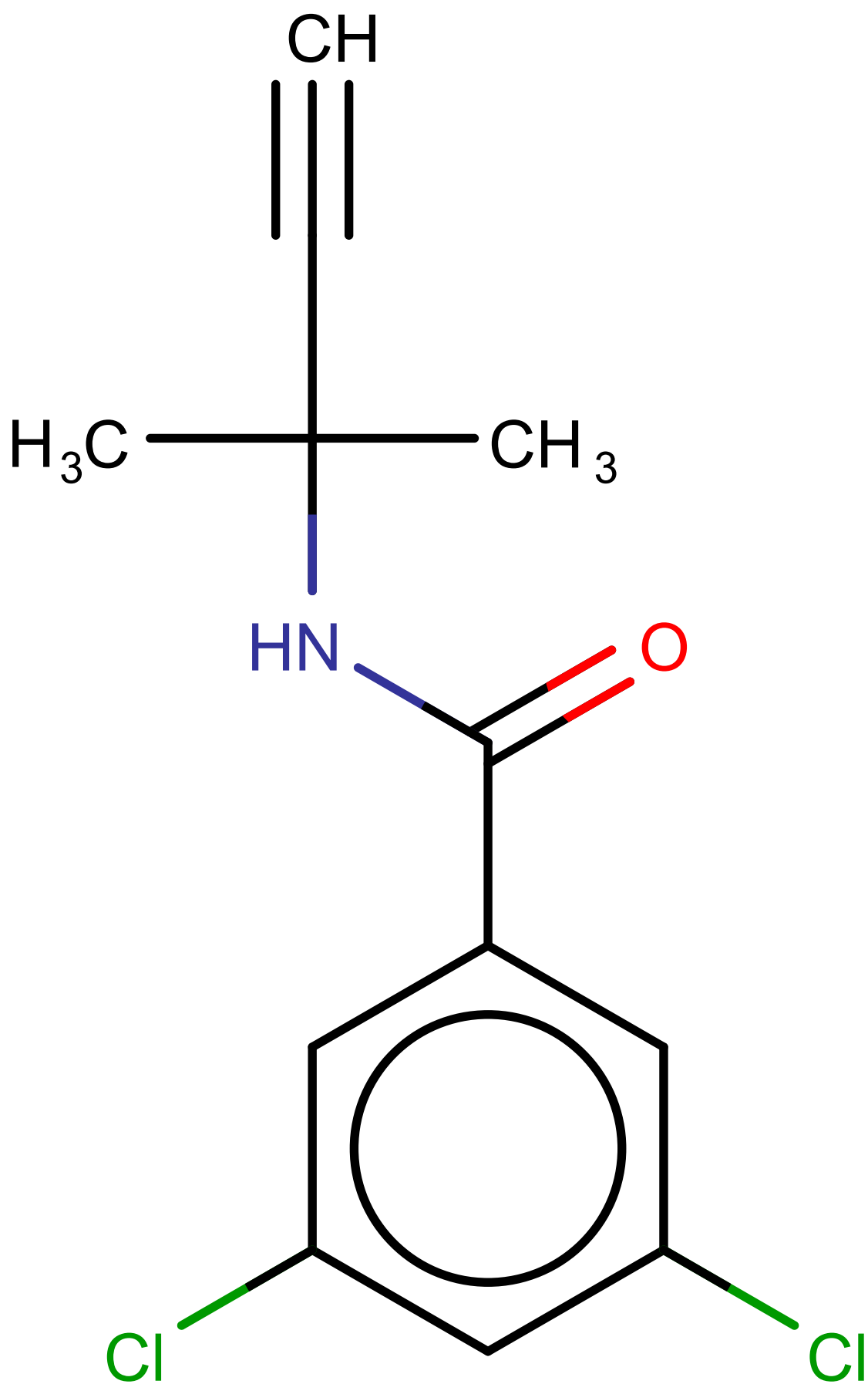

Supplement: Supplementary file 1 [file toxics-12-00425-s001.zip › Supplementary Materials/2D chemical structures/5525.pdf]

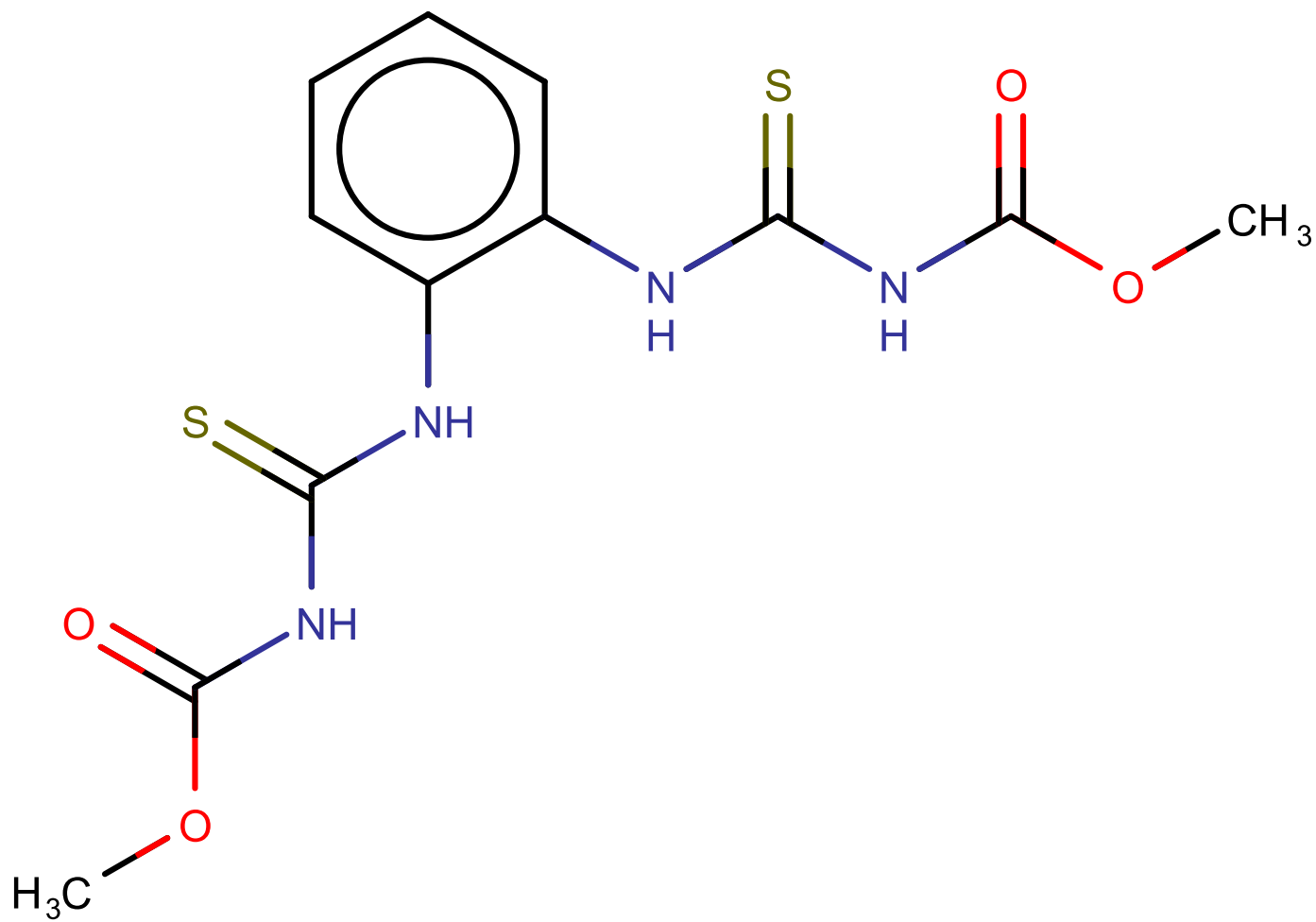

Supplement: Supplementary file 1 [file toxics-12-00425-s001.zip › Supplementary Materials/2D chemical structures/5528.pdf]

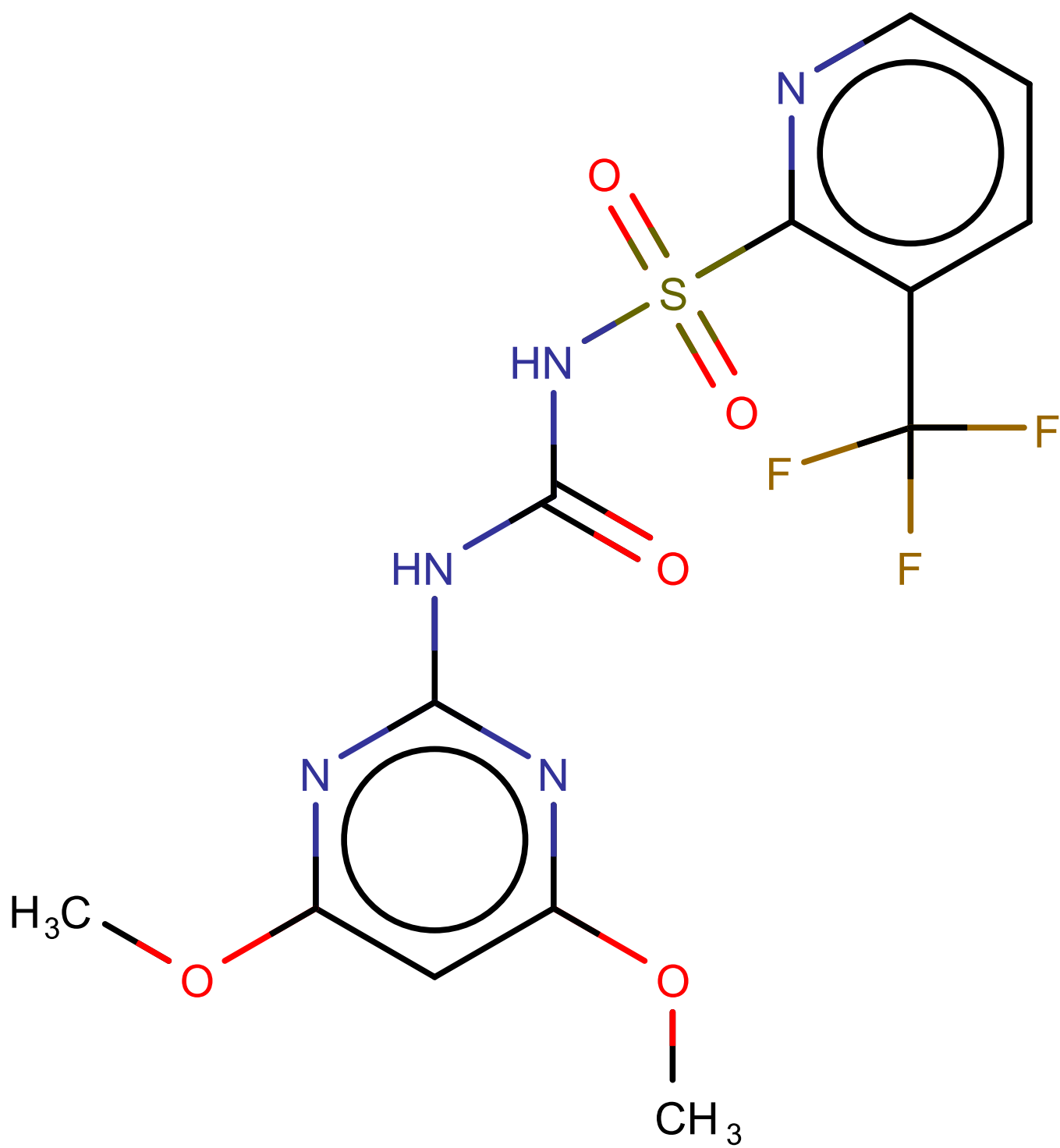

Supplement: Supplementary file 1 [file toxics-12-00425-s001.zip › Supplementary Materials/2D chemical structures/5531.pdf]

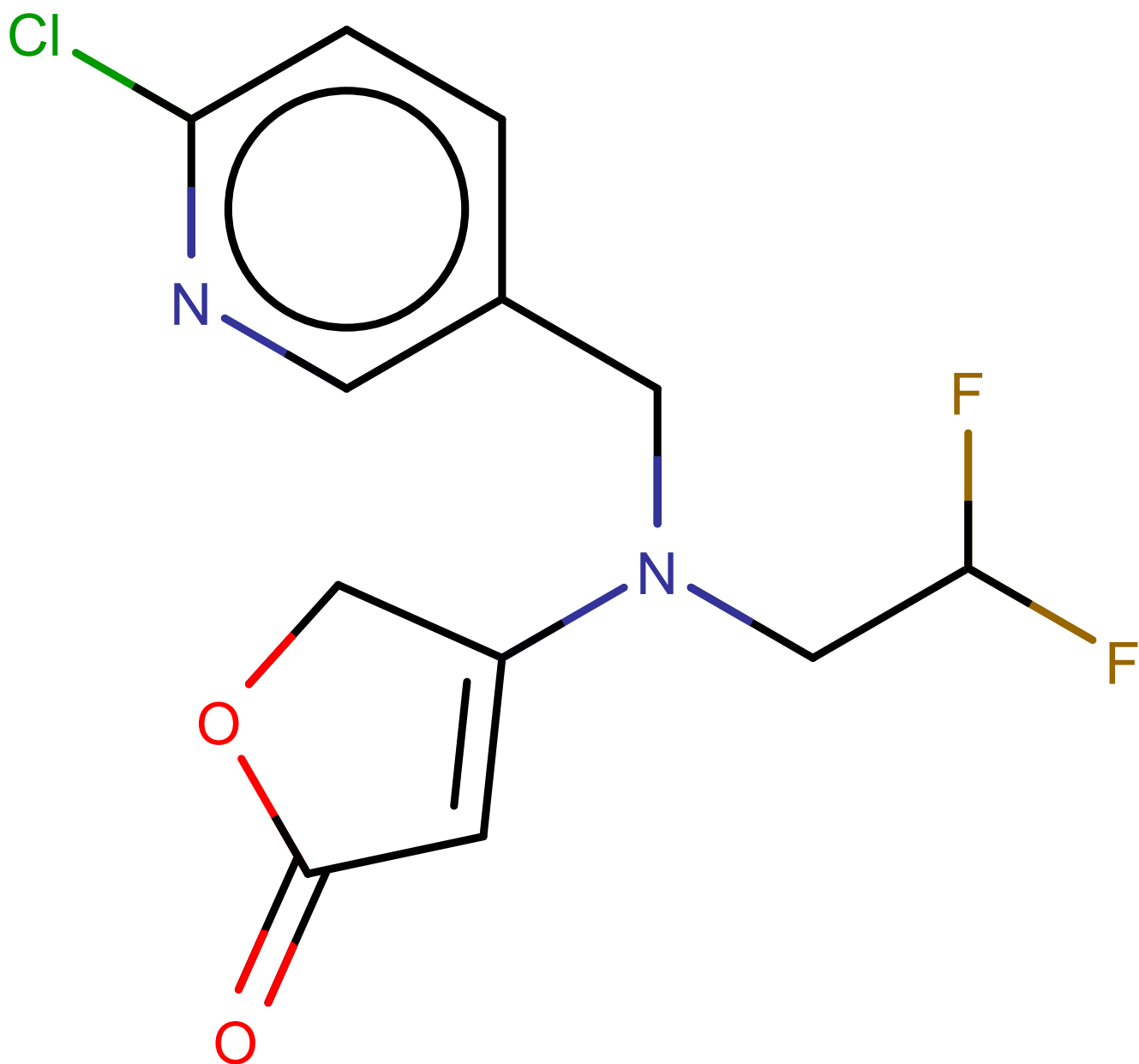

Supplement: Supplementary file 1 [file toxics-12-00425-s001.zip › Supplementary Materials/2D chemical structures/5536.pdf]

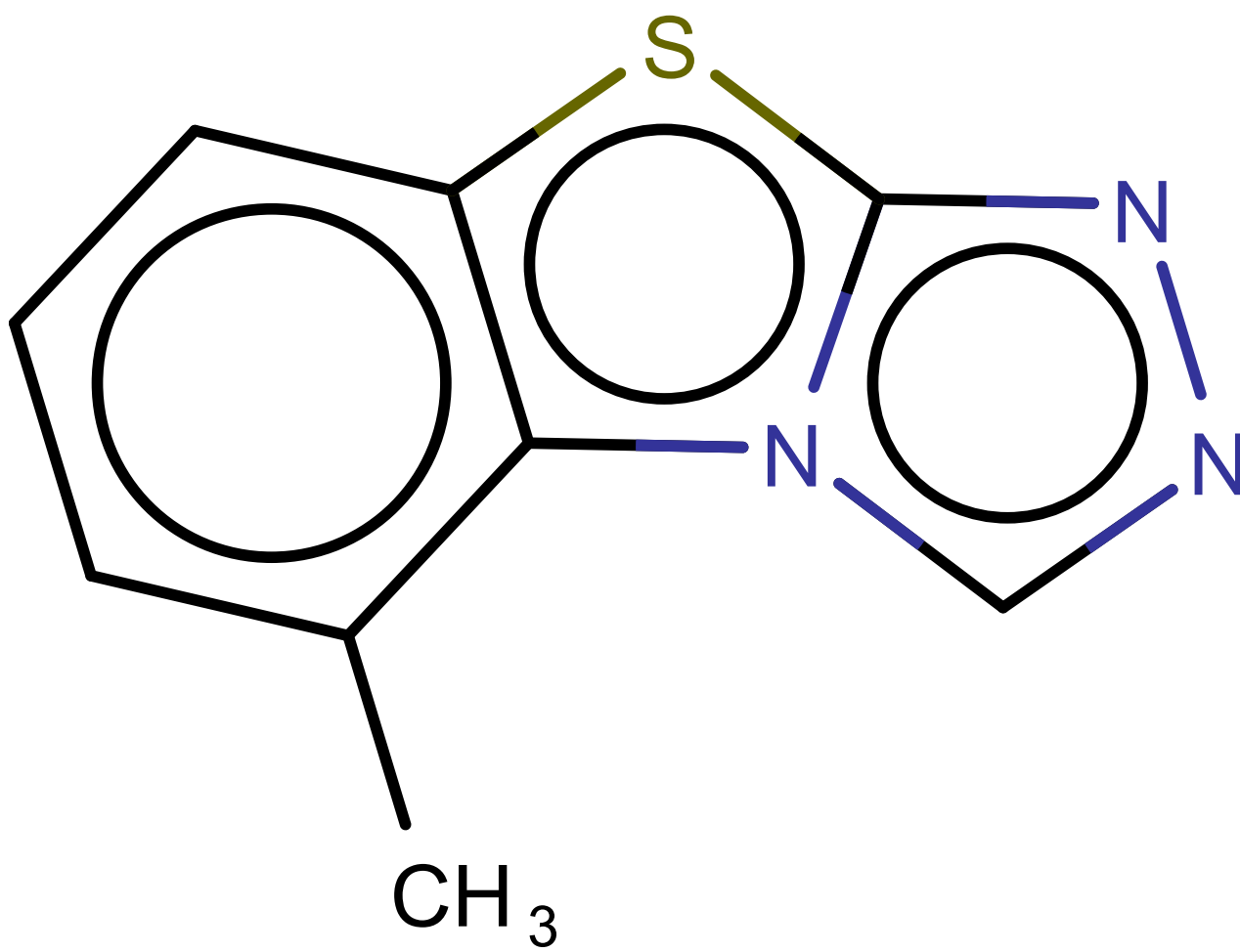

Supplement: Supplementary file 1 [file toxics-12-00425-s001.zip › Supplementary Materials/2D chemical structures/5551.pdf]

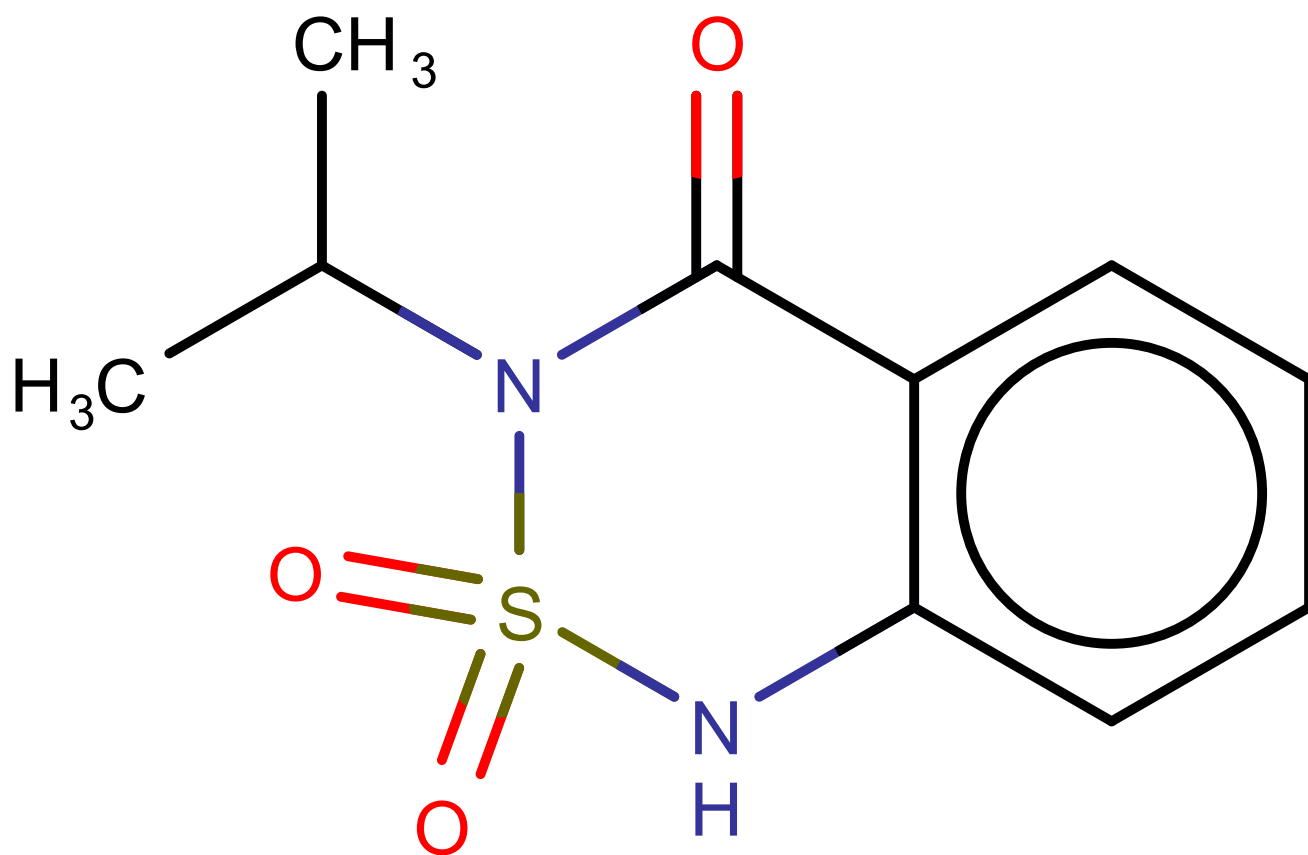

Supplement: Supplementary file 1 [file toxics-12-00425-s001.zip › Supplementary Materials/2D chemical structures/5568.pdf]

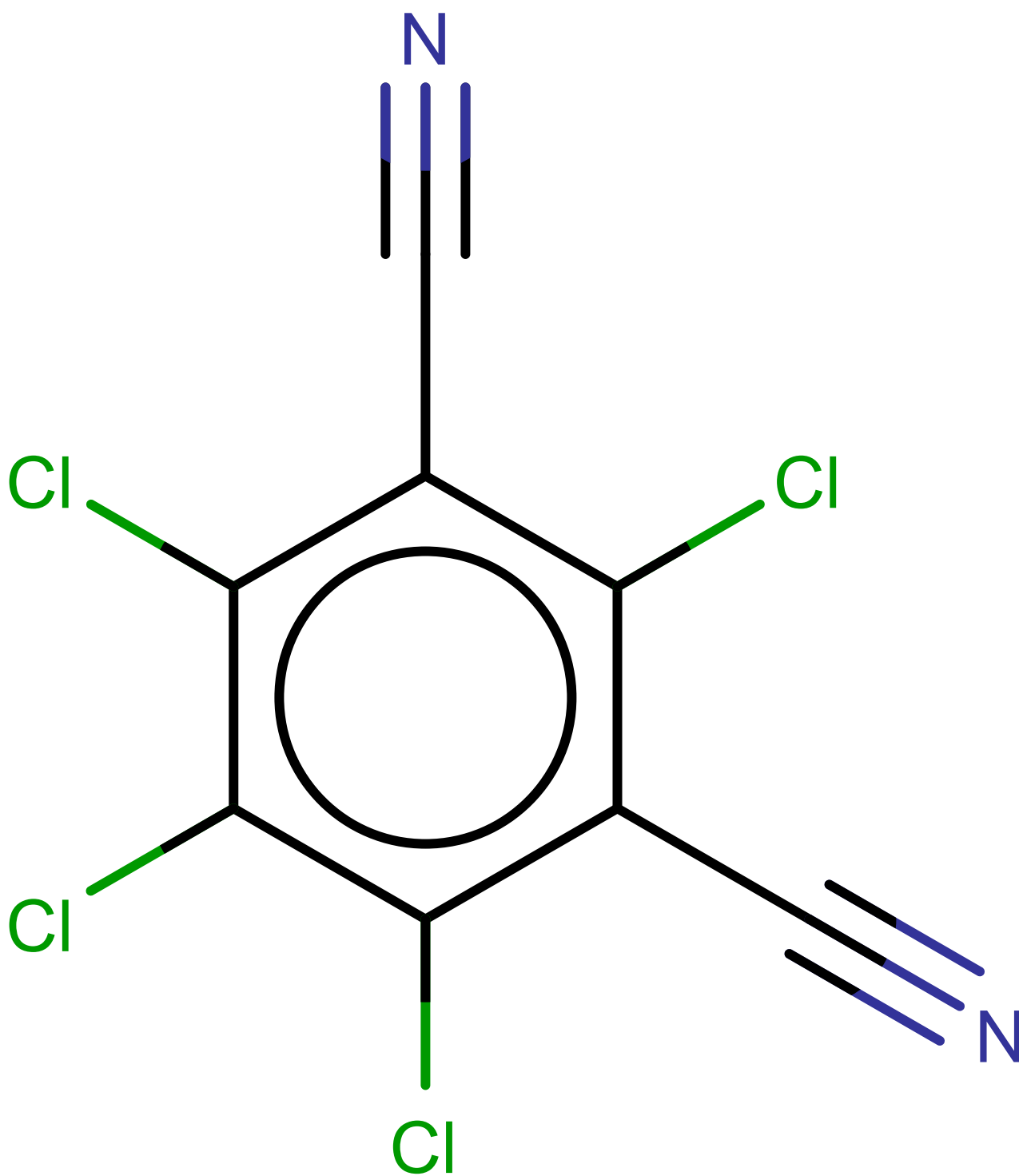

Supplement: Supplementary file 1 [file toxics-12-00425-s001.zip › Supplementary Materials/2D chemical structures/5604.pdf]

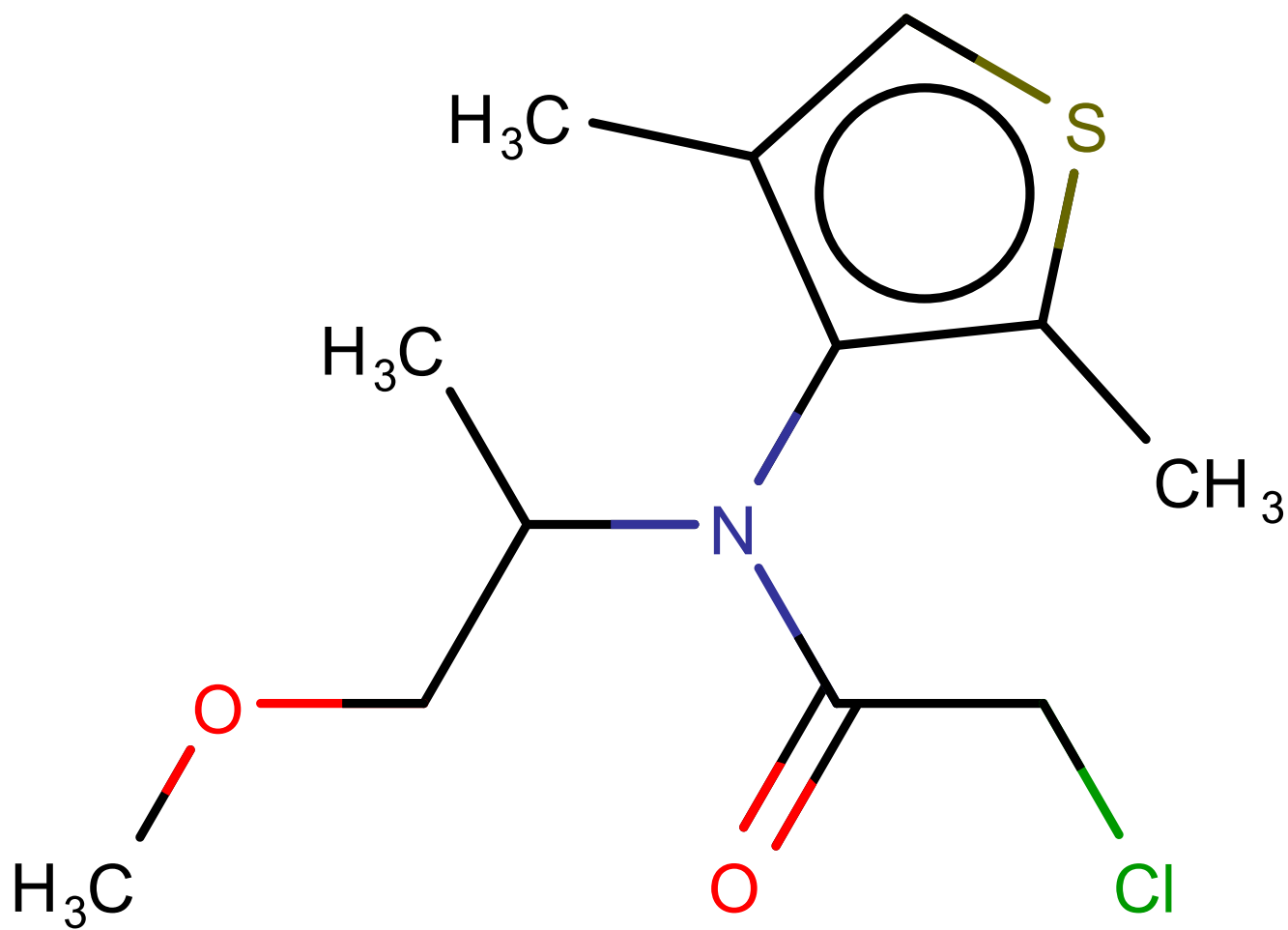

Supplement: Supplementary file 1 [file toxics-12-00425-s001.zip › Supplementary Materials/2D chemical structures/5620.pdf]

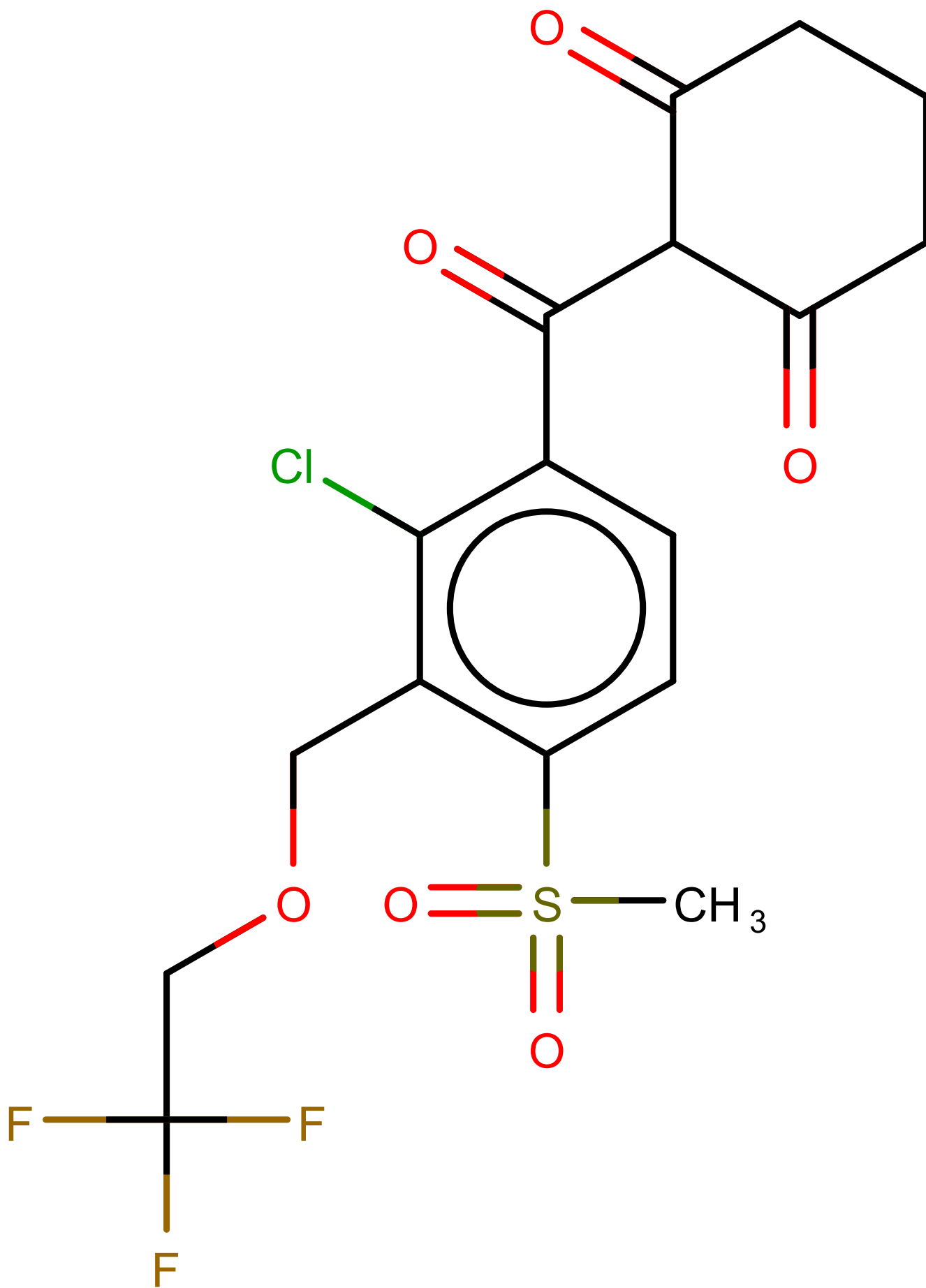

Supplement: Supplementary file 1 [file toxics-12-00425-s001.zip › Supplementary Materials/2D chemical structures/5621.pdf]

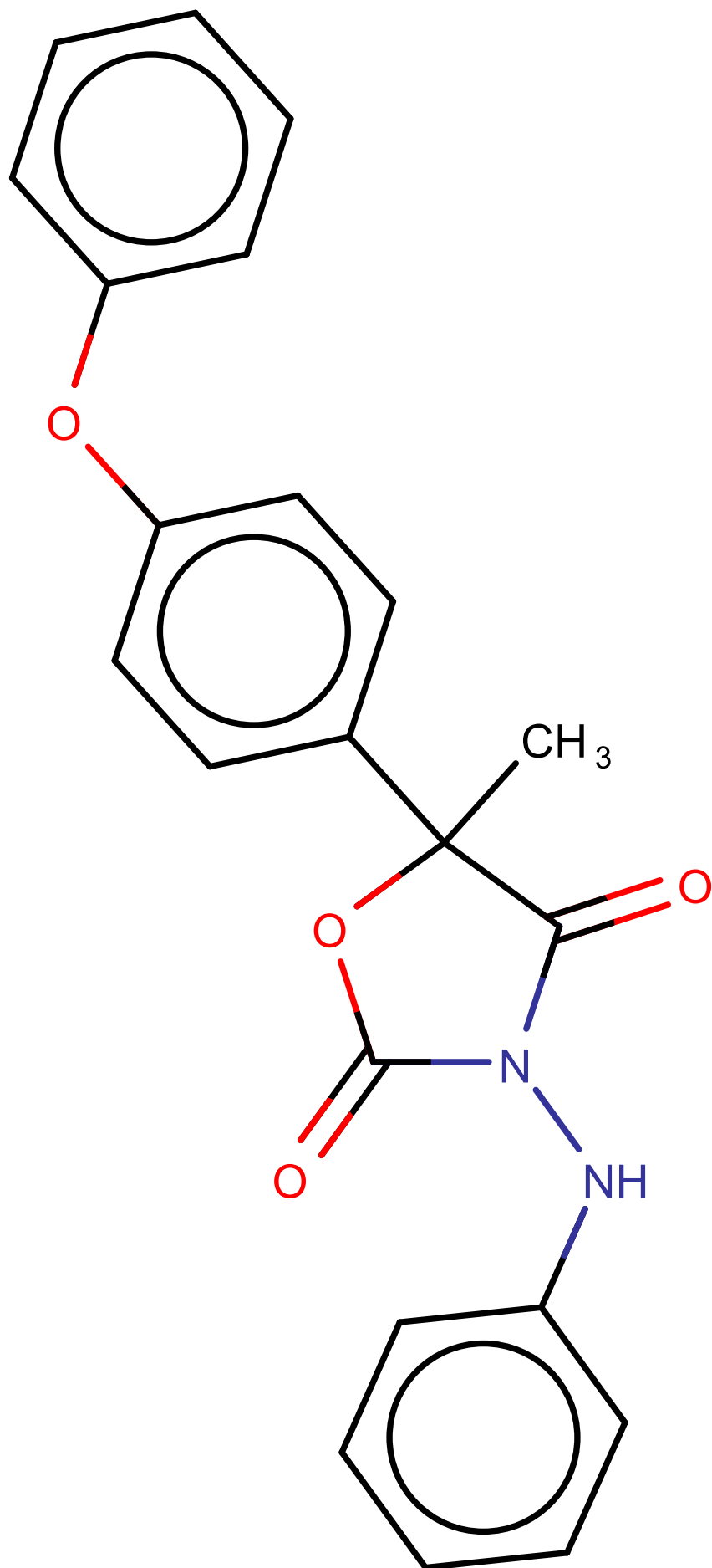

Supplement: Supplementary file 1 [file toxics-12-00425-s001.zip › Supplementary Materials/2D chemical structures/5628.pdf]

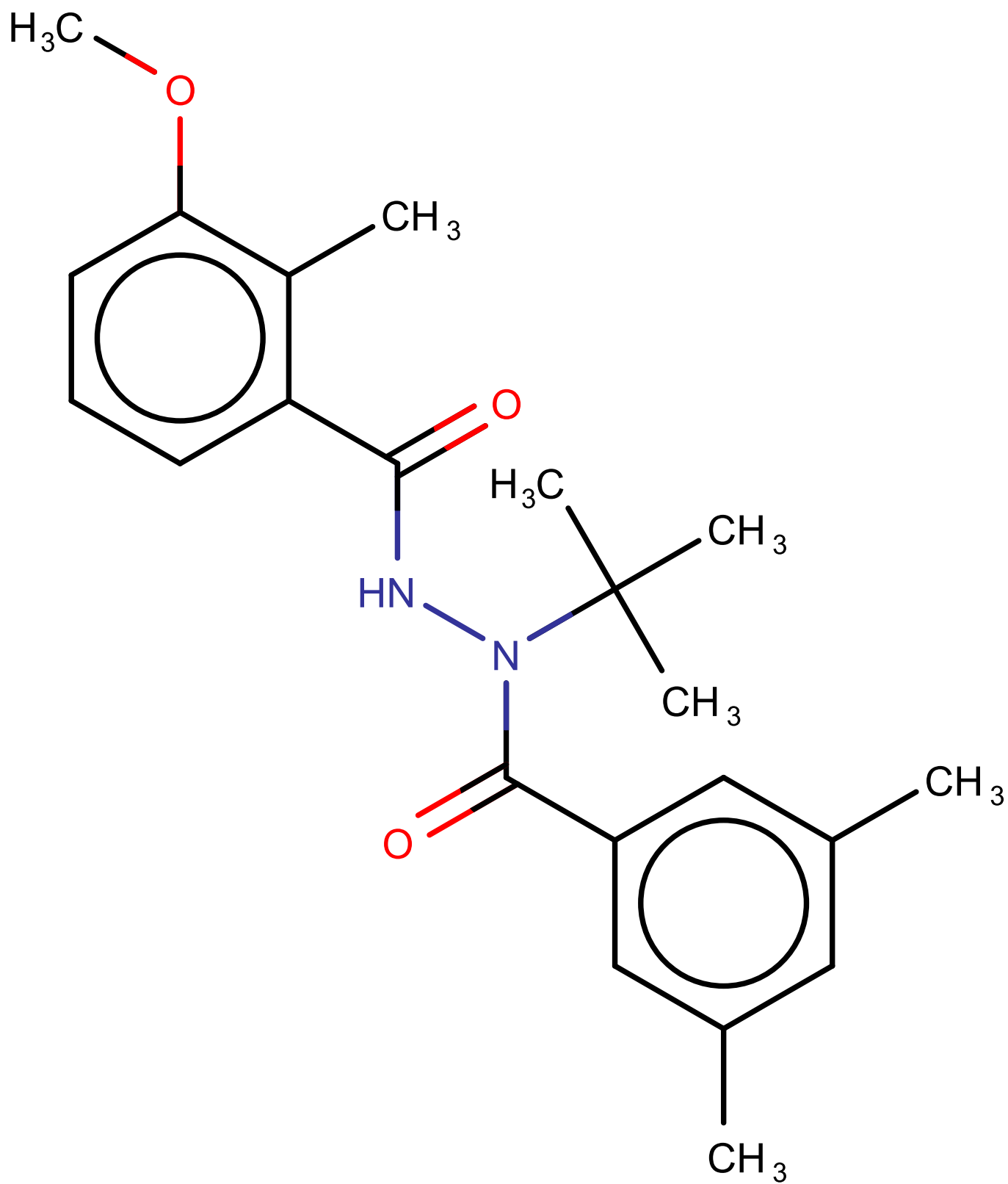

Supplement: Supplementary file 1 [file toxics-12-00425-s001.zip › Supplementary Materials/2D chemical structures/5636.pdf]

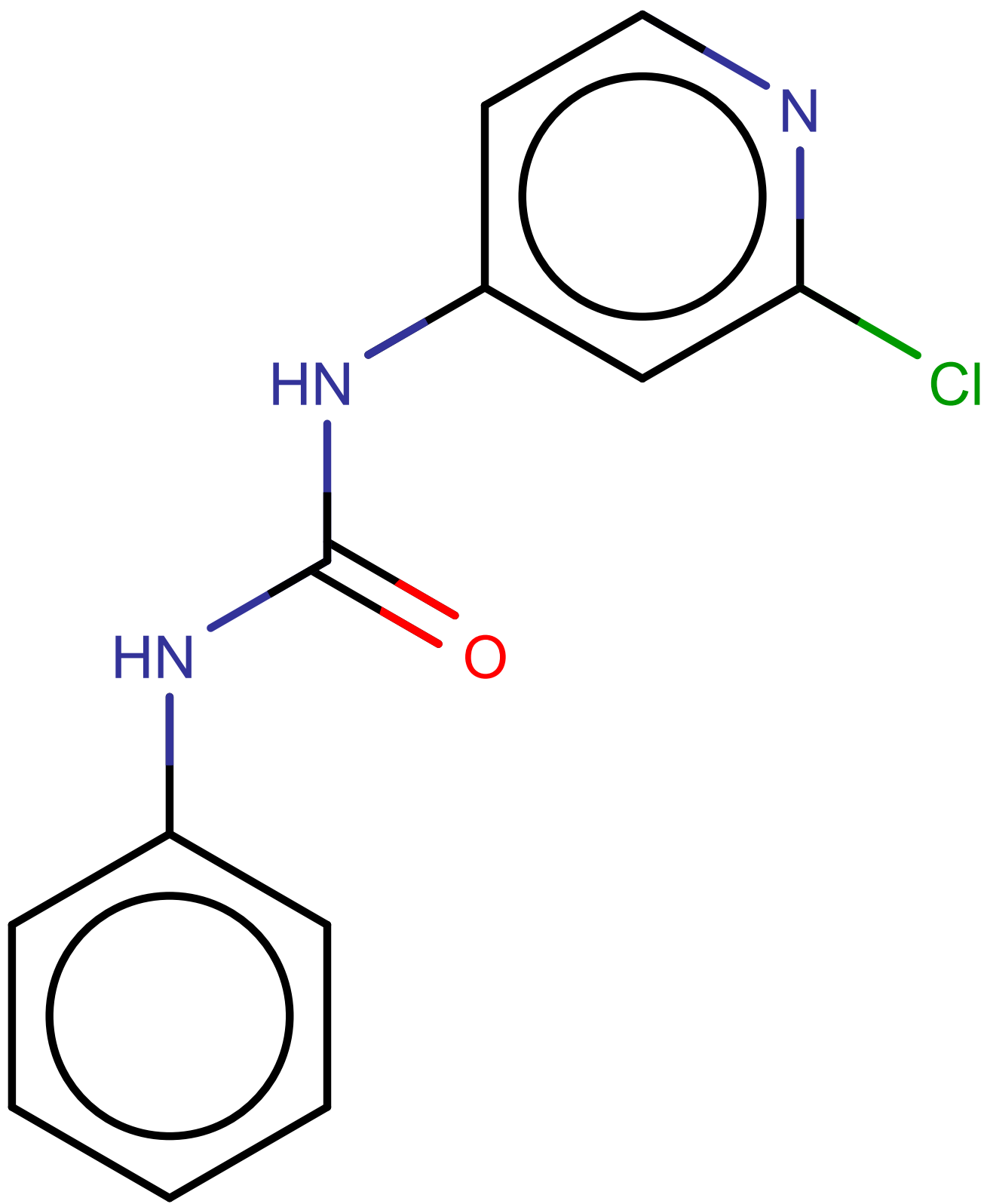

Supplement: Supplementary file 1 [file toxics-12-00425-s001.zip › Supplementary Materials/2D chemical structures/5638.pdf]

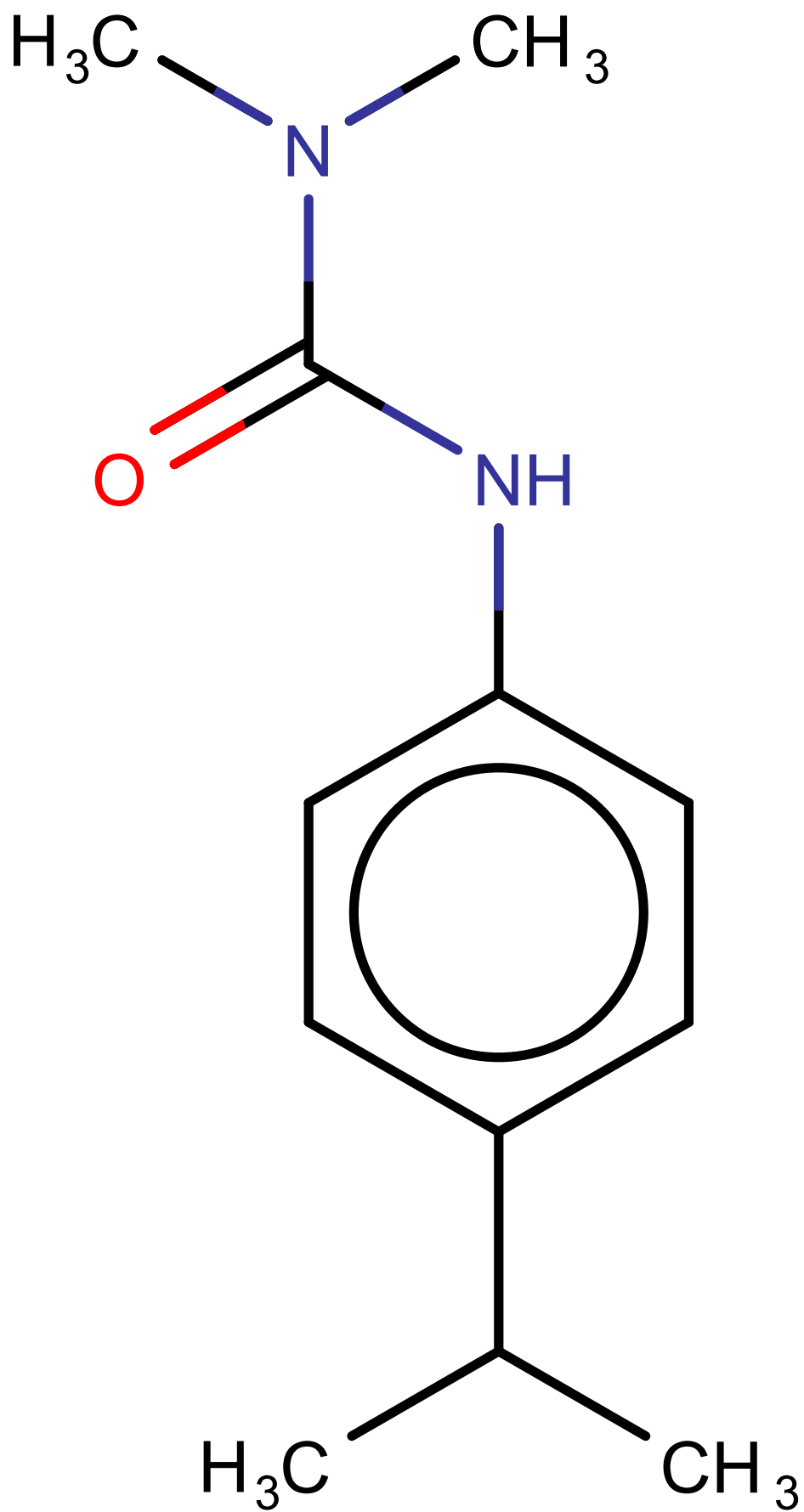

Supplement: Supplementary file 1 [file toxics-12-00425-s001.zip › Supplementary Materials/2D chemical structures/5668.pdf]

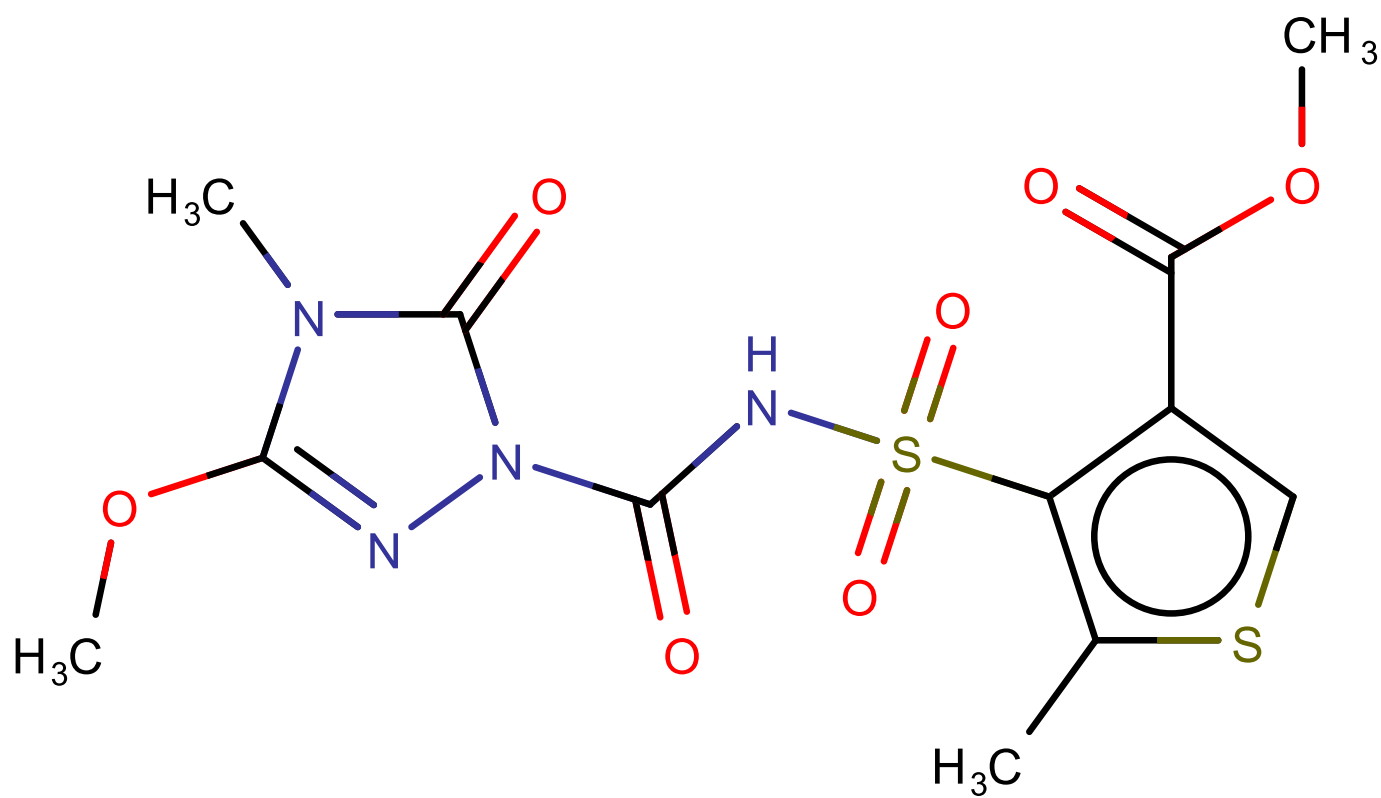

Supplement: Supplementary file 1 [file toxics-12-00425-s001.zip › Supplementary Materials/2D chemical structures/5827.pdf]

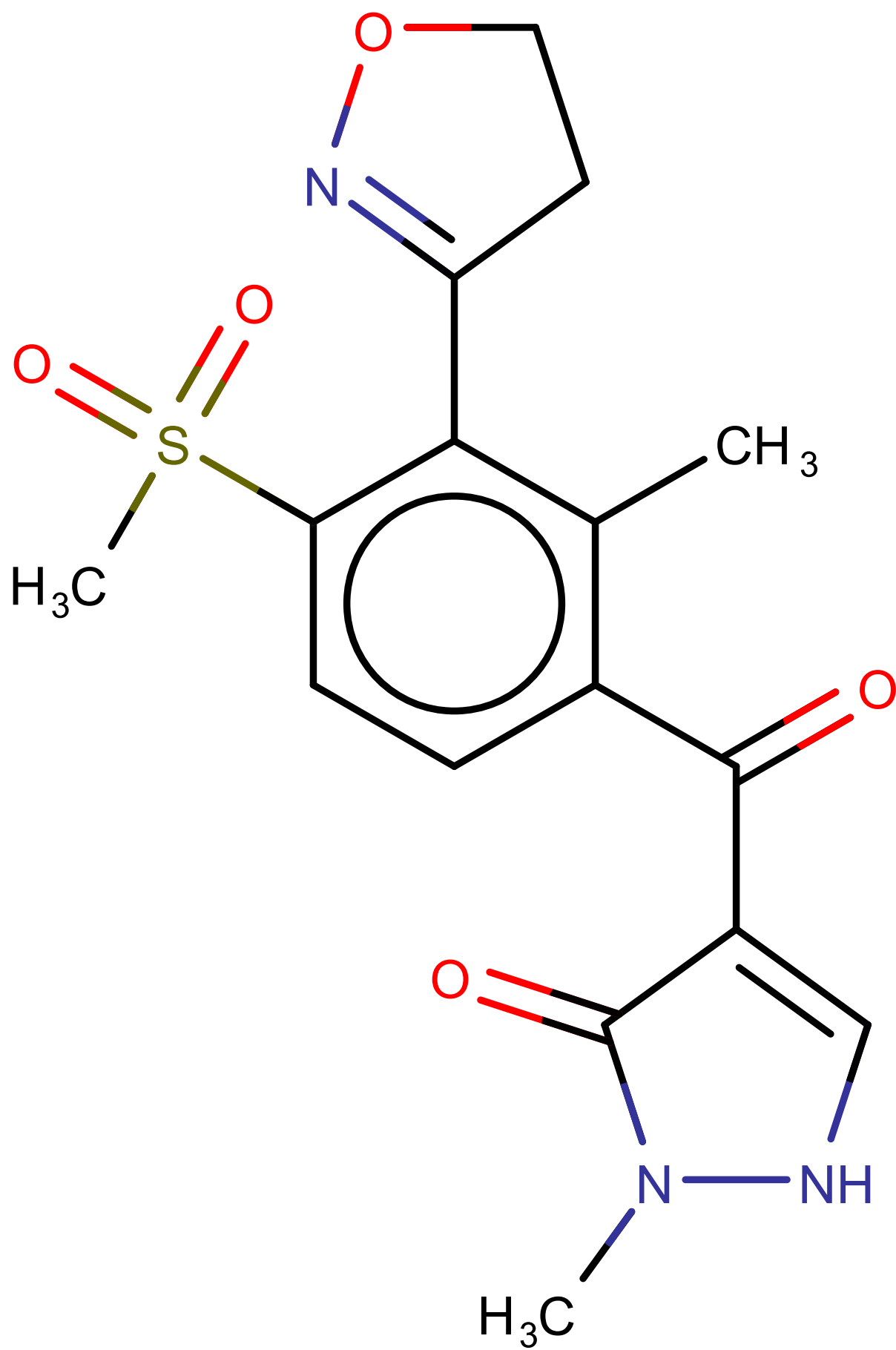

Supplement: Supplementary file 1 [file toxics-12-00425-s001.zip › Supplementary Materials/2D chemical structures/5829.pdf]

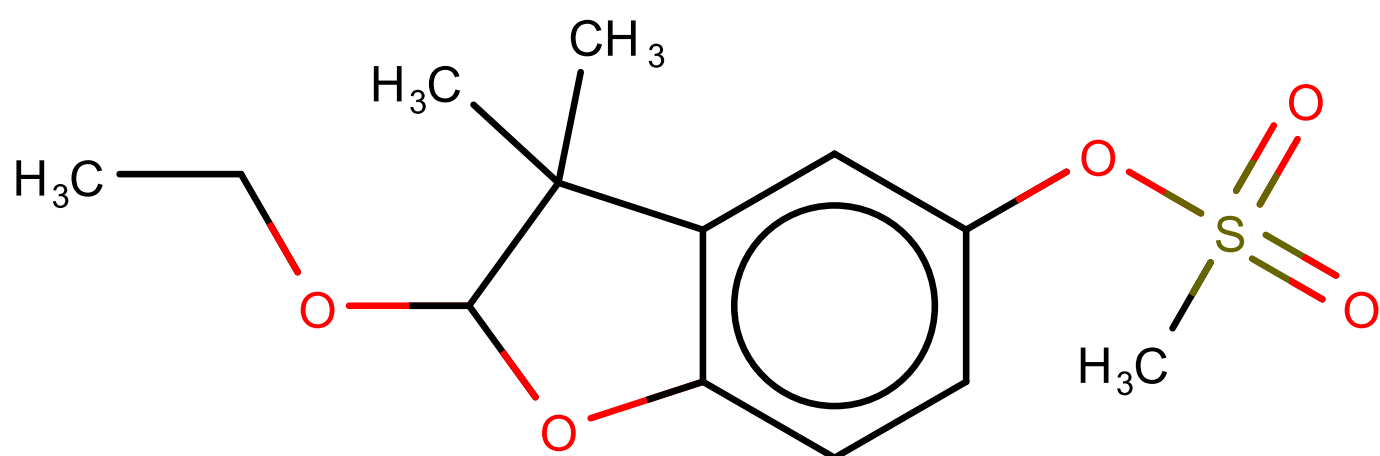

Supplement: Supplementary file 1 [file toxics-12-00425-s001.zip › Supplementary Materials/2D chemical structures/5834.pdf]

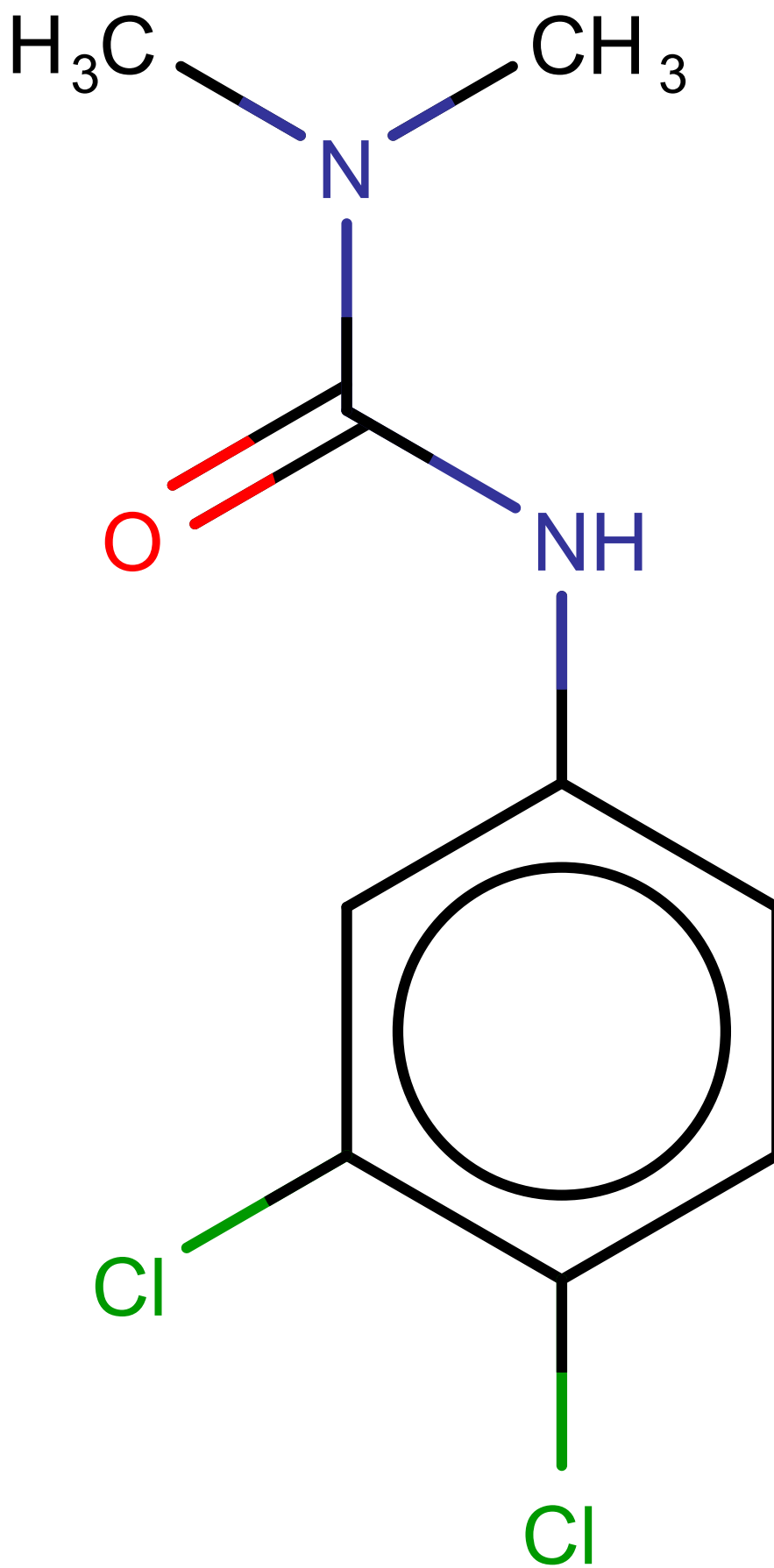

Supplement: Supplementary file 1 [file toxics-12-00425-s001.zip › Supplementary Materials/2D chemical structures/5838.pdf]

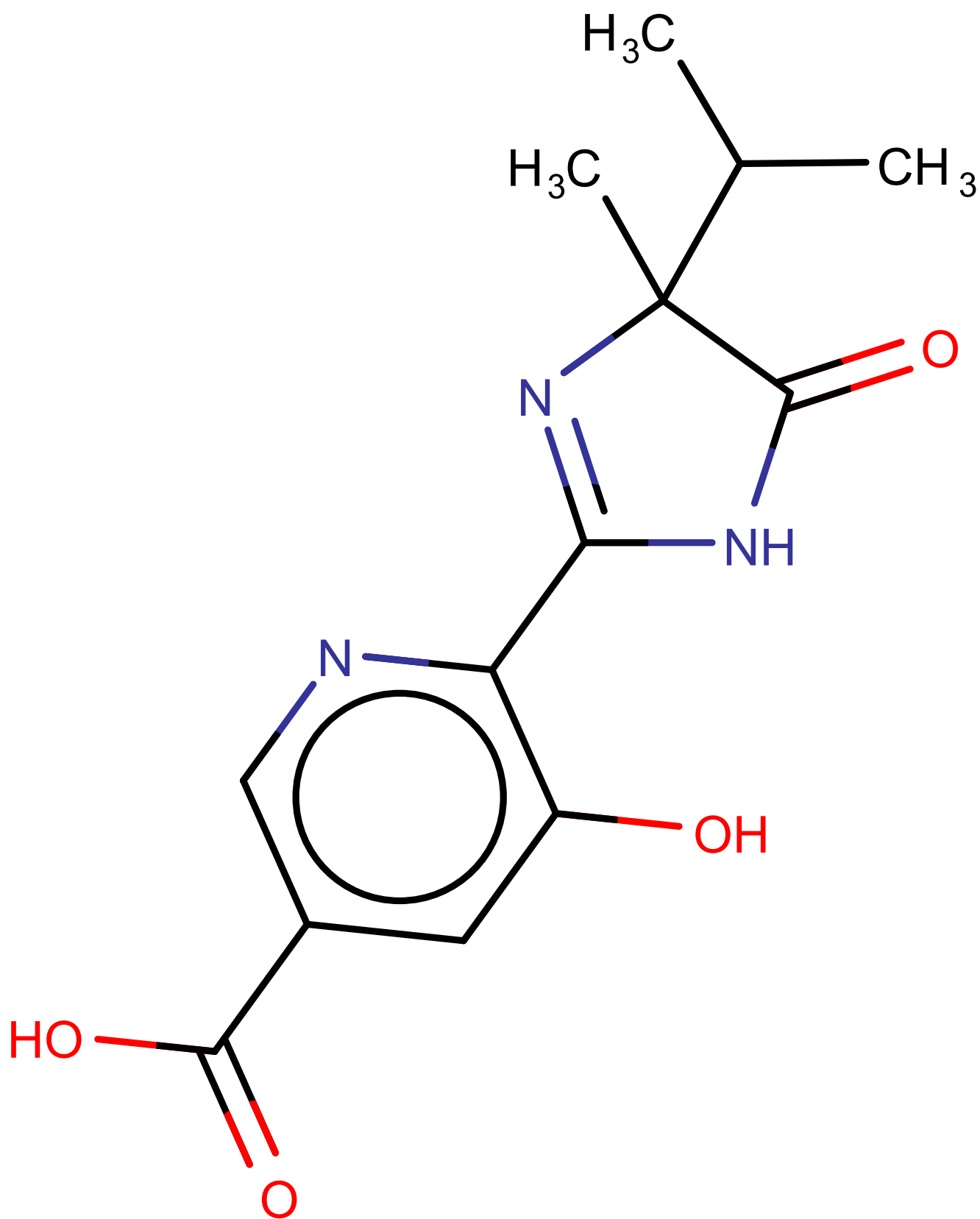

Supplement: Supplementary file 1 [file toxics-12-00425-s001.zip › Supplementary Materials/2D chemical structures/5970.pdf]

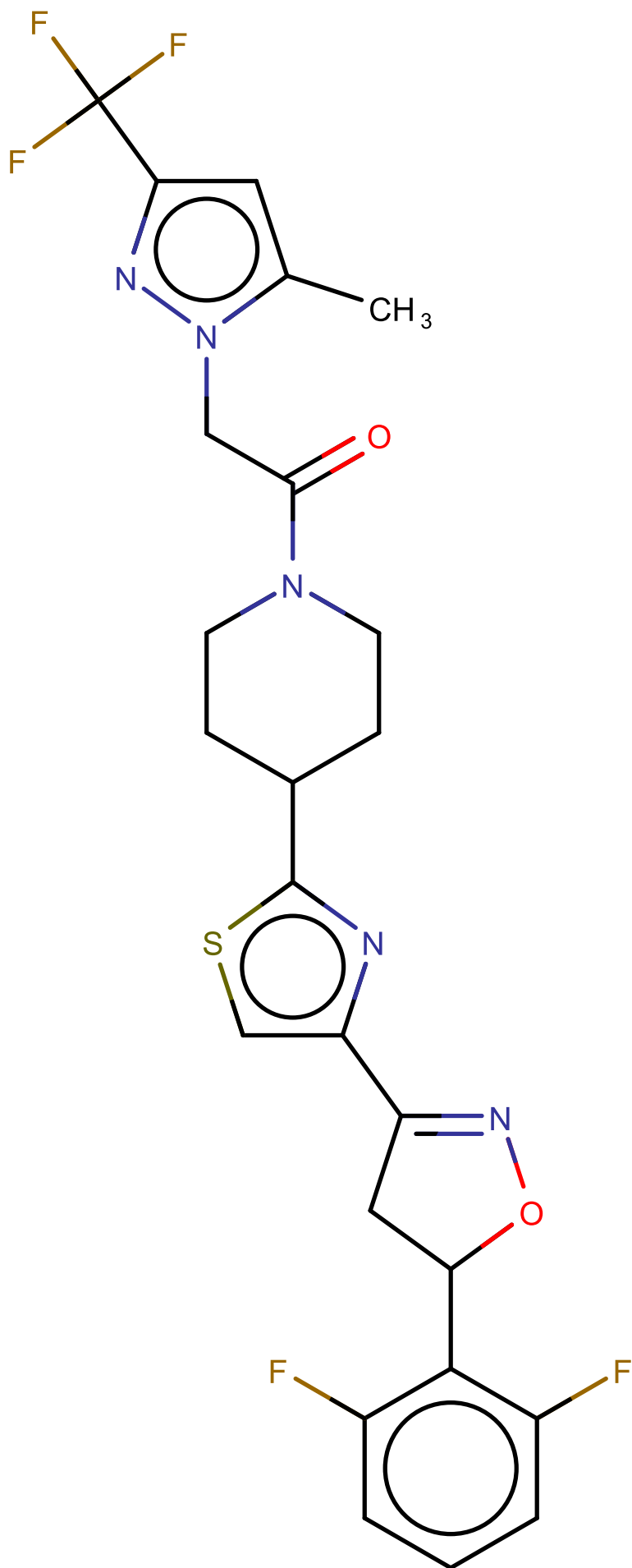

Supplement: Supplementary file 1 [file toxics-12-00425-s001.zip › Supplementary Materials/2D chemical structures/6068.pdf]

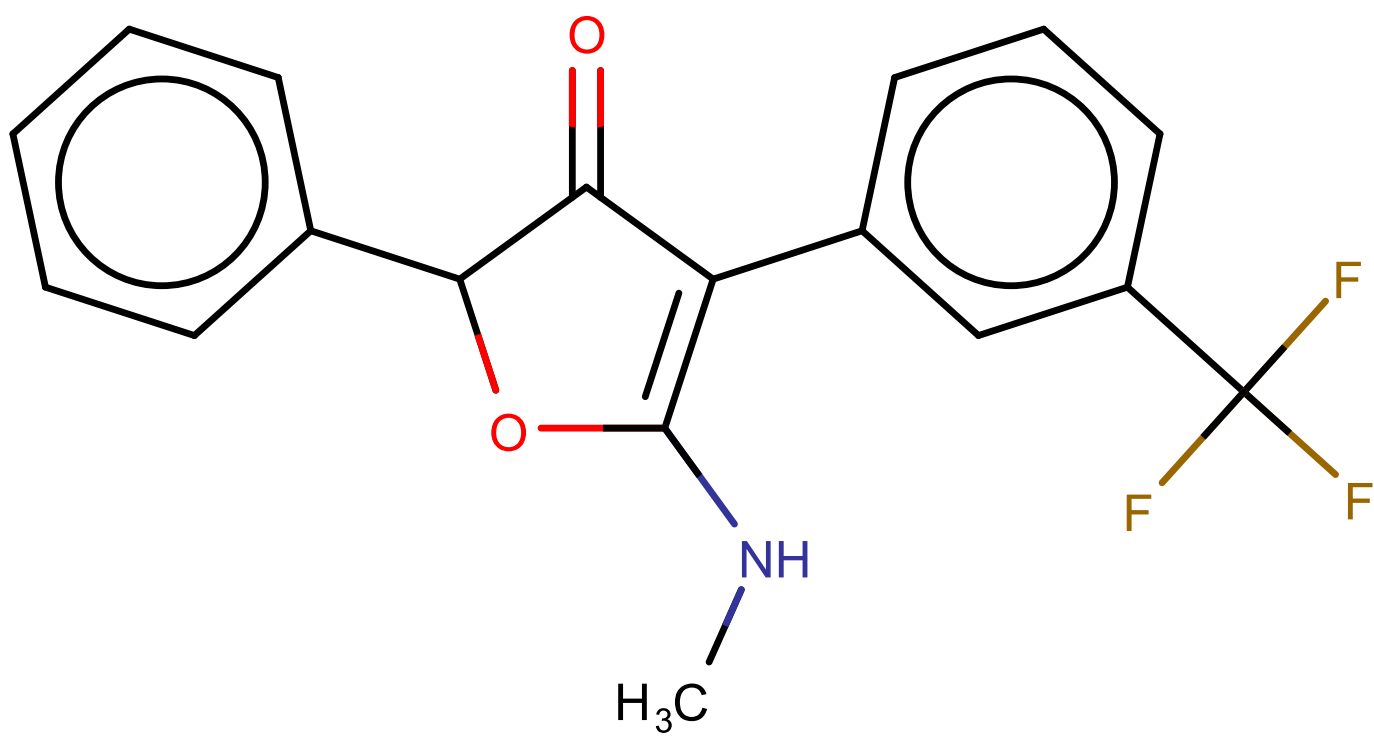

Supplement: Supplementary file 1 [file toxics-12-00425-s001.zip › Supplementary Materials/2D chemical structures/6078.pdf]

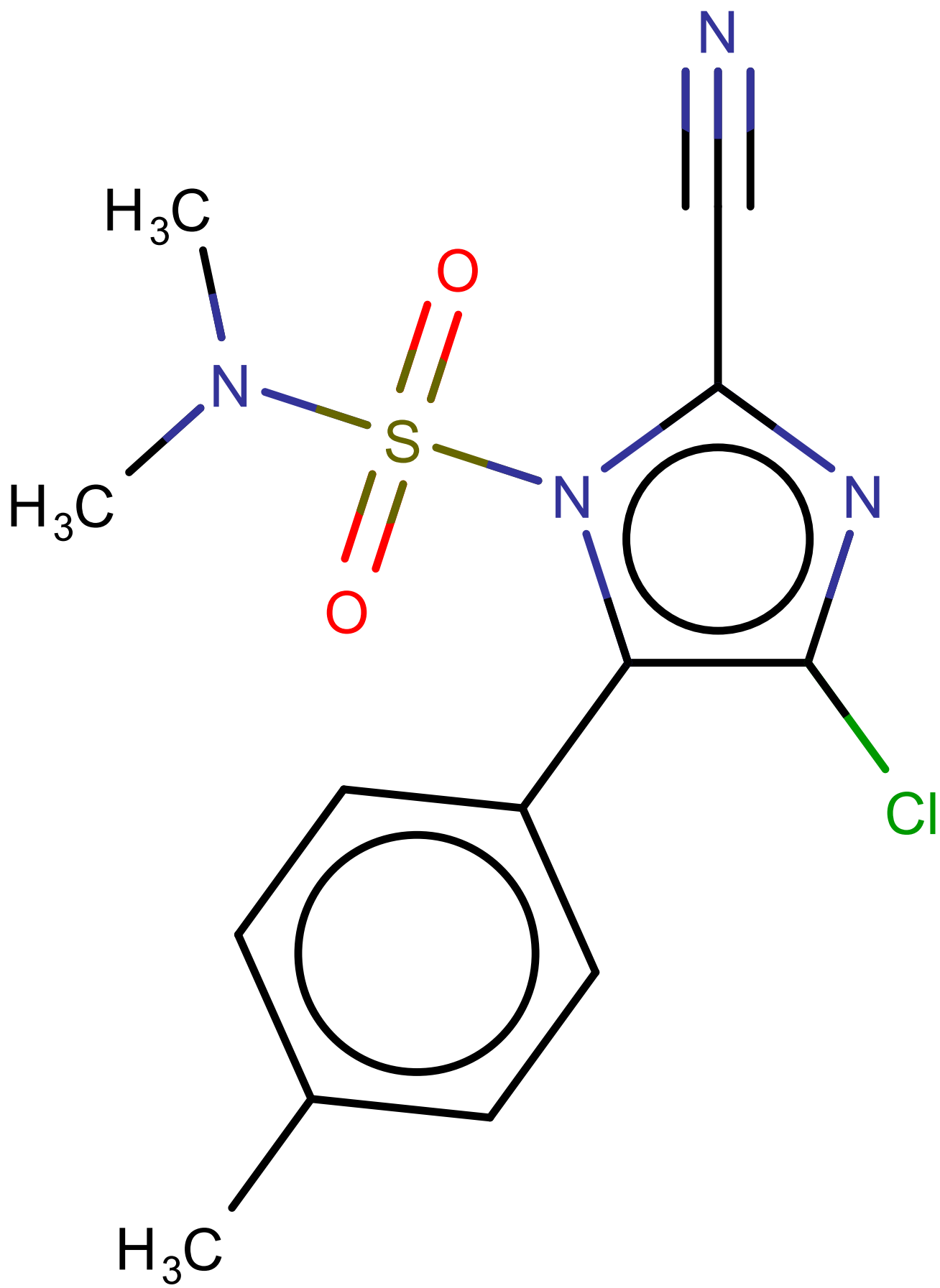

Supplement: Supplementary file 1 [file toxics-12-00425-s001.zip › Supplementary Materials/2D chemical structures/6080.pdf]

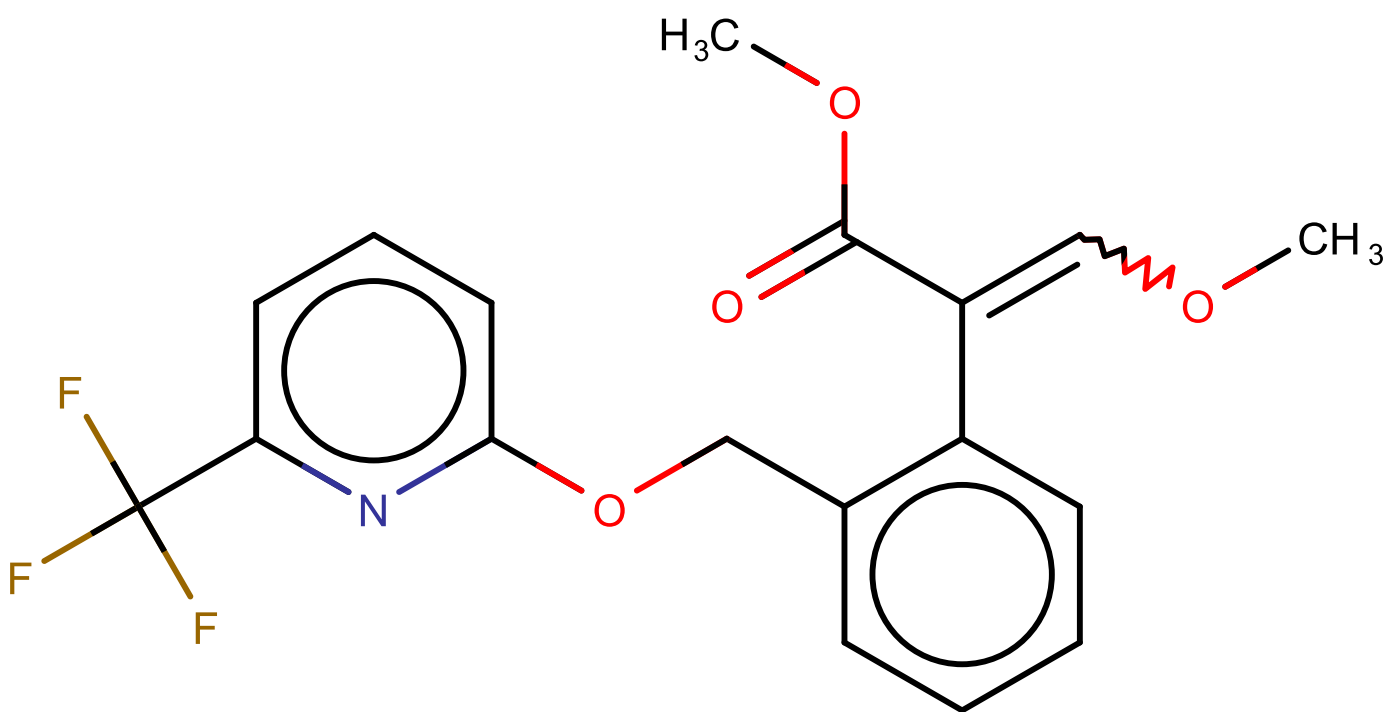

Supplement: Supplementary file 1 [file toxics-12-00425-s001.zip › Supplementary Materials/2D chemical structures/6084.pdf]

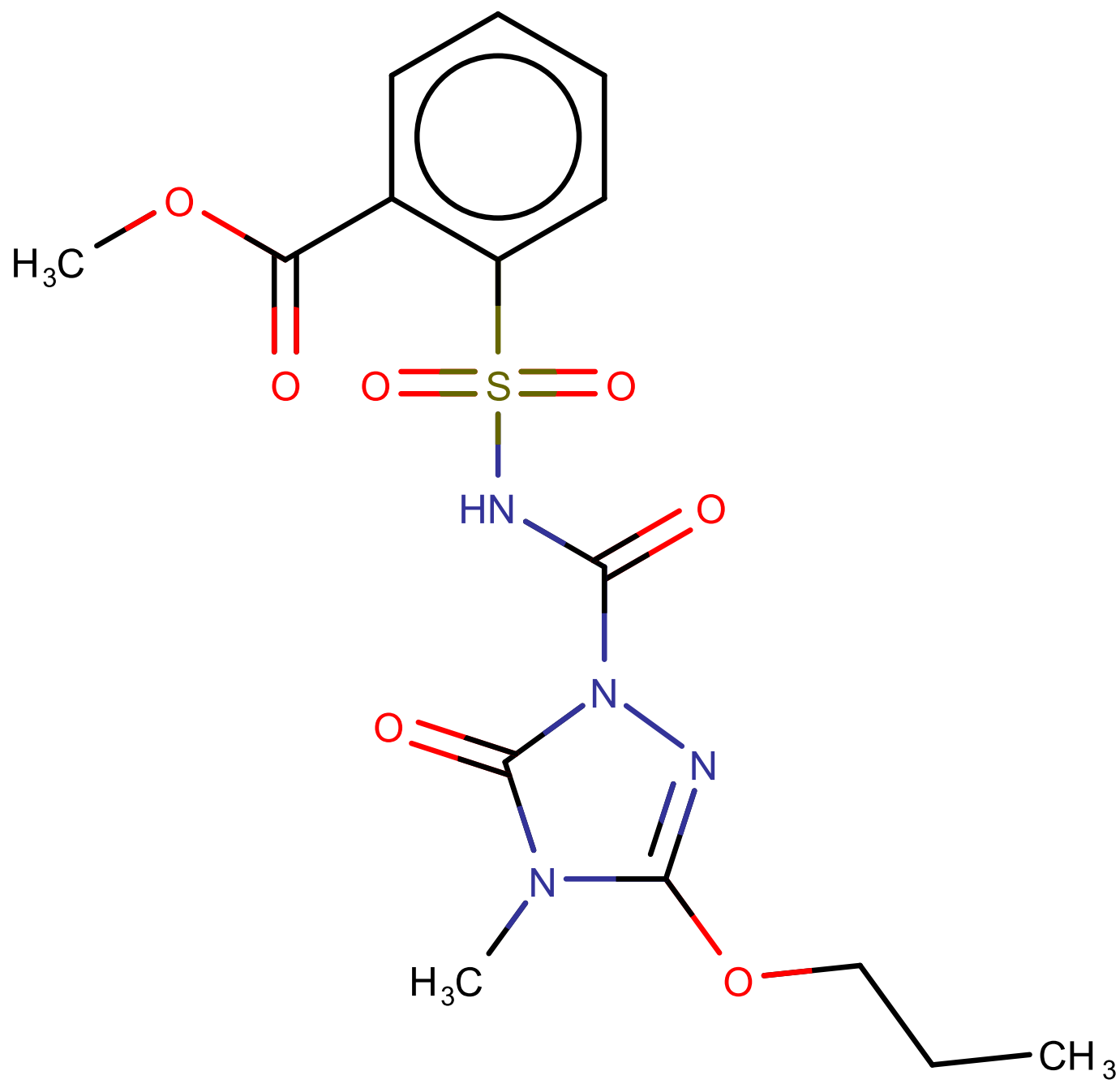

Supplement: Supplementary file 1 [file toxics-12-00425-s001.zip › Supplementary Materials/2D chemical structures/6242.pdf]

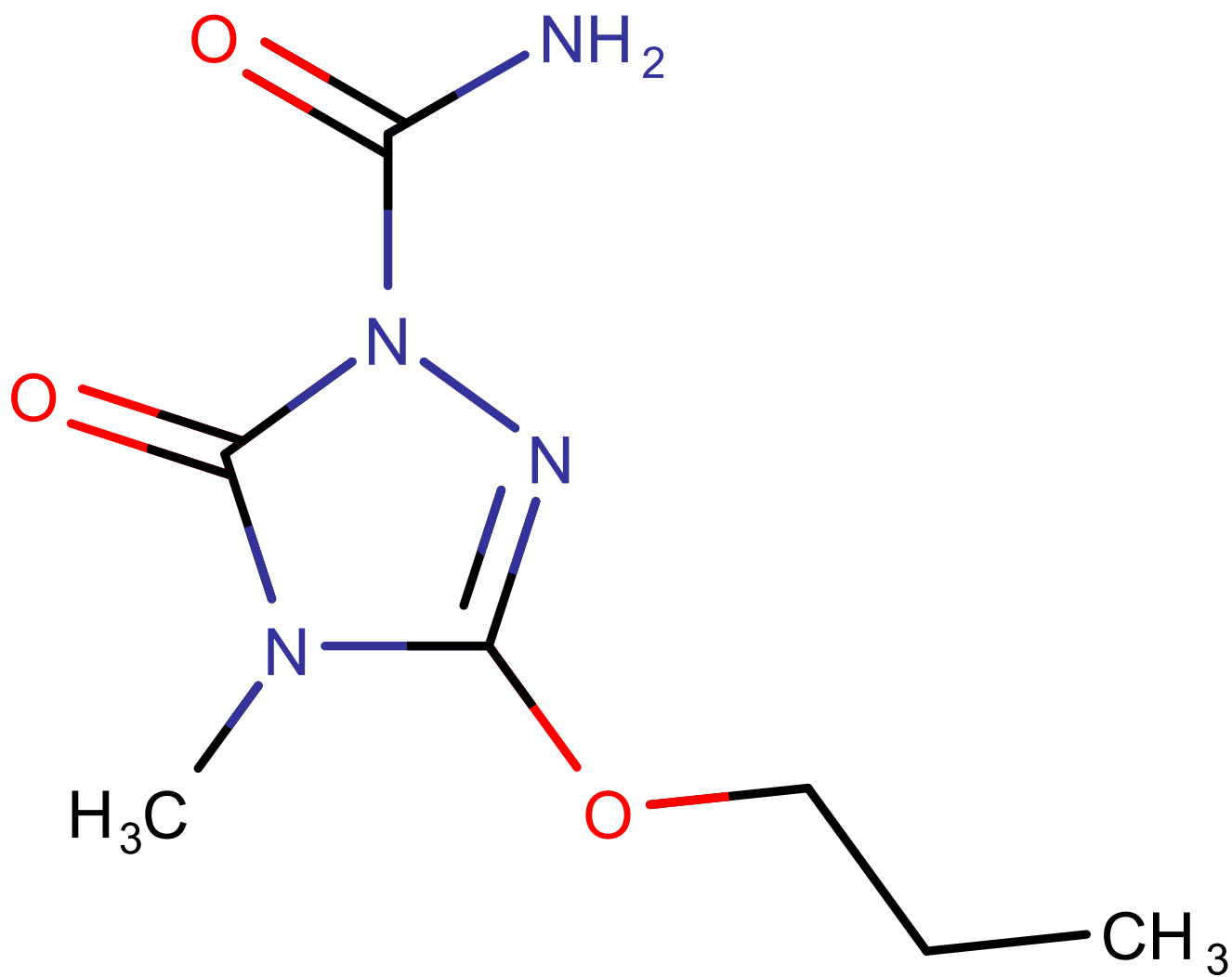

Supplement: Supplementary file 1 [file toxics-12-00425-s001.zip › Supplementary Materials/2D chemical structures/6288.pdf]

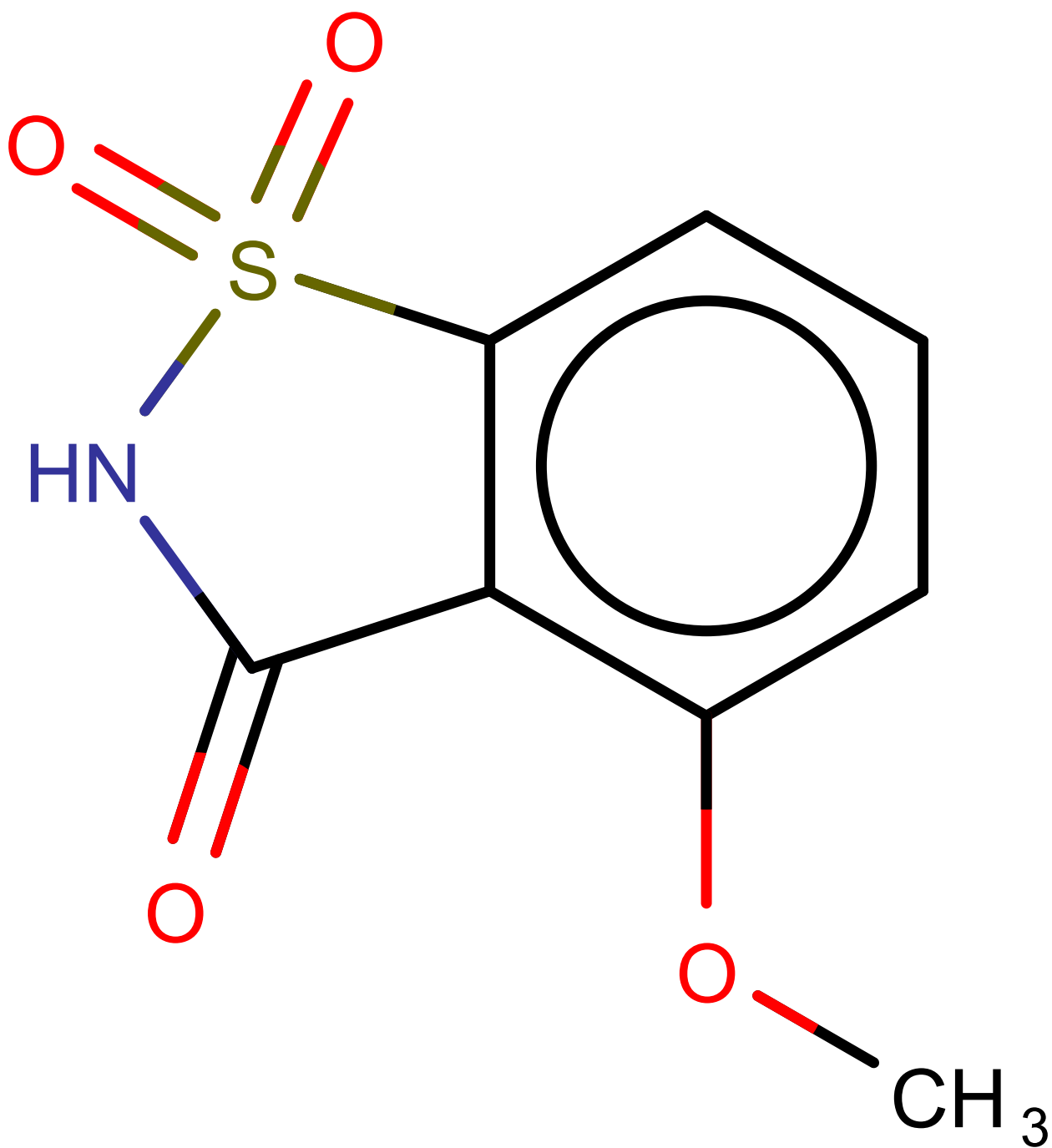

Supplement: Supplementary file 1 [file toxics-12-00425-s001.zip › Supplementary Materials/2D chemical structures/6292.pdf]

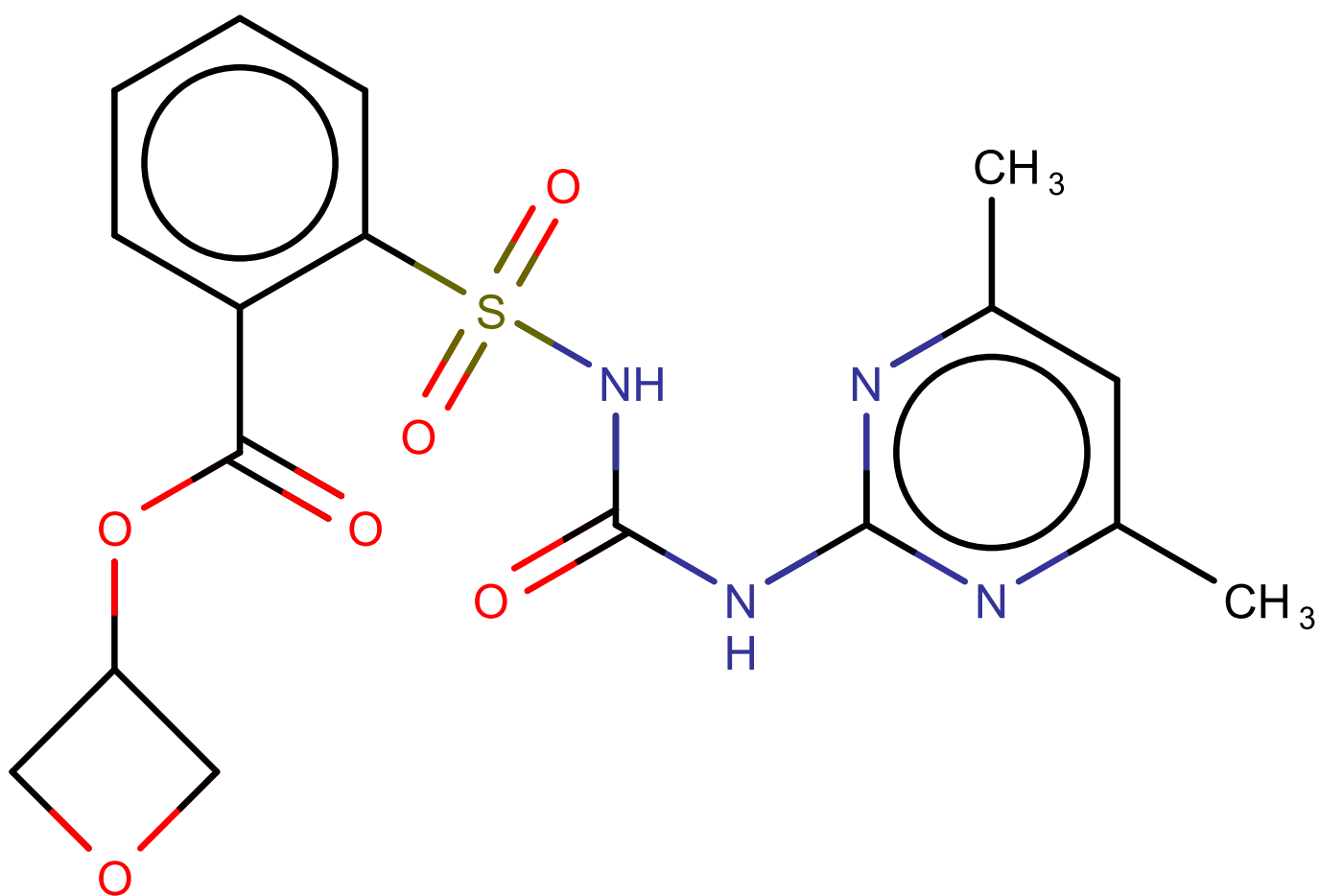

Supplement: Supplementary file 1 [file toxics-12-00425-s001.zip › Supplementary Materials/2D chemical structures/6423.pdf]

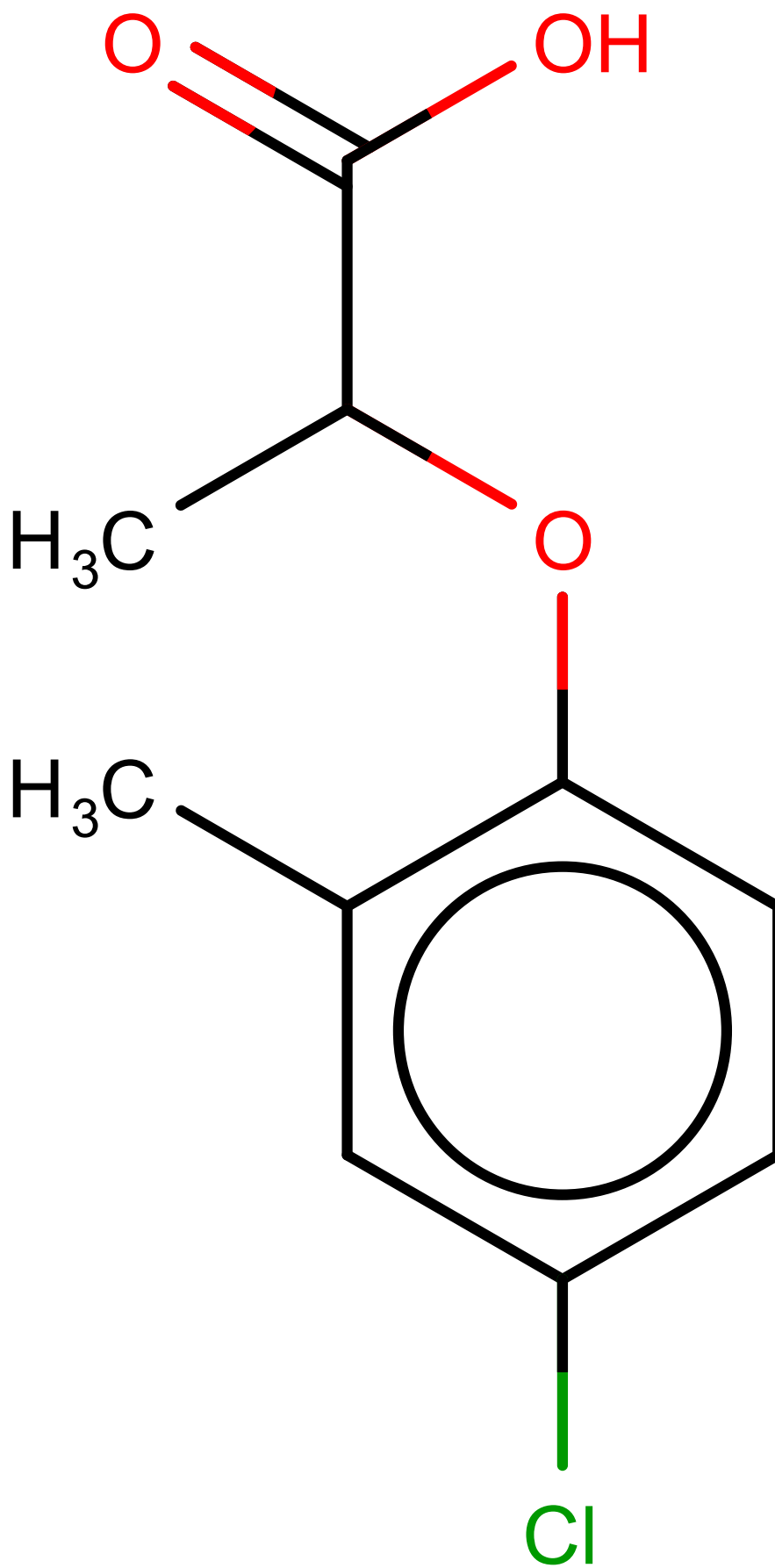

Supplement: Supplementary file 1 [file toxics-12-00425-s001.zip › Supplementary Materials/2D chemical structures/6460.pdf]

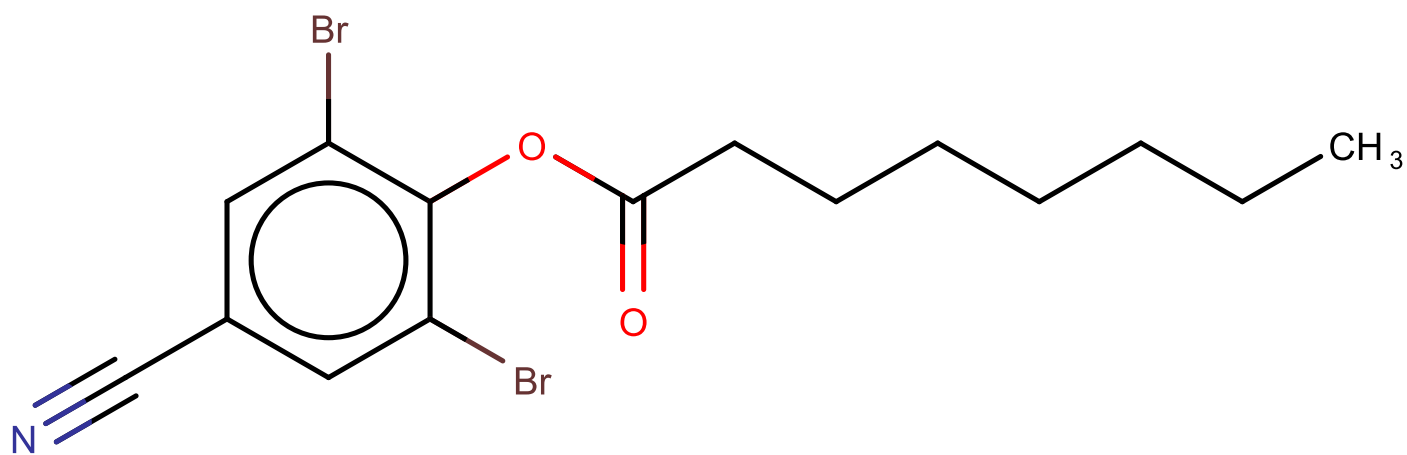

Supplement: Supplementary file 1 [file toxics-12-00425-s001.zip › Supplementary Materials/2D chemical structures/6473.pdf]

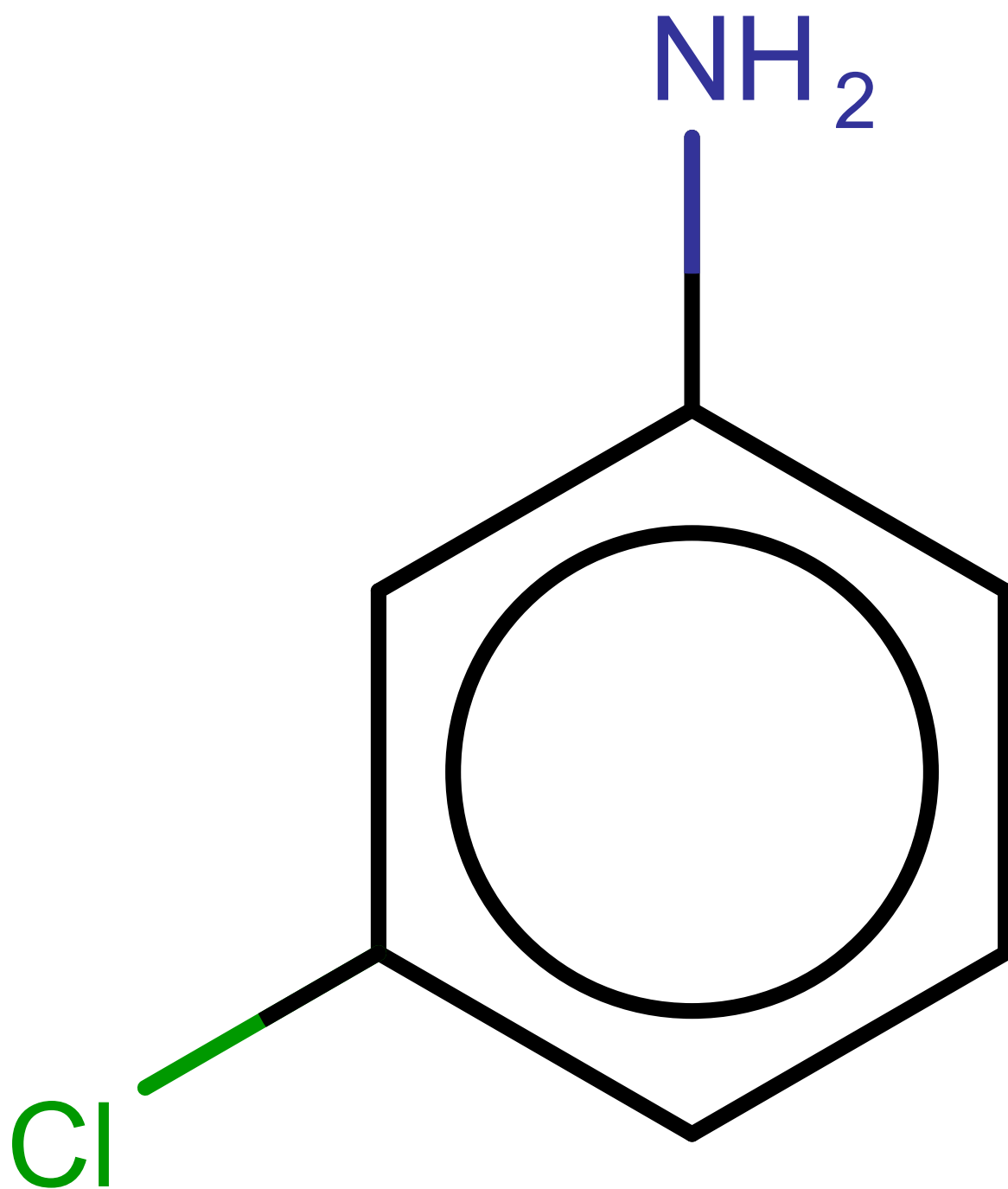

Supplement: Supplementary file 1 [file toxics-12-00425-s001.zip › Supplementary Materials/2D chemical structures/6522.pdf]

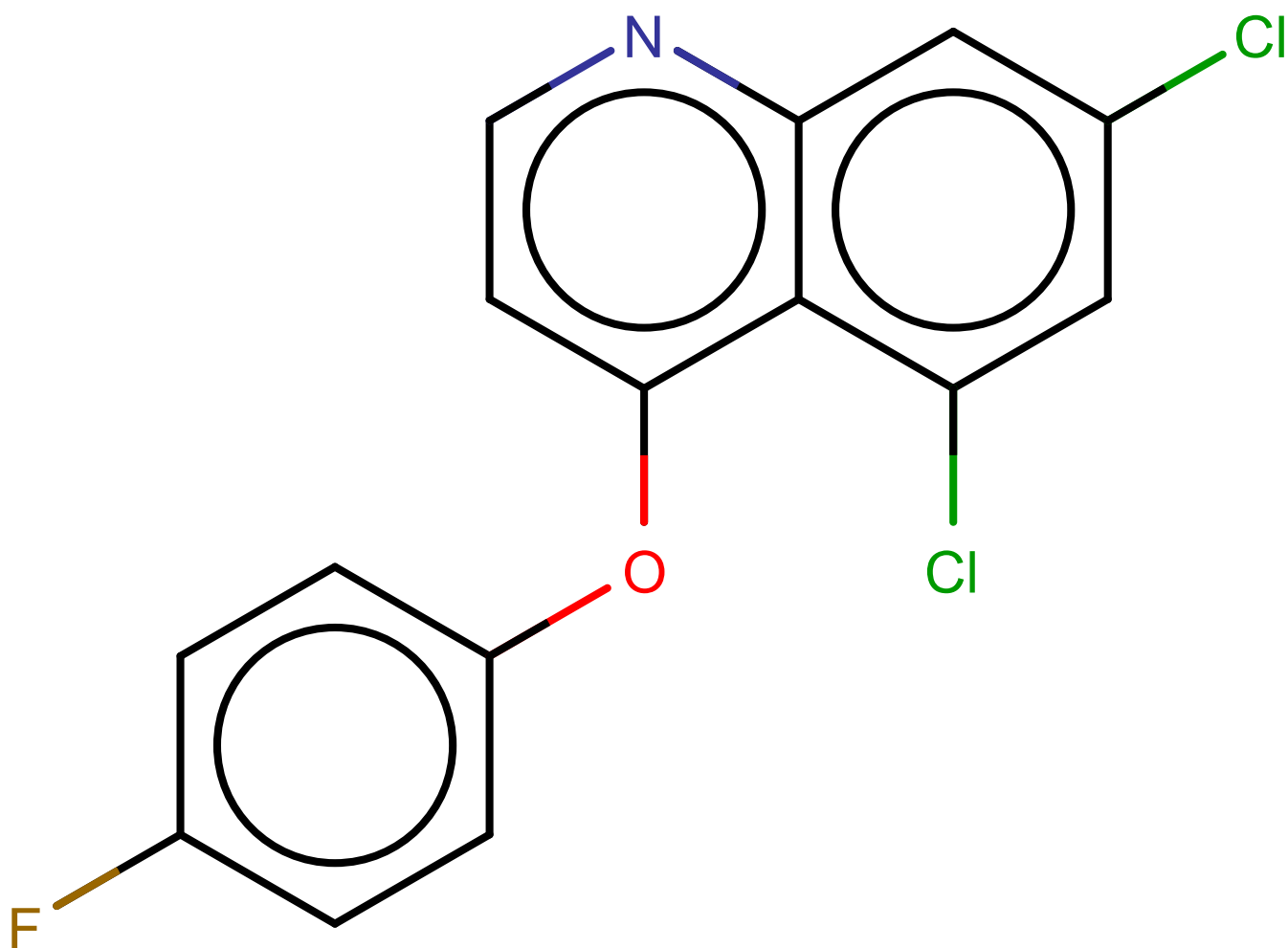

Supplement: Supplementary file 1 [file toxics-12-00425-s001.zip › Supplementary Materials/2D chemical structures/6840.pdf]

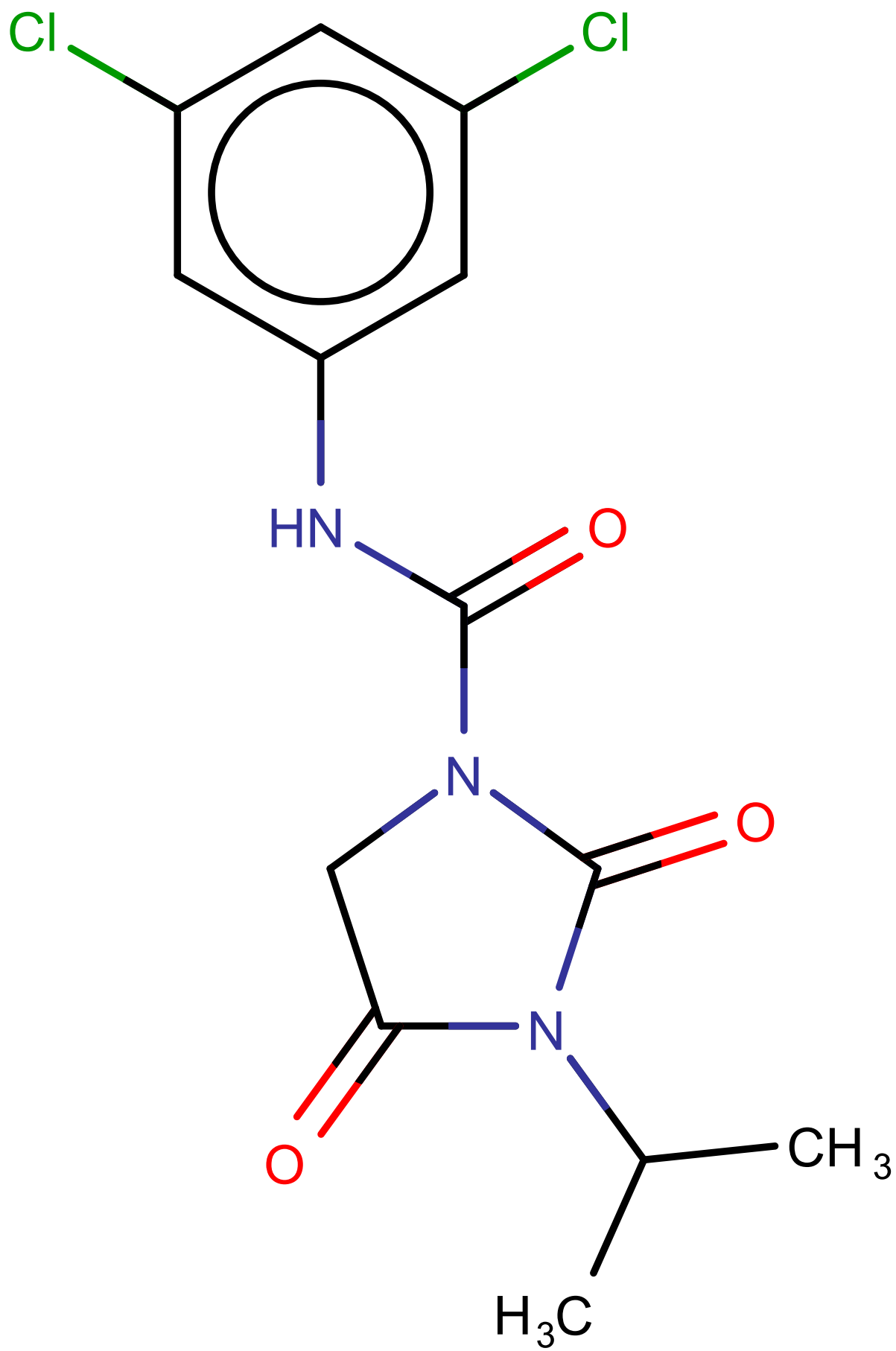

Supplement: Supplementary file 1 [file toxics-12-00425-s001.zip › Supplementary Materials/2D chemical structures/6908.pdf]

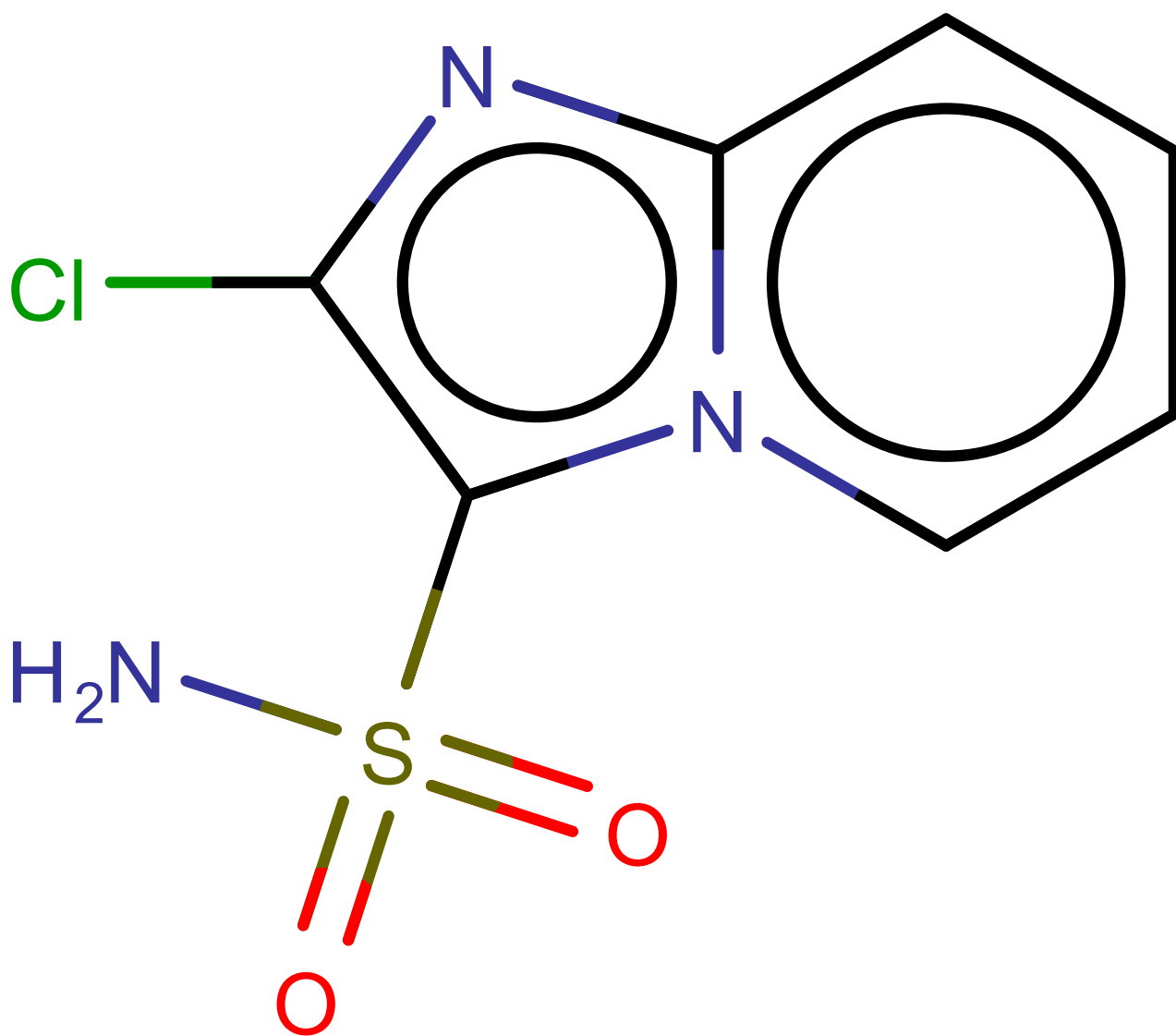

Supplement: Supplementary file 1 [file toxics-12-00425-s001.zip › Supplementary Materials/2D chemical structures/7128.pdf]
