# Supplementary figures and images for: Models for the No-Observed-Effect Concentration (NOEC) and Maximal Half-Effective Concentration (EC50) (part 1 of 2)
Source: Toxics. 2024 Jun 12;12(6):425. doi: 10.3390/toxics12060425 (PMC11209108; doi:10.3390/toxics12060425)

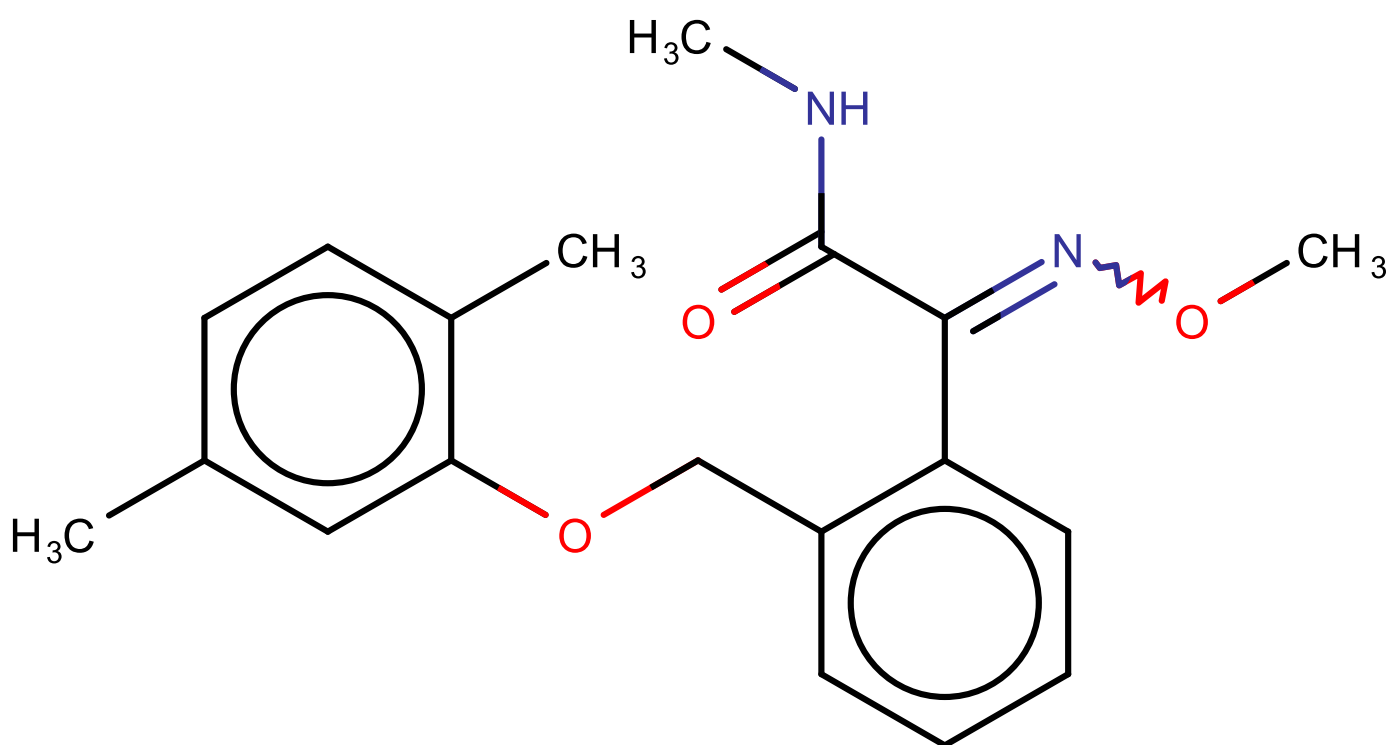

Supplement: Supplementary file 1 [file toxics-12-00425-s001.zip › Supplementary Materials/2D chemical structures/1668.pdf]

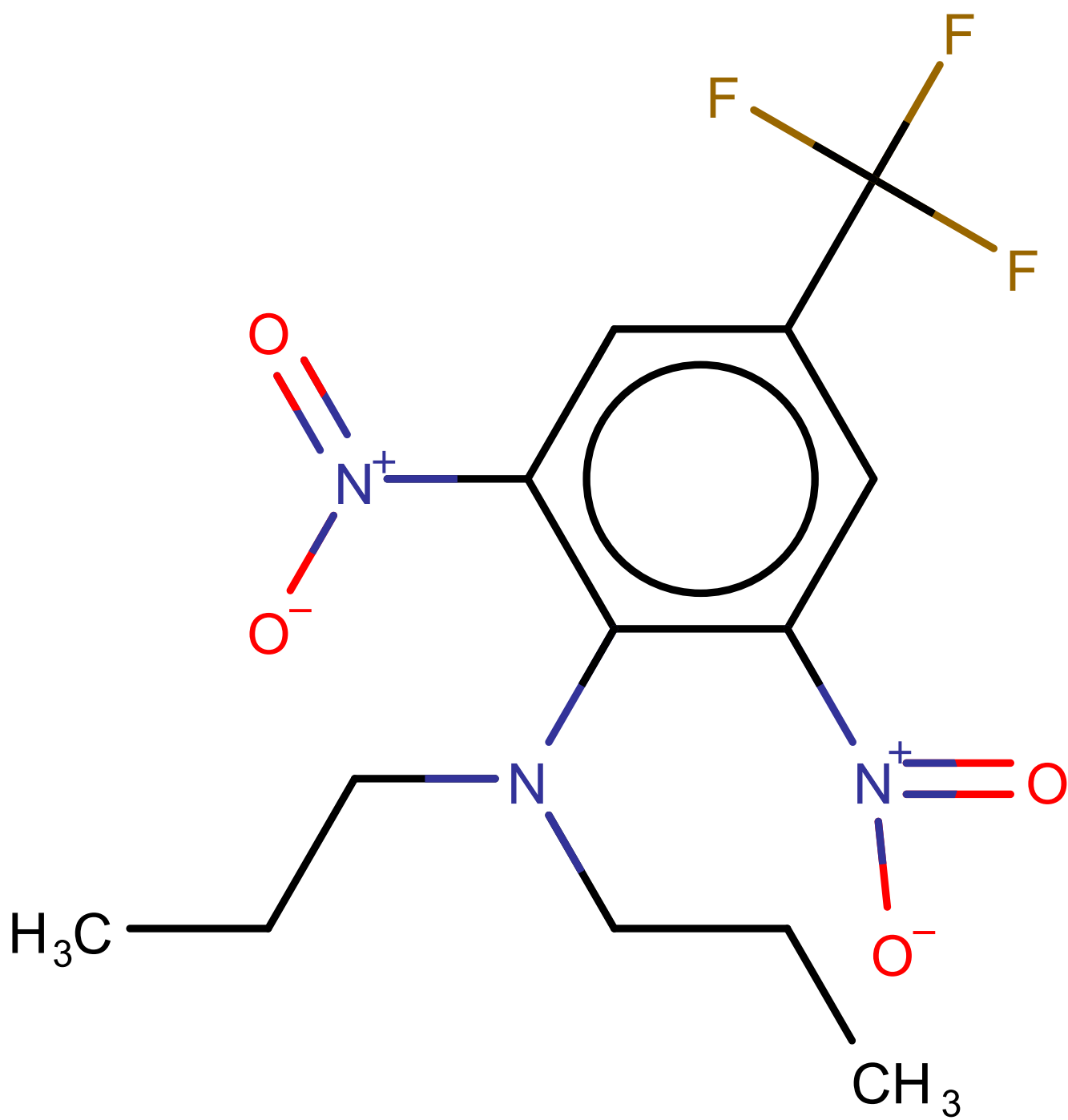

Supplement: Supplementary file 1 [file toxics-12-00425-s001.zip › Supplementary Materials/2D chemical structures/1680.pdf]

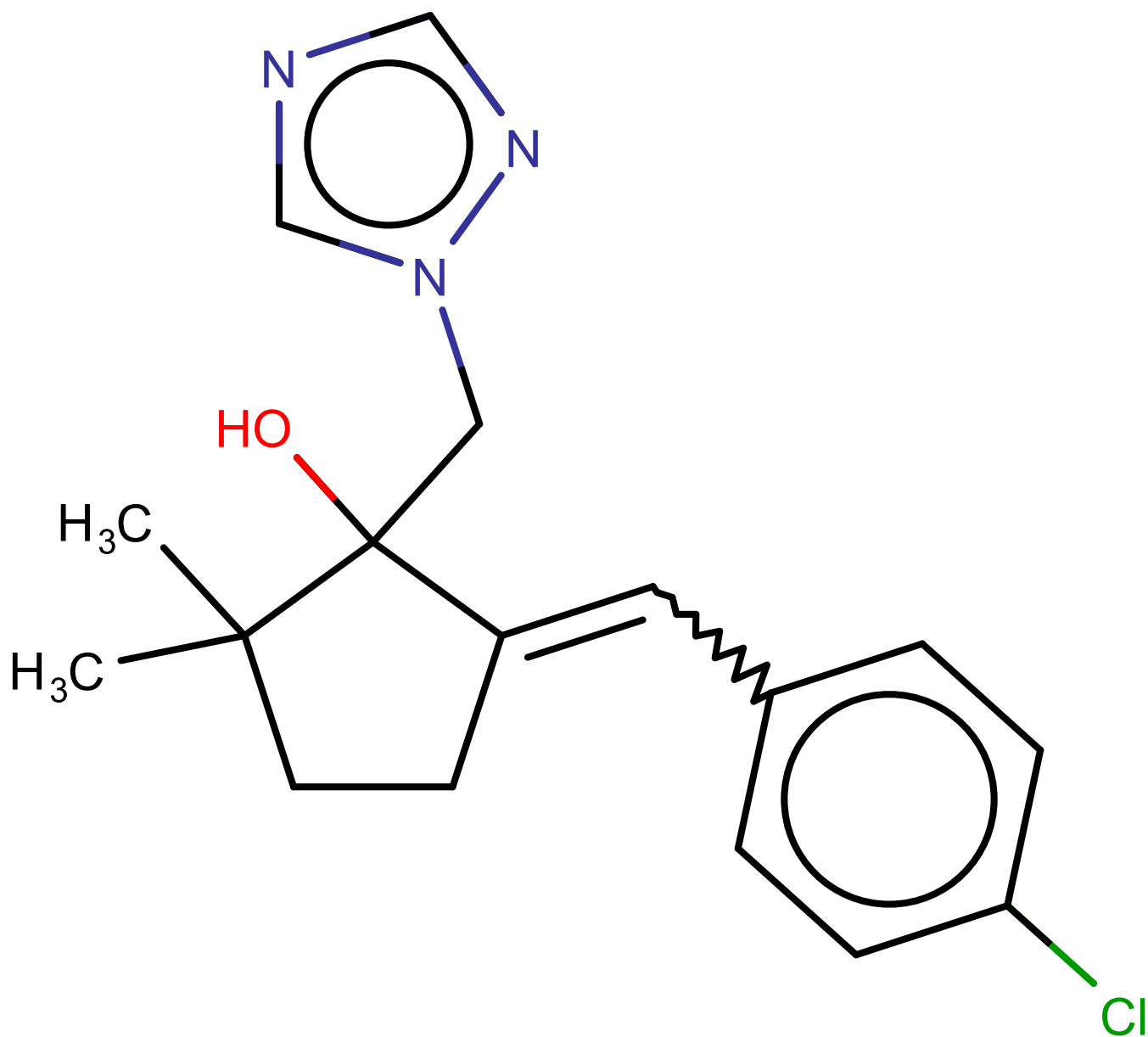

Supplement: Supplementary file 1 [file toxics-12-00425-s001.zip › Supplementary Materials/2D chemical structures/1686.pdf]

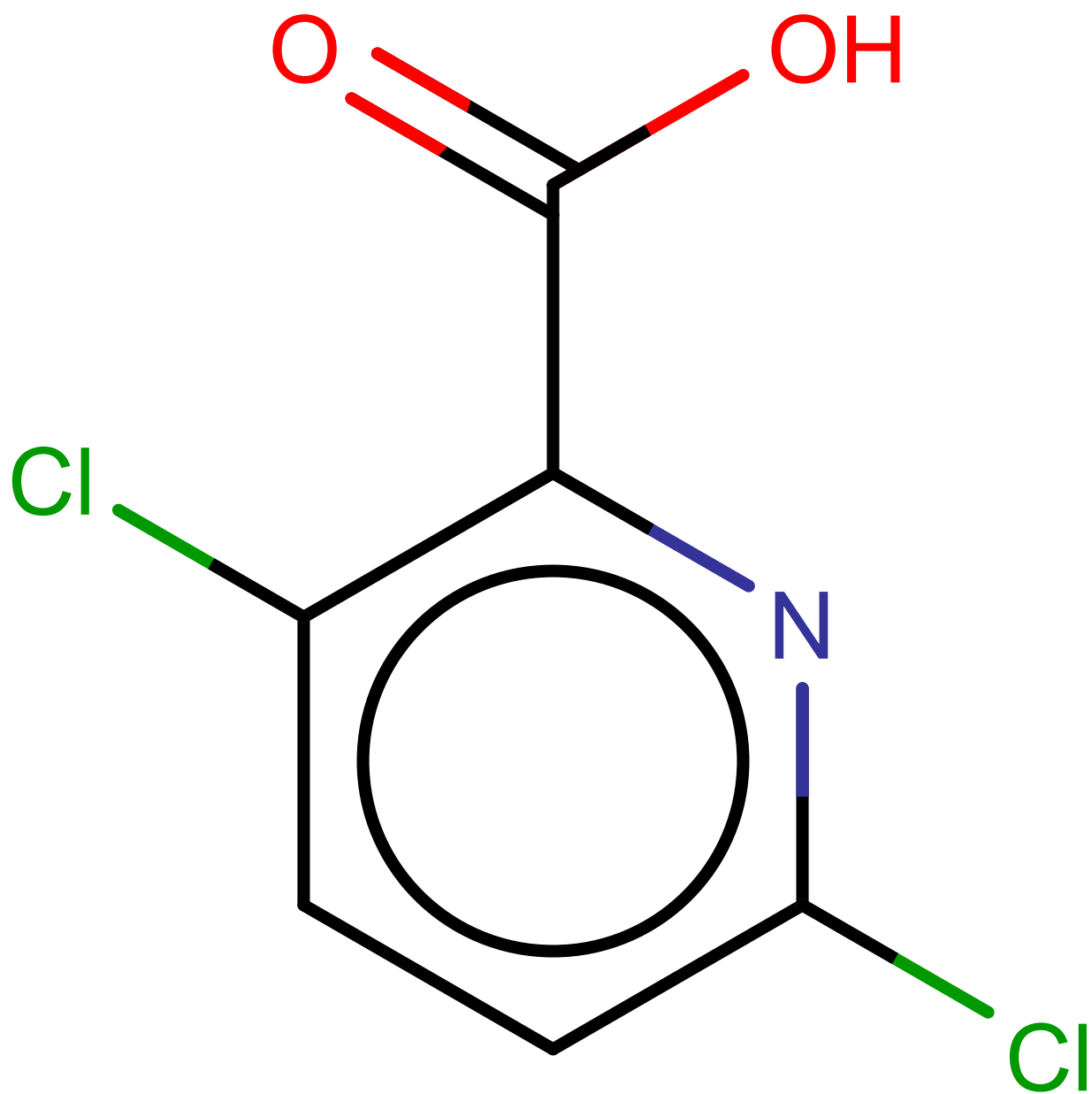

Supplement: Supplementary file 1 [file toxics-12-00425-s001.zip › Supplementary Materials/2D chemical structures/1688.pdf]

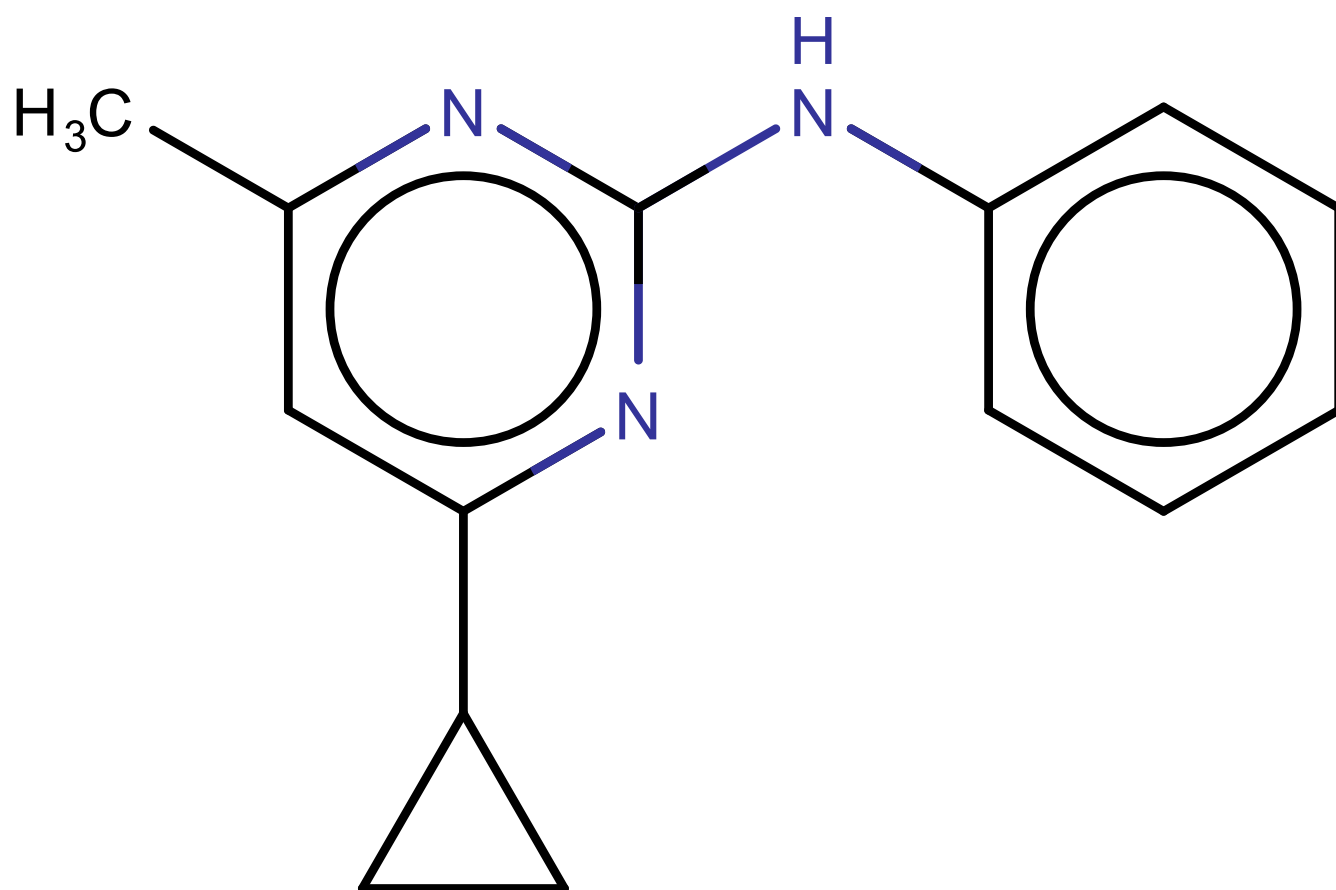

Supplement: Supplementary file 1 [file toxics-12-00425-s001.zip › Supplementary Materials/2D chemical structures/1689.pdf]

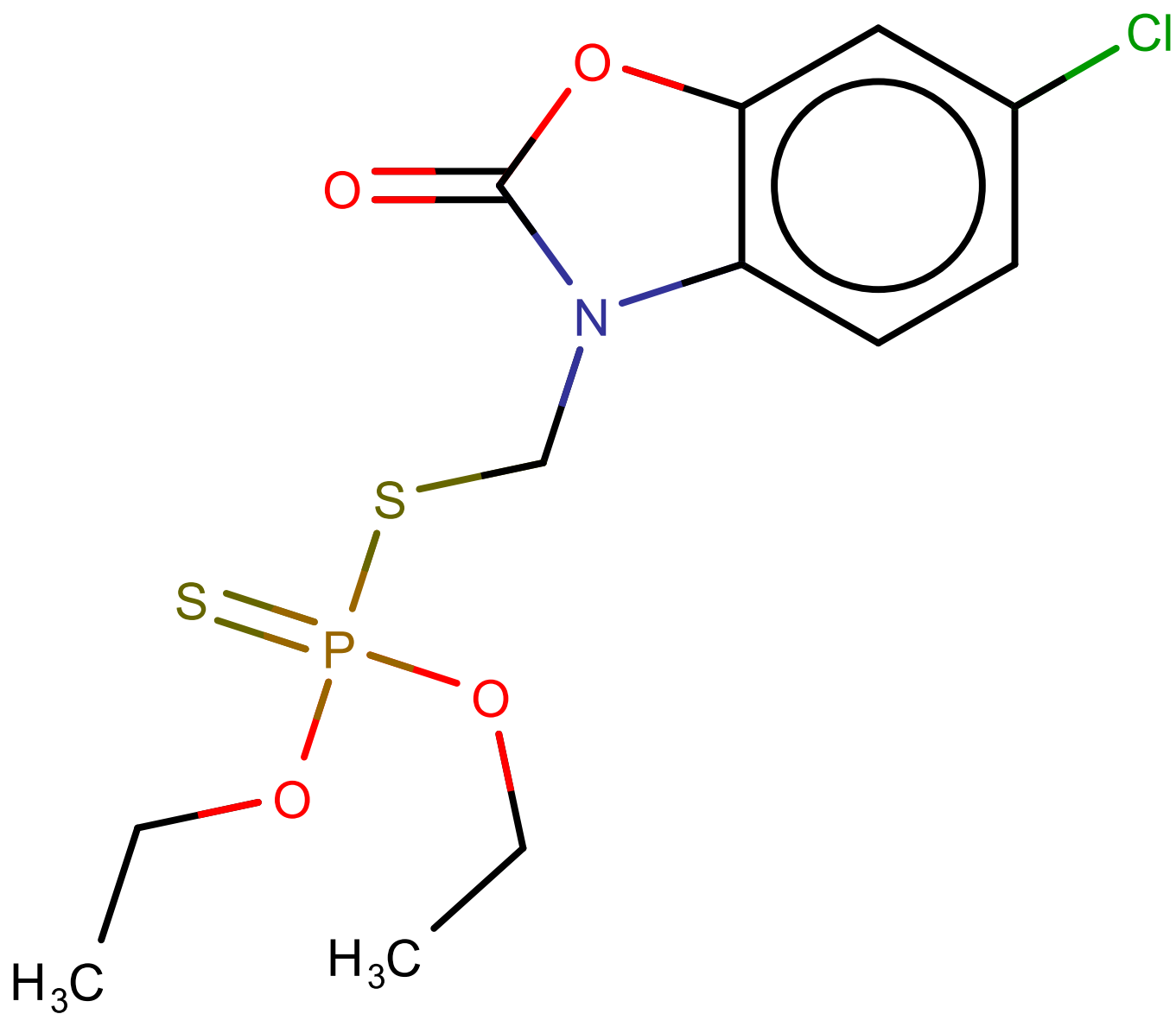

Supplement: Supplementary file 1 [file toxics-12-00425-s001.zip › Supplementary Materials/2D chemical structures/1693.pdf]

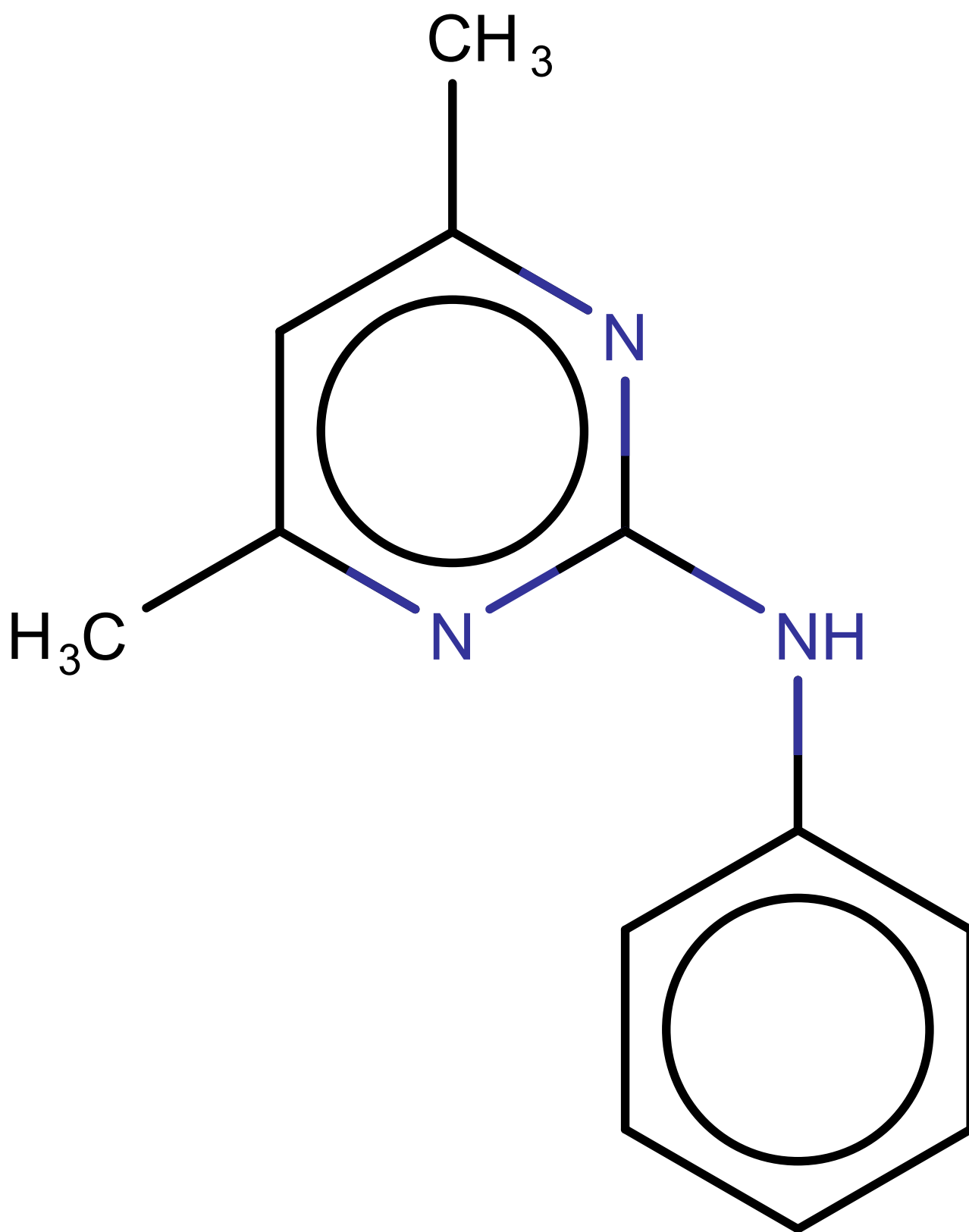

Supplement: Supplementary file 1 [file toxics-12-00425-s001.zip › Supplementary Materials/2D chemical structures/1696.pdf]

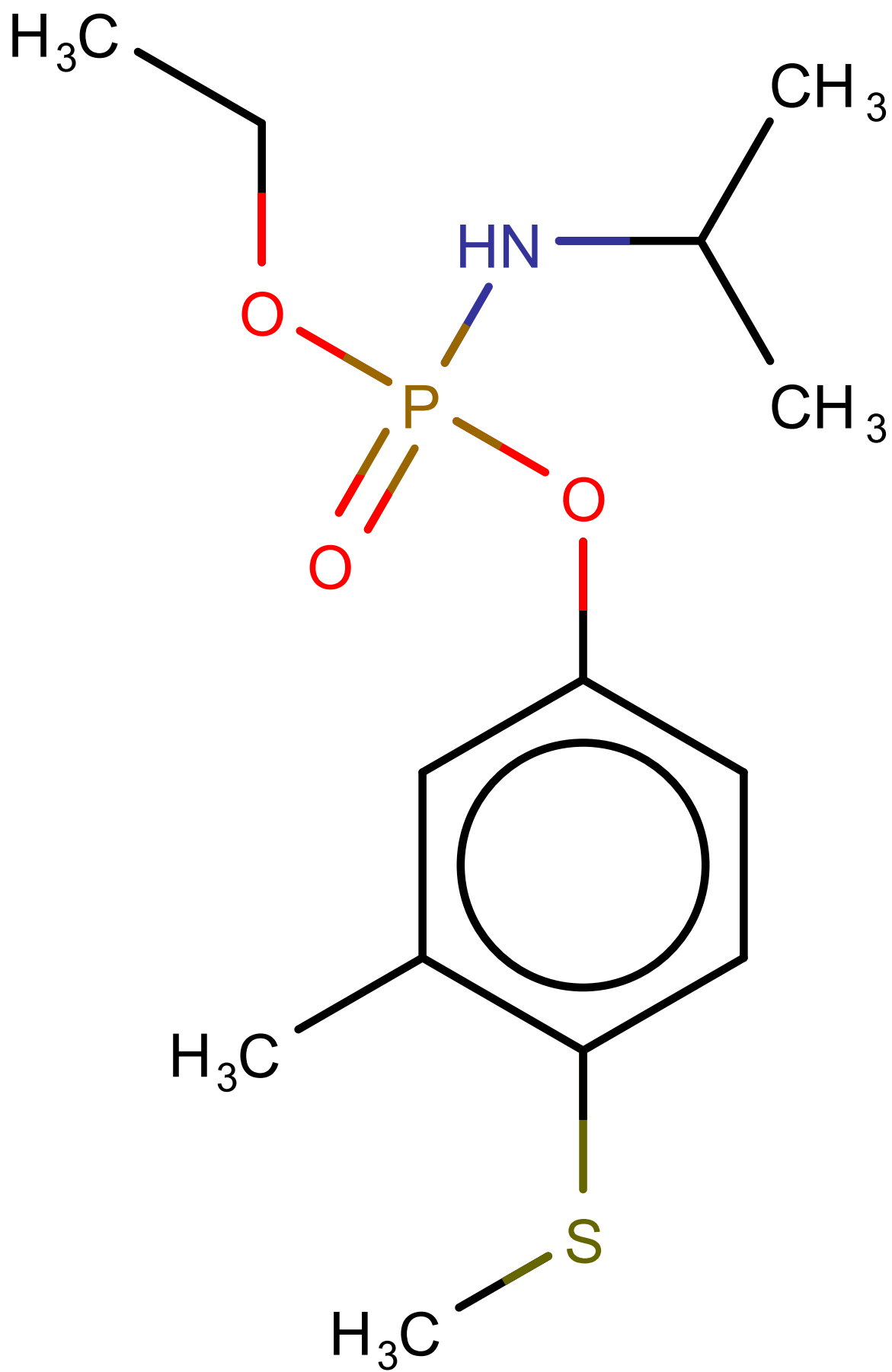

Supplement: Supplementary file 1 [file toxics-12-00425-s001.zip › Supplementary Materials/2D chemical structures/1697.pdf]

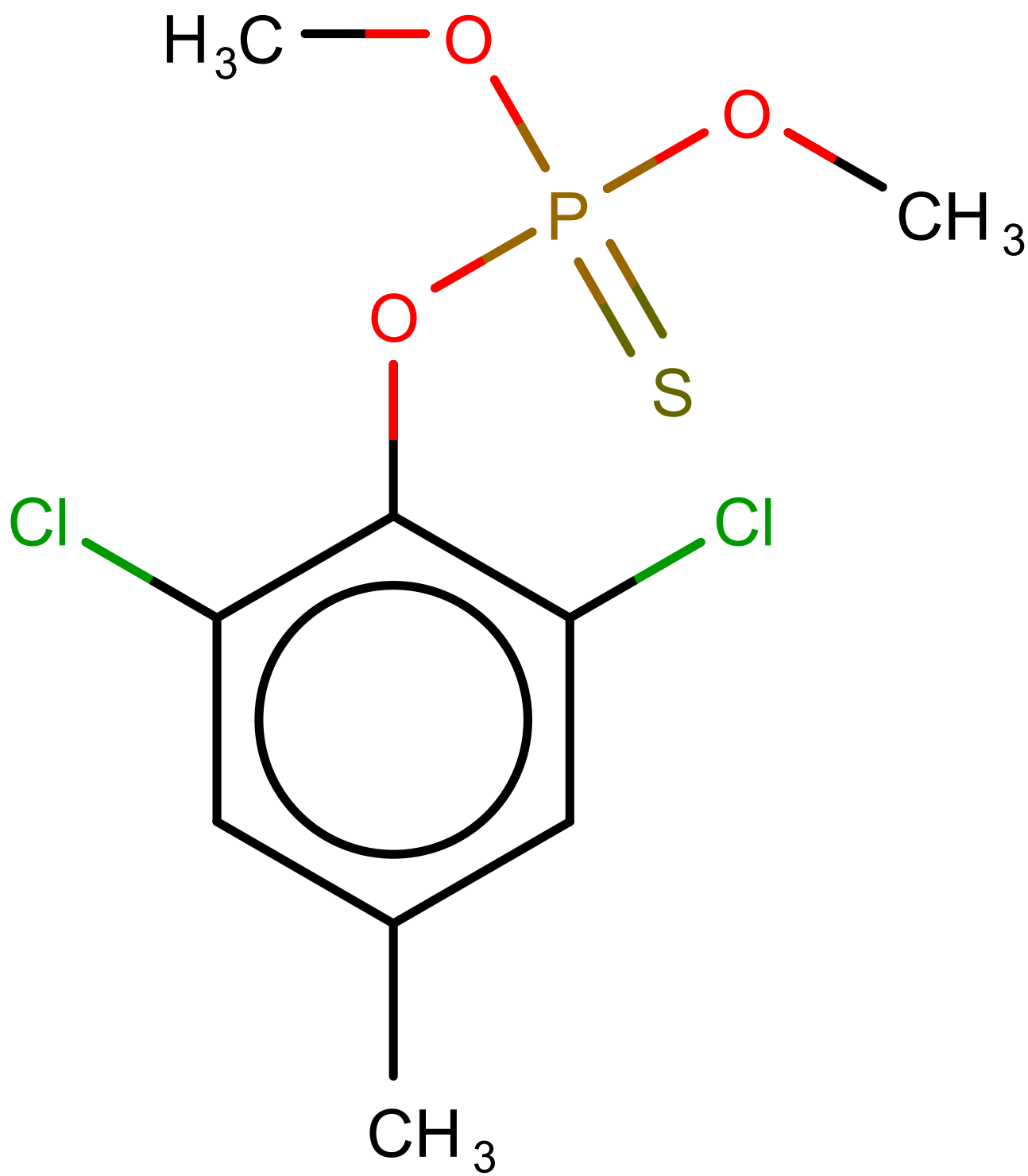

Supplement: Supplementary file 1 [file toxics-12-00425-s001.zip › Supplementary Materials/2D chemical structures/1703.pdf]

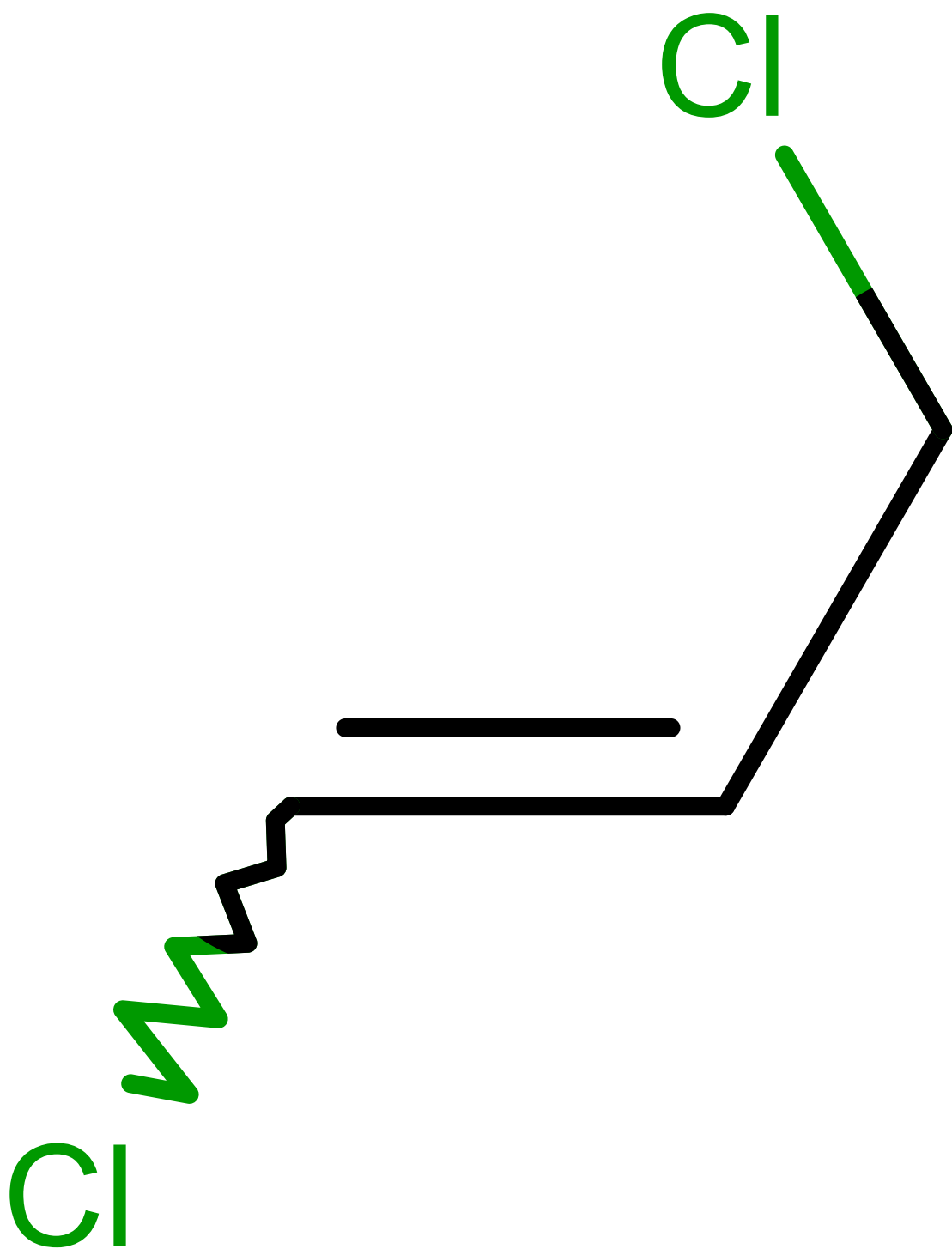

Supplement: Supplementary file 1 [file toxics-12-00425-s001.zip › Supplementary Materials/2D chemical structures/1704.pdf]

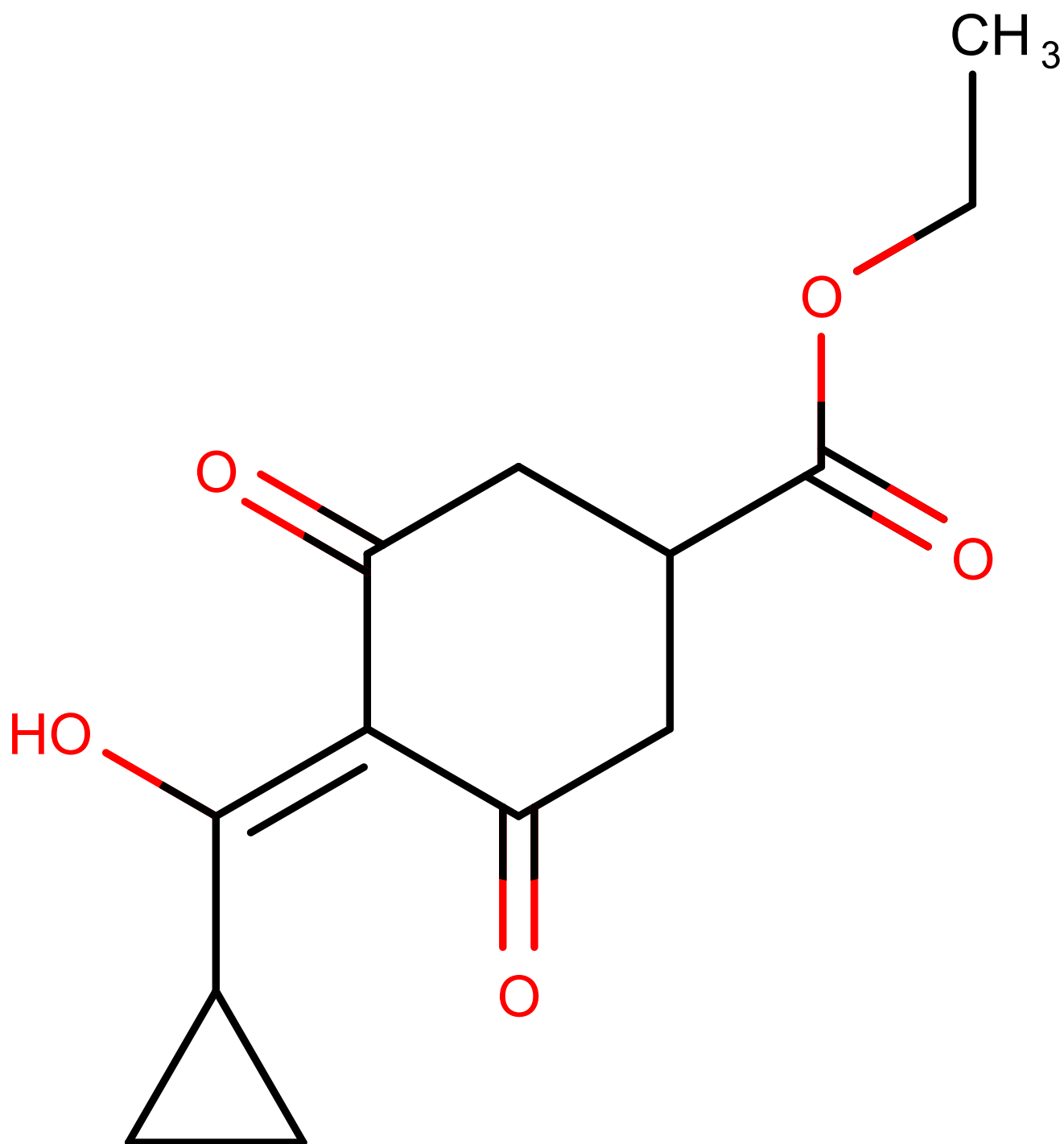

Supplement: Supplementary file 1 [file toxics-12-00425-s001.zip › Supplementary Materials/2D chemical structures/1712.pdf]

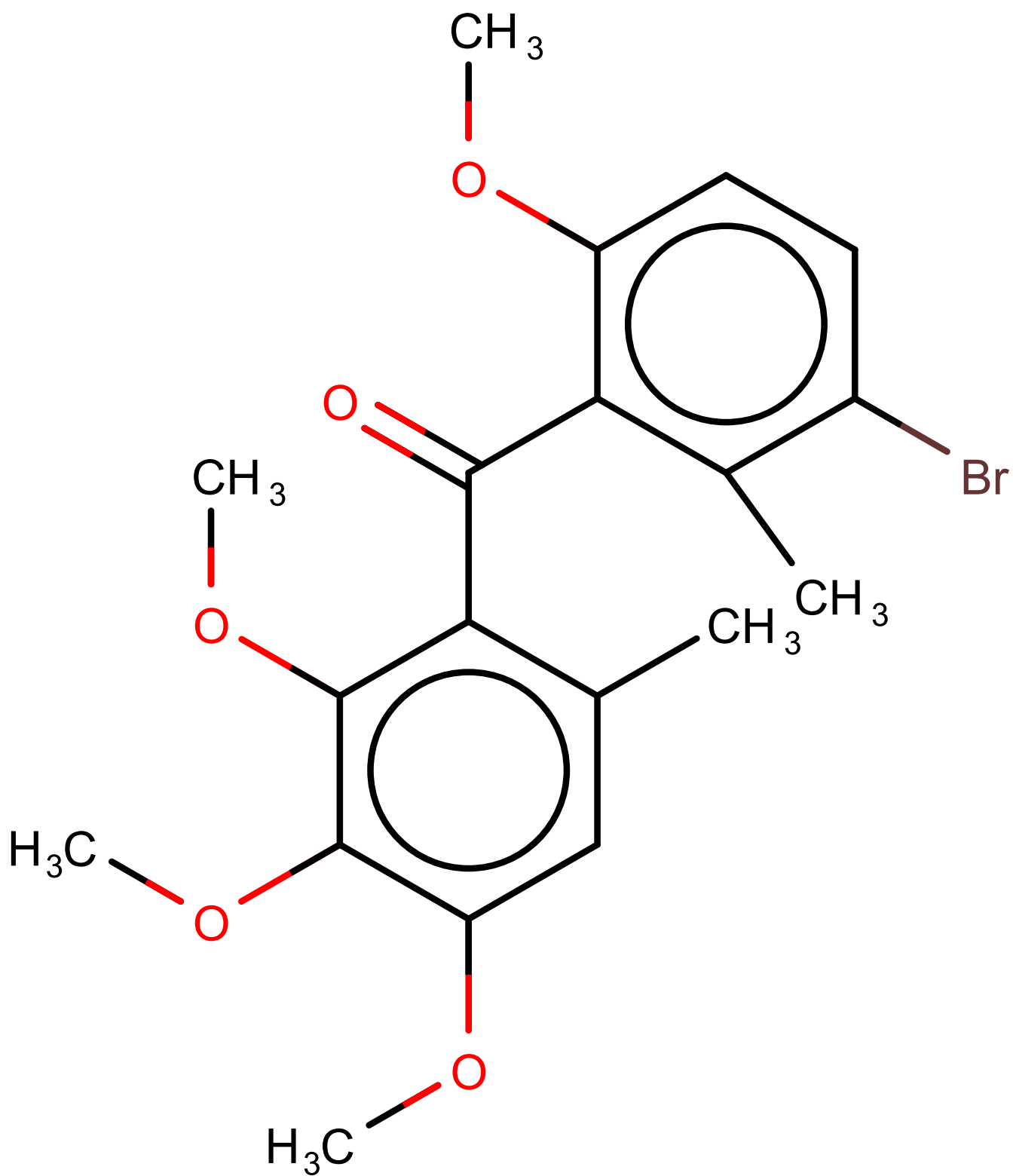

Supplement: Supplementary file 1 [file toxics-12-00425-s001.zip › Supplementary Materials/2D chemical structures/1713.pdf]

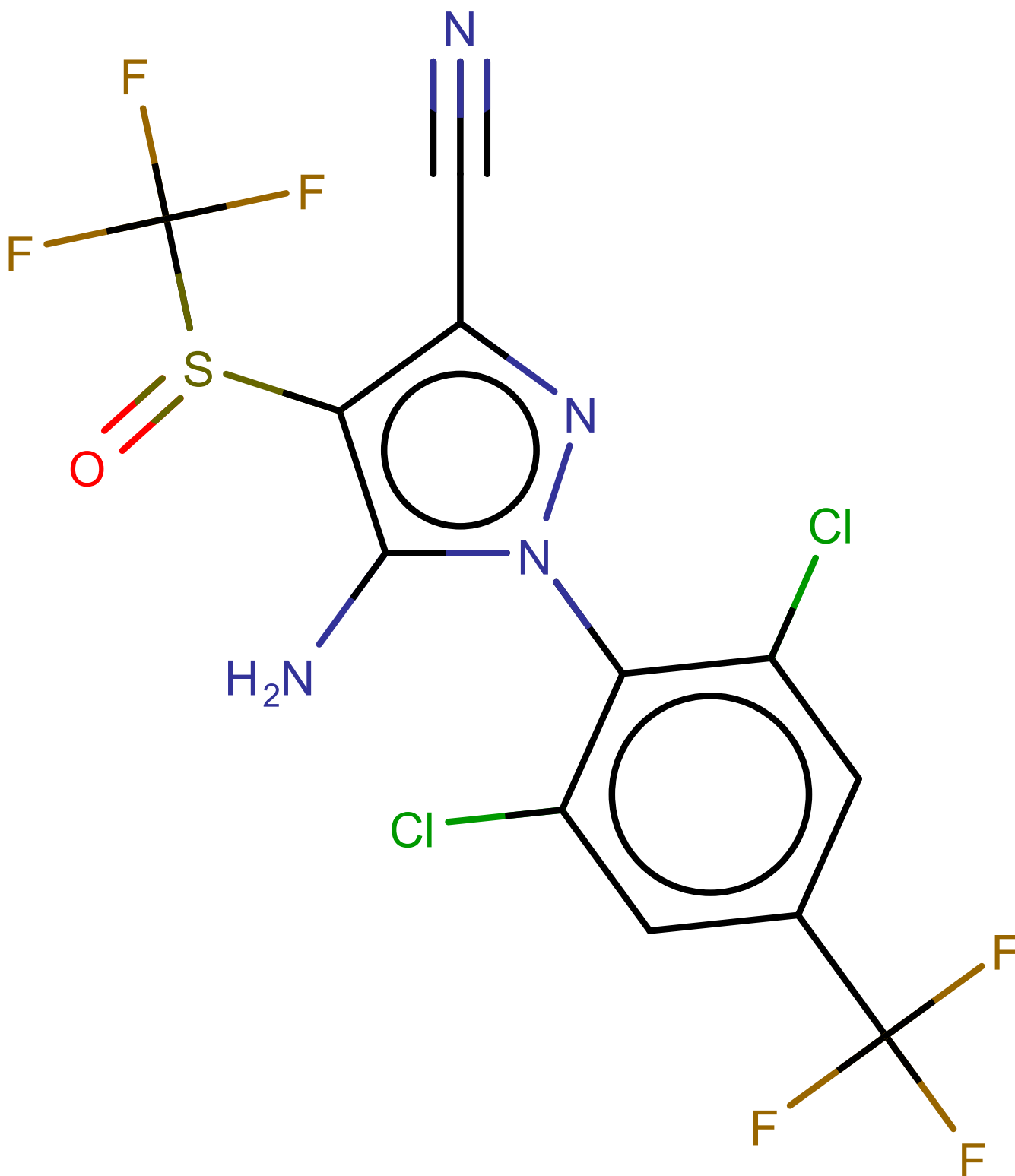

Supplement: Supplementary file 1 [file toxics-12-00425-s001.zip › Supplementary Materials/2D chemical structures/1714.pdf]

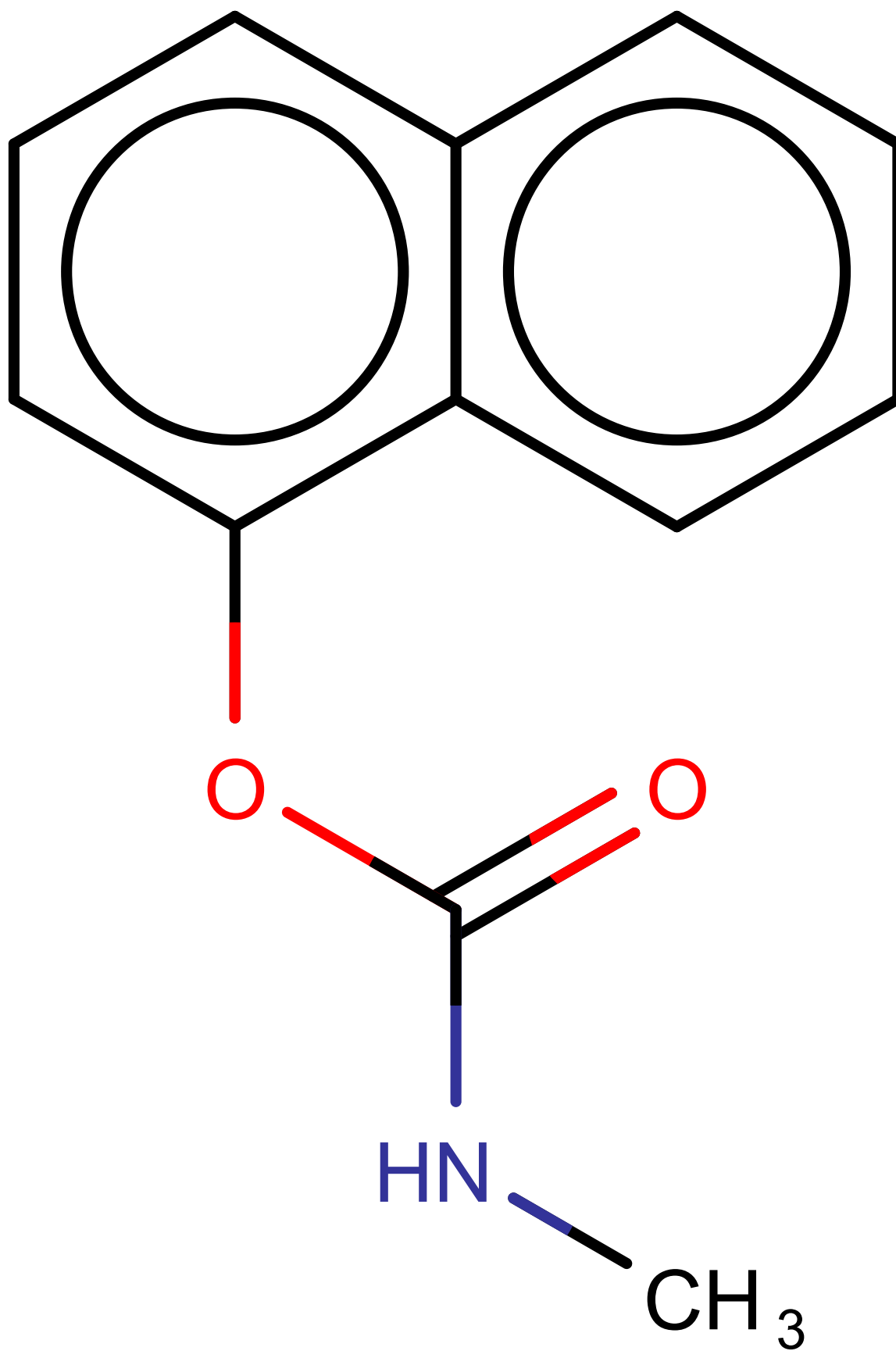

Supplement: Supplementary file 1 [file toxics-12-00425-s001.zip › Supplementary Materials/2D chemical structures/1717.pdf]

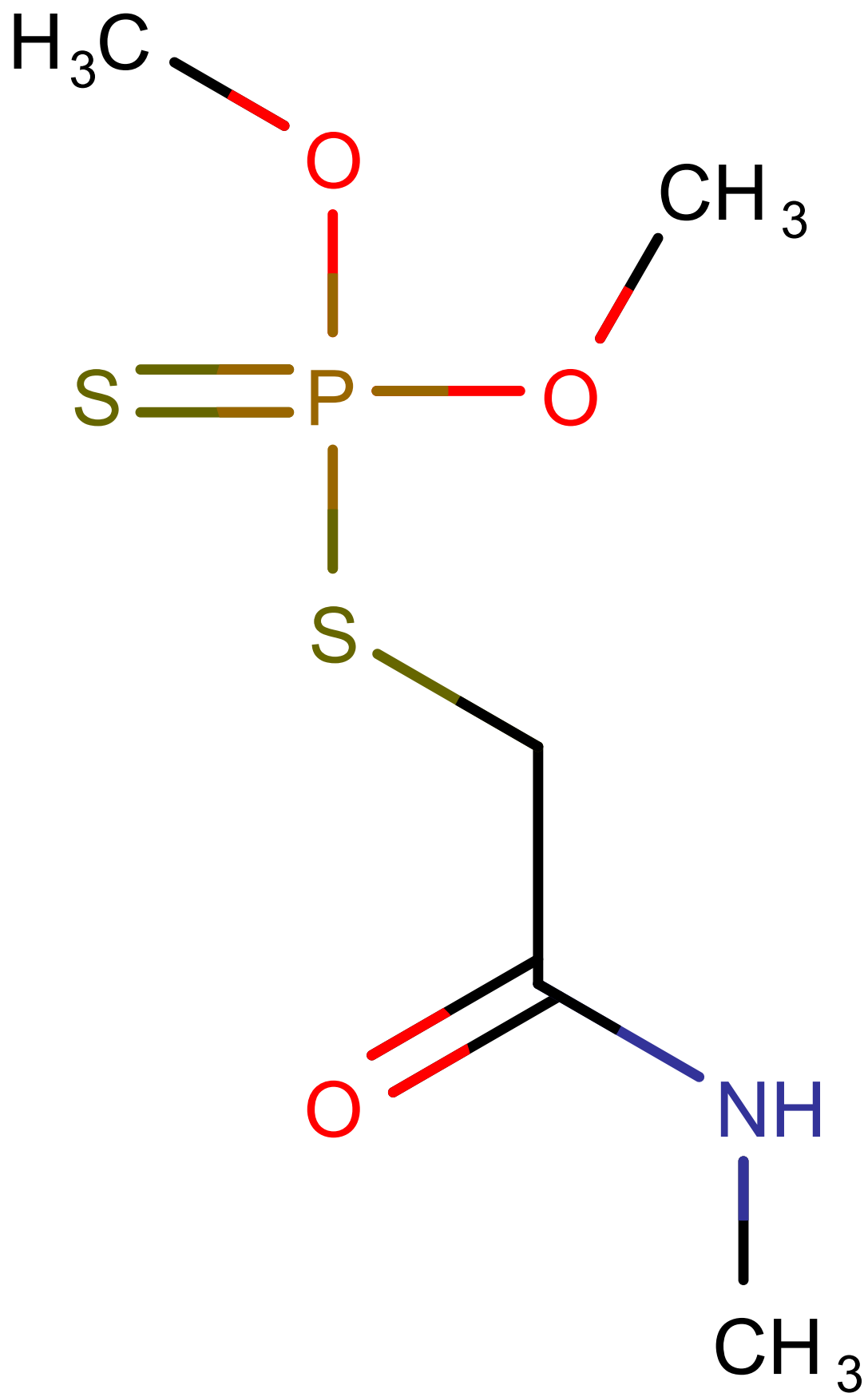

Supplement: Supplementary file 1 [file toxics-12-00425-s001.zip › Supplementary Materials/2D chemical structures/1720.pdf]

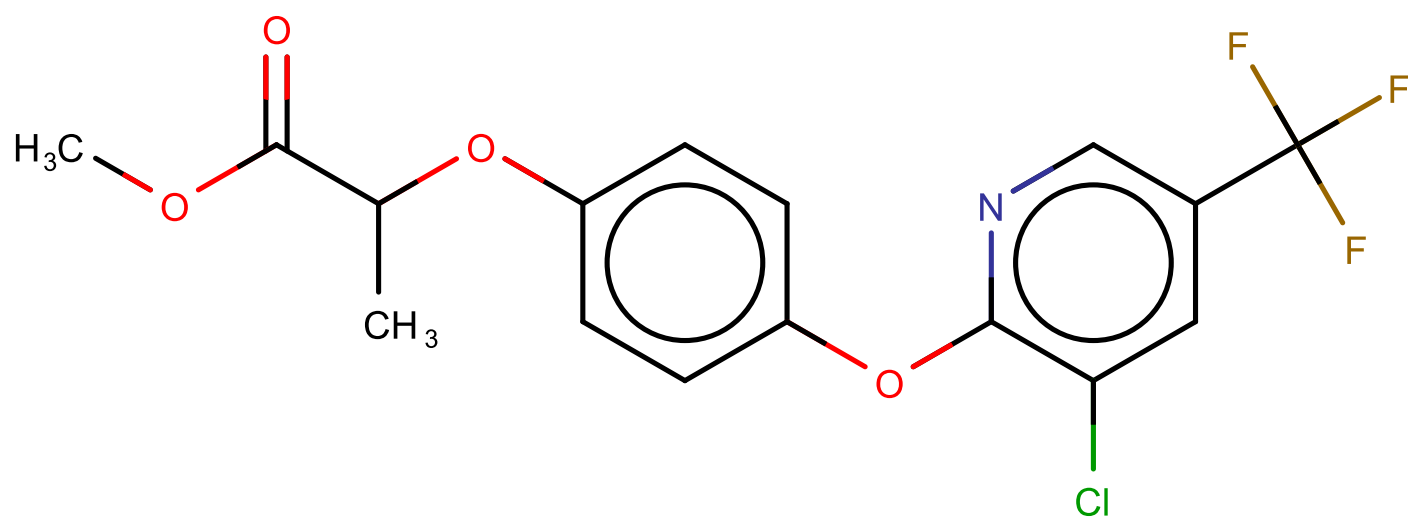

Supplement: Supplementary file 1 [file toxics-12-00425-s001.zip › Supplementary Materials/2D chemical structures/1723.pdf]

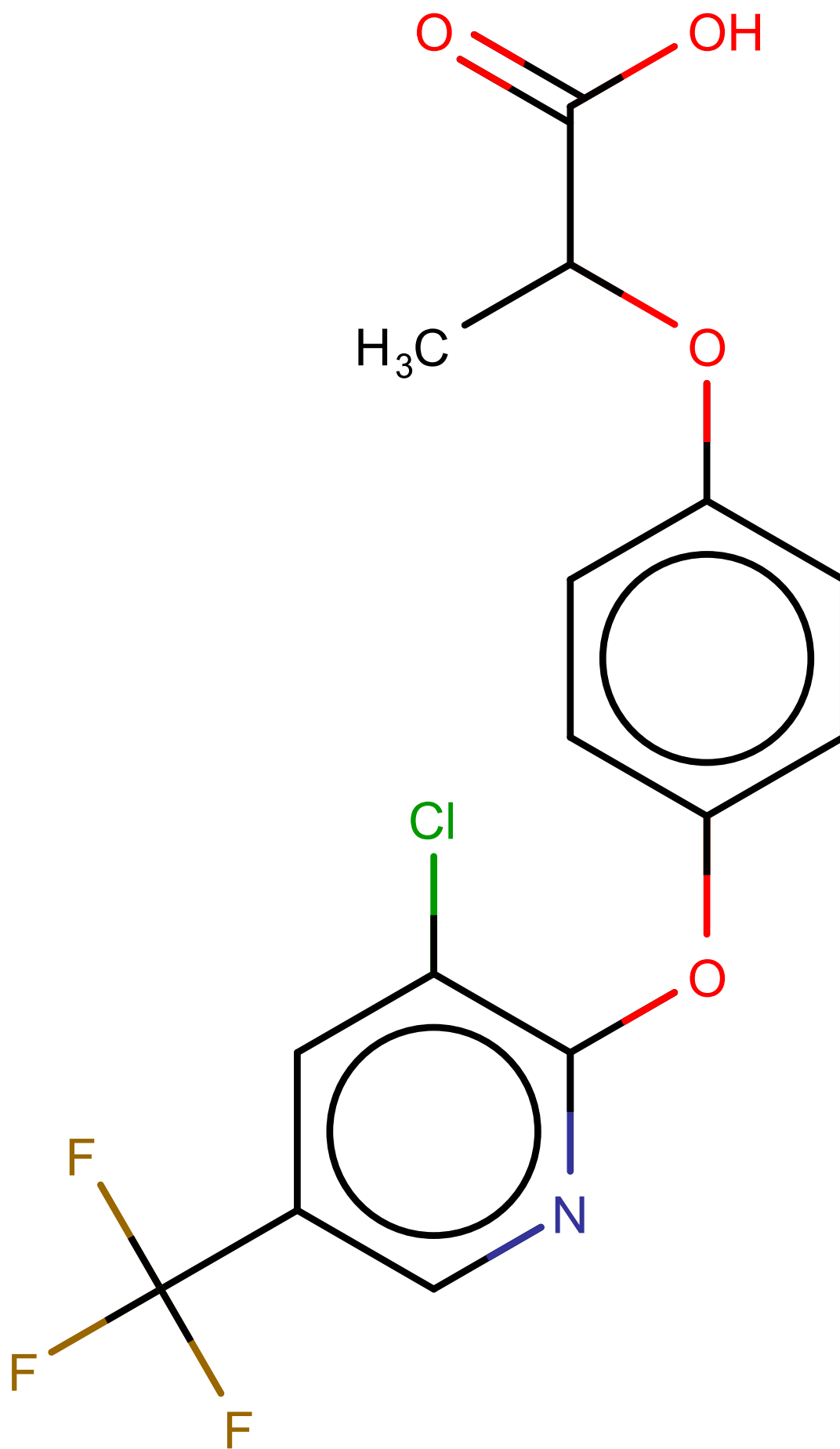

Supplement: Supplementary file 1 [file toxics-12-00425-s001.zip › Supplementary Materials/2D chemical structures/1724.pdf]

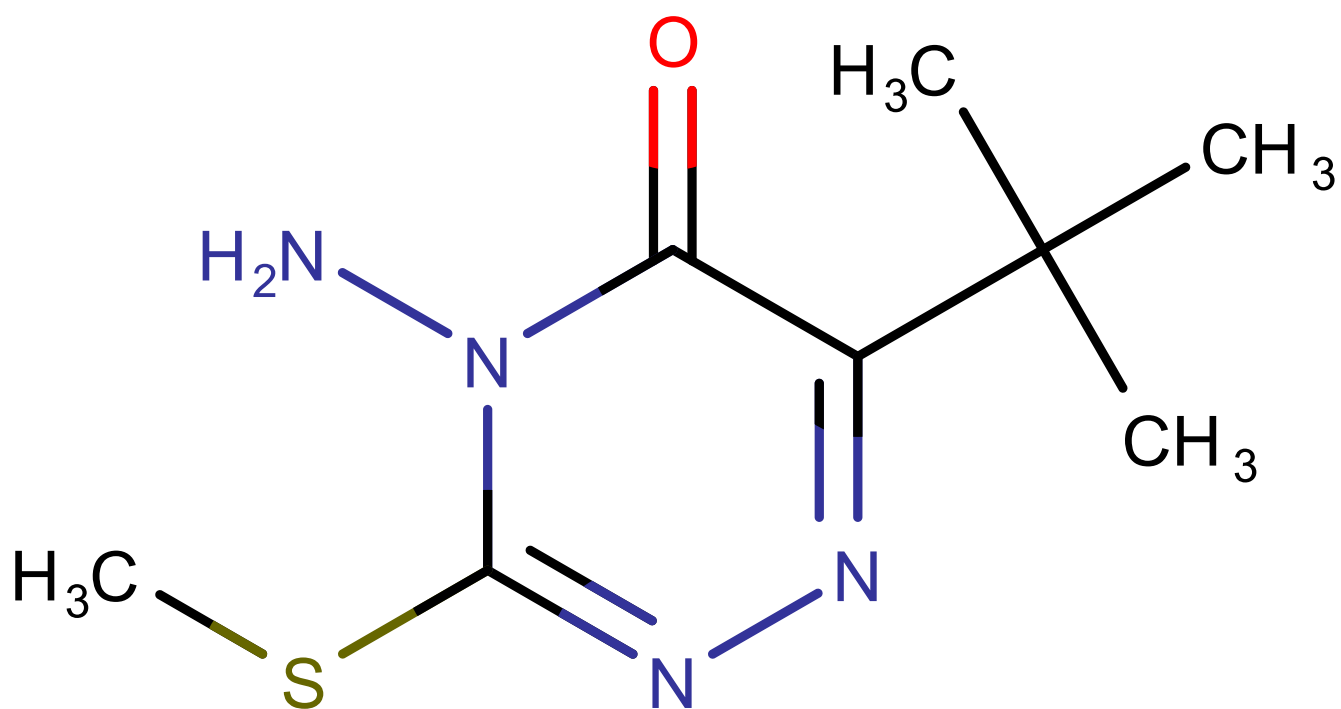

Supplement: Supplementary file 1 [file toxics-12-00425-s001.zip › Supplementary Materials/2D chemical structures/1725.pdf]

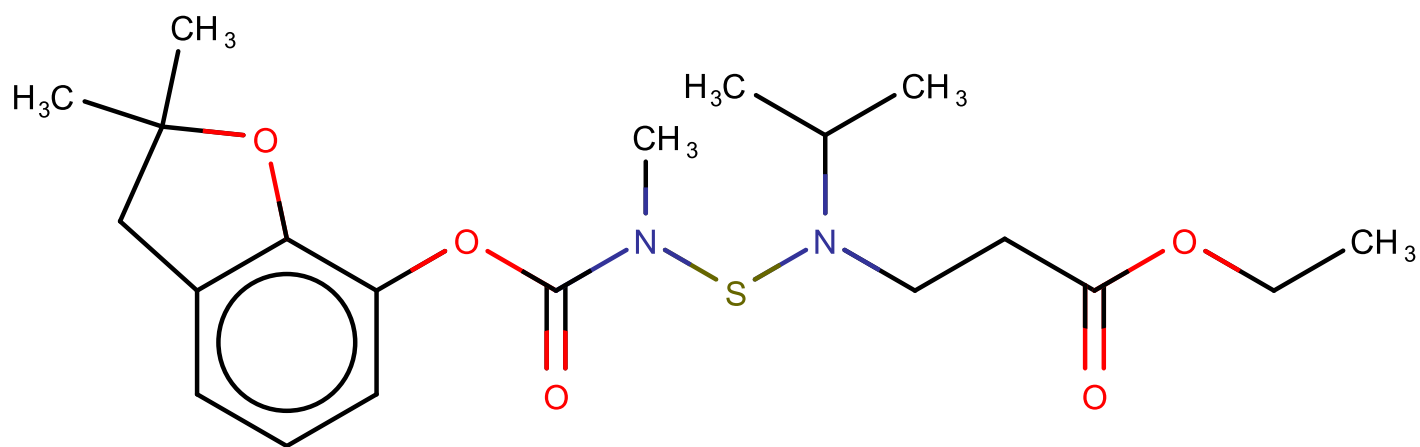

Supplement: Supplementary file 1 [file toxics-12-00425-s001.zip › Supplementary Materials/2D chemical structures/1726.pdf]

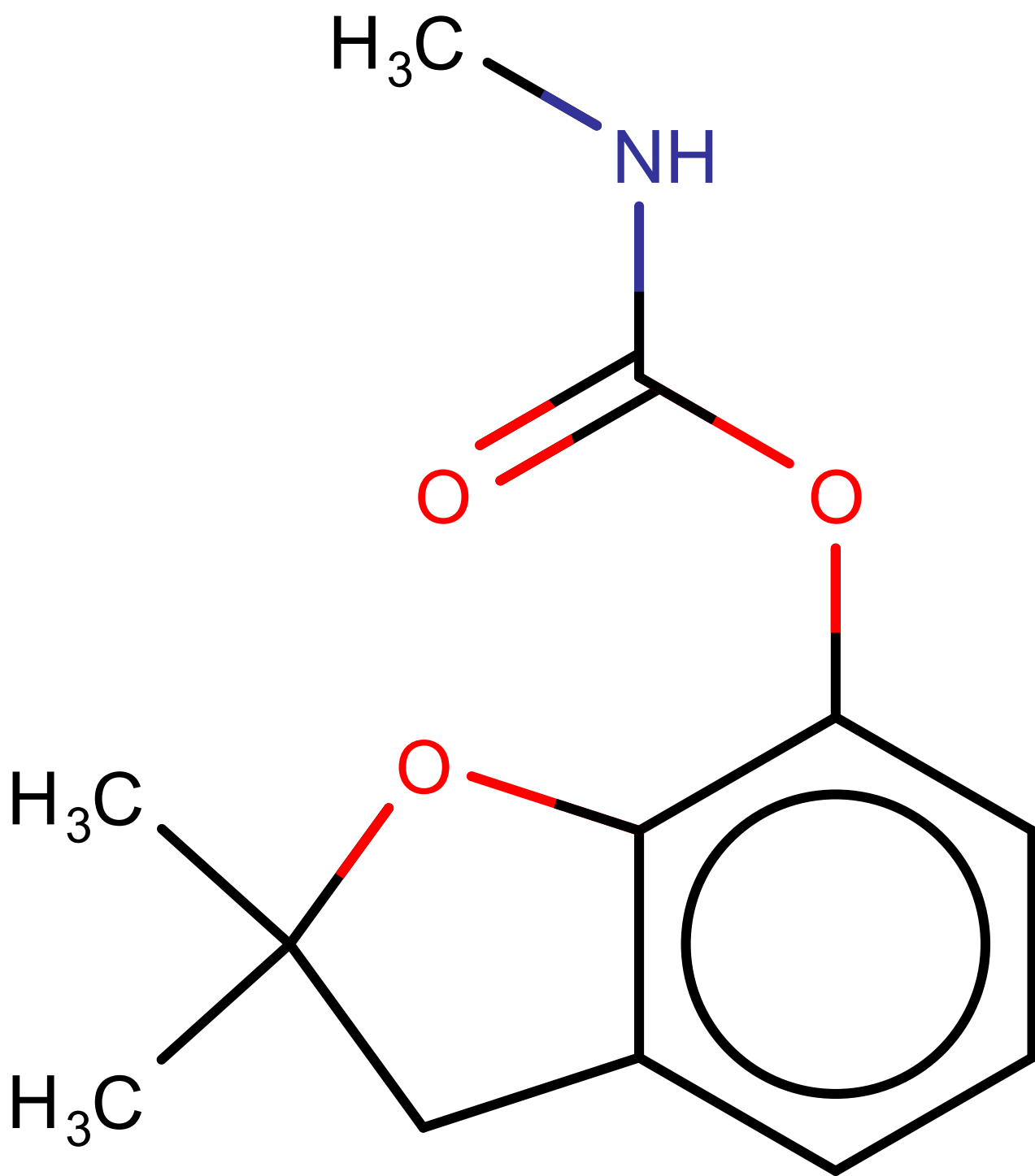

Supplement: Supplementary file 1 [file toxics-12-00425-s001.zip › Supplementary Materials/2D chemical structures/1727.pdf]

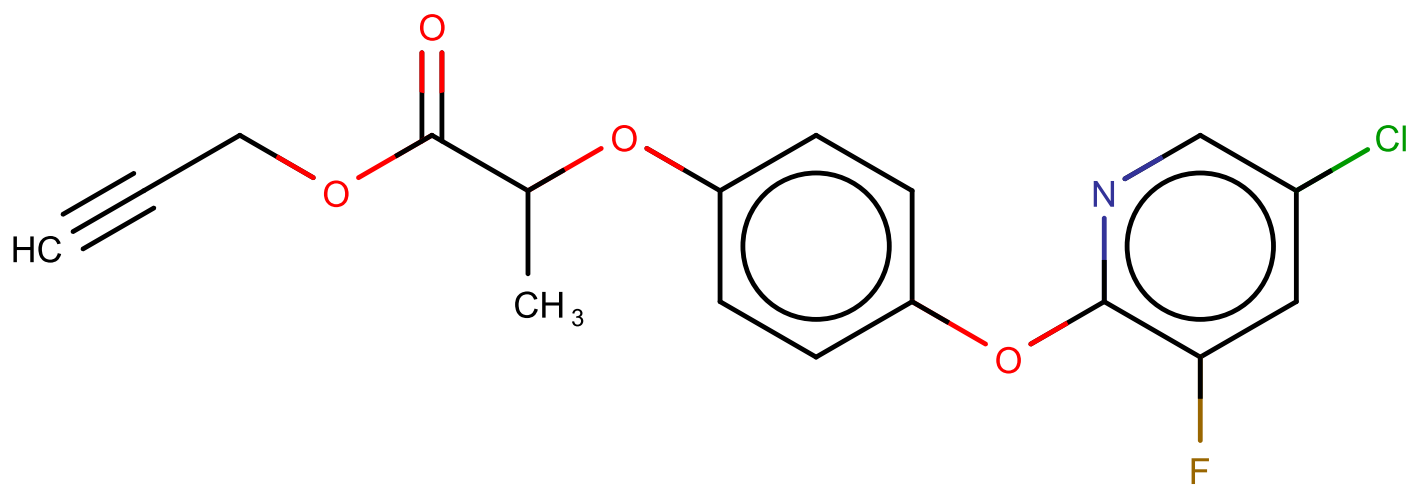

Supplement: Supplementary file 1 [file toxics-12-00425-s001.zip › Supplementary Materials/2D chemical structures/1729.pdf]

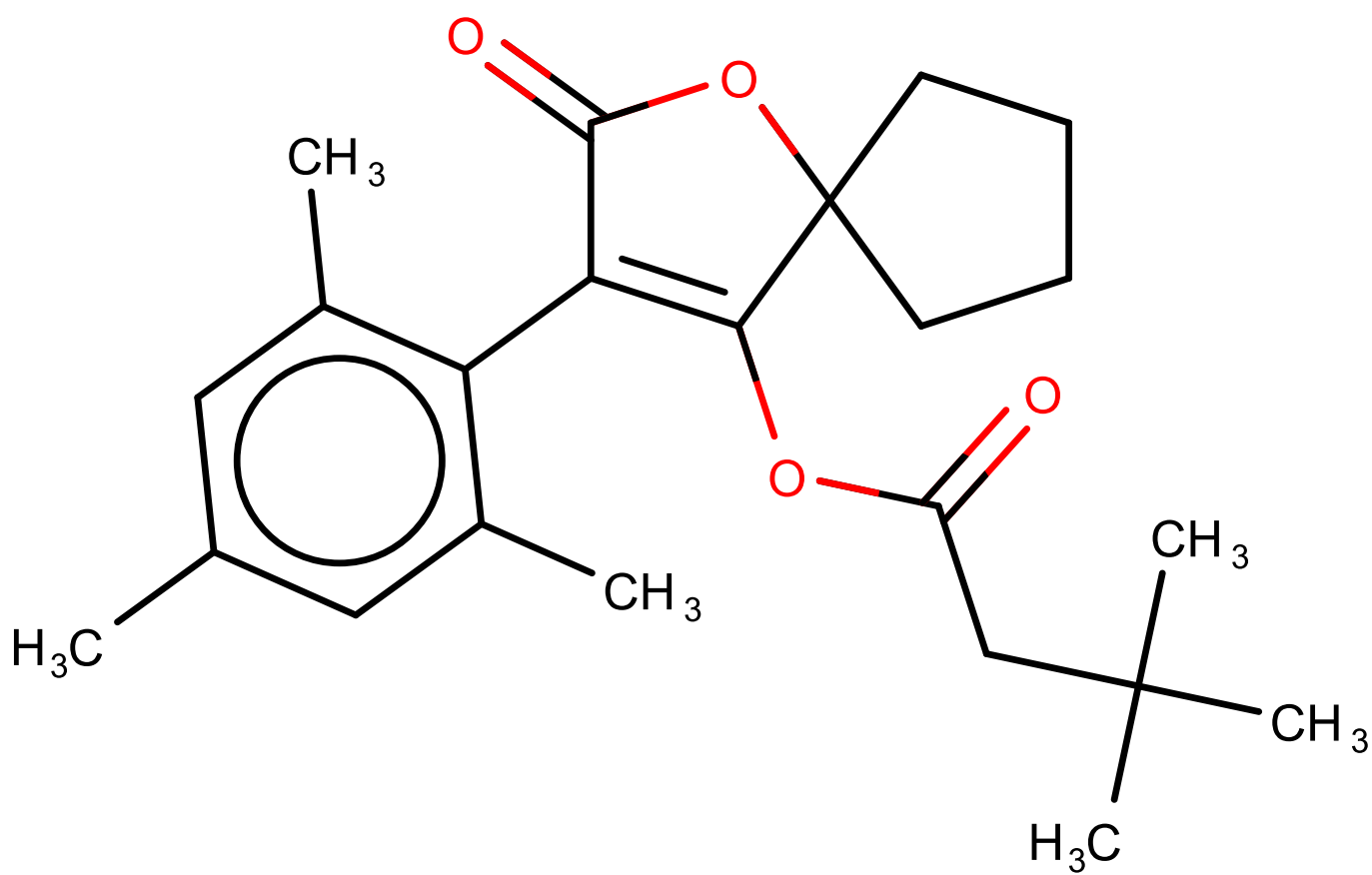

Supplement: Supplementary file 1 [file toxics-12-00425-s001.zip › Supplementary Materials/2D chemical structures/1730.pdf]

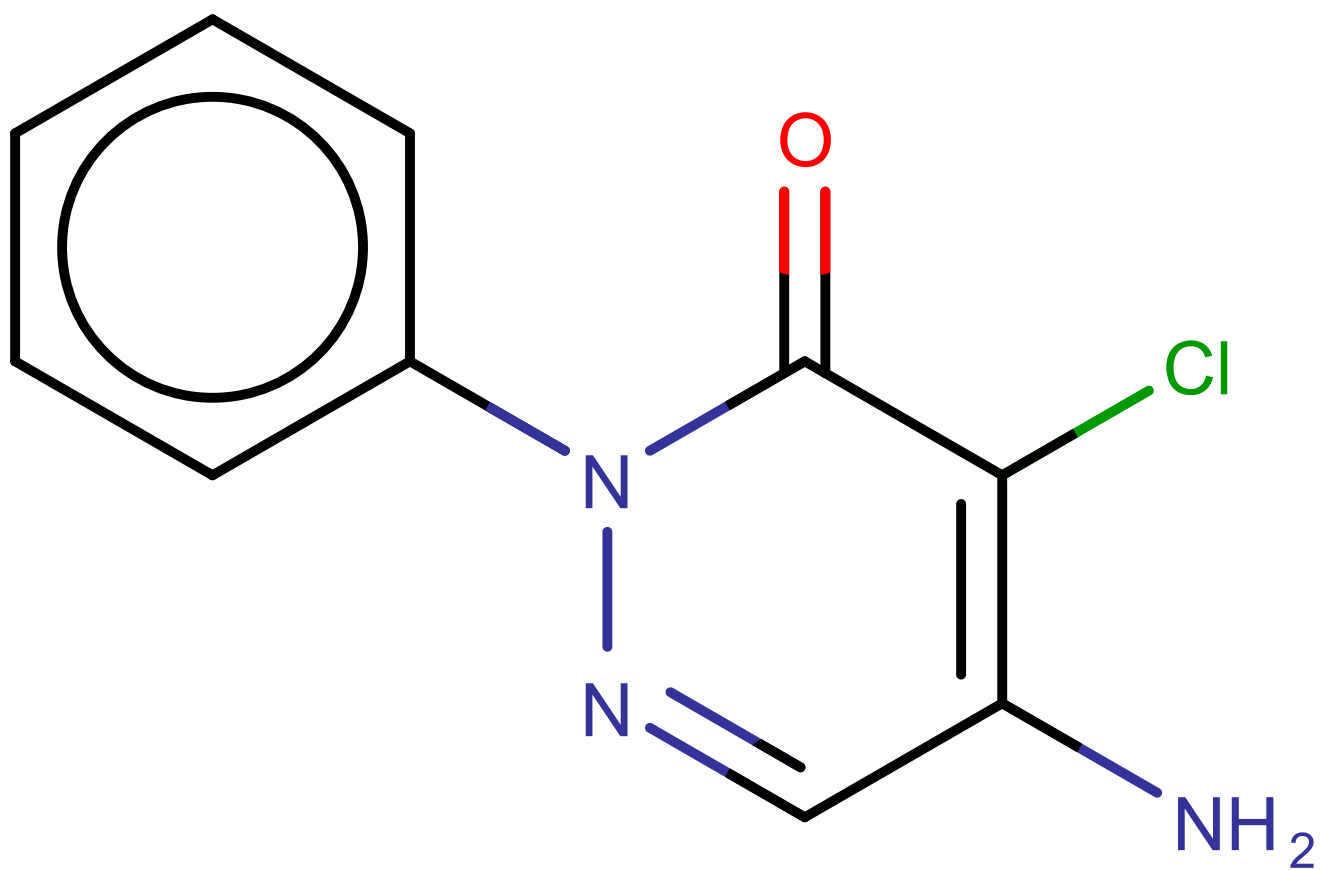

Supplement: Supplementary file 1 [file toxics-12-00425-s001.zip › Supplementary Materials/2D chemical structures/1731.pdf]

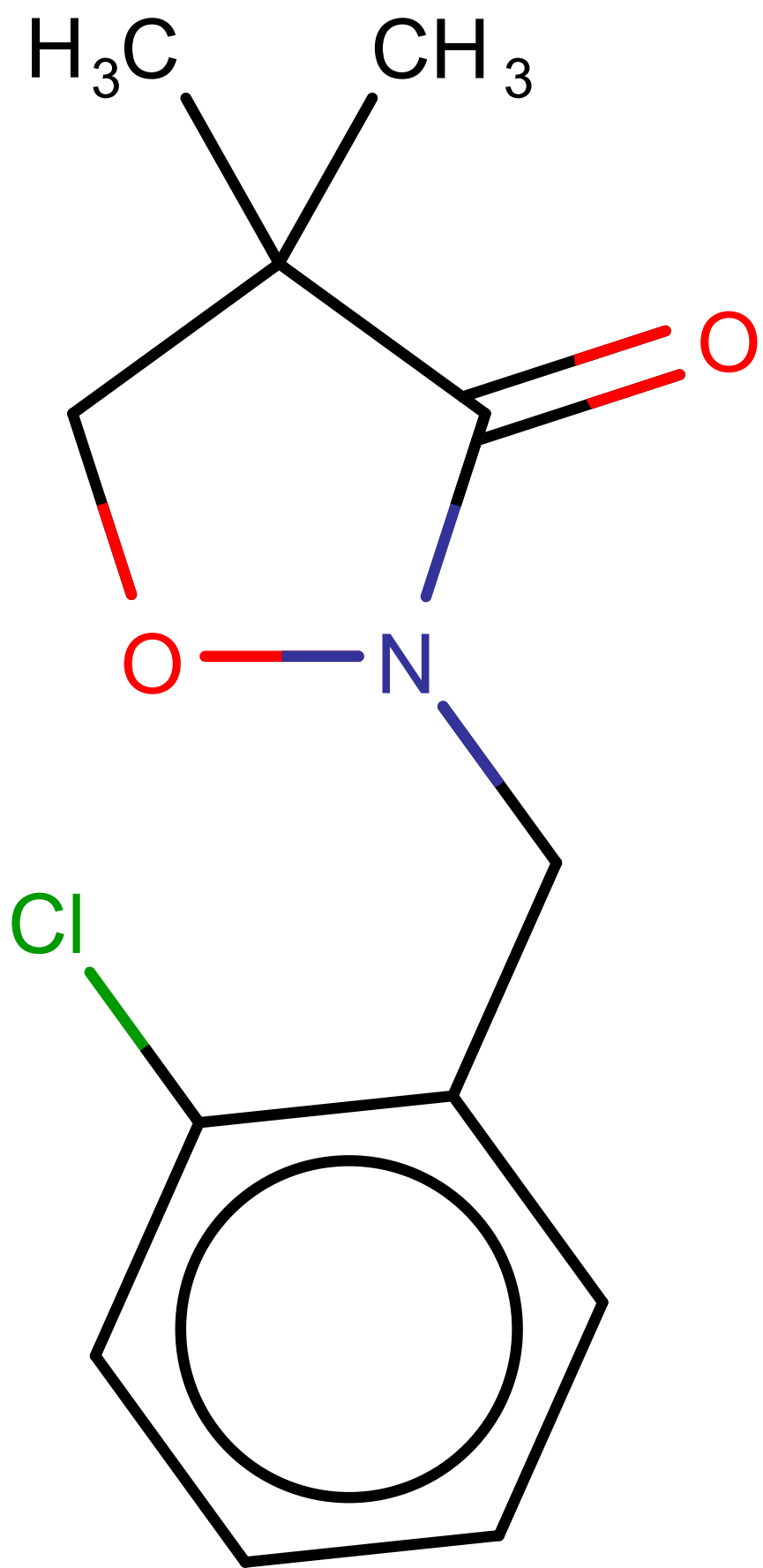

Supplement: Supplementary file 1 [file toxics-12-00425-s001.zip › Supplementary Materials/2D chemical structures/1732.pdf]

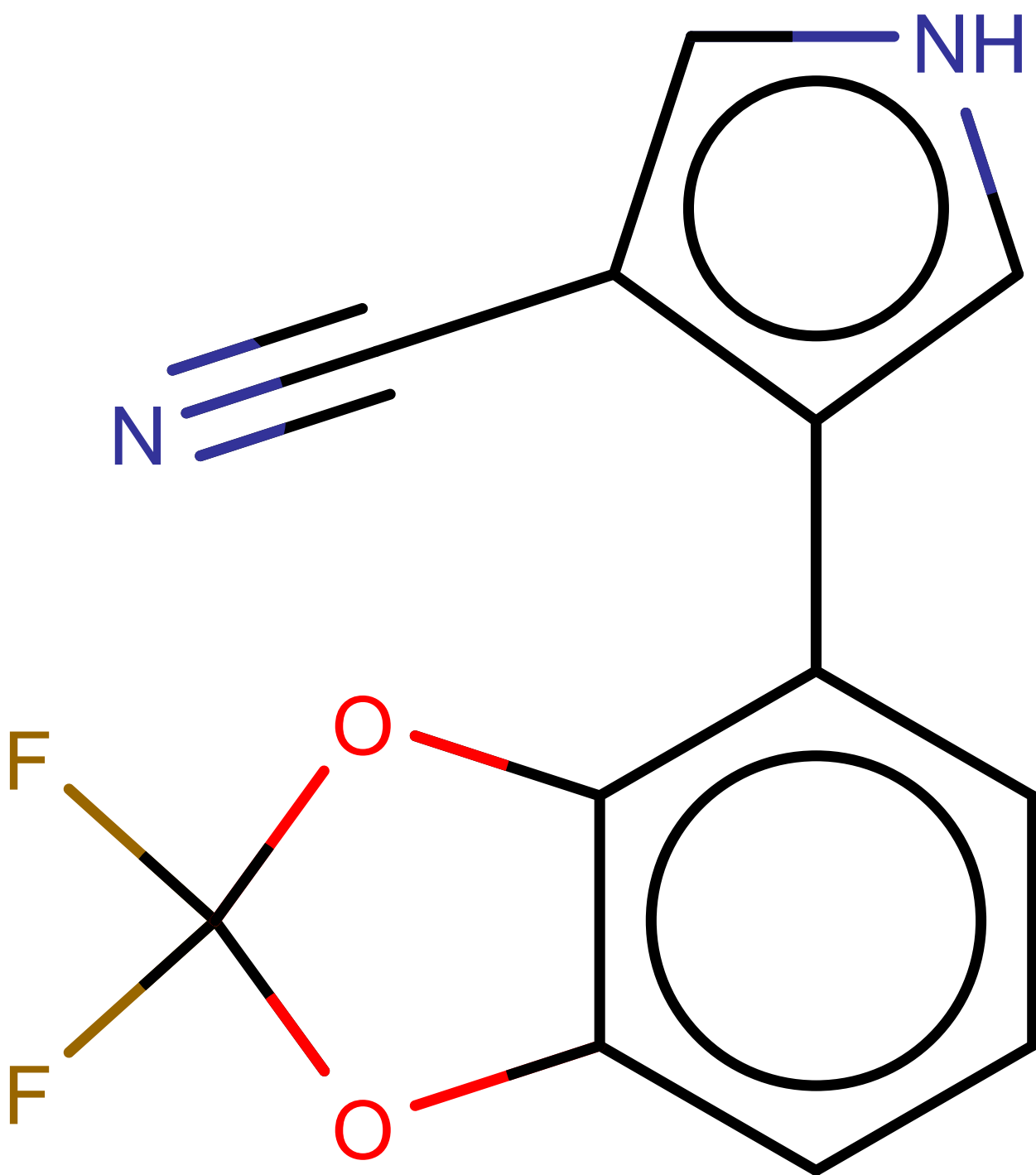

Supplement: Supplementary file 1 [file toxics-12-00425-s001.zip › Supplementary Materials/2D chemical structures/1733.pdf]

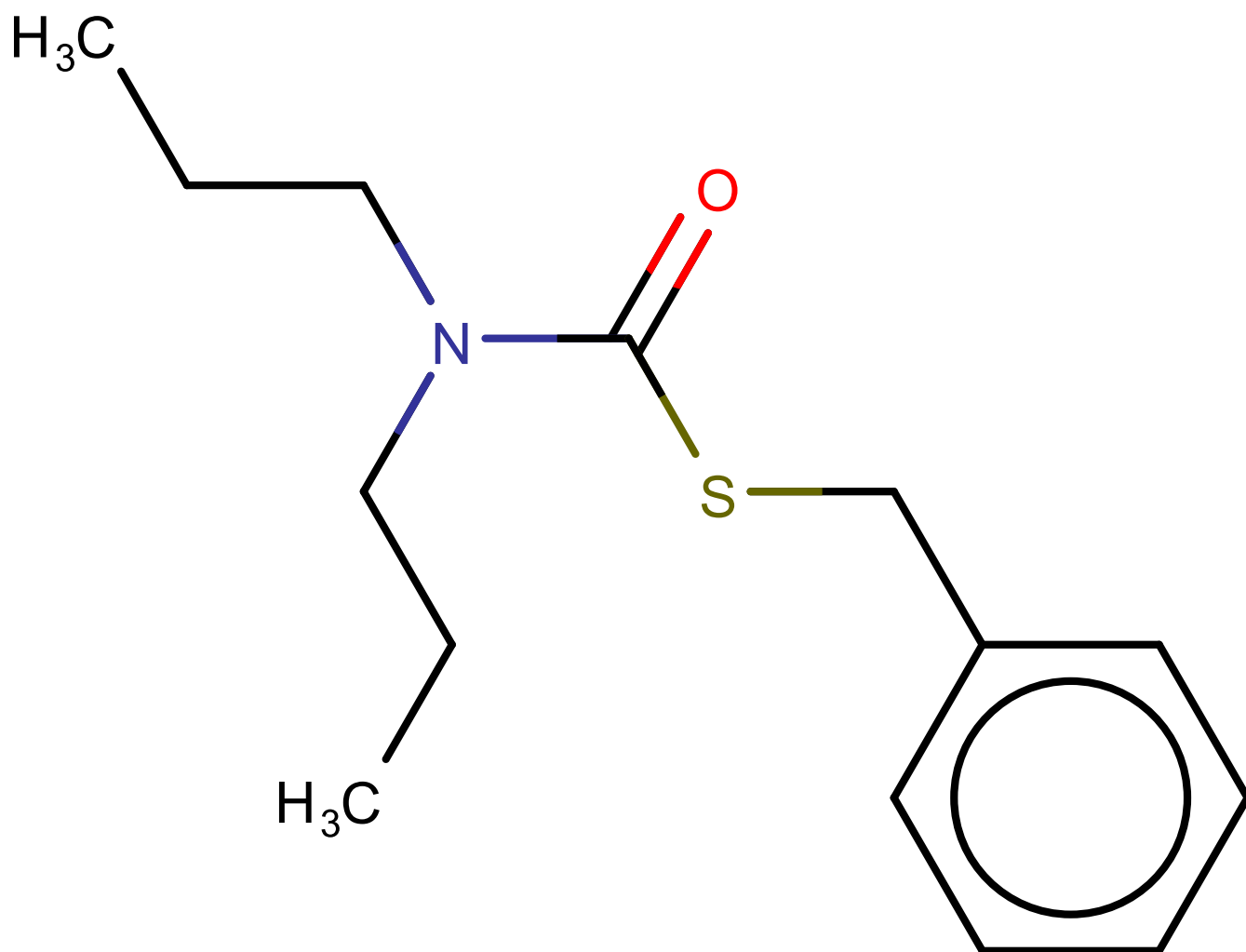

Supplement: Supplementary file 1 [file toxics-12-00425-s001.zip › Supplementary Materials/2D chemical structures/1734.pdf]

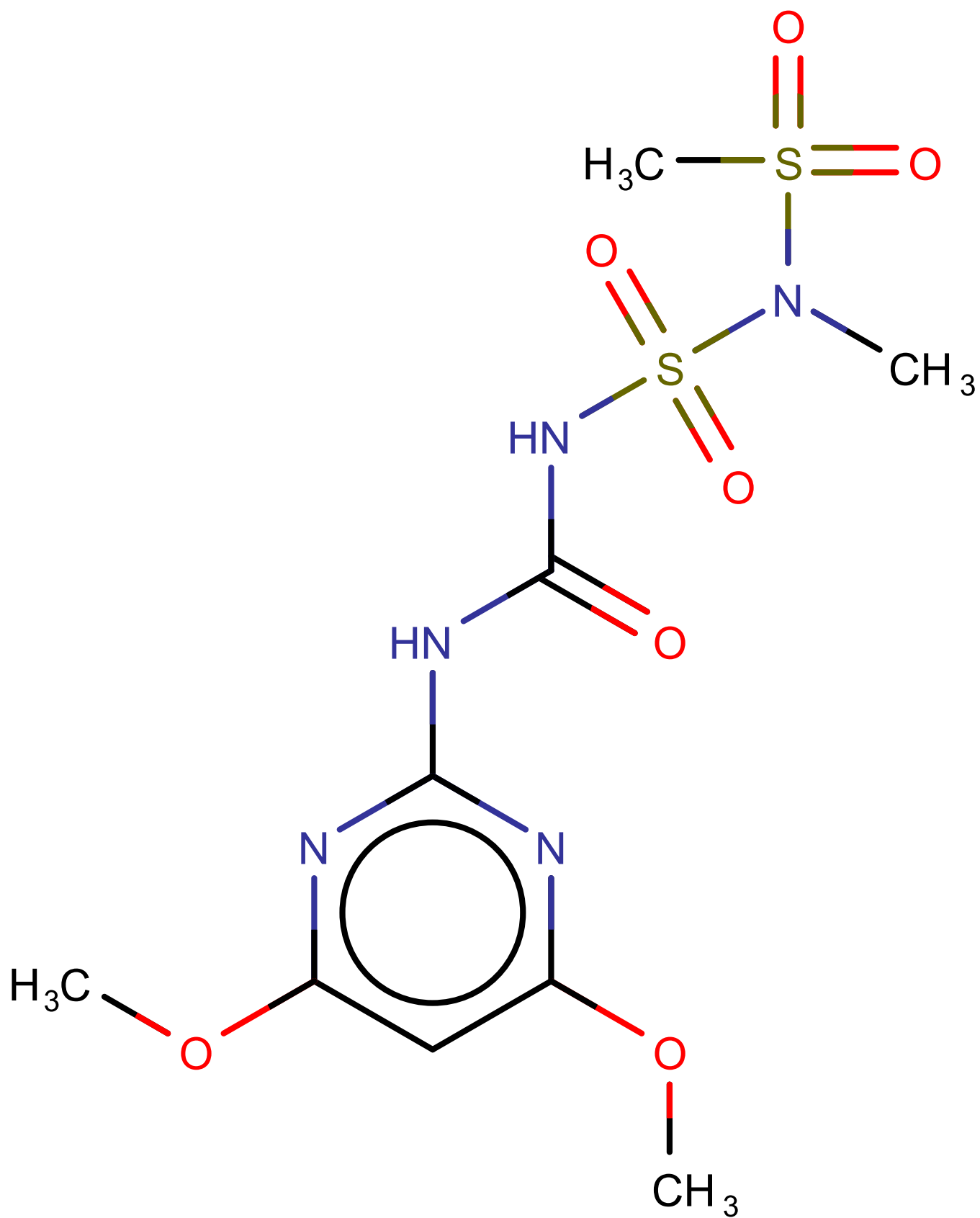

Supplement: Supplementary file 1 [file toxics-12-00425-s001.zip › Supplementary Materials/2D chemical structures/1735.pdf]

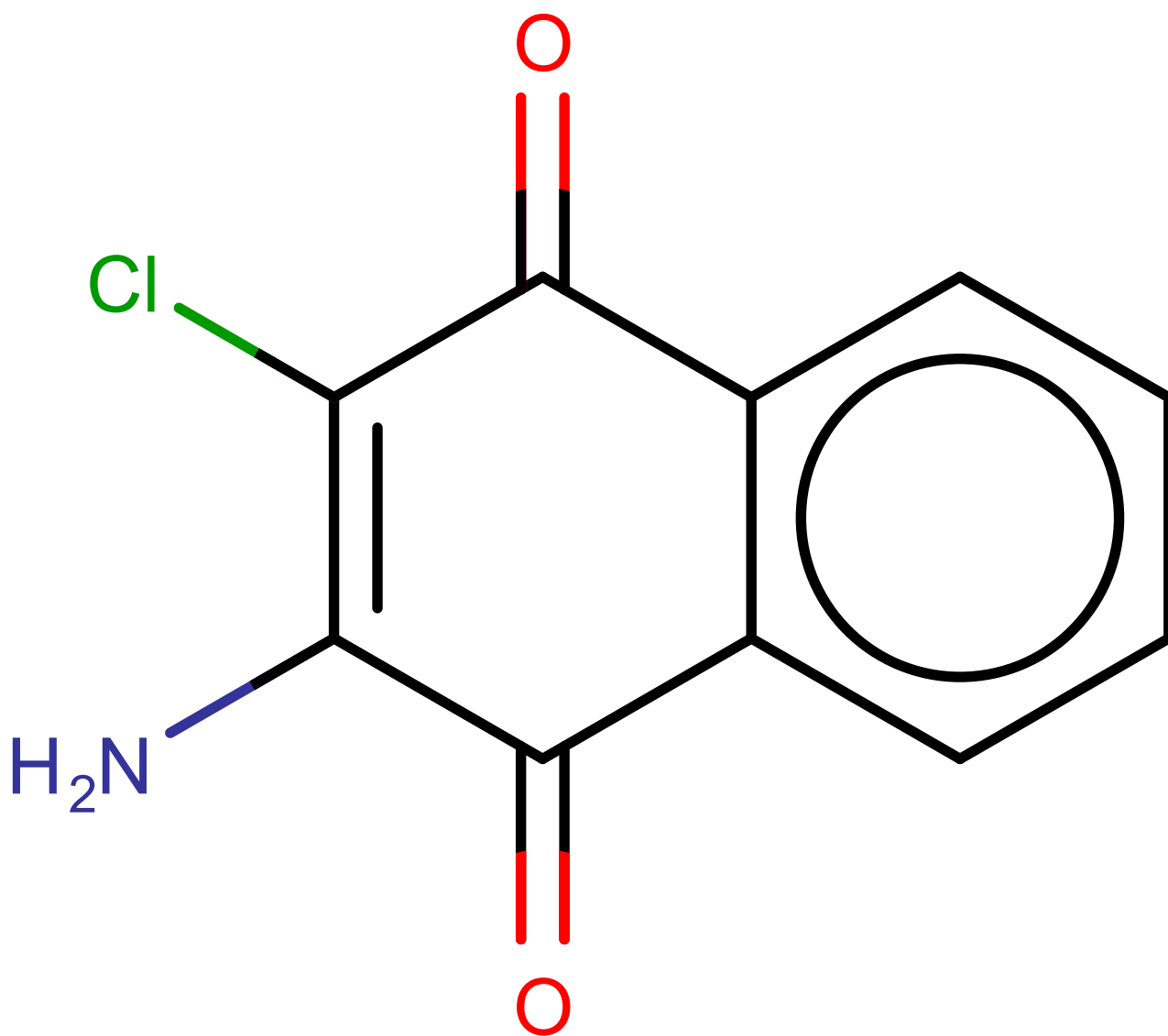

Supplement: Supplementary file 1 [file toxics-12-00425-s001.zip › Supplementary Materials/2D chemical structures/1736.pdf]

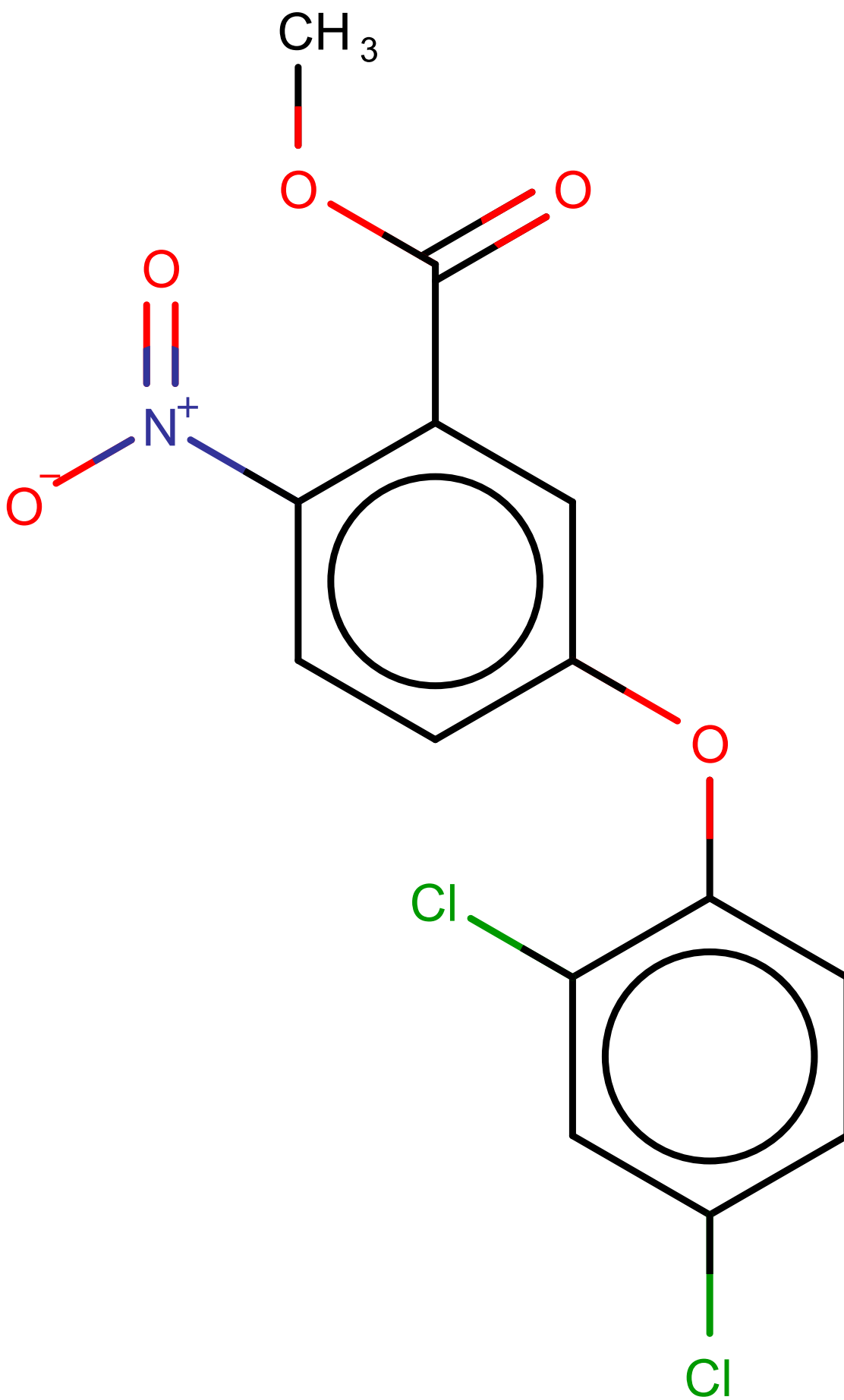

Supplement: Supplementary file 1 [file toxics-12-00425-s001.zip › Supplementary Materials/2D chemical structures/1738.pdf]

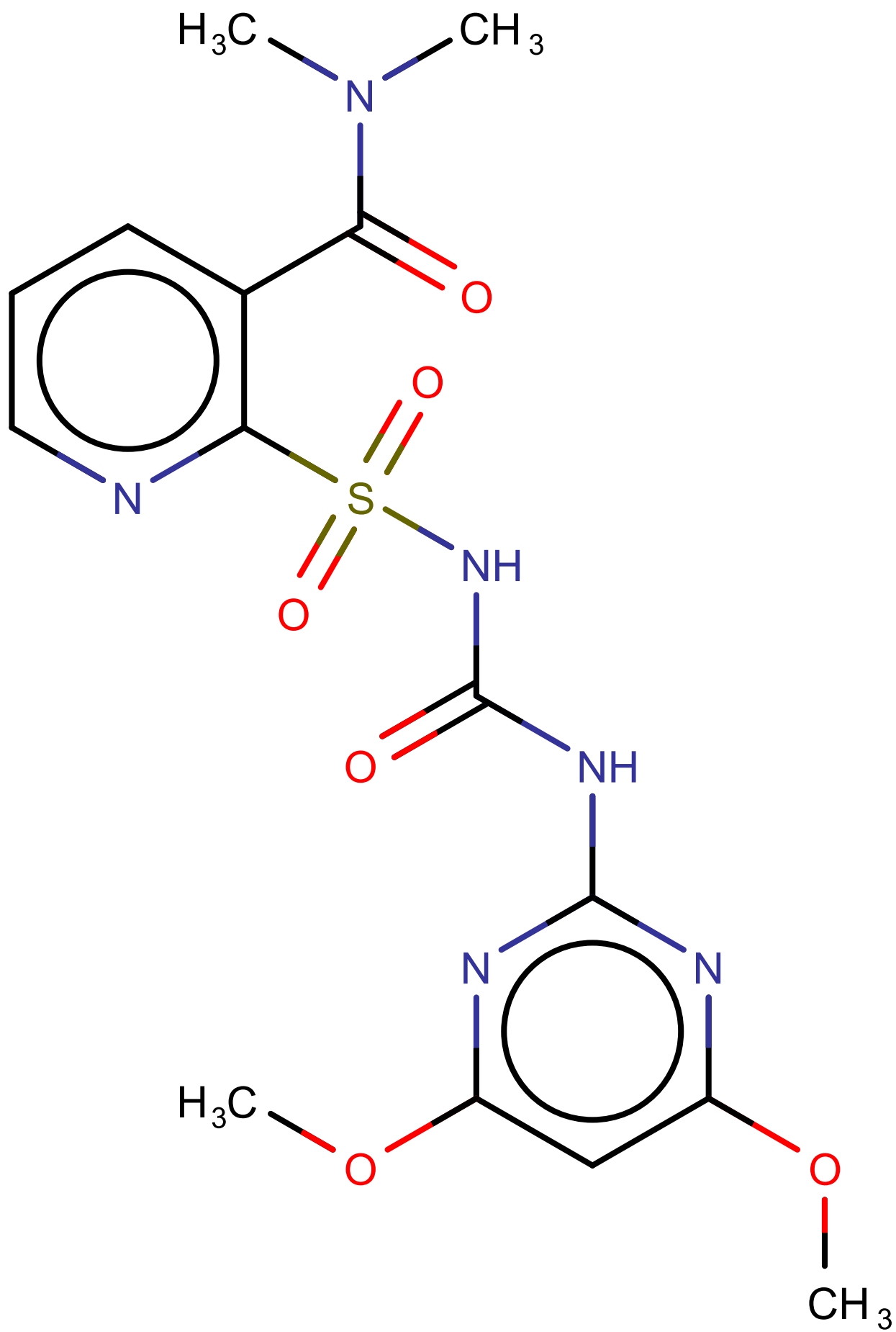

Supplement: Supplementary file 1 [file toxics-12-00425-s001.zip › Supplementary Materials/2D chemical structures/1739.pdf]

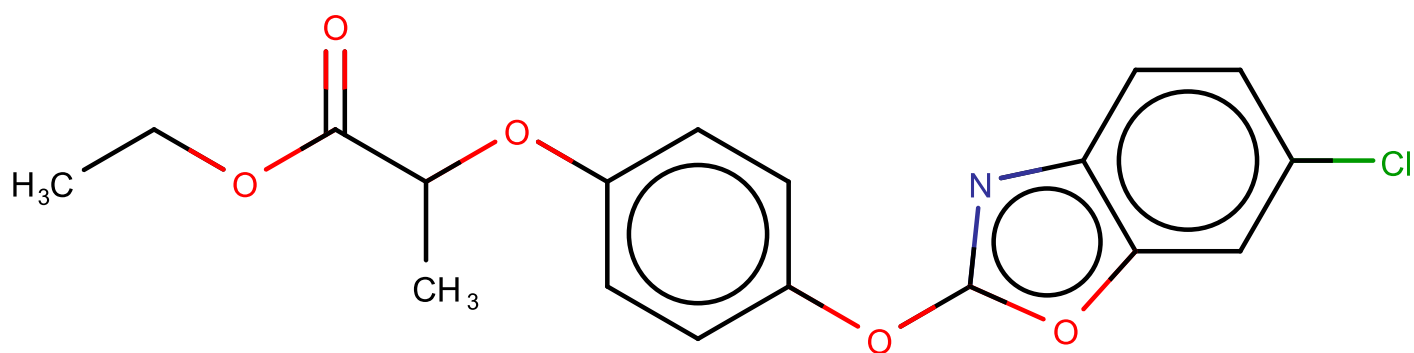

Supplement: Supplementary file 1 [file toxics-12-00425-s001.zip › Supplementary Materials/2D chemical structures/1740.pdf]

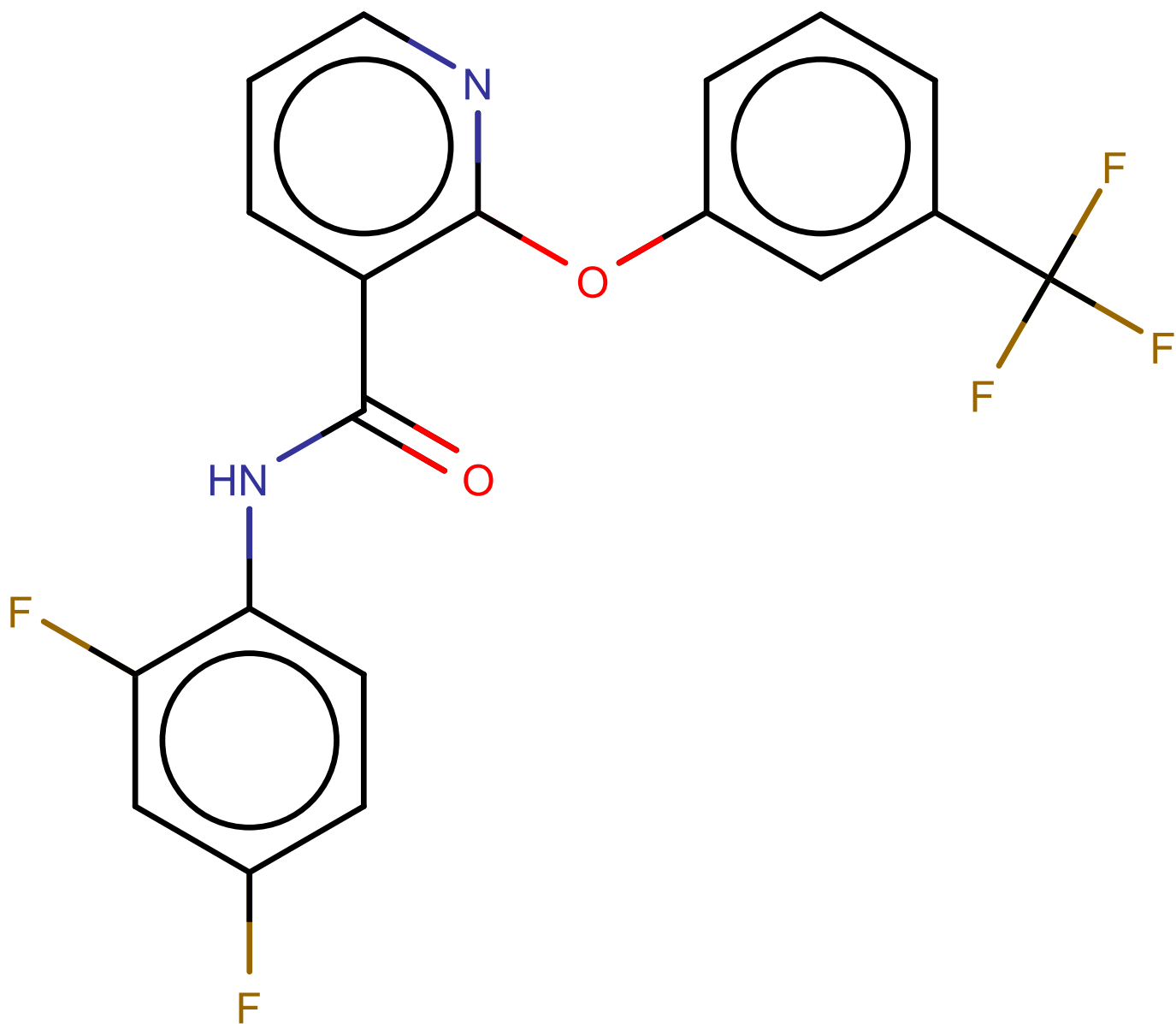

Supplement: Supplementary file 1 [file toxics-12-00425-s001.zip › Supplementary Materials/2D chemical structures/1742.pdf]

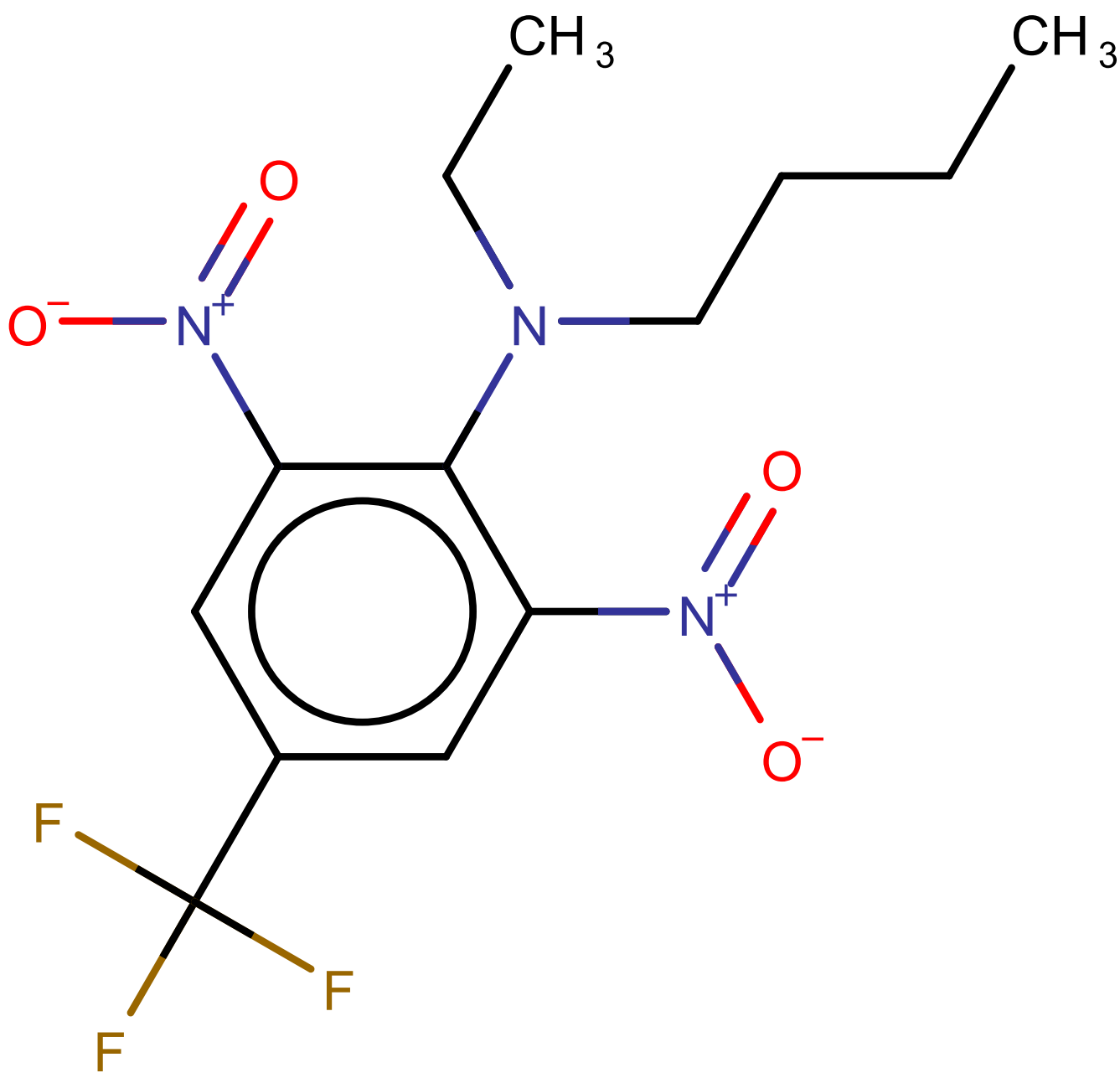

Supplement: Supplementary file 1 [file toxics-12-00425-s001.zip › Supplementary Materials/2D chemical structures/1746.pdf]

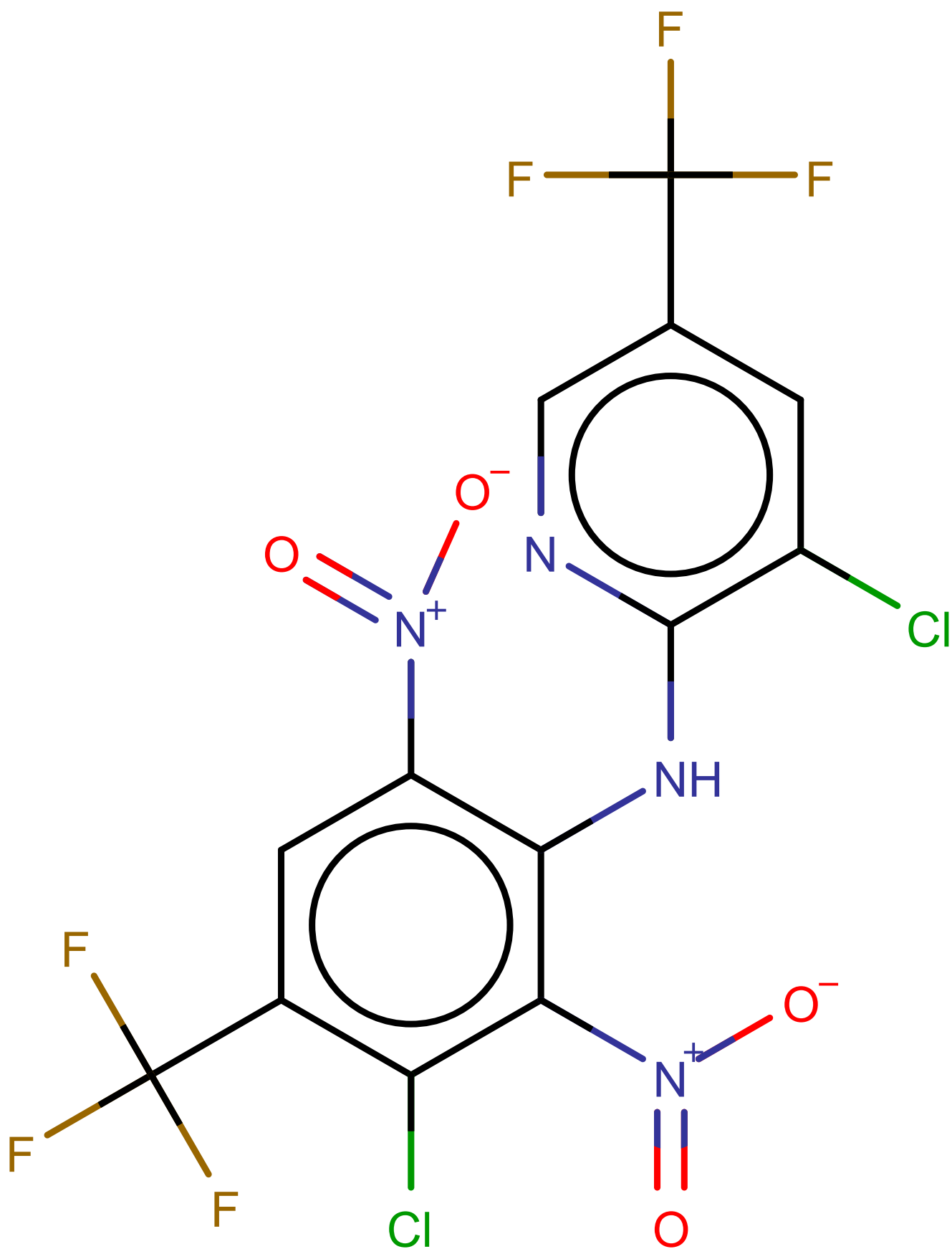

Supplement: Supplementary file 1 [file toxics-12-00425-s001.zip › Supplementary Materials/2D chemical structures/1749.pdf]

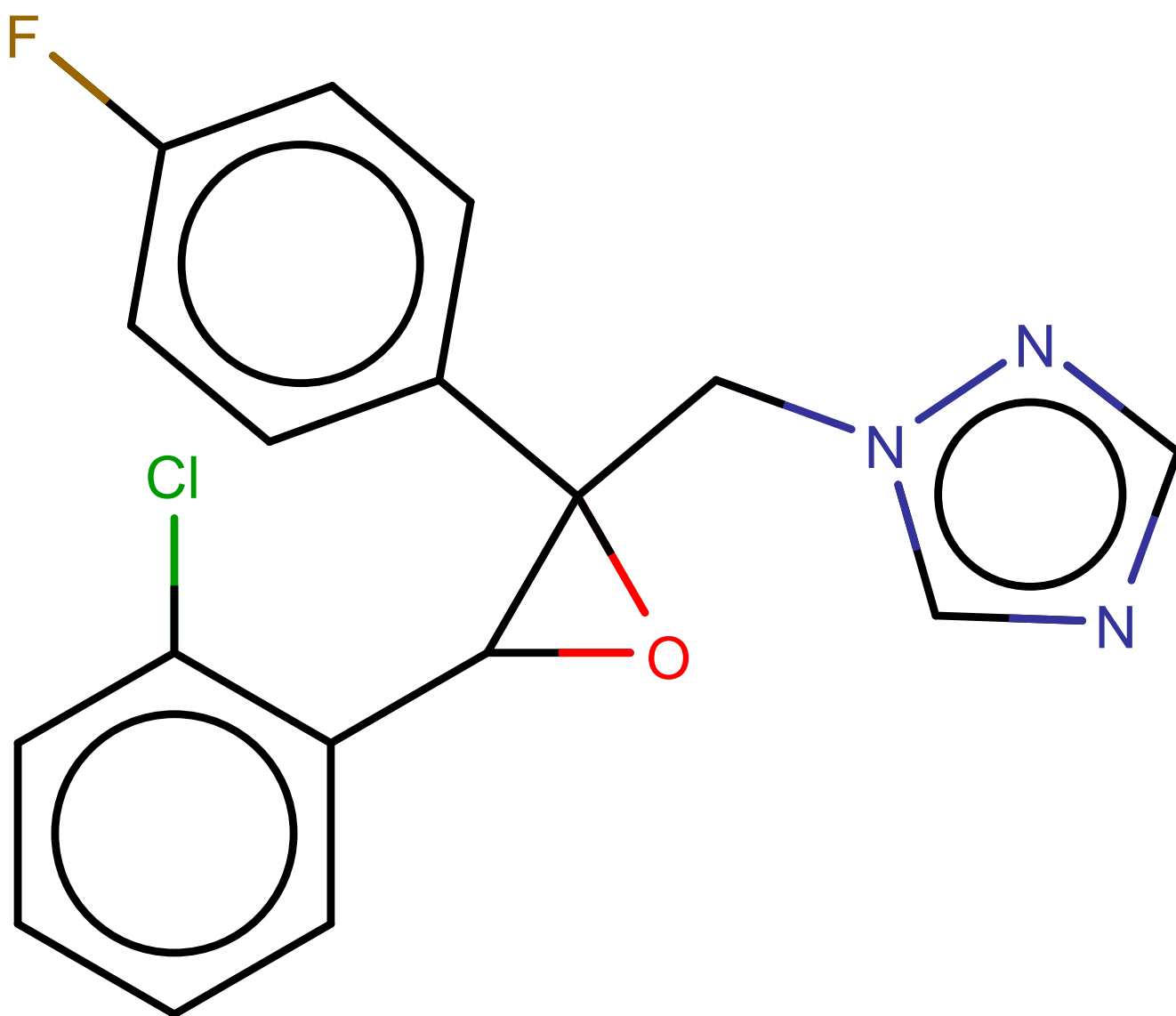

Supplement: Supplementary file 1 [file toxics-12-00425-s001.zip › Supplementary Materials/2D chemical structures/1750.pdf]

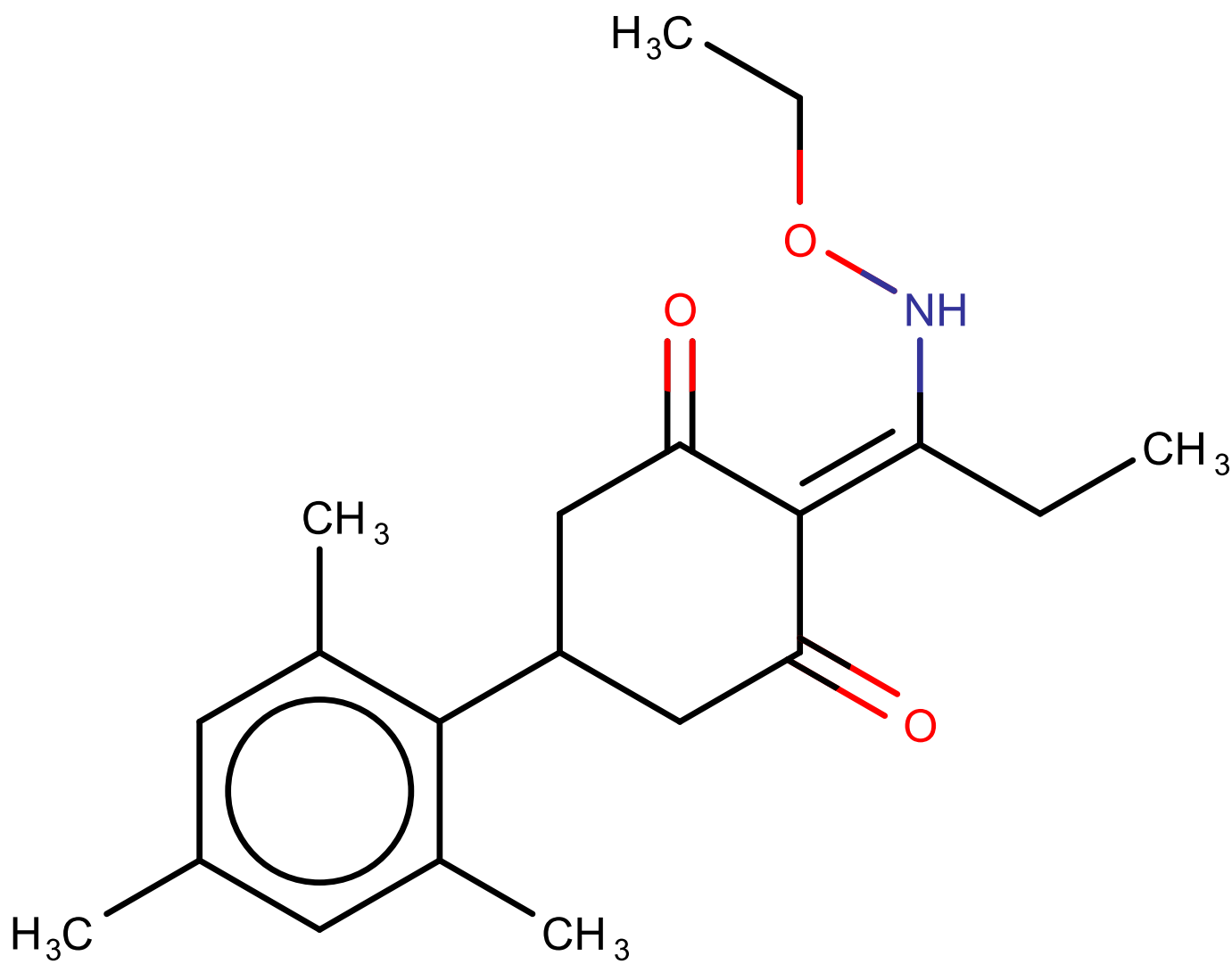

Supplement: Supplementary file 1 [file toxics-12-00425-s001.zip › Supplementary Materials/2D chemical structures/1751.pdf]

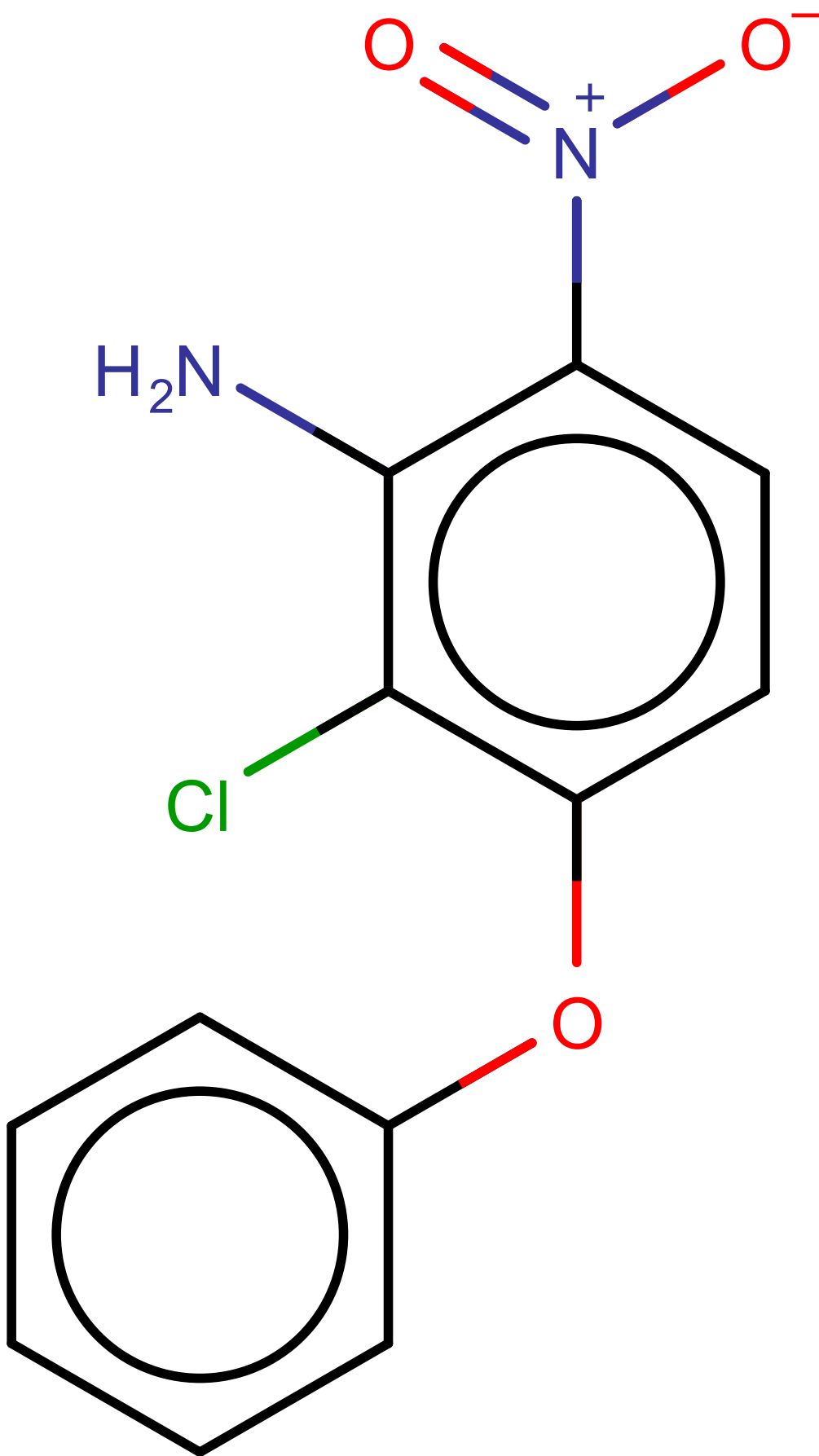

Supplement: Supplementary file 1 [file toxics-12-00425-s001.zip › Supplementary Materials/2D chemical structures/1757.pdf]

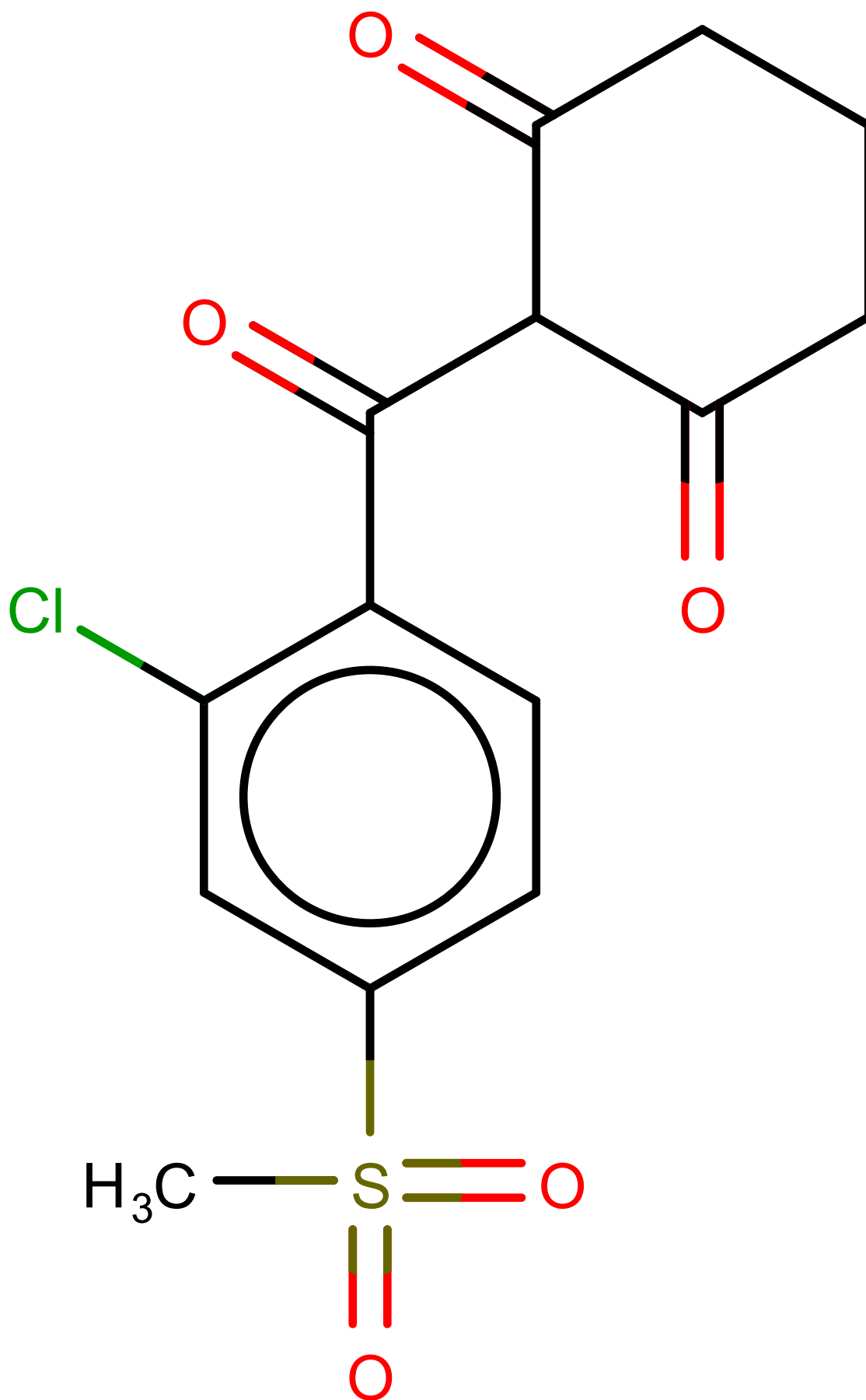

Supplement: Supplementary file 1 [file toxics-12-00425-s001.zip › Supplementary Materials/2D chemical structures/1758.pdf]

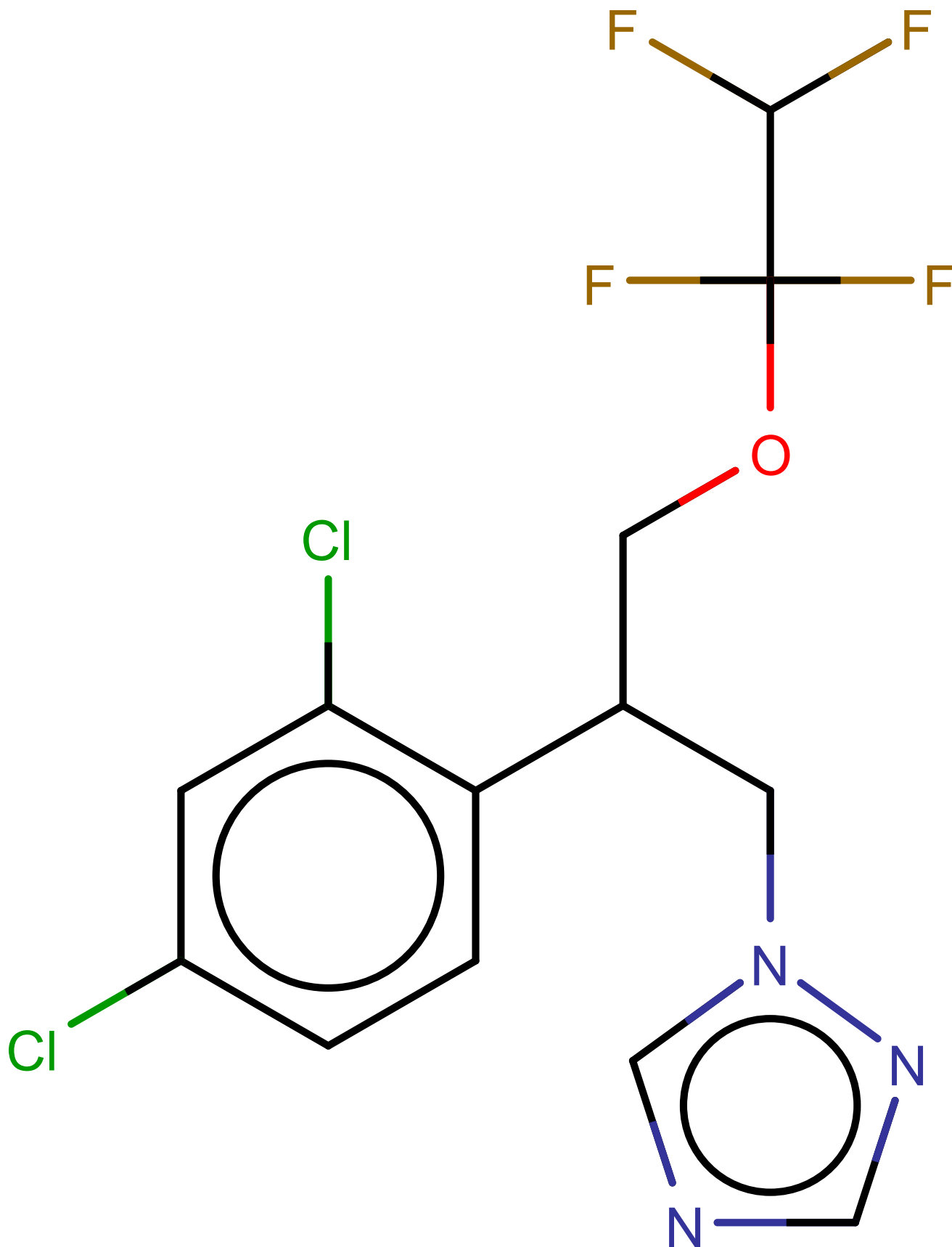

Supplement: Supplementary file 1 [file toxics-12-00425-s001.zip › Supplementary Materials/2D chemical structures/1760.pdf]

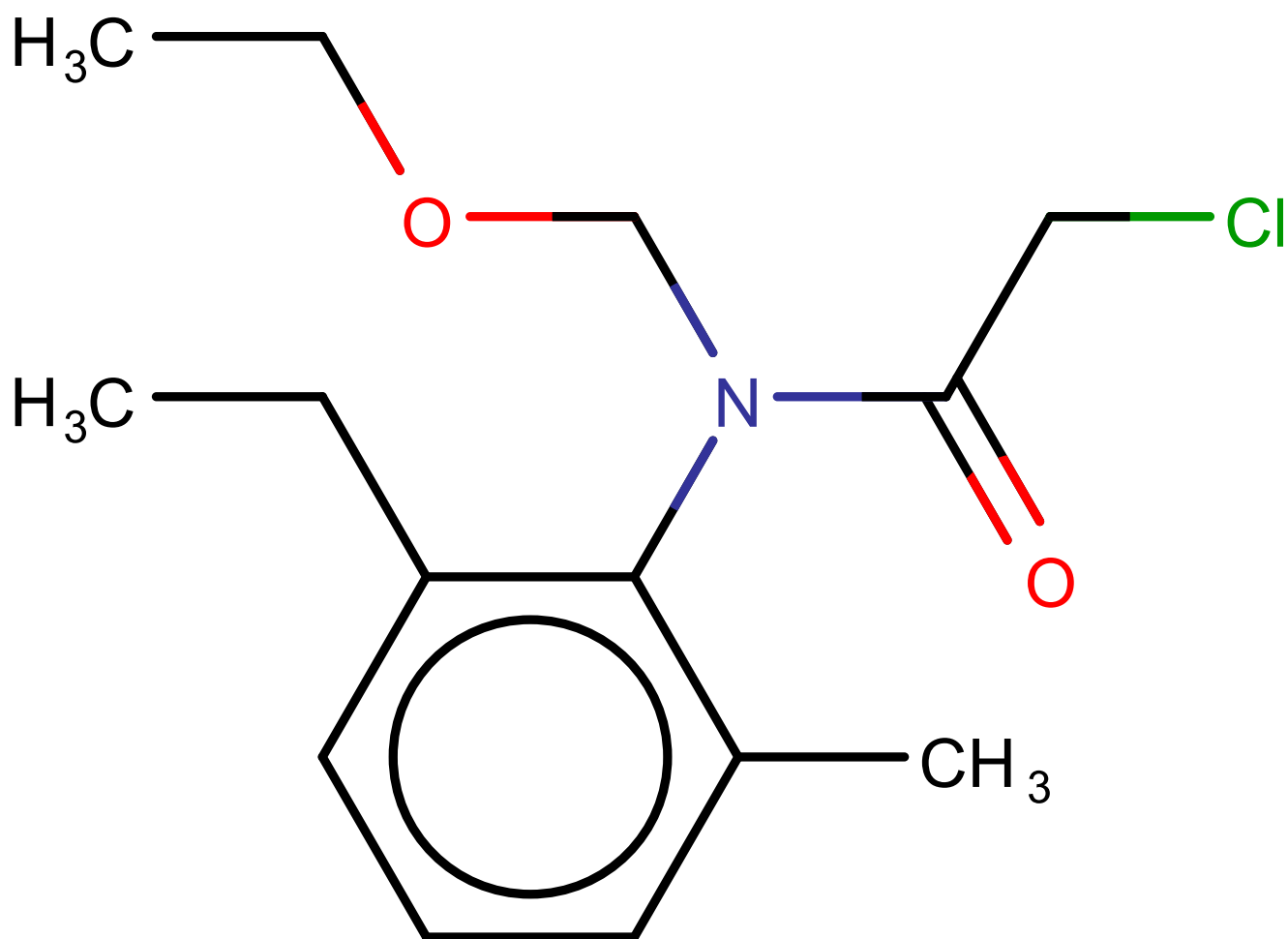

Supplement: Supplementary file 1 [file toxics-12-00425-s001.zip › Supplementary Materials/2D chemical structures/1761.pdf]

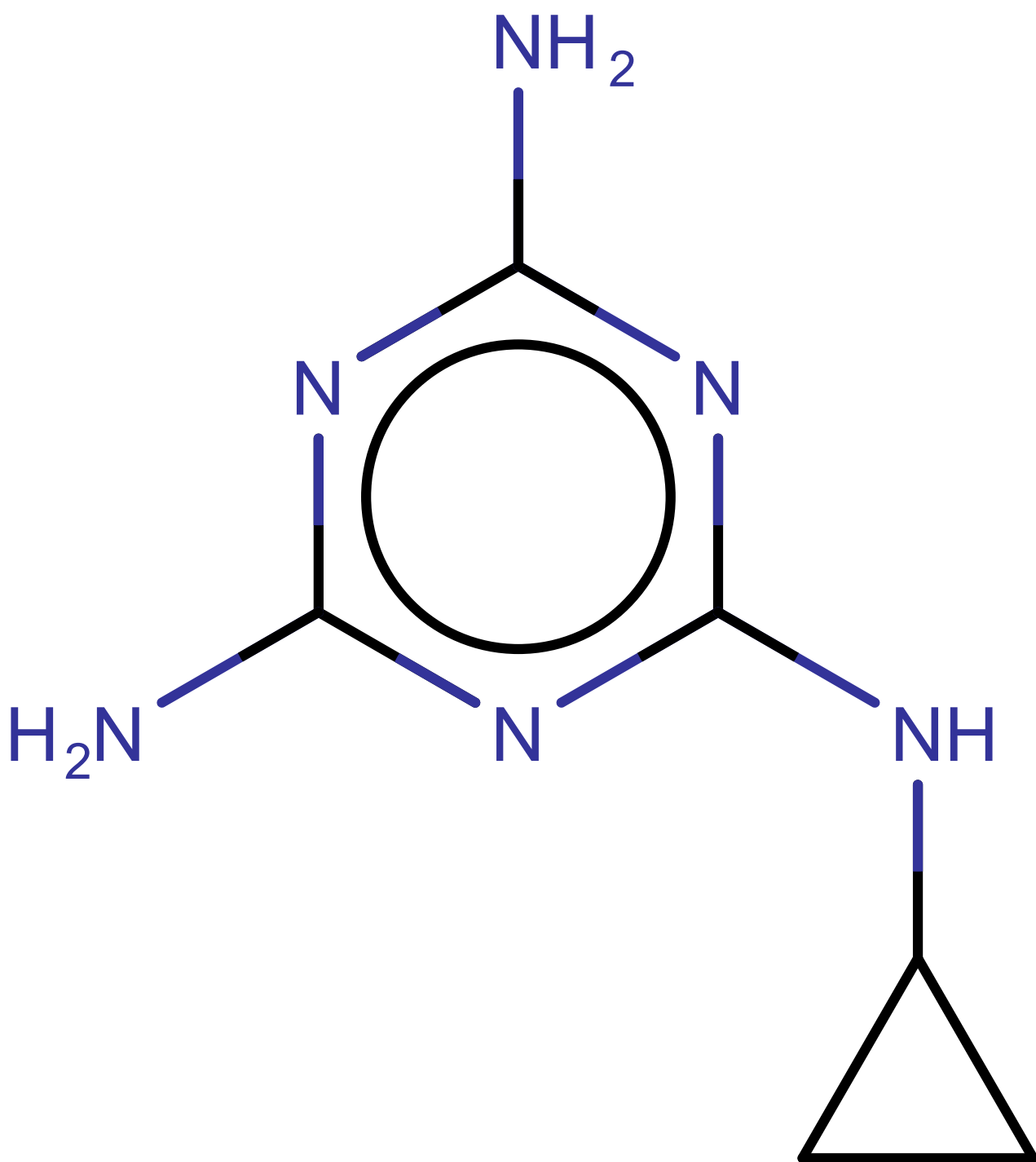

Supplement: Supplementary file 1 [file toxics-12-00425-s001.zip › Supplementary Materials/2D chemical structures/1763.pdf]

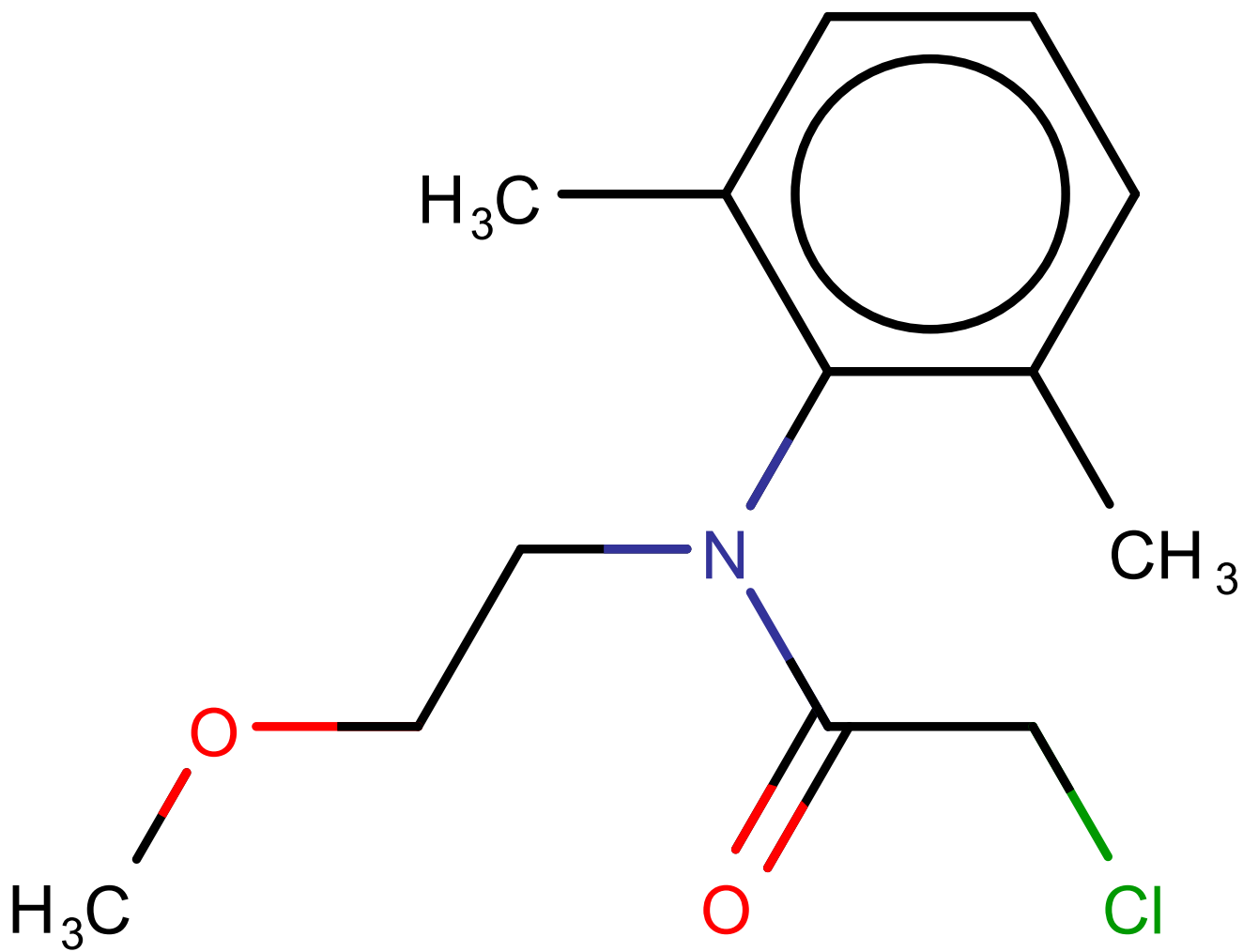

Supplement: Supplementary file 1 [file toxics-12-00425-s001.zip › Supplementary Materials/2D chemical structures/1764.pdf]

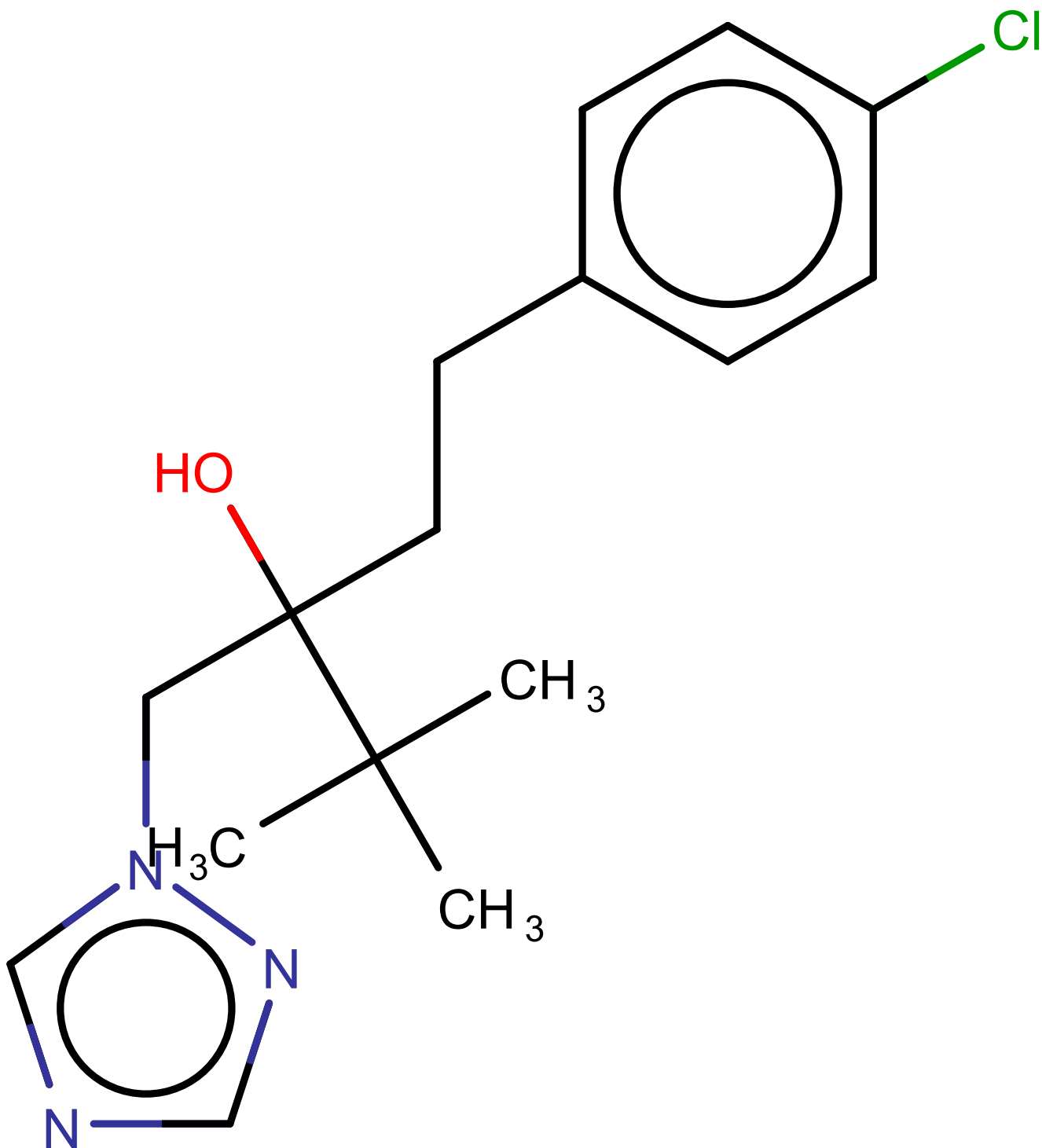

Supplement: Supplementary file 1 [file toxics-12-00425-s001.zip › Supplementary Materials/2D chemical structures/1768.pdf]

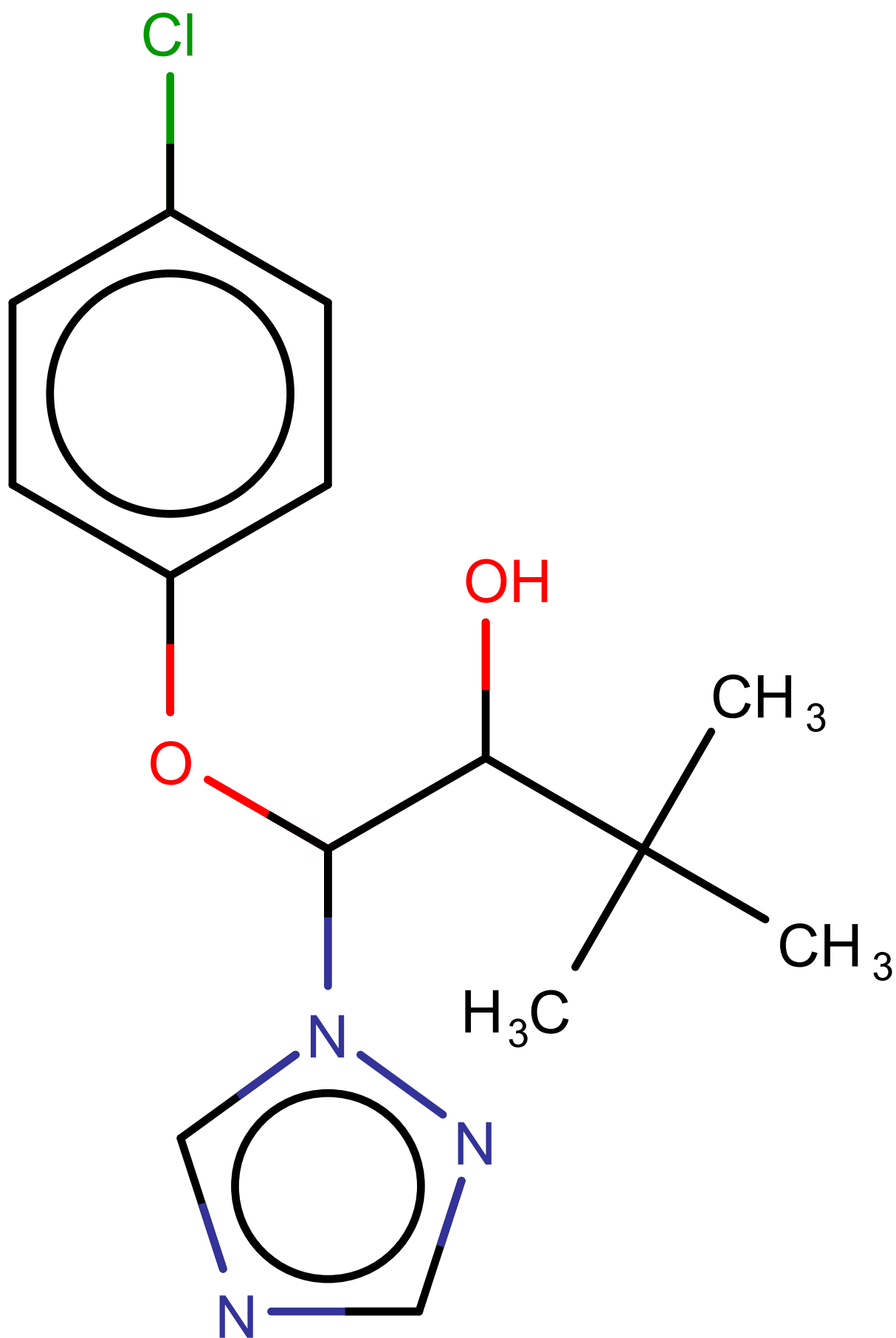

Supplement: Supplementary file 1 [file toxics-12-00425-s001.zip › Supplementary Materials/2D chemical structures/1769.pdf]

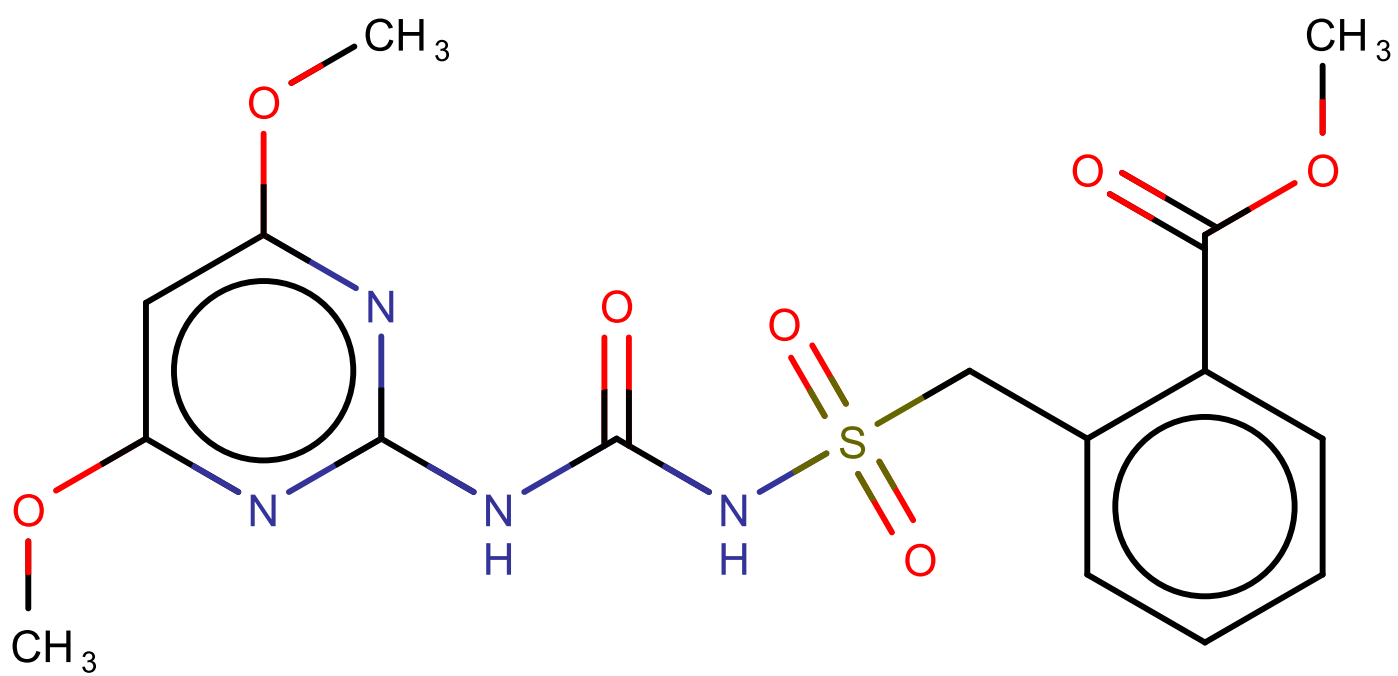

Supplement: Supplementary file 1 [file toxics-12-00425-s001.zip › Supplementary Materials/2D chemical structures/1770.pdf]

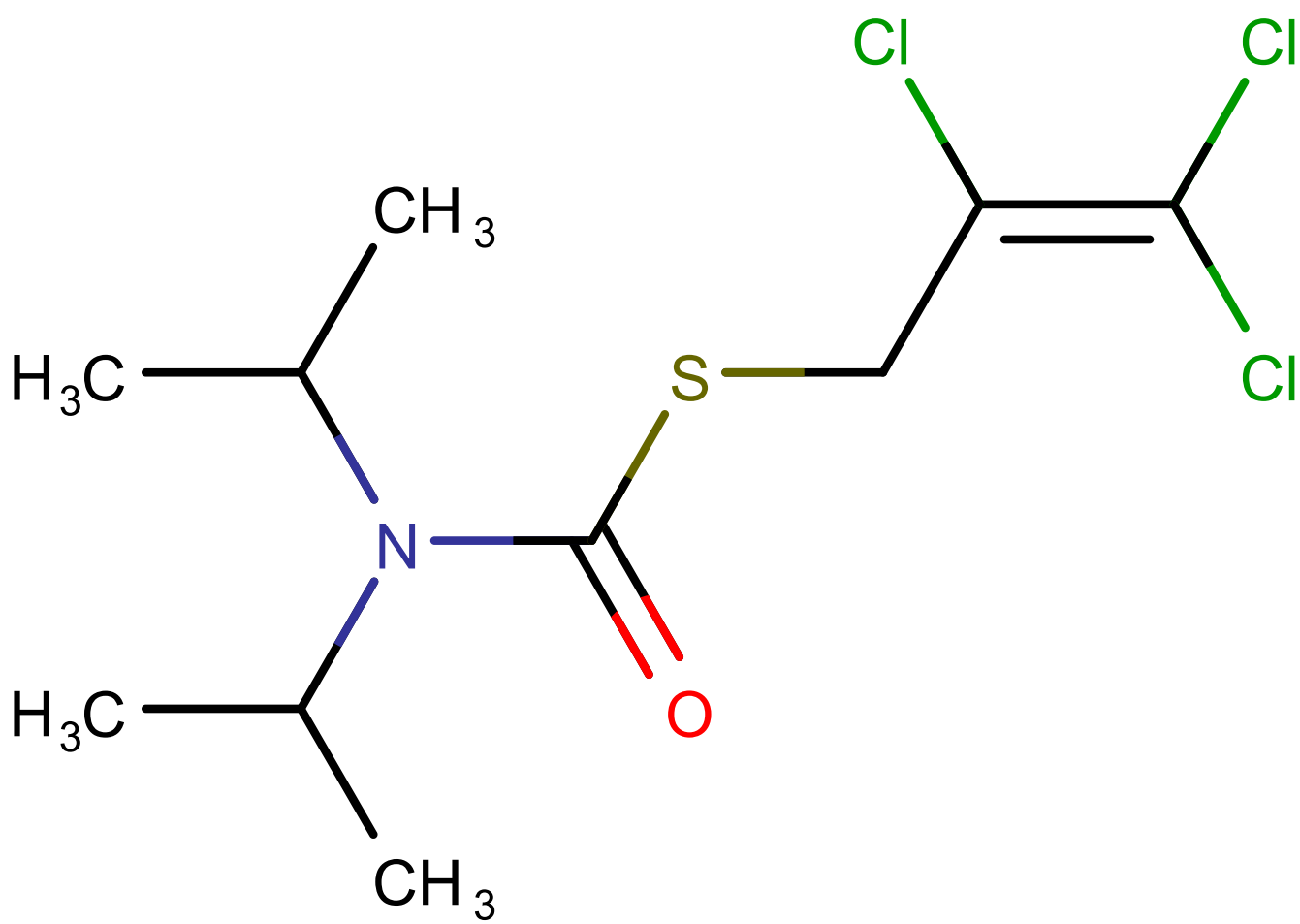

Supplement: Supplementary file 1 [file toxics-12-00425-s001.zip › Supplementary Materials/2D chemical structures/1773.pdf]

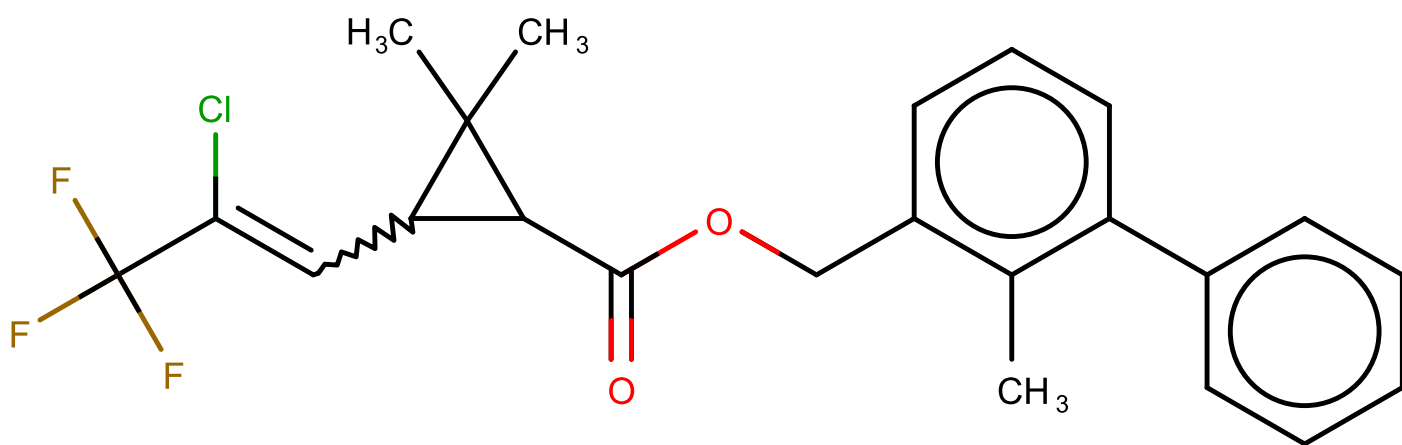

Supplement: Supplementary file 1 [file toxics-12-00425-s001.zip › Supplementary Materials/2D chemical structures/1778.pdf]

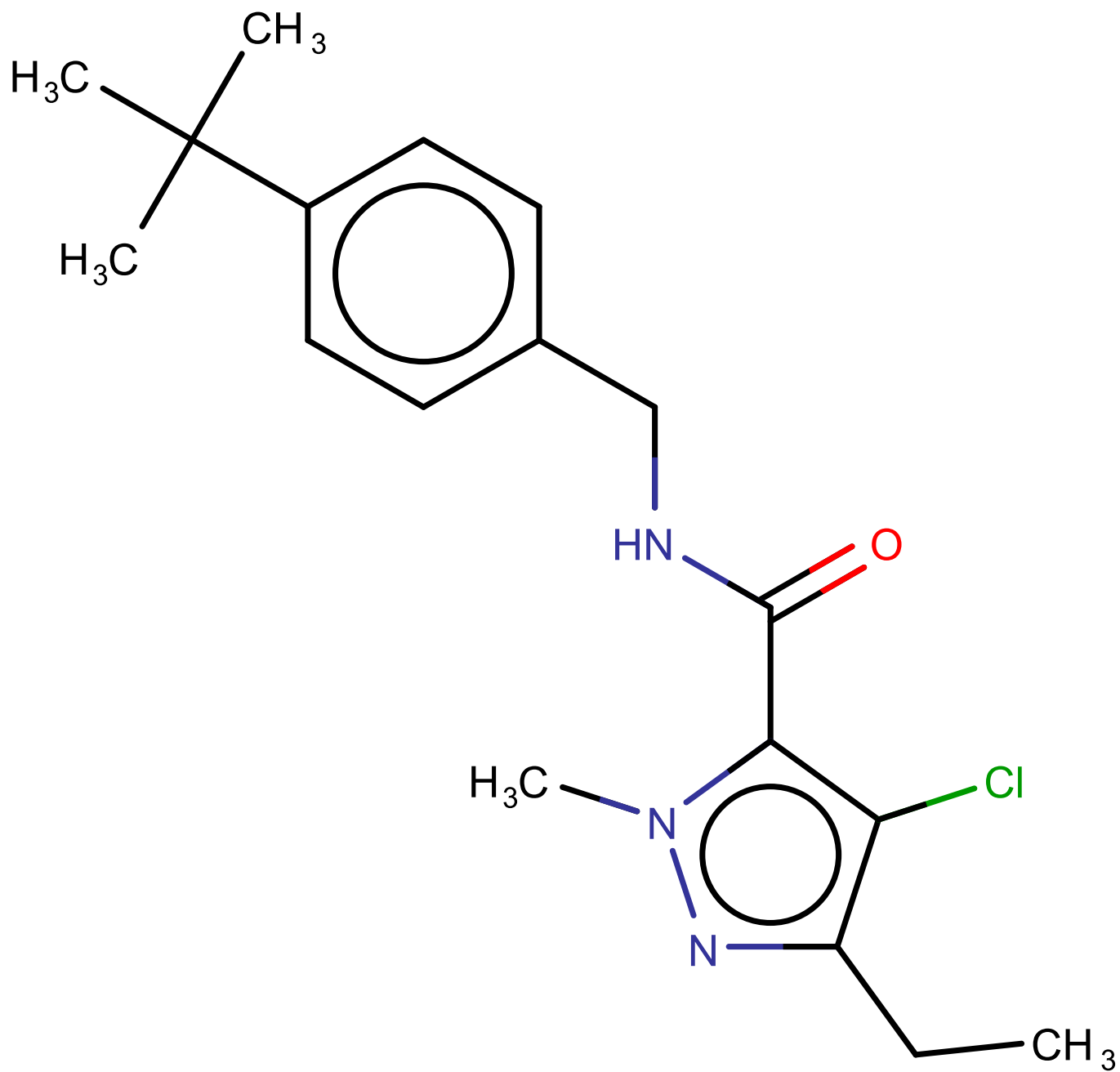

Supplement: Supplementary file 1 [file toxics-12-00425-s001.zip › Supplementary Materials/2D chemical structures/1782.pdf]

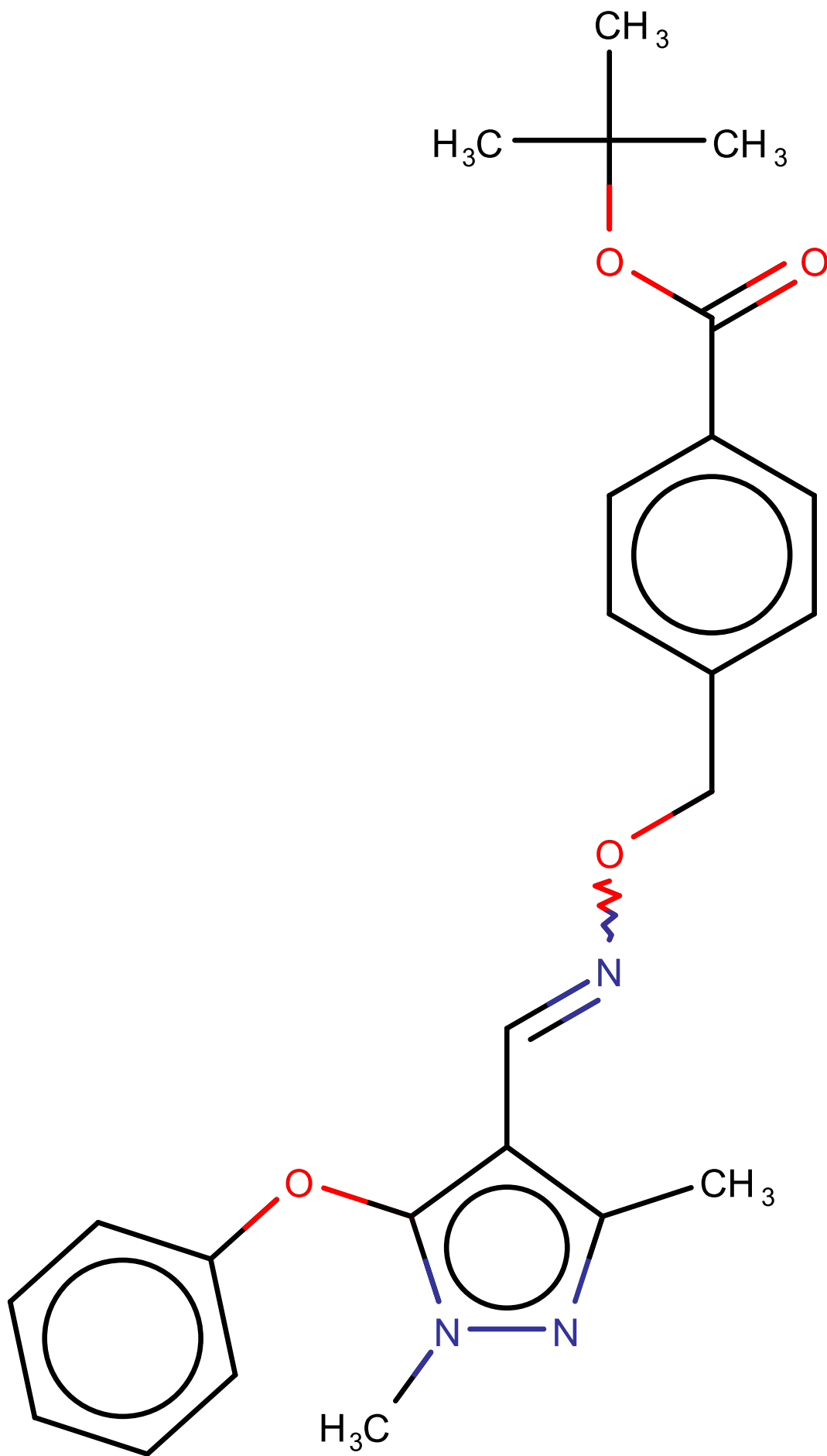

Supplement: Supplementary file 1 [file toxics-12-00425-s001.zip › Supplementary Materials/2D chemical structures/1785.pdf]

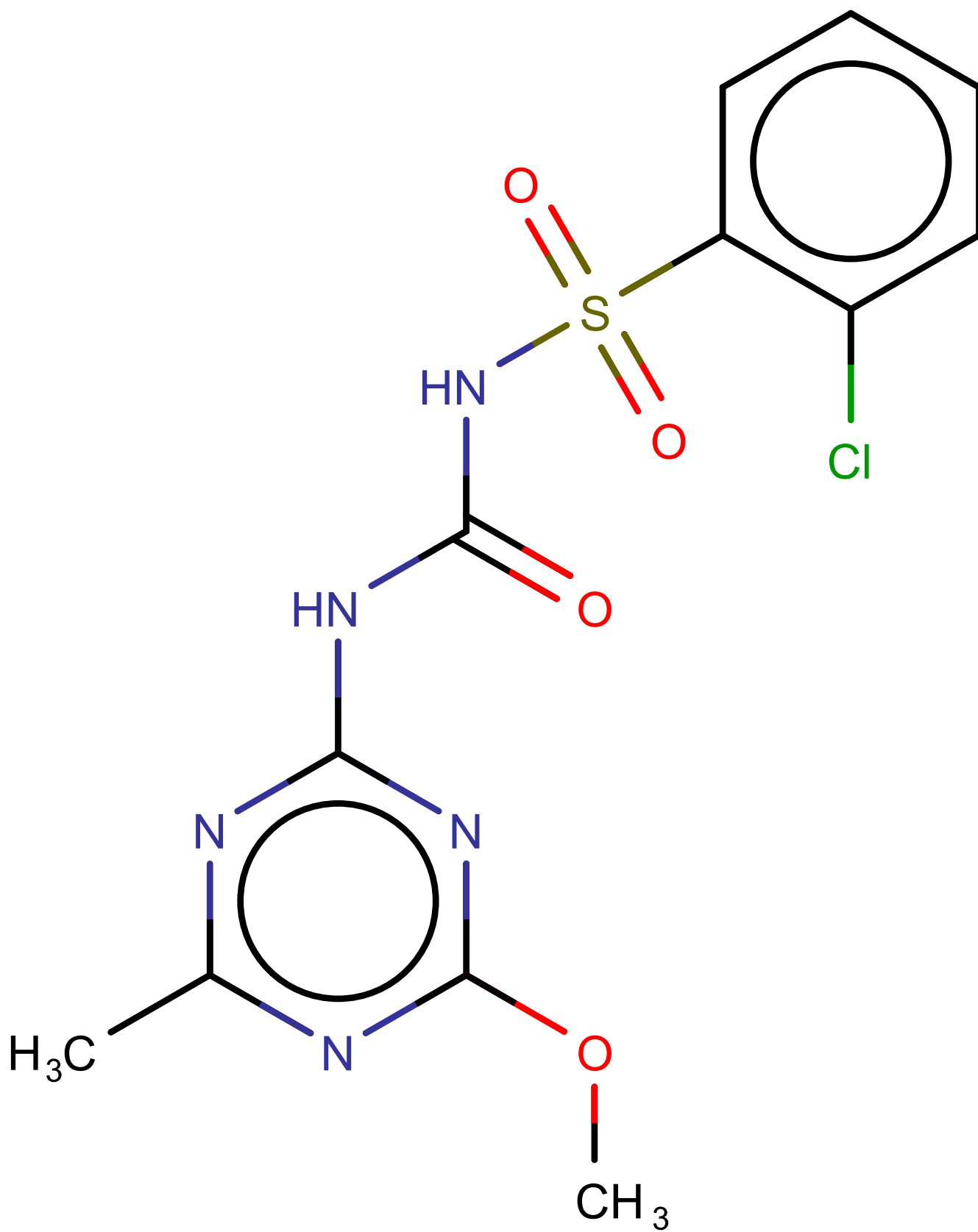

Supplement: Supplementary file 1 [file toxics-12-00425-s001.zip › Supplementary Materials/2D chemical structures/1786.pdf]

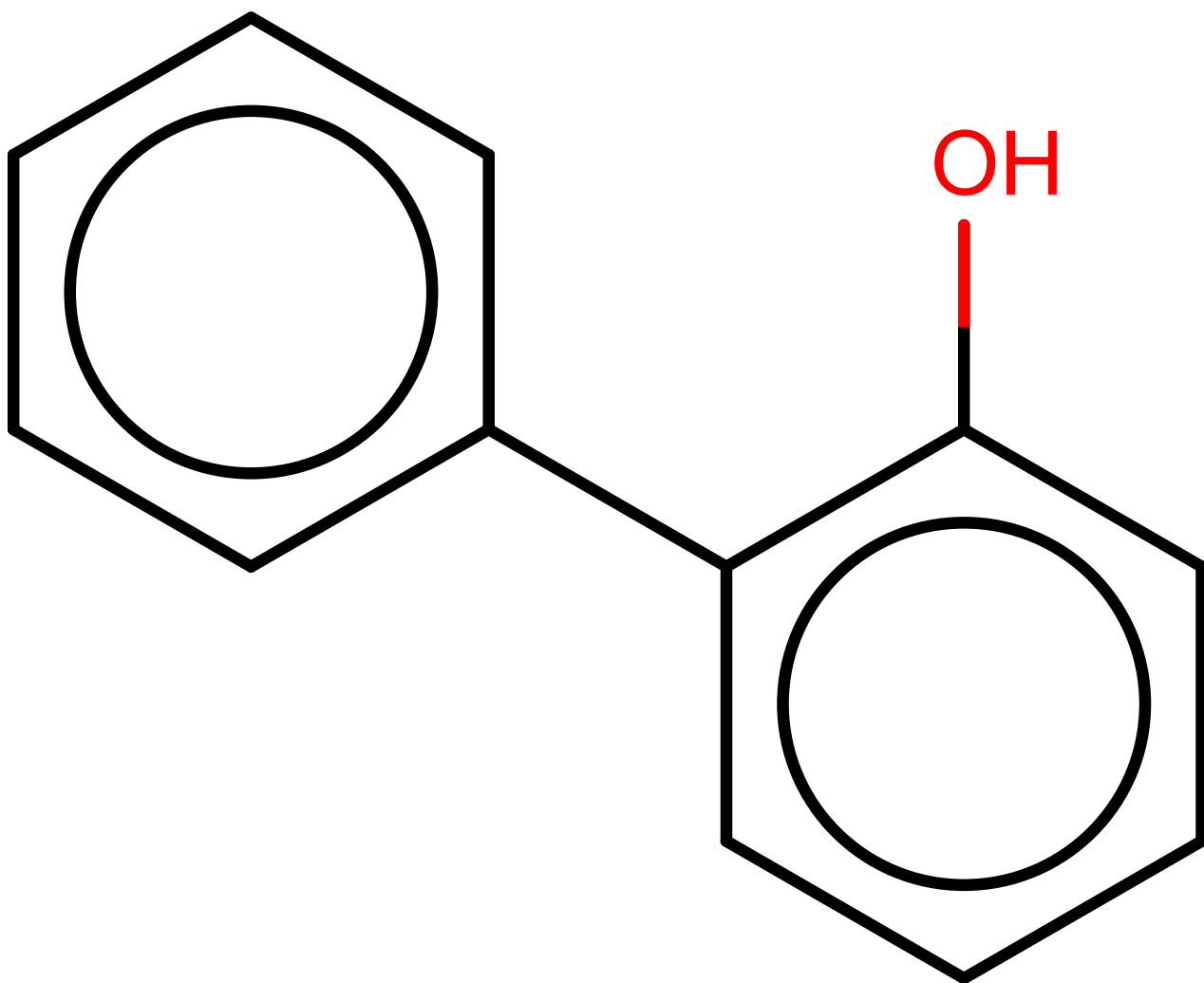

Supplement: Supplementary file 1 [file toxics-12-00425-s001.zip › Supplementary Materials/2D chemical structures/1791.pdf]

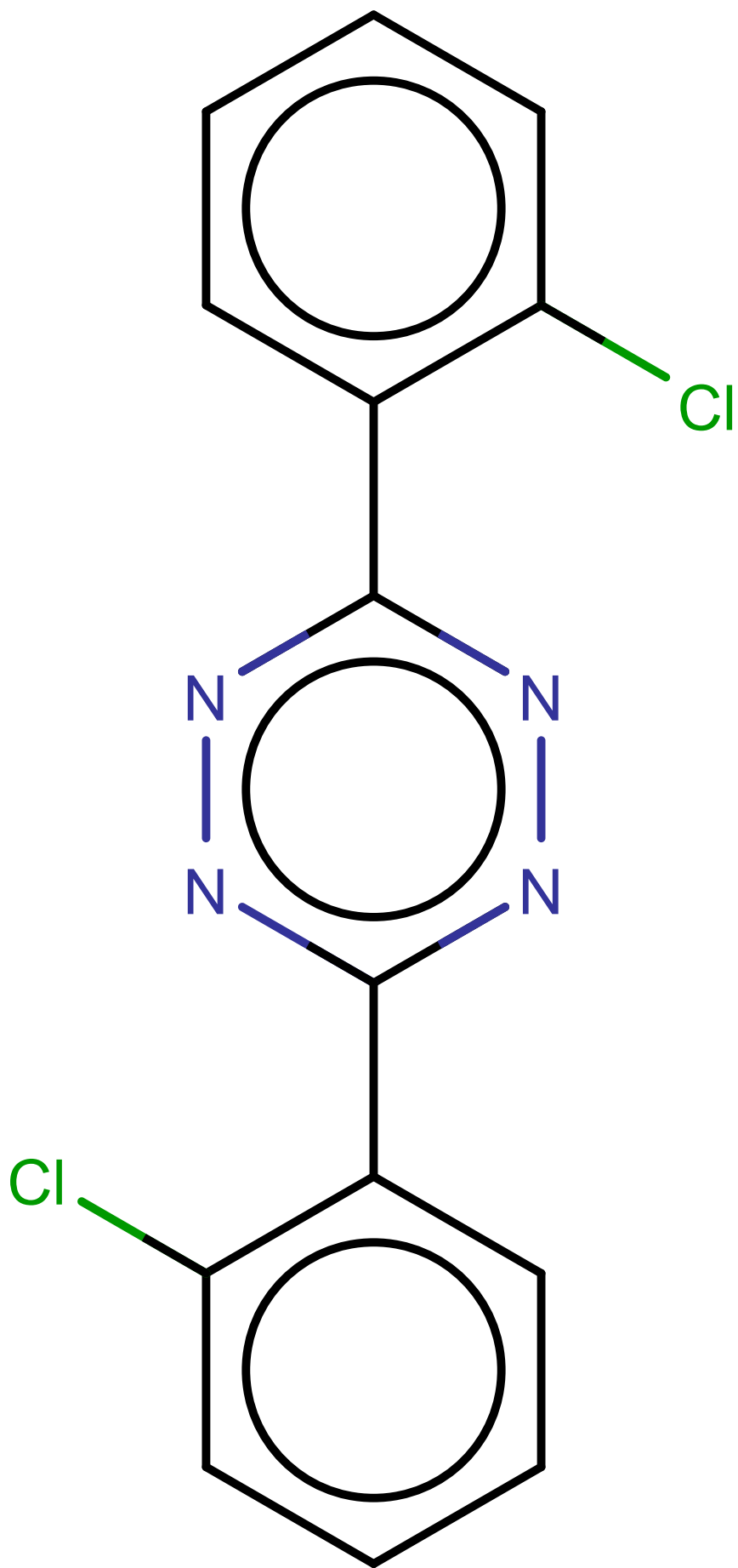

Supplement: Supplementary file 1 [file toxics-12-00425-s001.zip › Supplementary Materials/2D chemical structures/1794.pdf]

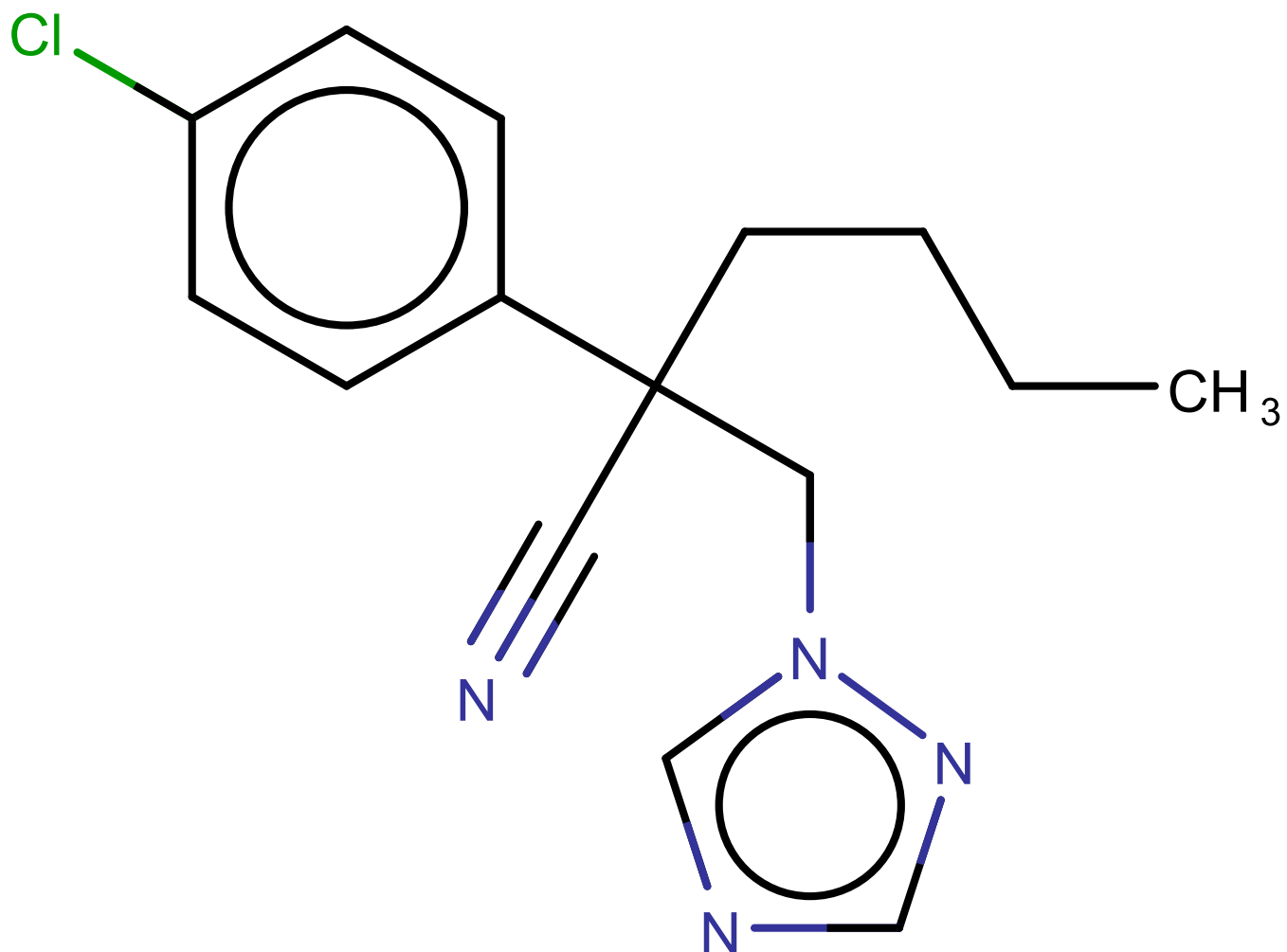

Supplement: Supplementary file 1 [file toxics-12-00425-s001.zip › Supplementary Materials/2D chemical structures/1797.pdf]

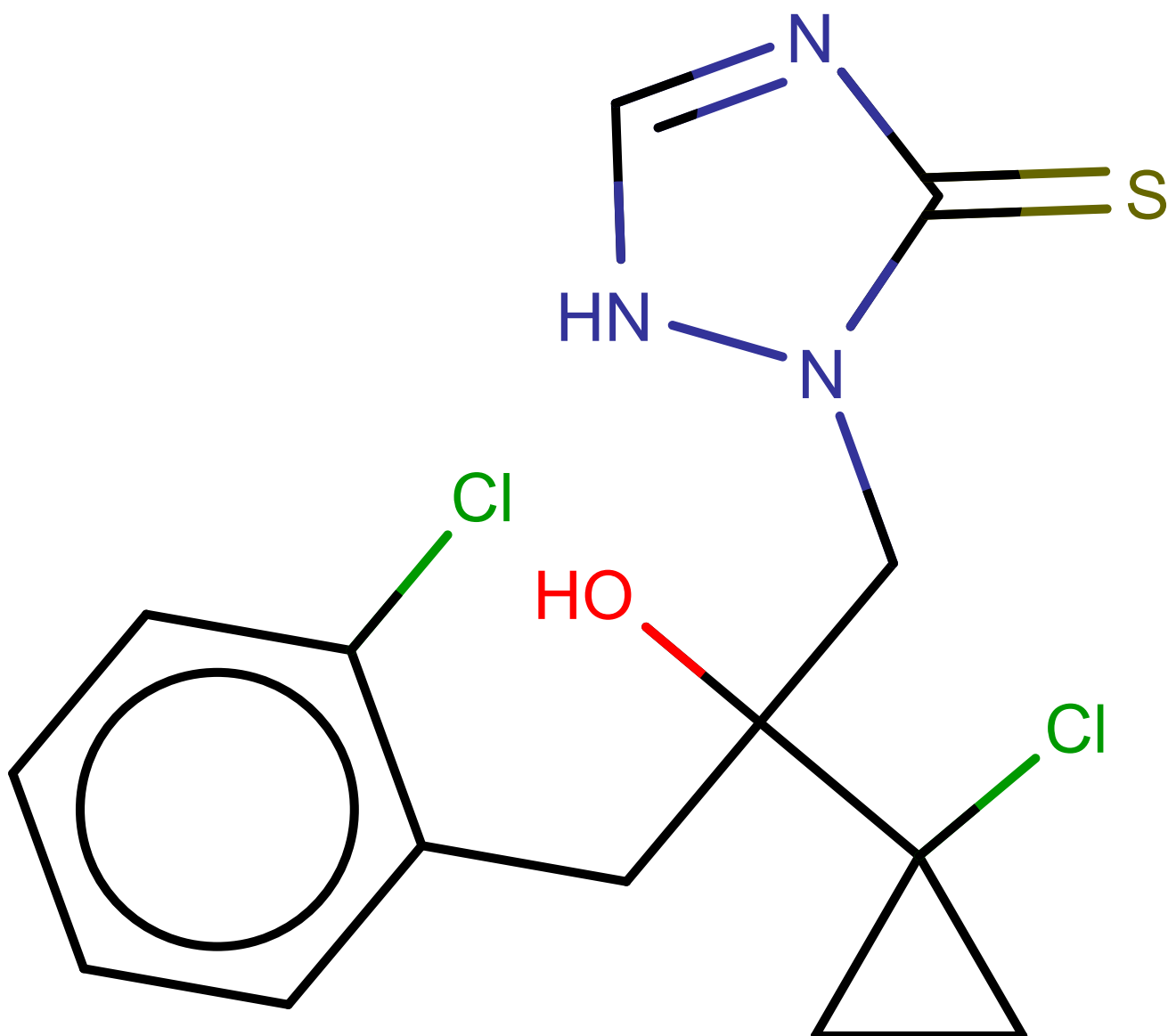

Supplement: Supplementary file 1 [file toxics-12-00425-s001.zip › Supplementary Materials/2D chemical structures/1800.pdf]

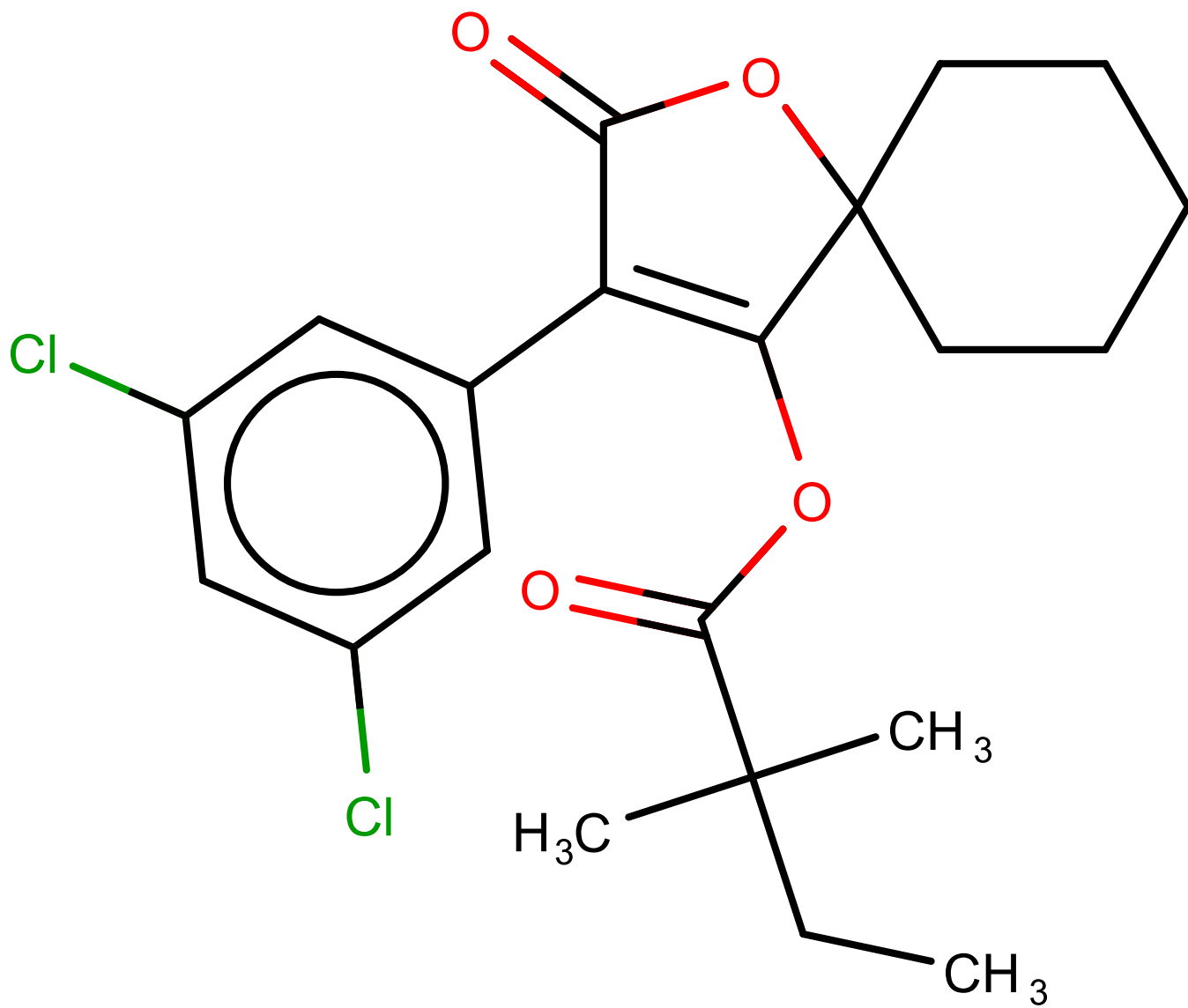

Supplement: Supplementary file 1 [file toxics-12-00425-s001.zip › Supplementary Materials/2D chemical structures/1805.pdf]

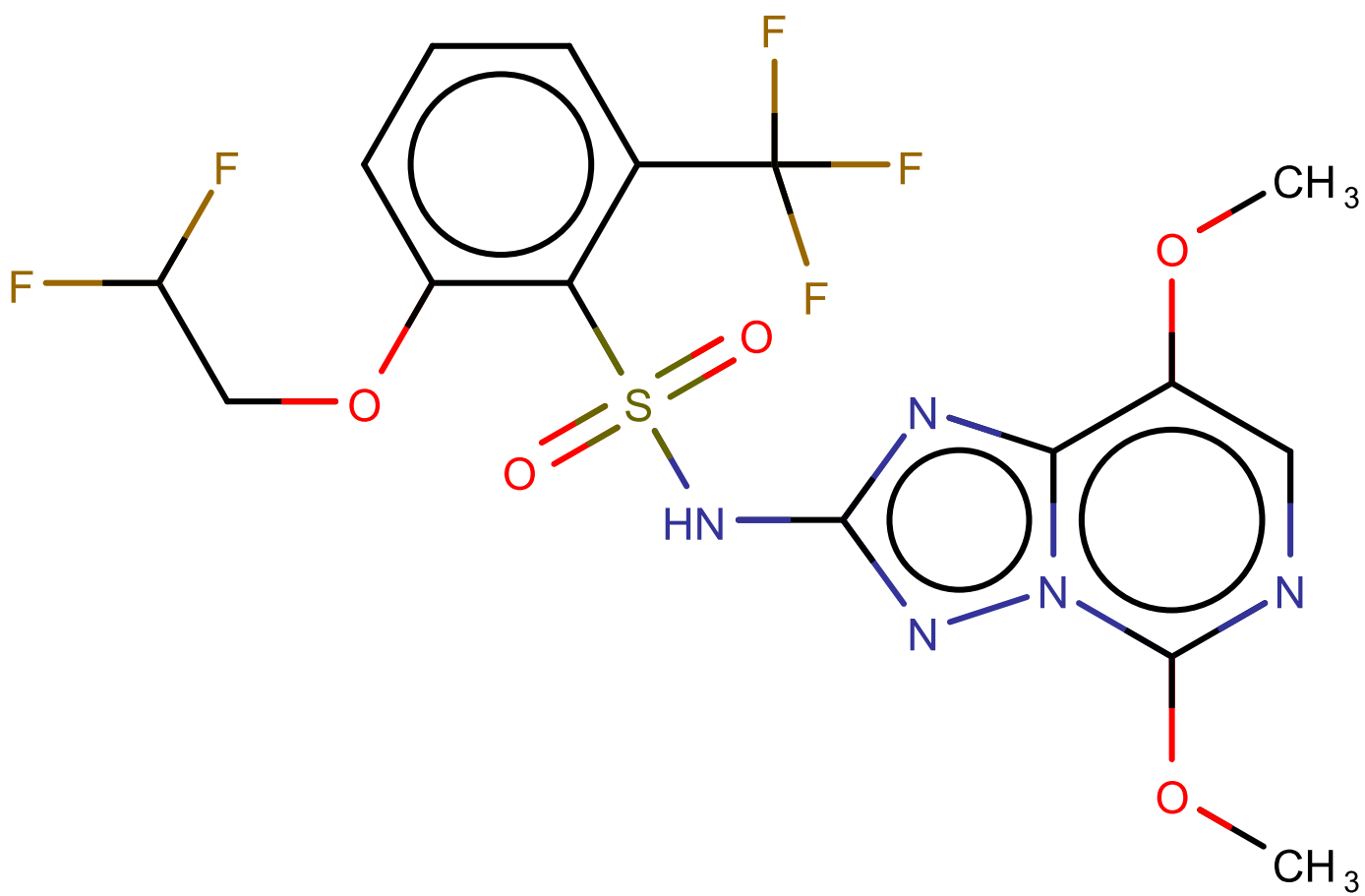

Supplement: Supplementary file 1 [file toxics-12-00425-s001.zip › Supplementary Materials/2D chemical structures/1806.pdf]

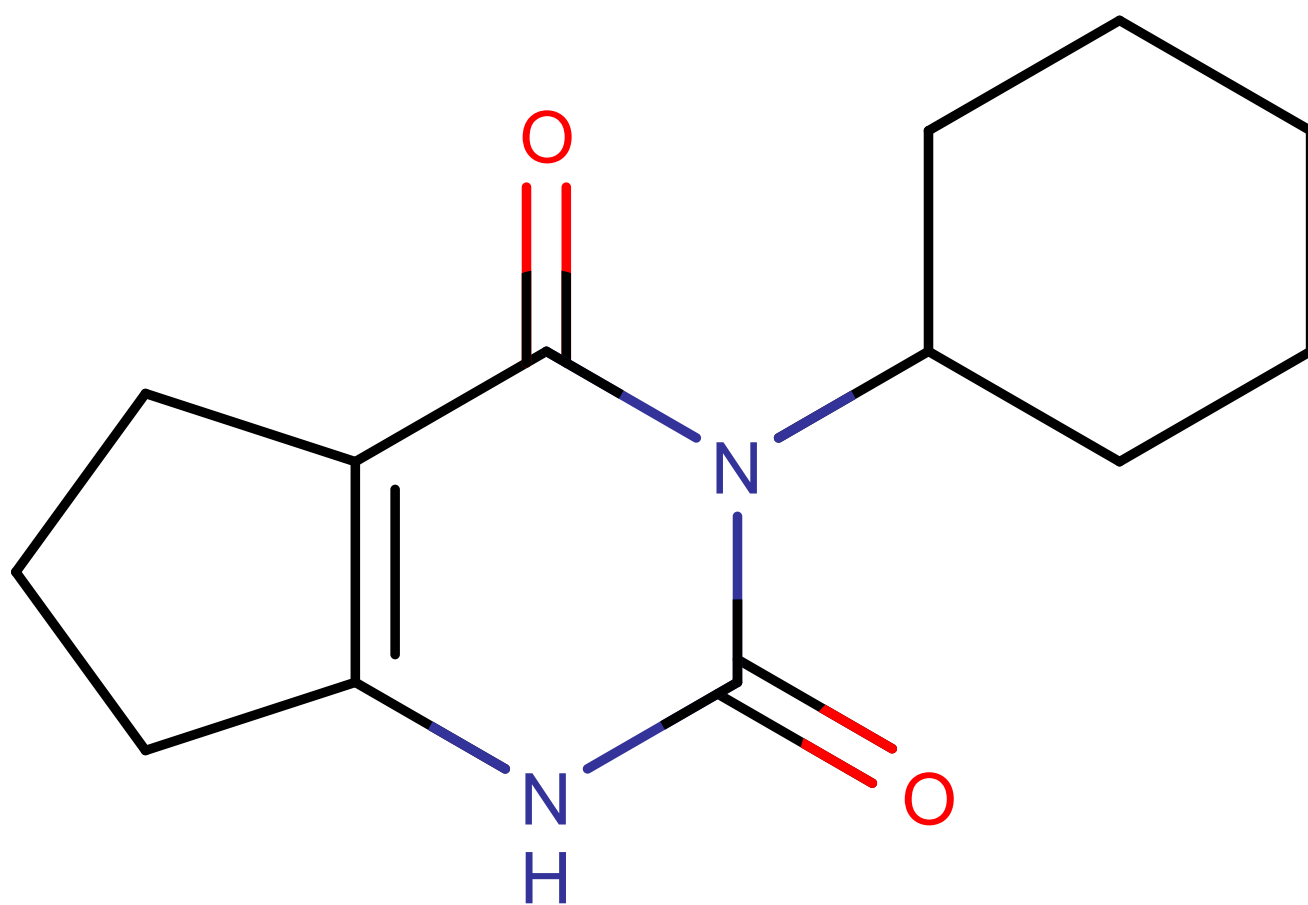

Supplement: Supplementary file 1 [file toxics-12-00425-s001.zip › Supplementary Materials/2D chemical structures/1807.pdf]

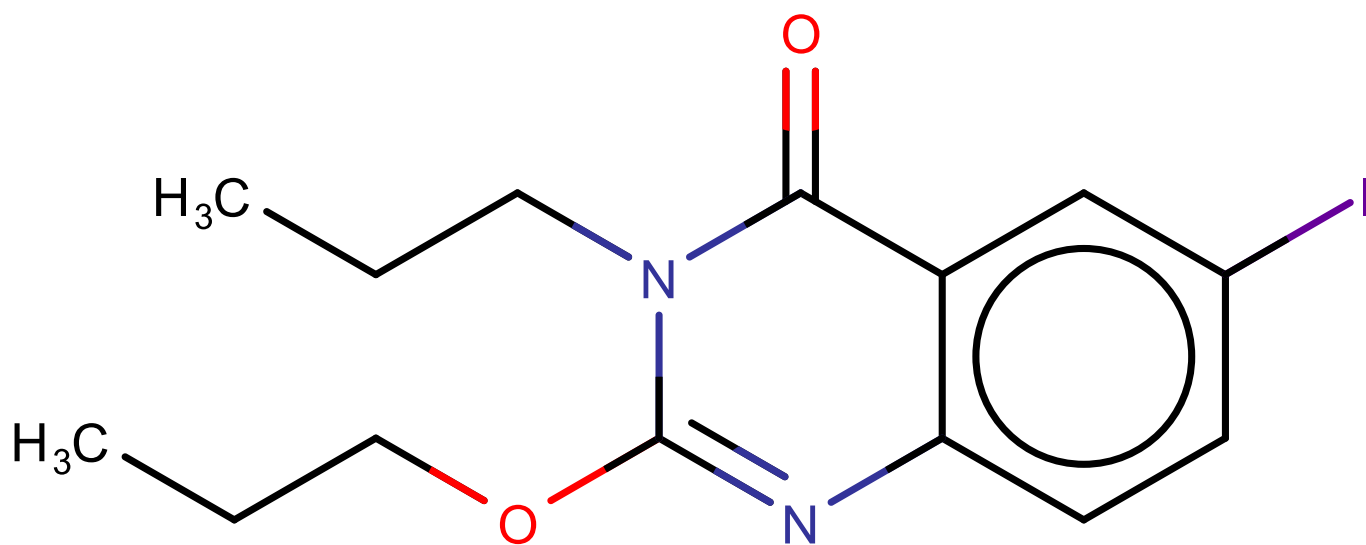

Supplement: Supplementary file 1 [file toxics-12-00425-s001.zip › Supplementary Materials/2D chemical structures/1808.pdf]

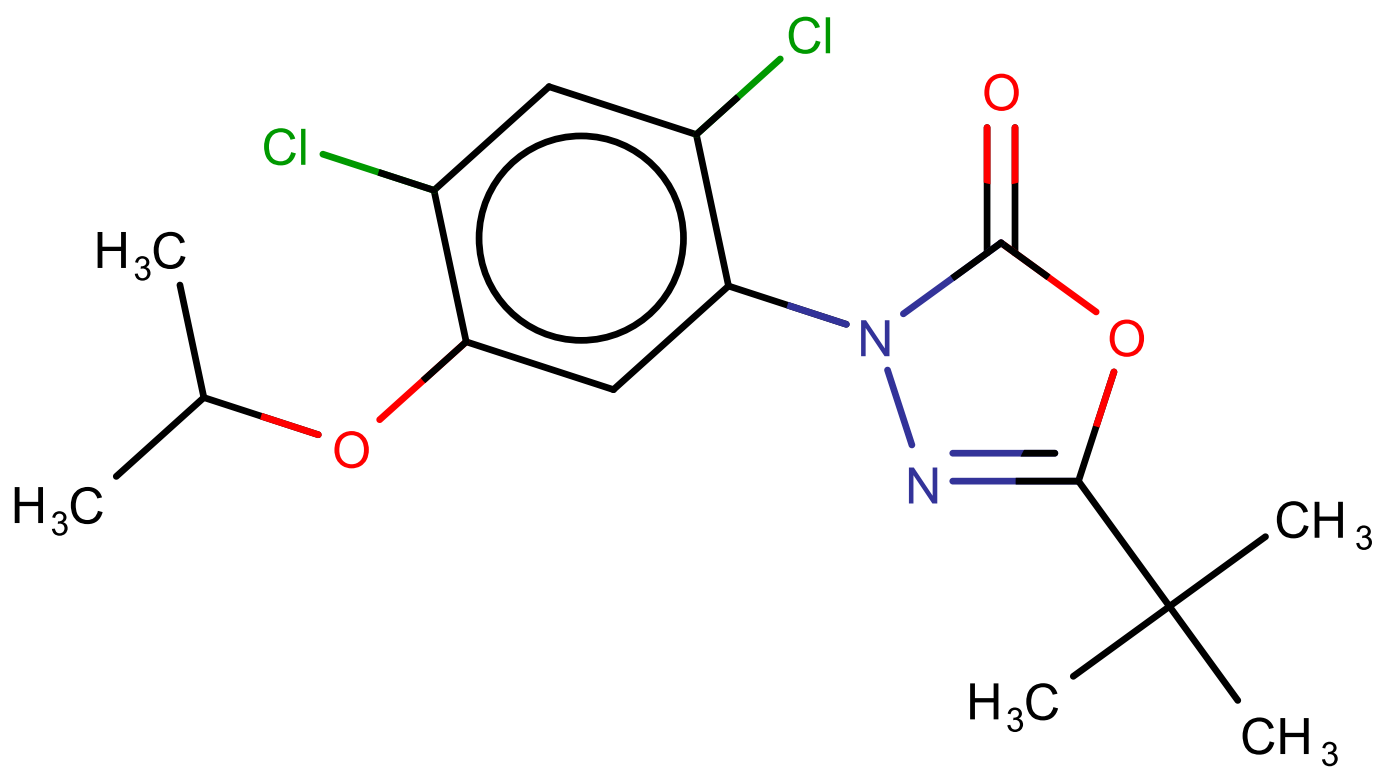

Supplement: Supplementary file 1 [file toxics-12-00425-s001.zip › Supplementary Materials/2D chemical structures/1809.pdf]

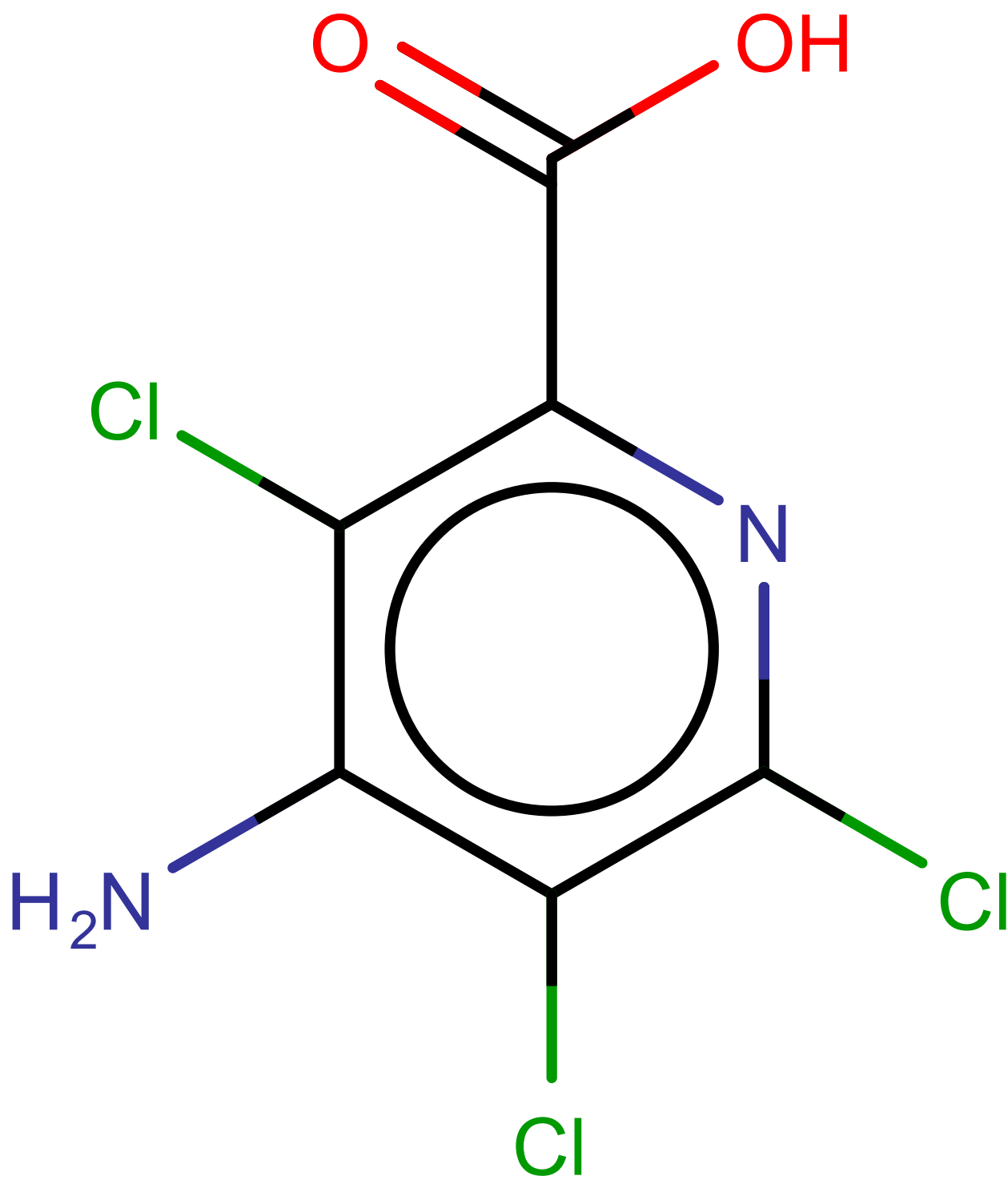

Supplement: Supplementary file 1 [file toxics-12-00425-s001.zip › Supplementary Materials/2D chemical structures/1810.pdf]

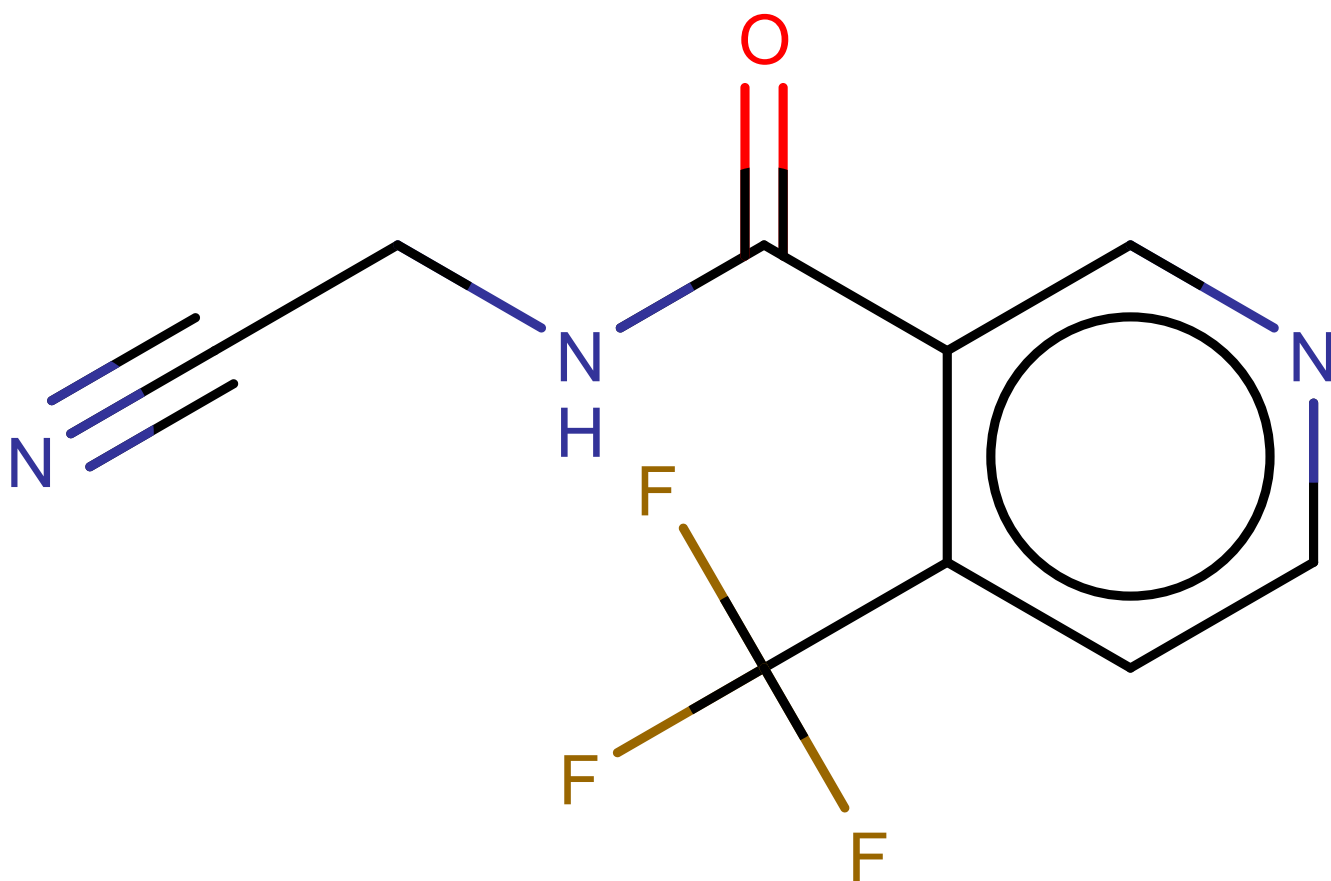

Supplement: Supplementary file 1 [file toxics-12-00425-s001.zip › Supplementary Materials/2D chemical structures/1813.pdf]

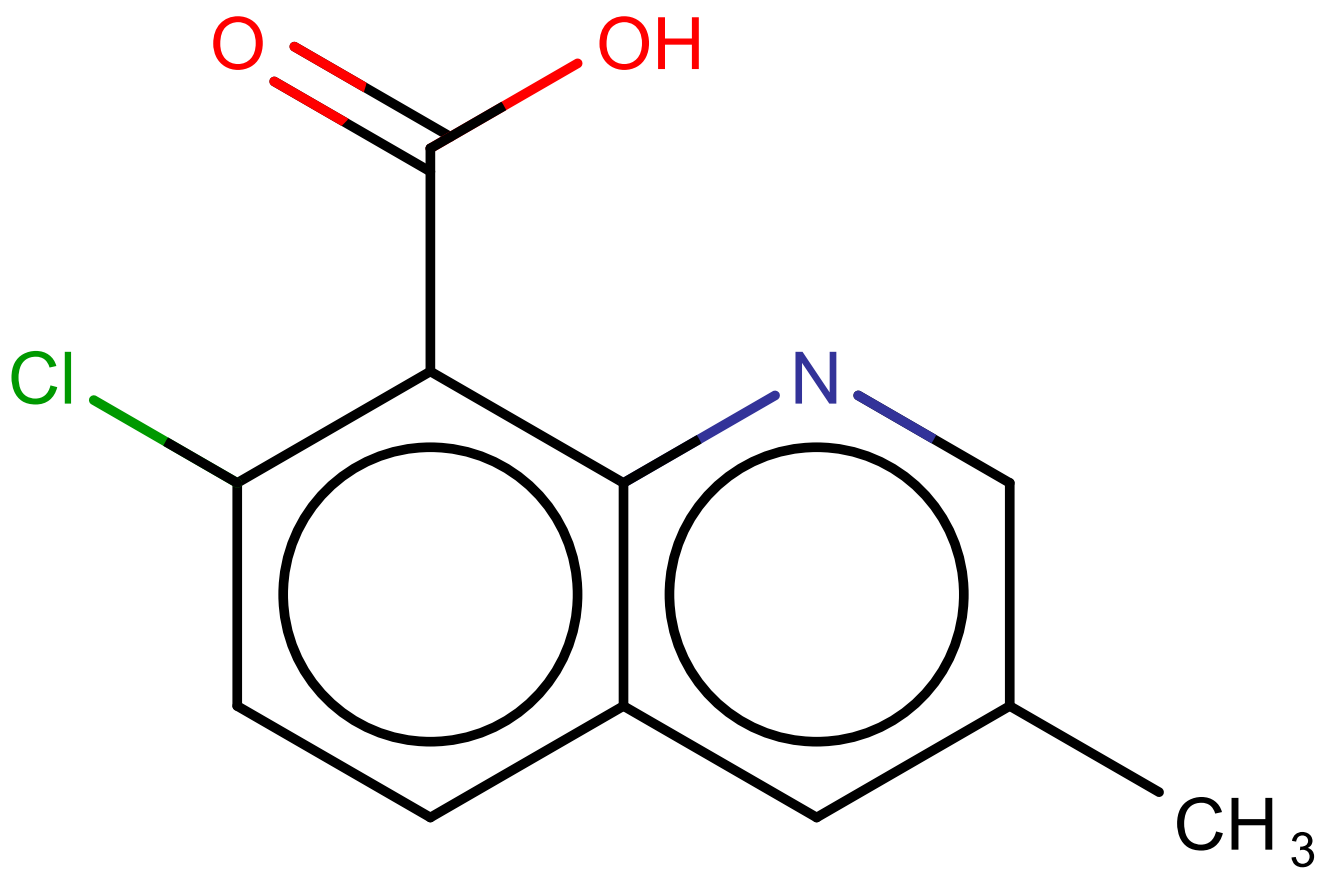

Supplement: Supplementary file 1 [file toxics-12-00425-s001.zip › Supplementary Materials/2D chemical structures/1815.pdf]

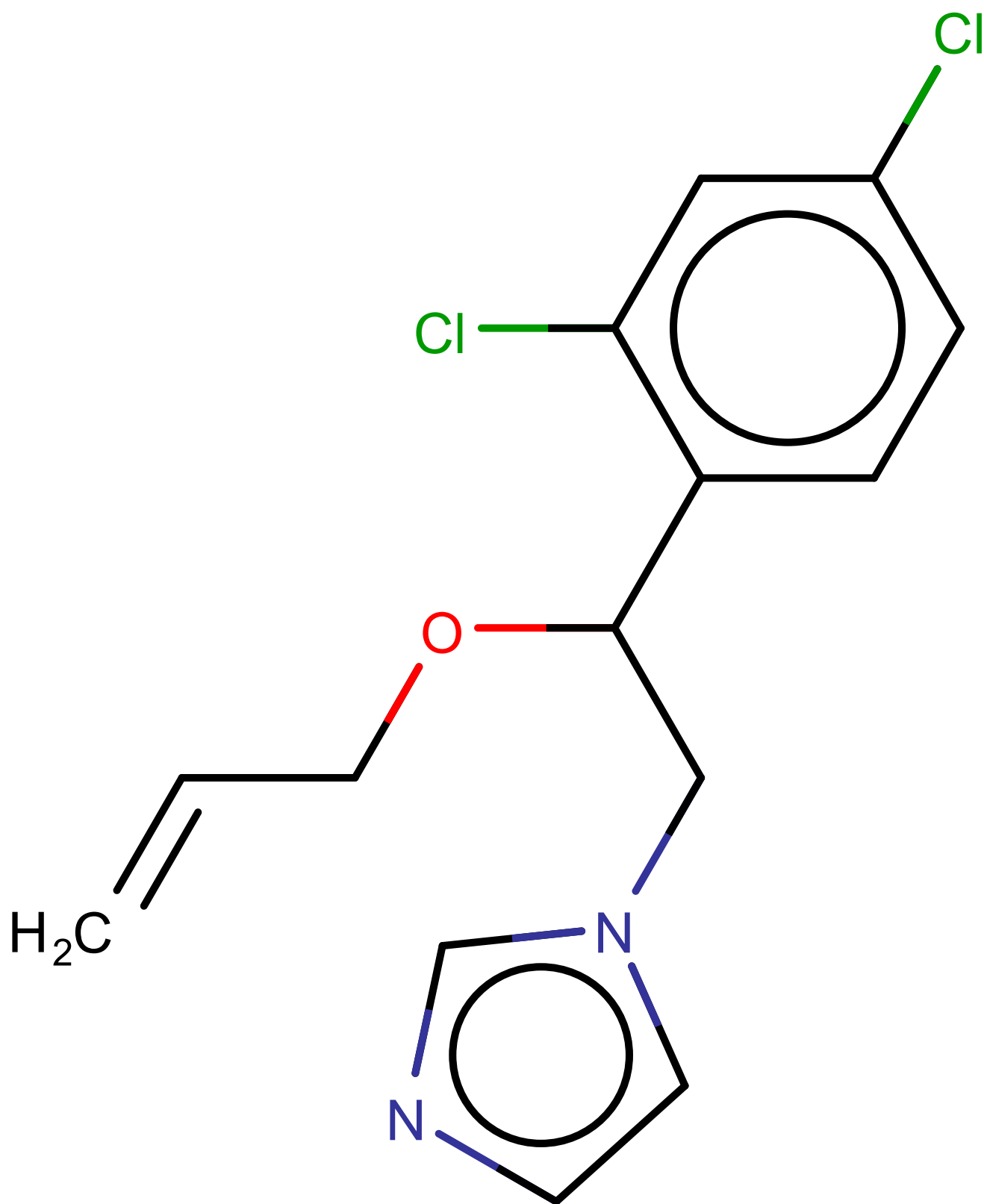

Supplement: Supplementary file 1 [file toxics-12-00425-s001.zip › Supplementary Materials/2D chemical structures/1816.pdf]

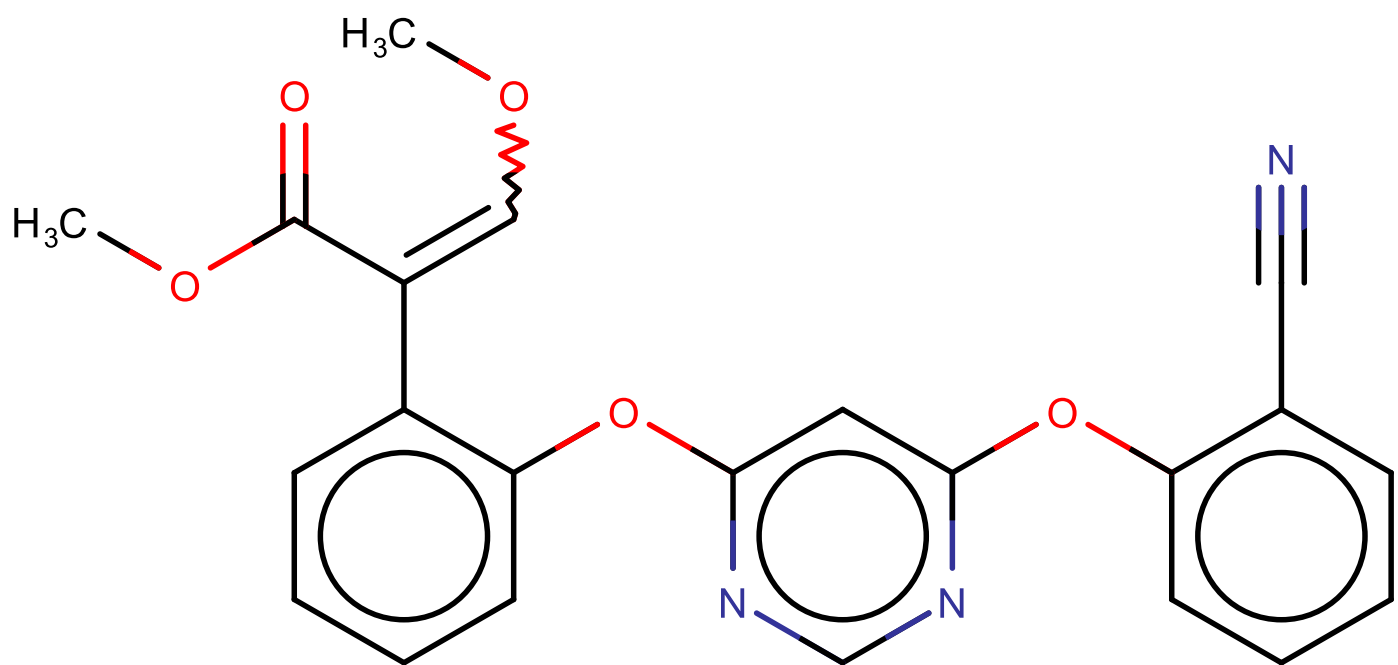

Supplement: Supplementary file 1 [file toxics-12-00425-s001.zip › Supplementary Materials/2D chemical structures/1817.pdf]

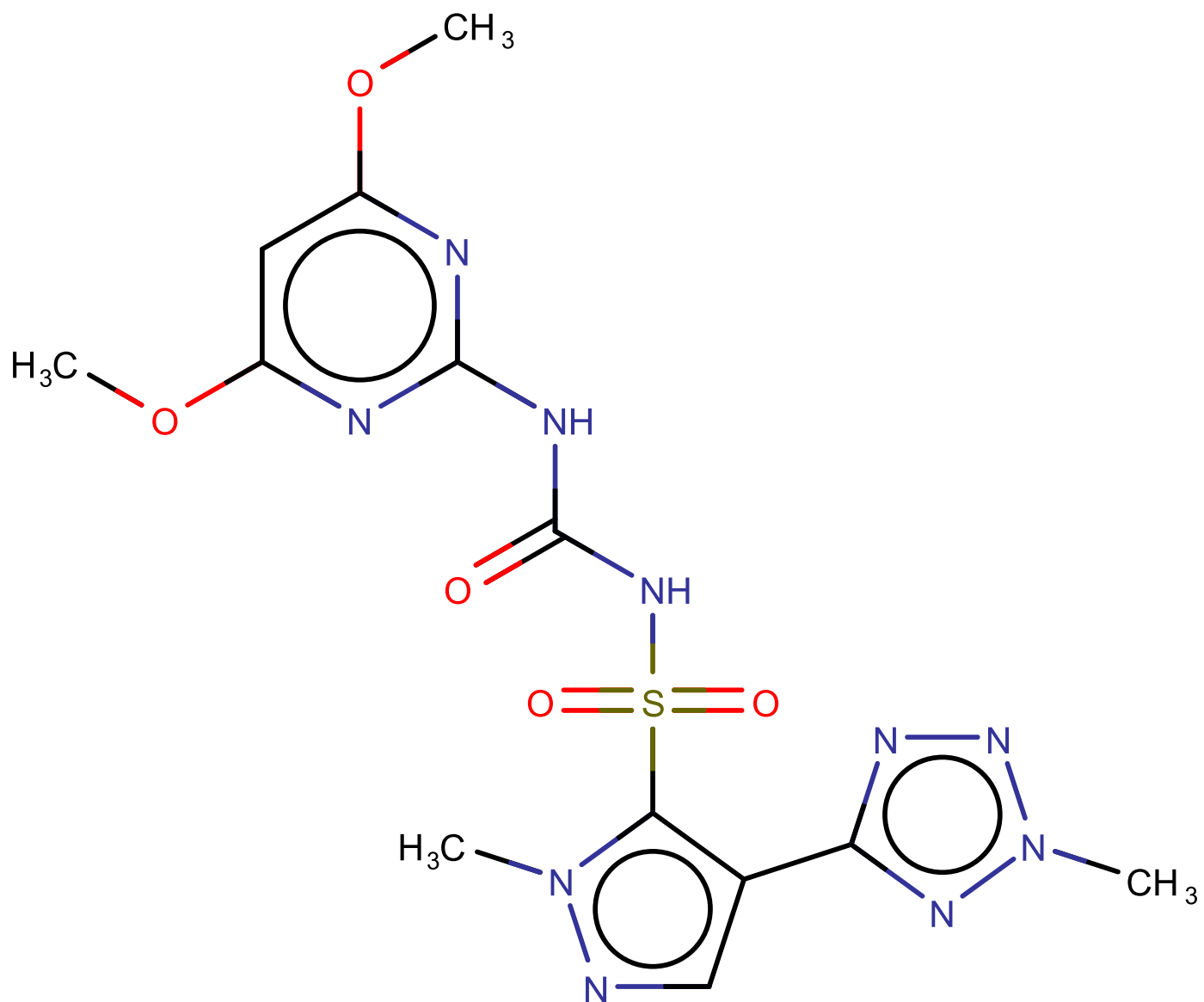

Supplement: Supplementary file 1 [file toxics-12-00425-s001.zip › Supplementary Materials/2D chemical structures/1818.pdf]

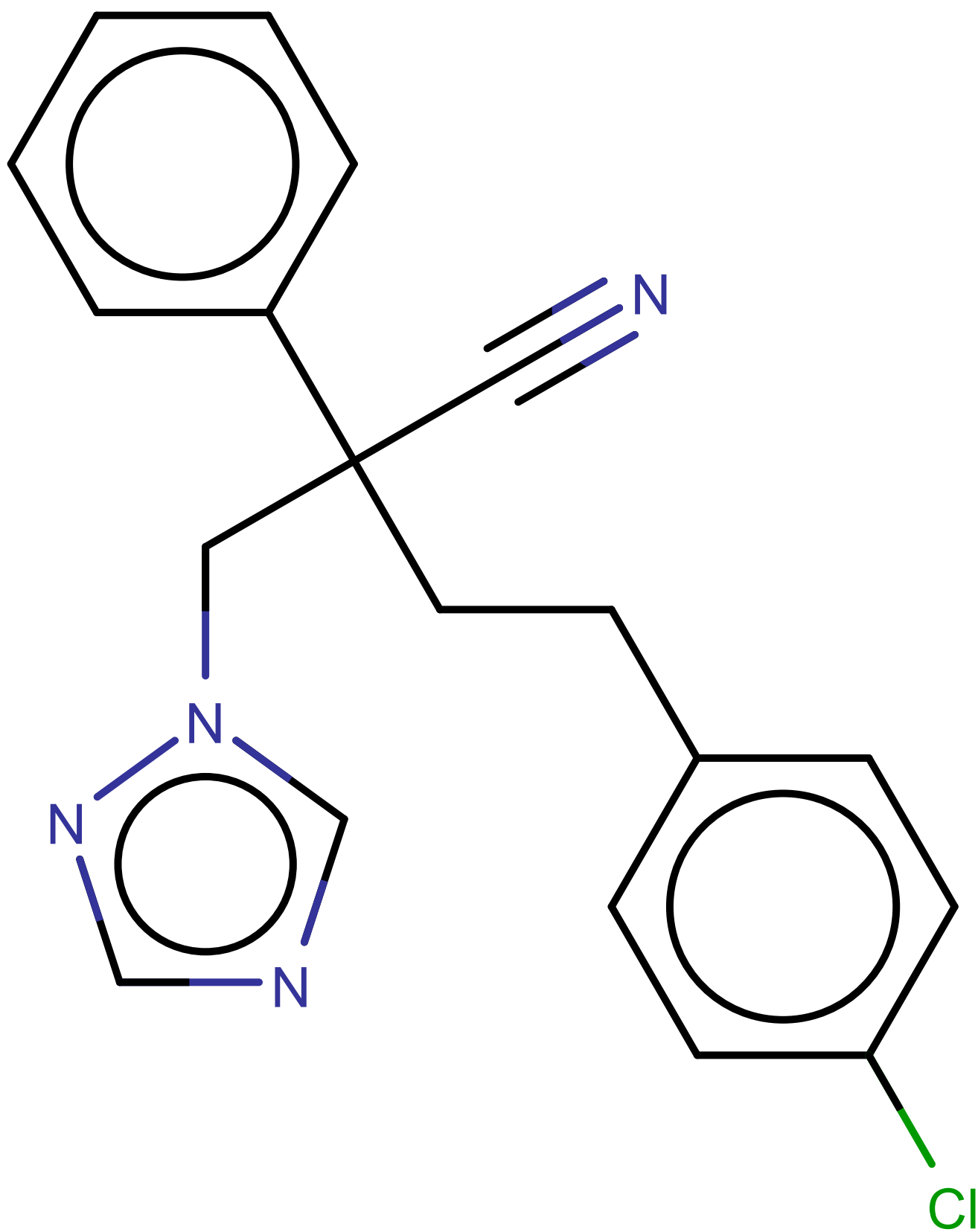

Supplement: Supplementary file 1 [file toxics-12-00425-s001.zip › Supplementary Materials/2D chemical structures/1820.pdf]

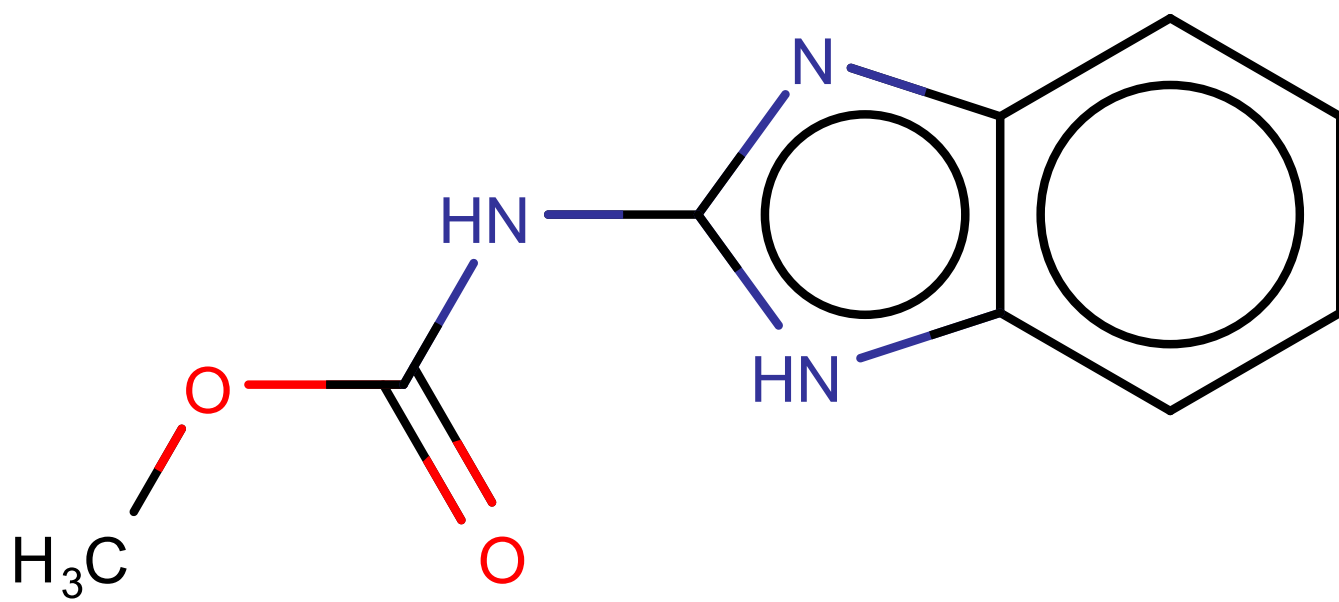

Supplement: Supplementary file 1 [file toxics-12-00425-s001.zip › Supplementary Materials/2D chemical structures/1822.pdf]

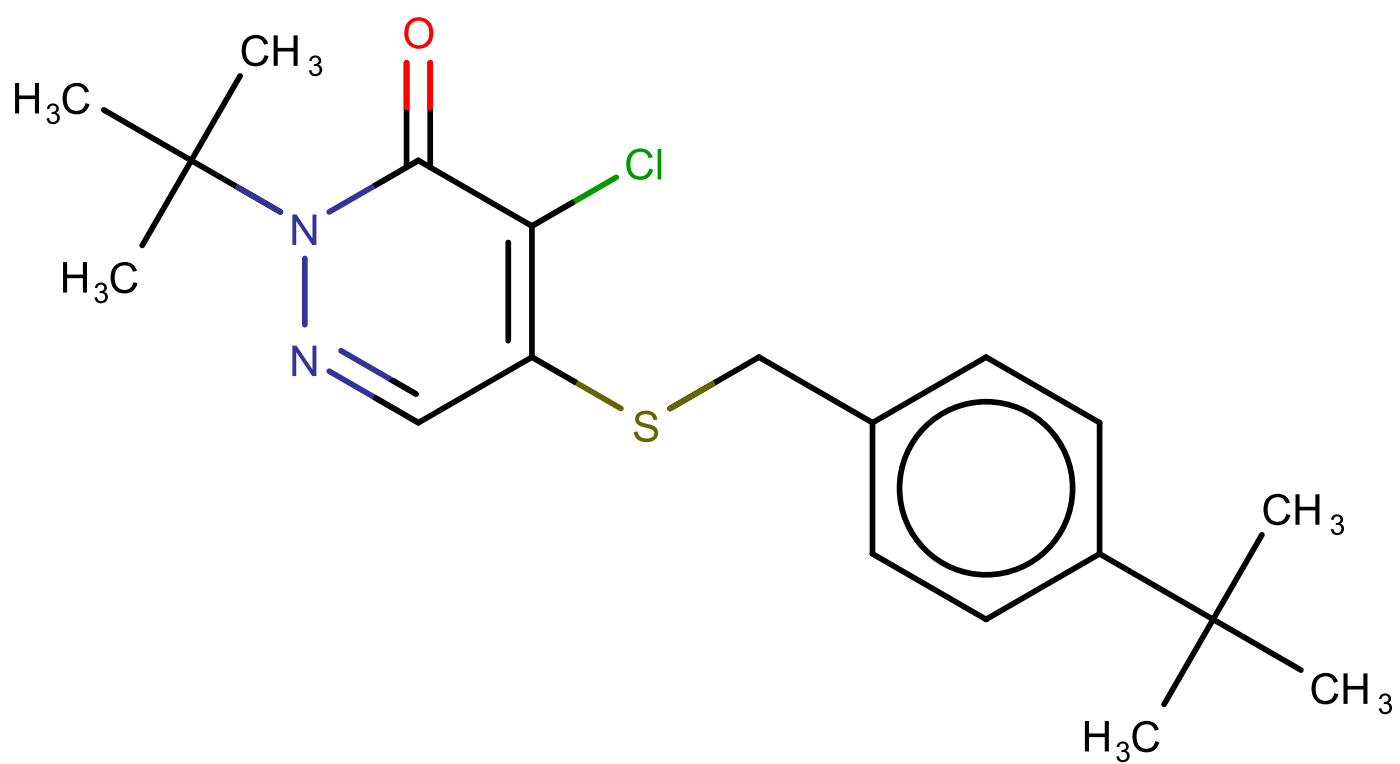

Supplement: Supplementary file 1 [file toxics-12-00425-s001.zip › Supplementary Materials/2D chemical structures/1824.pdf]

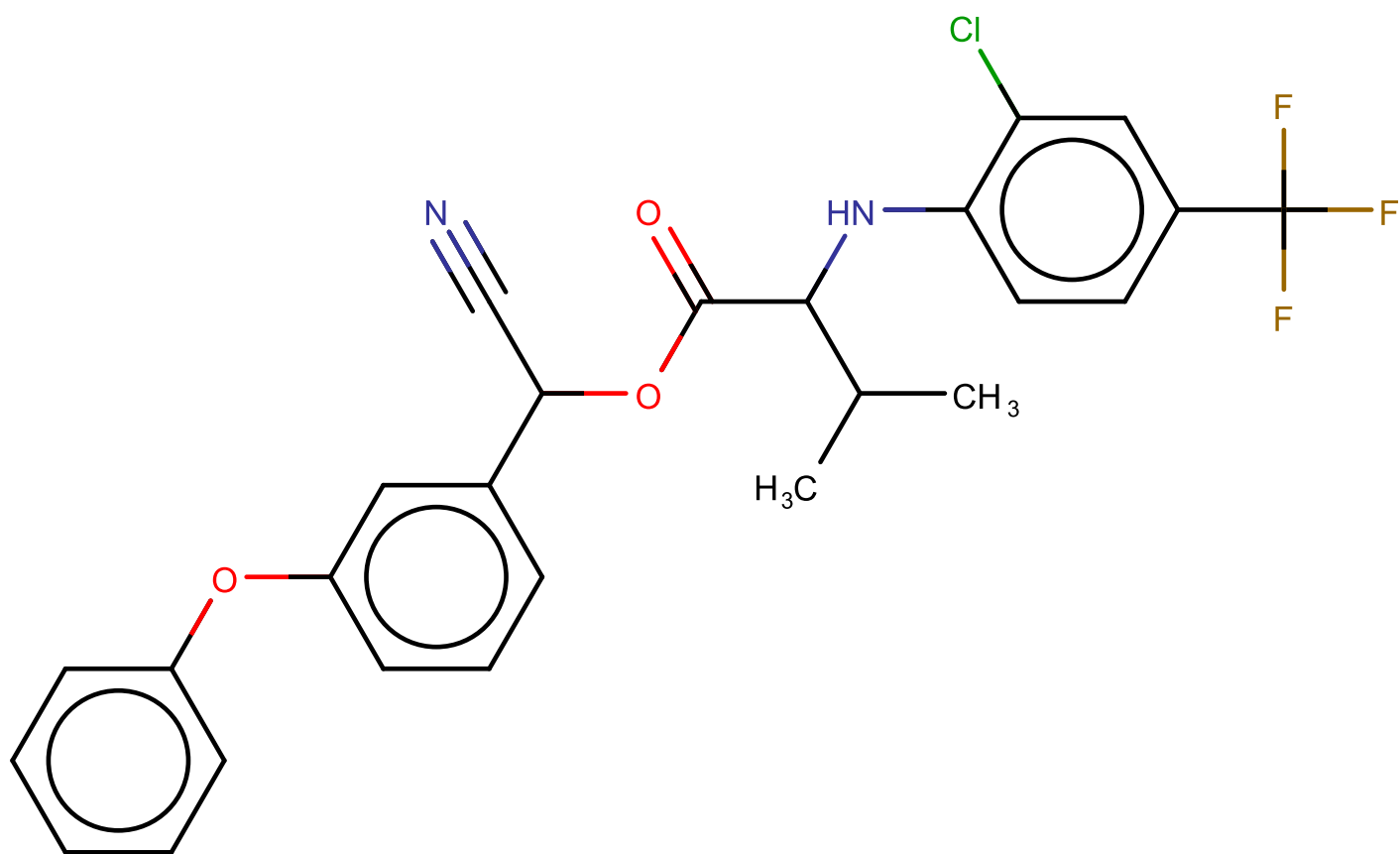

Supplement: Supplementary file 1 [file toxics-12-00425-s001.zip › Supplementary Materials/2D chemical structures/1825.pdf]

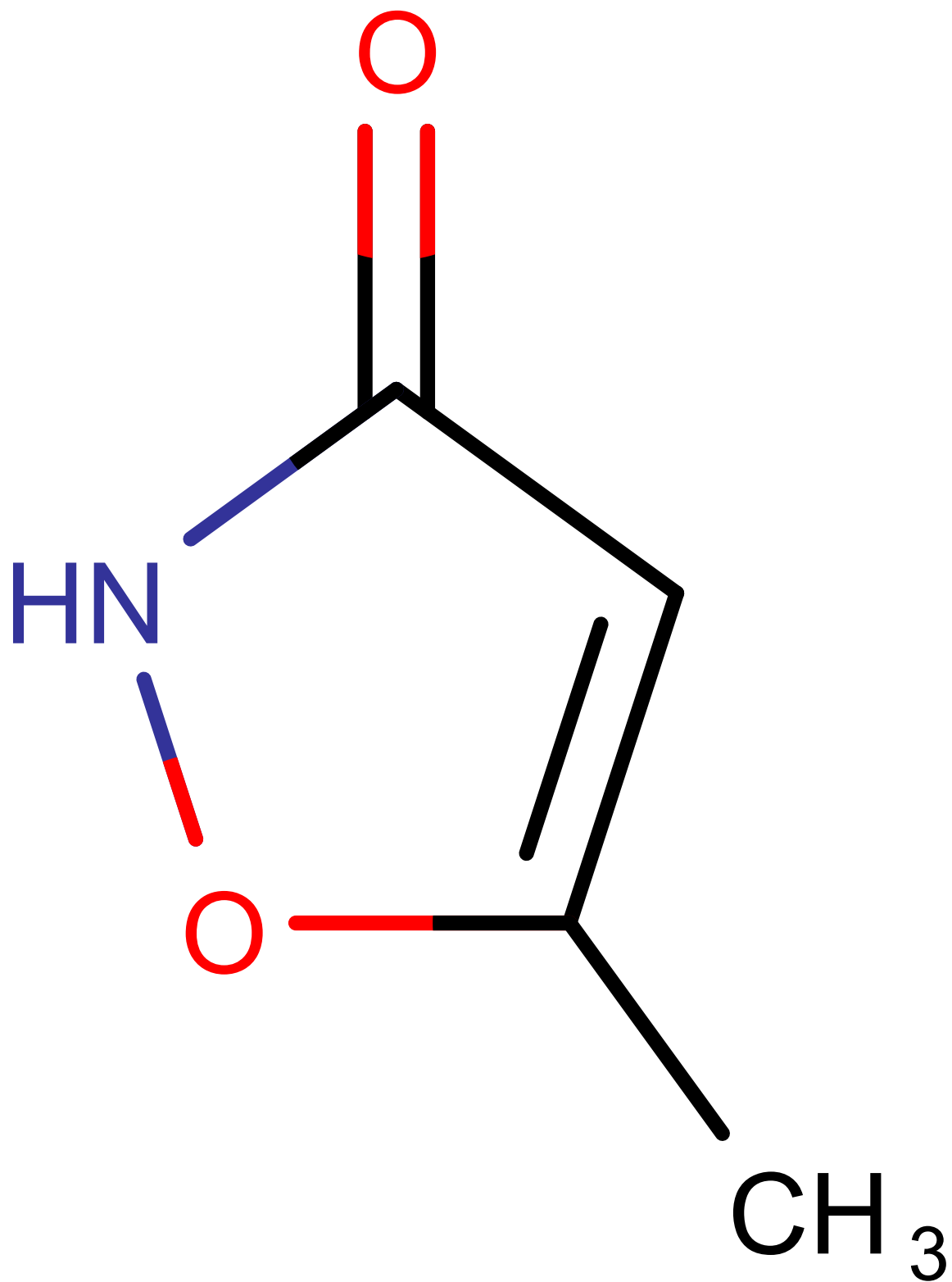

Supplement: Supplementary file 1 [file toxics-12-00425-s001.zip › Supplementary Materials/2D chemical structures/1826.pdf]

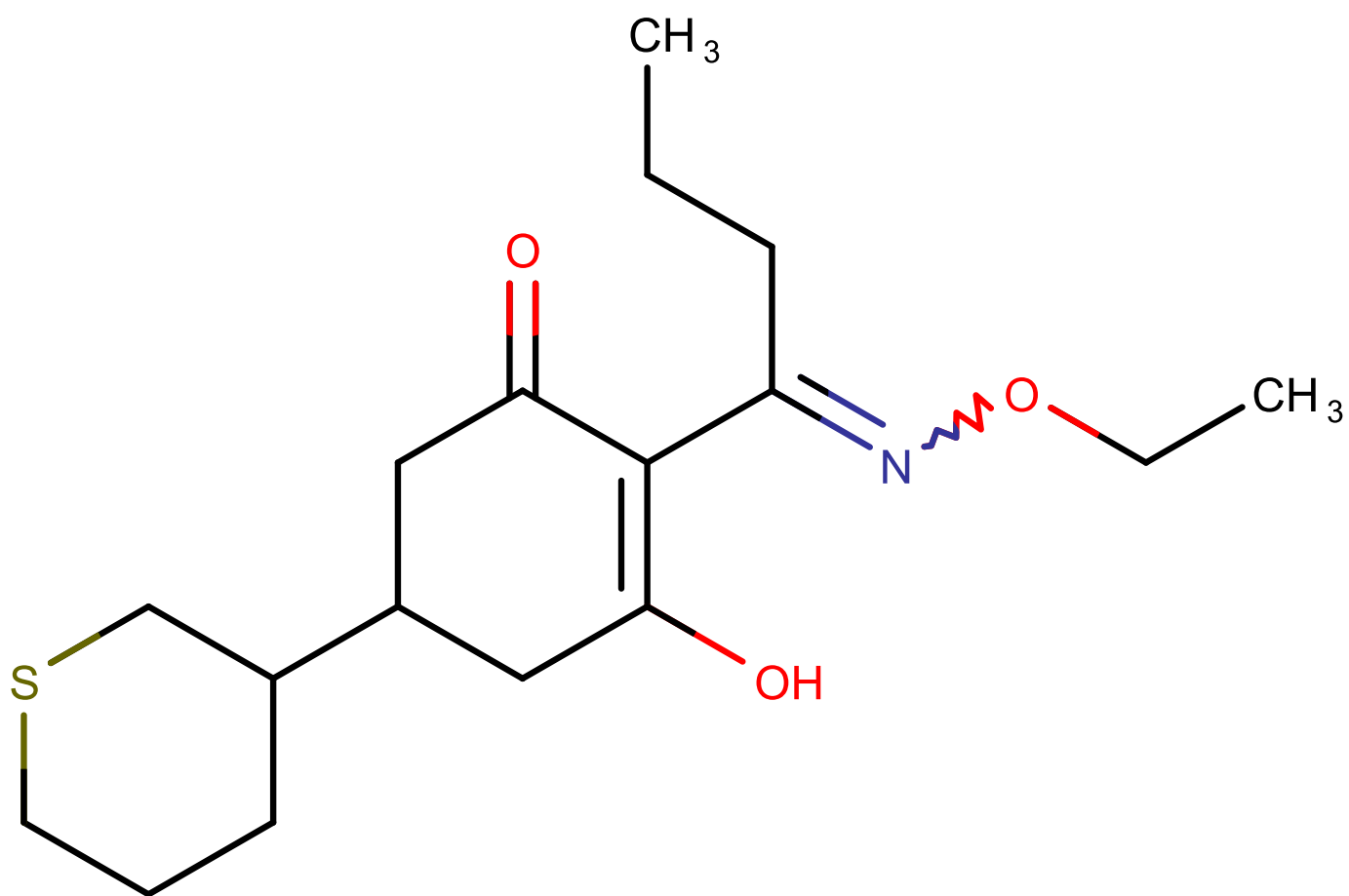

Supplement: Supplementary file 1 [file toxics-12-00425-s001.zip › Supplementary Materials/2D chemical structures/1827.pdf]

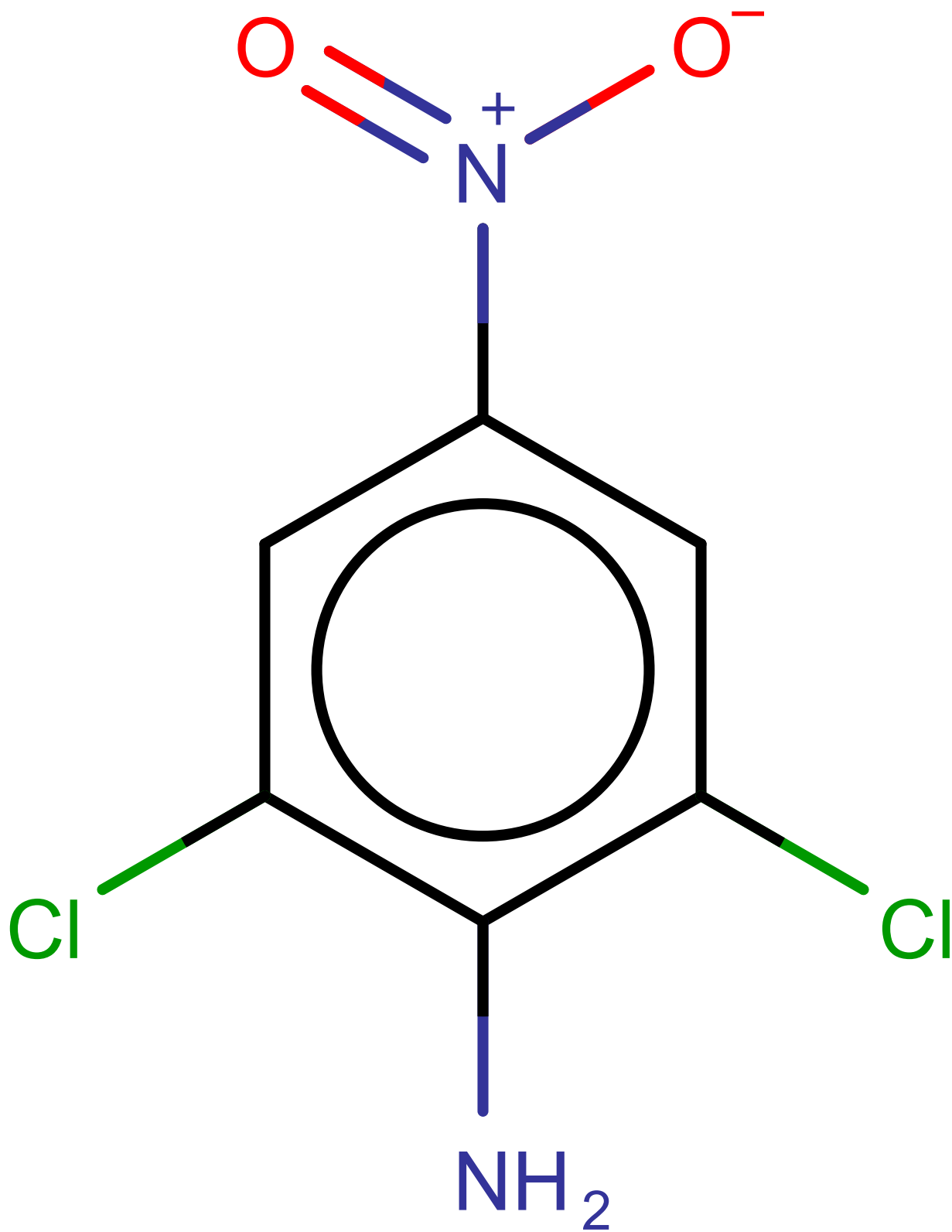

Supplement: Supplementary file 1 [file toxics-12-00425-s001.zip › Supplementary Materials/2D chemical structures/1830.pdf]

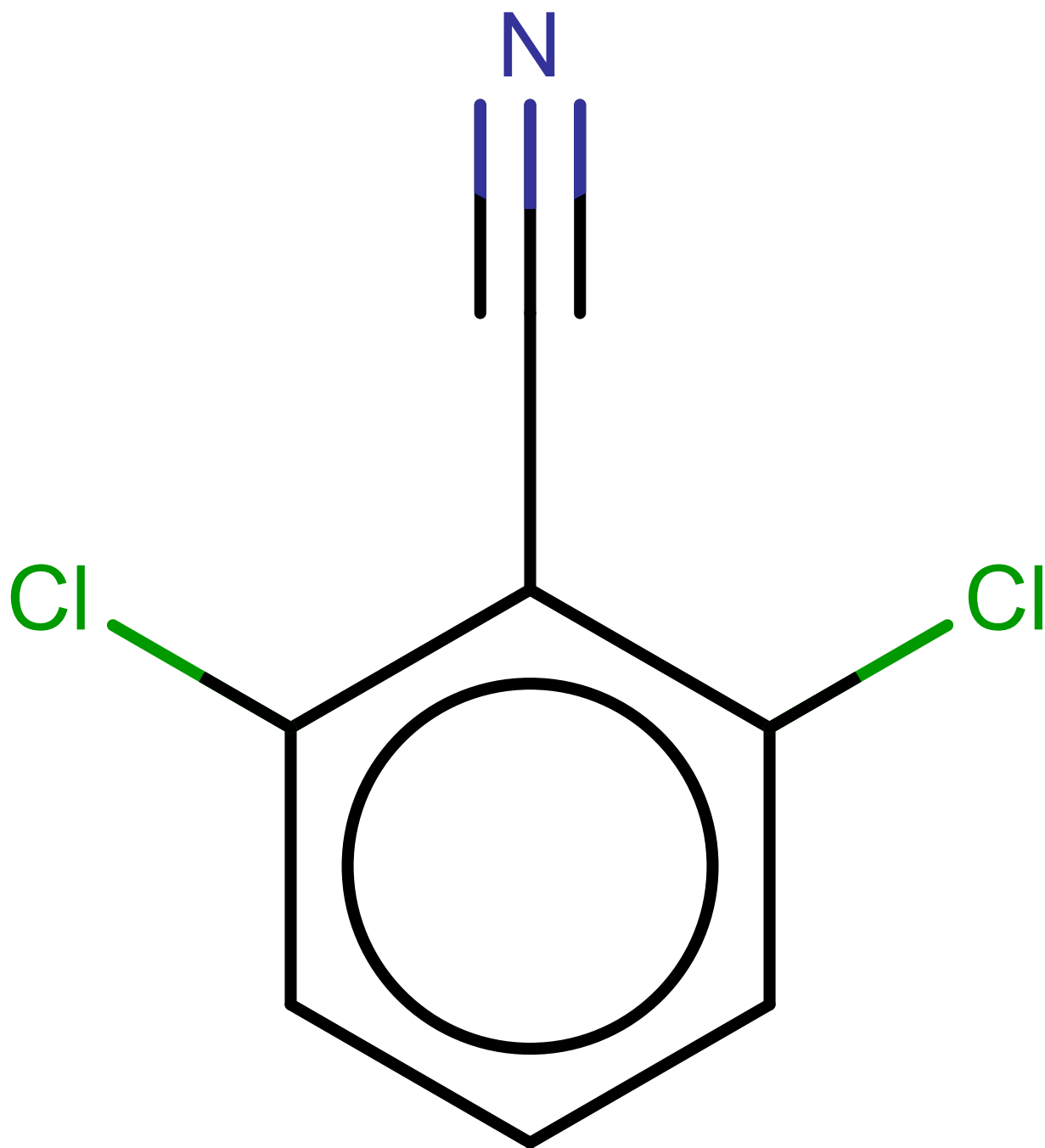

Supplement: Supplementary file 1 [file toxics-12-00425-s001.zip › Supplementary Materials/2D chemical structures/1831.pdf]

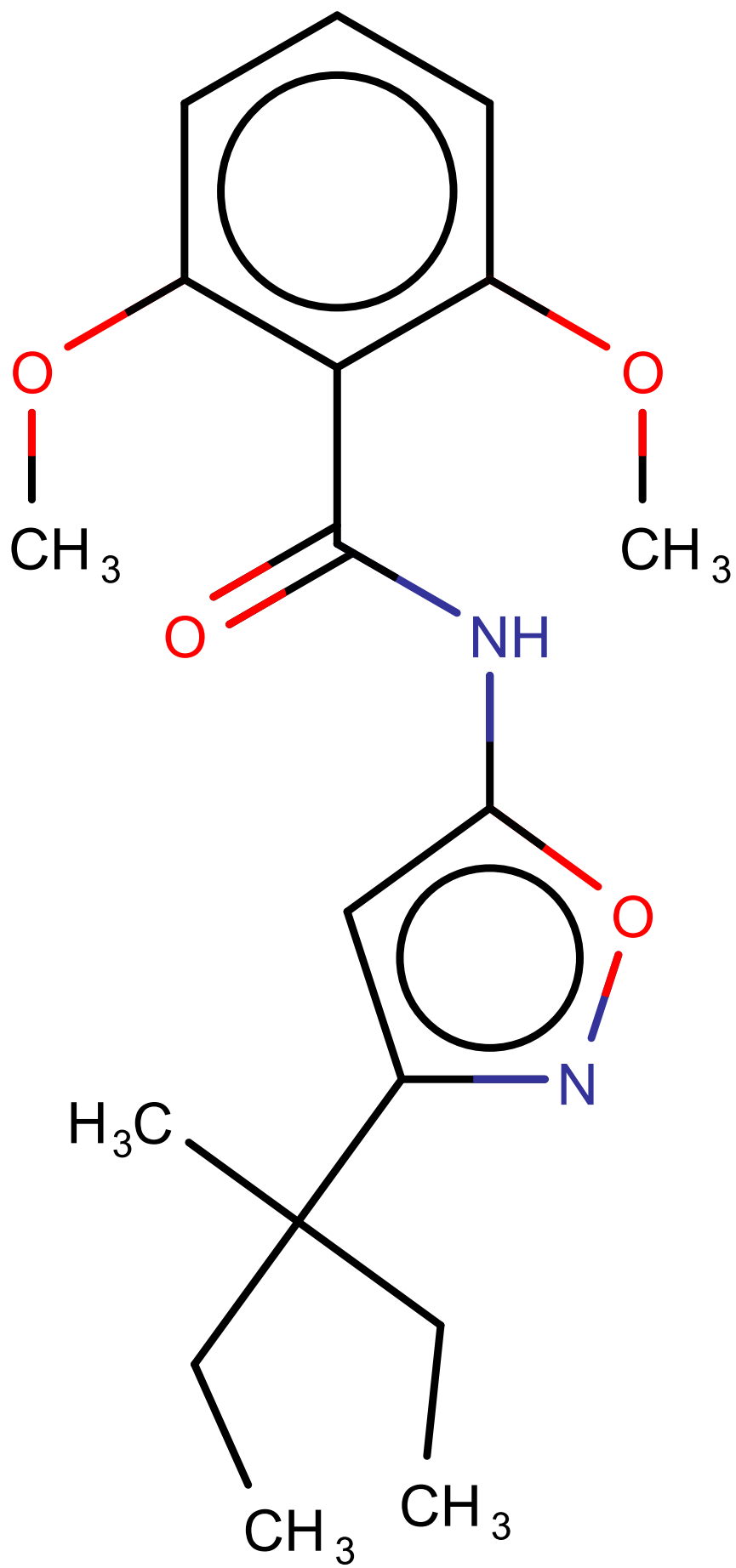

Supplement: Supplementary file 1 [file toxics-12-00425-s001.zip › Supplementary Materials/2D chemical structures/1834.pdf]

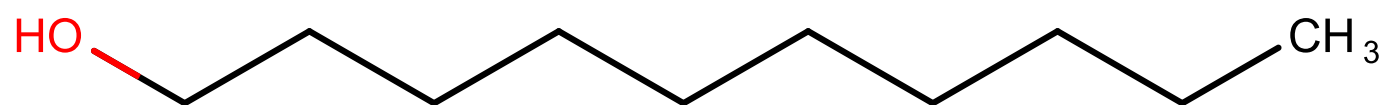

Supplement: Supplementary file 1 [file toxics-12-00425-s001.zip › Supplementary Materials/2D chemical structures/1835.pdf]

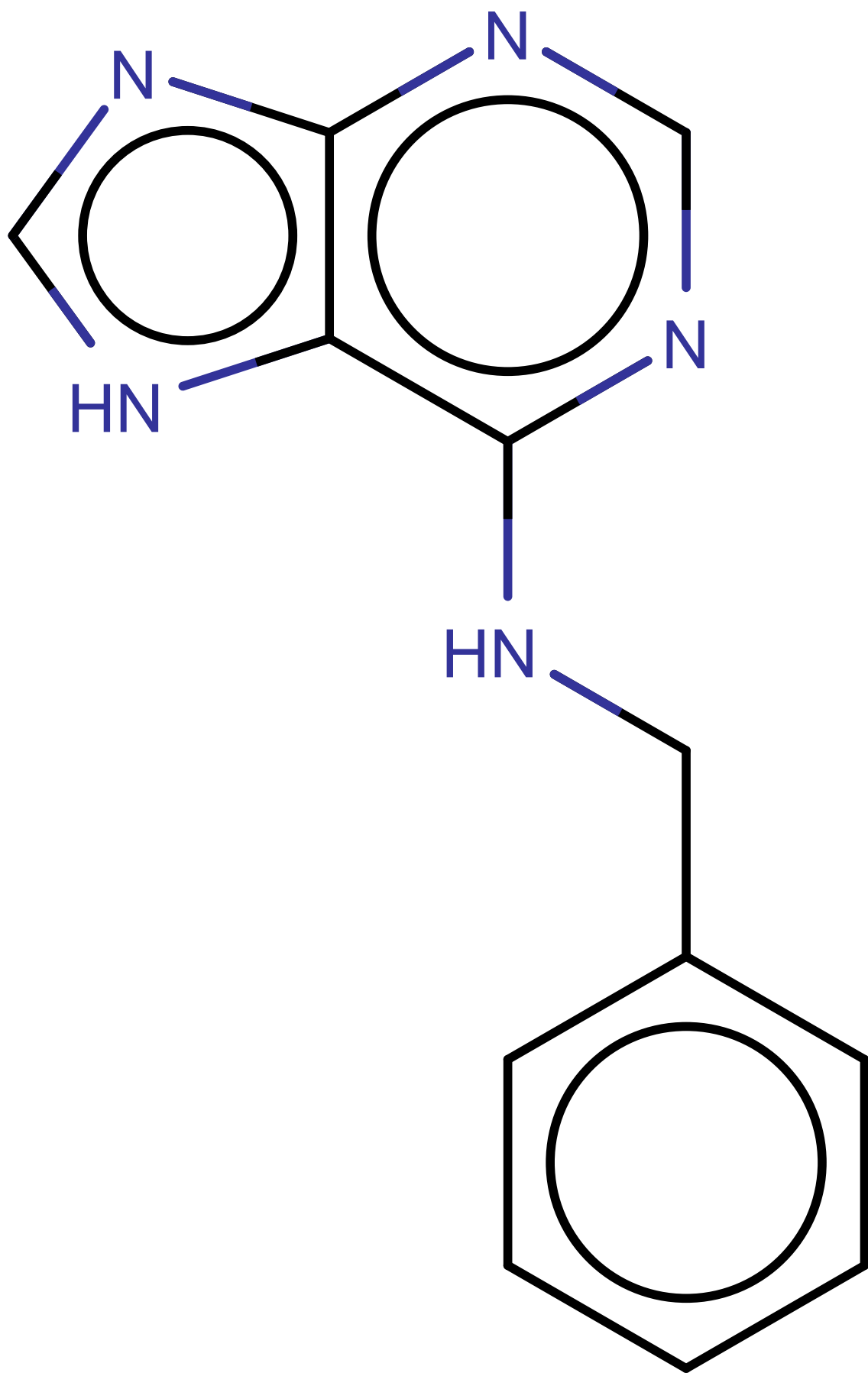

Supplement: Supplementary file 1 [file toxics-12-00425-s001.zip › Supplementary Materials/2D chemical structures/1836.pdf]

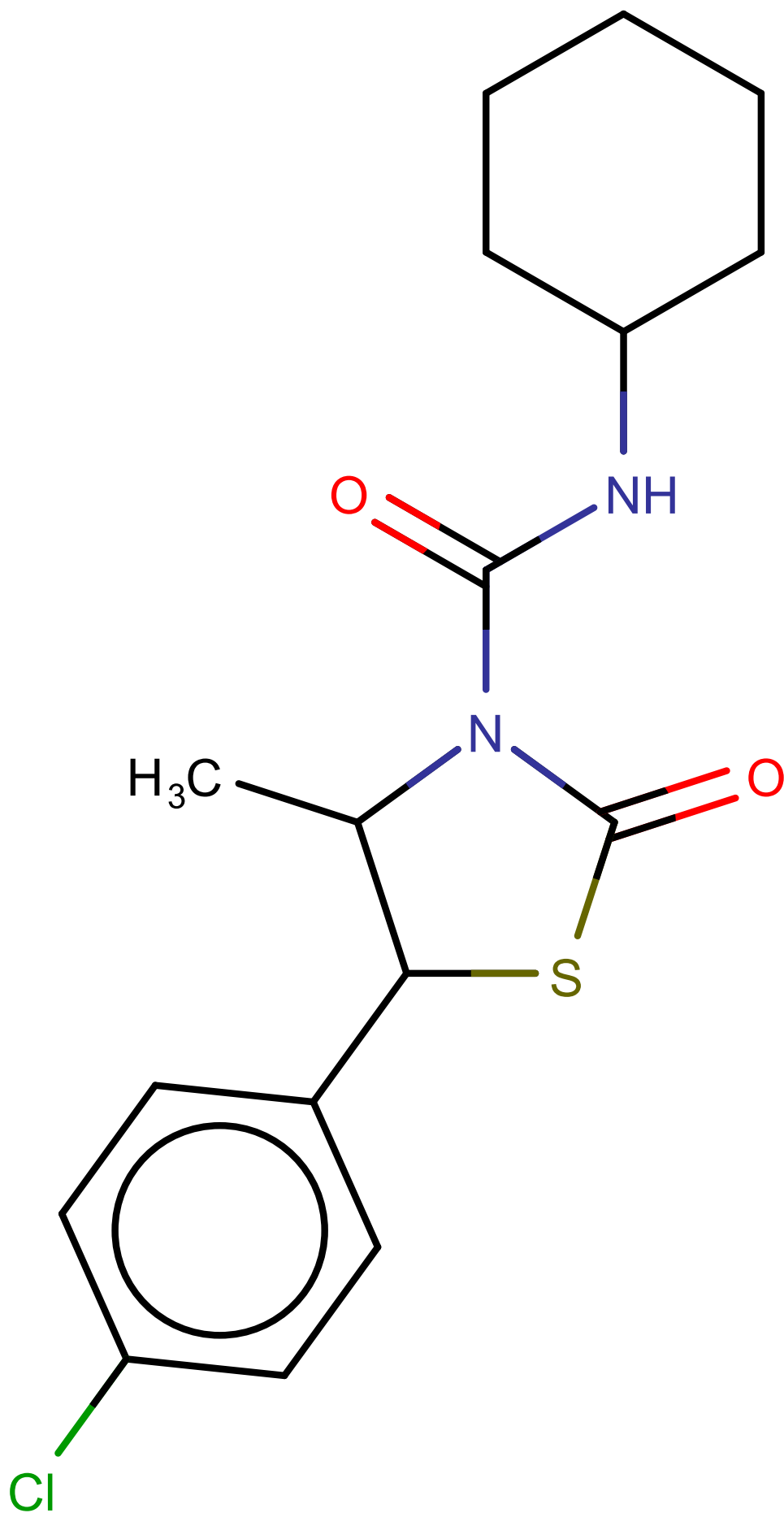

Supplement: Supplementary file 1 [file toxics-12-00425-s001.zip › Supplementary Materials/2D chemical structures/1840.pdf]

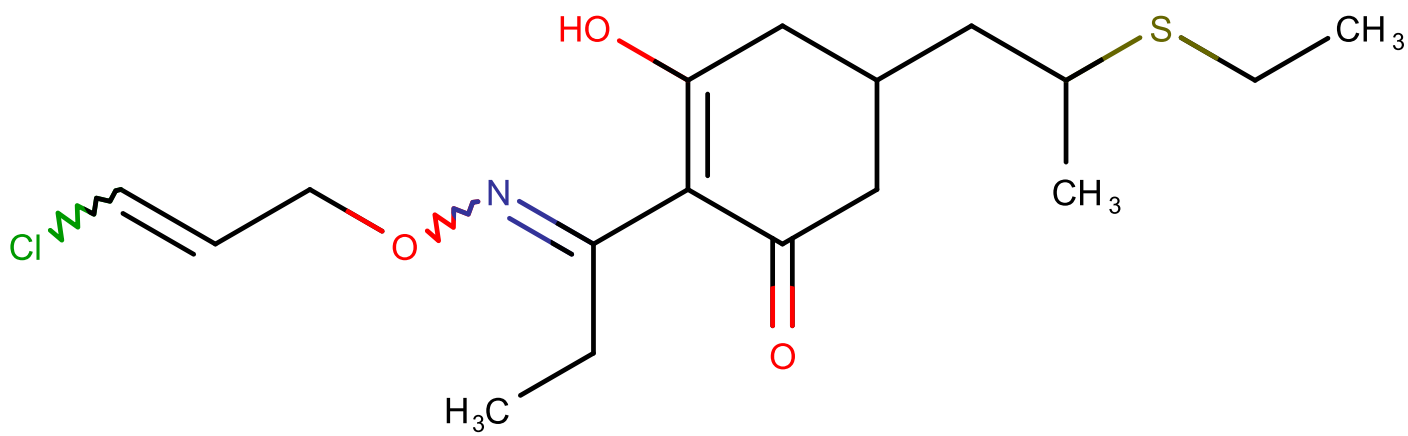

Supplement: Supplementary file 1 [file toxics-12-00425-s001.zip › Supplementary Materials/2D chemical structures/1842.pdf]

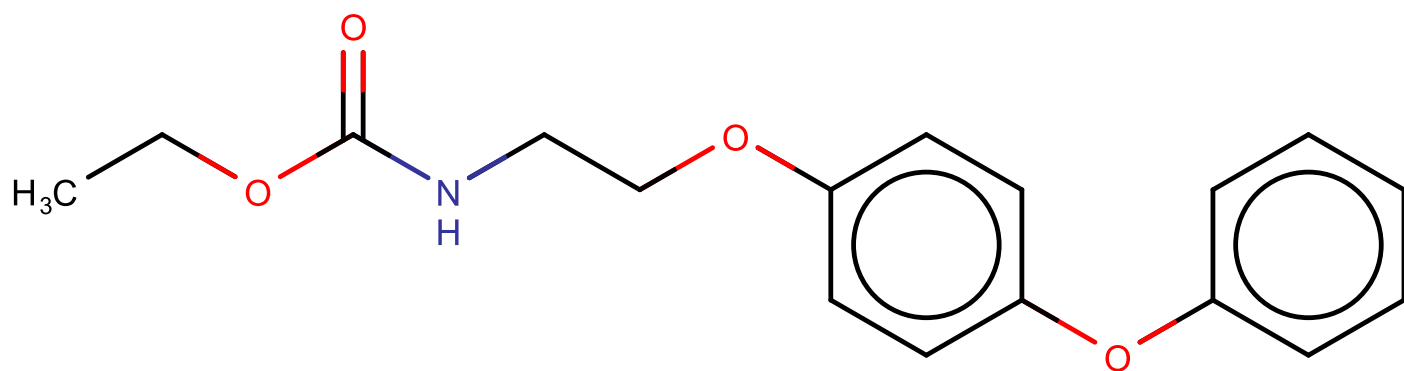

Supplement: Supplementary file 1 [file toxics-12-00425-s001.zip › Supplementary Materials/2D chemical structures/1843.pdf]

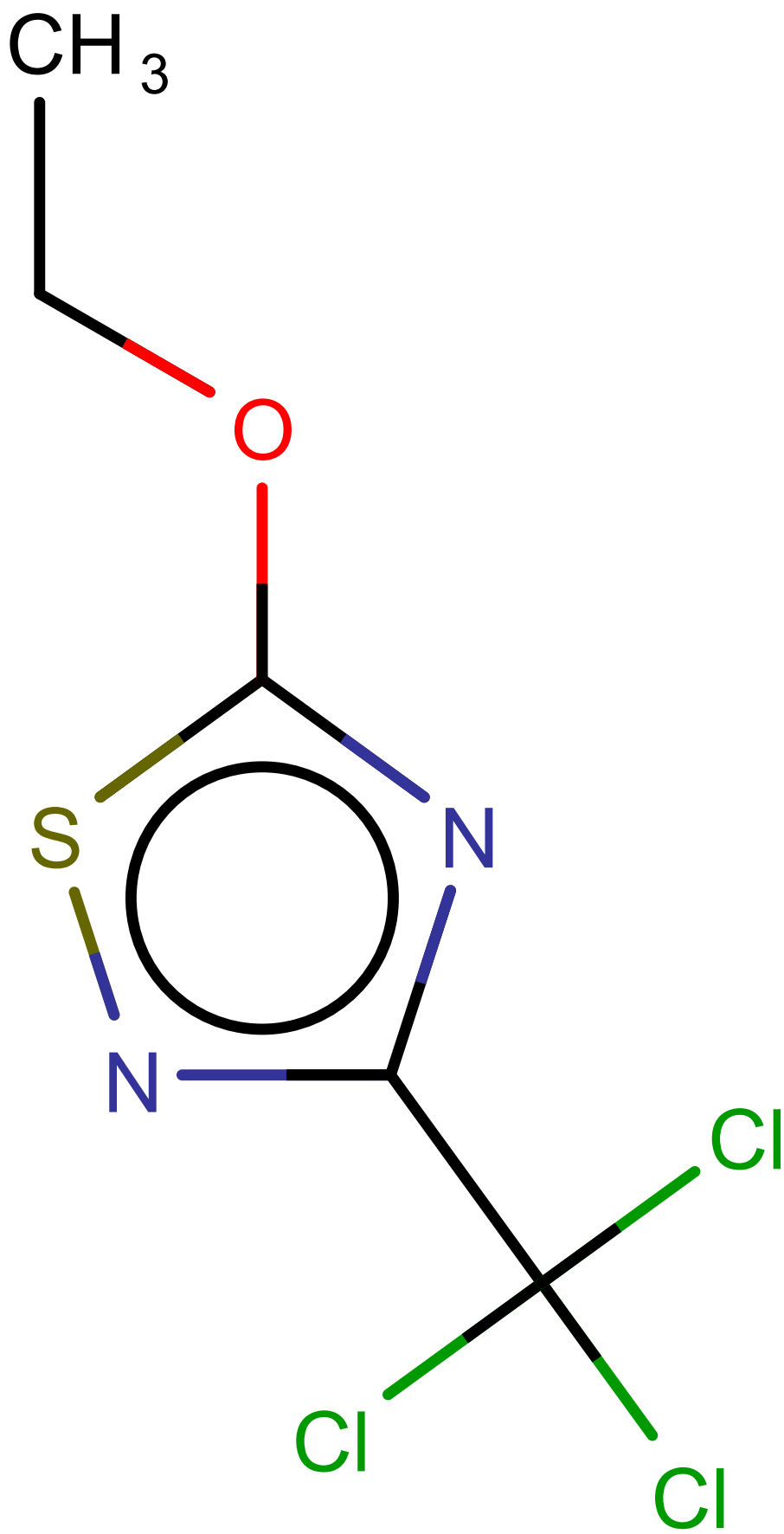

Supplement: Supplementary file 1 [file toxics-12-00425-s001.zip › Supplementary Materials/2D chemical structures/1867.pdf]

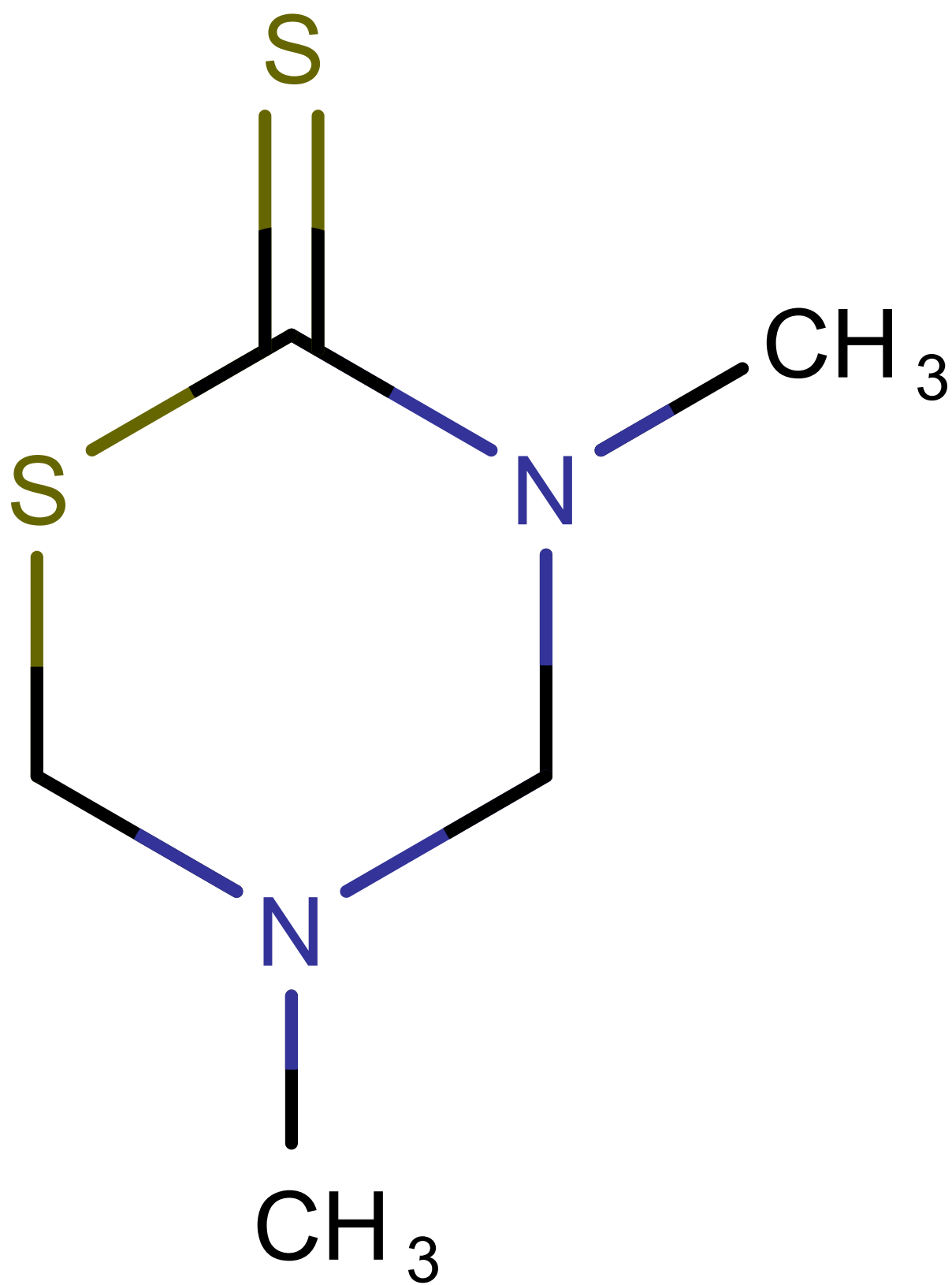

Supplement: Supplementary file 1 [file toxics-12-00425-s001.zip › Supplementary Materials/2D chemical structures/1870.pdf]

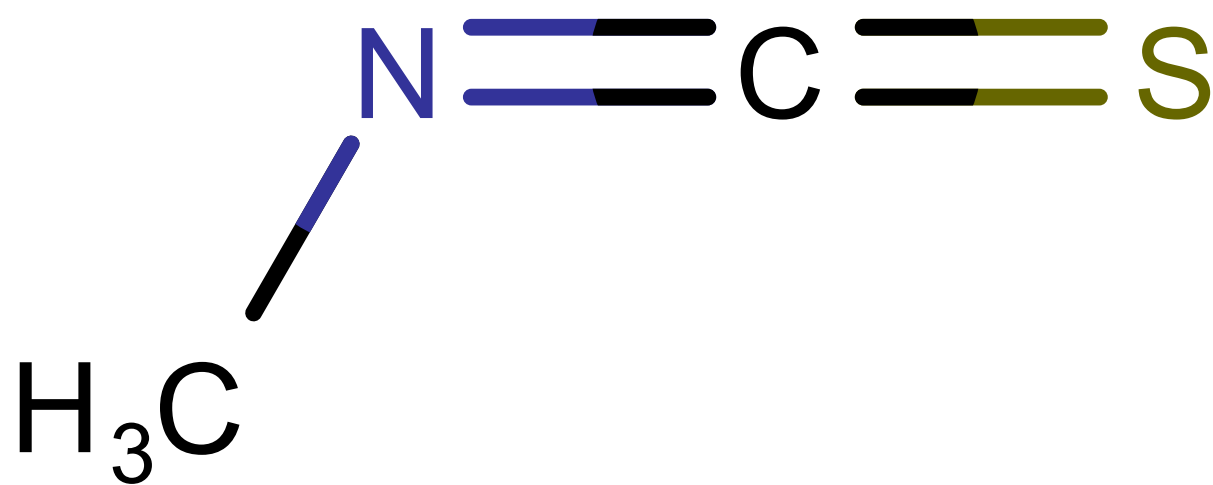

Supplement: Supplementary file 1 [file toxics-12-00425-s001.zip › Supplementary Materials/2D chemical structures/1872.pdf]

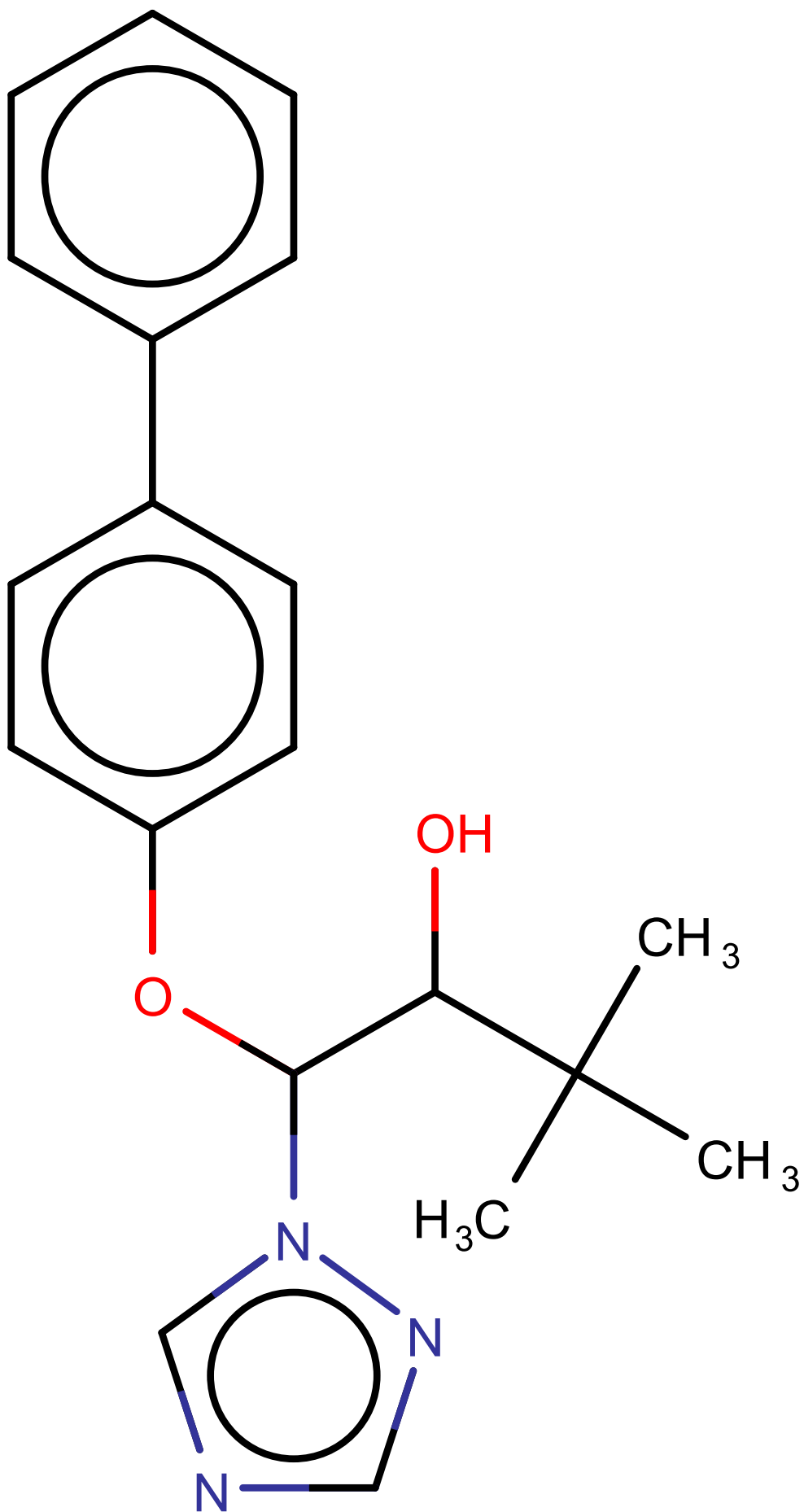

Supplement: Supplementary file 1 [file toxics-12-00425-s001.zip › Supplementary Materials/2D chemical structures/1873.pdf]

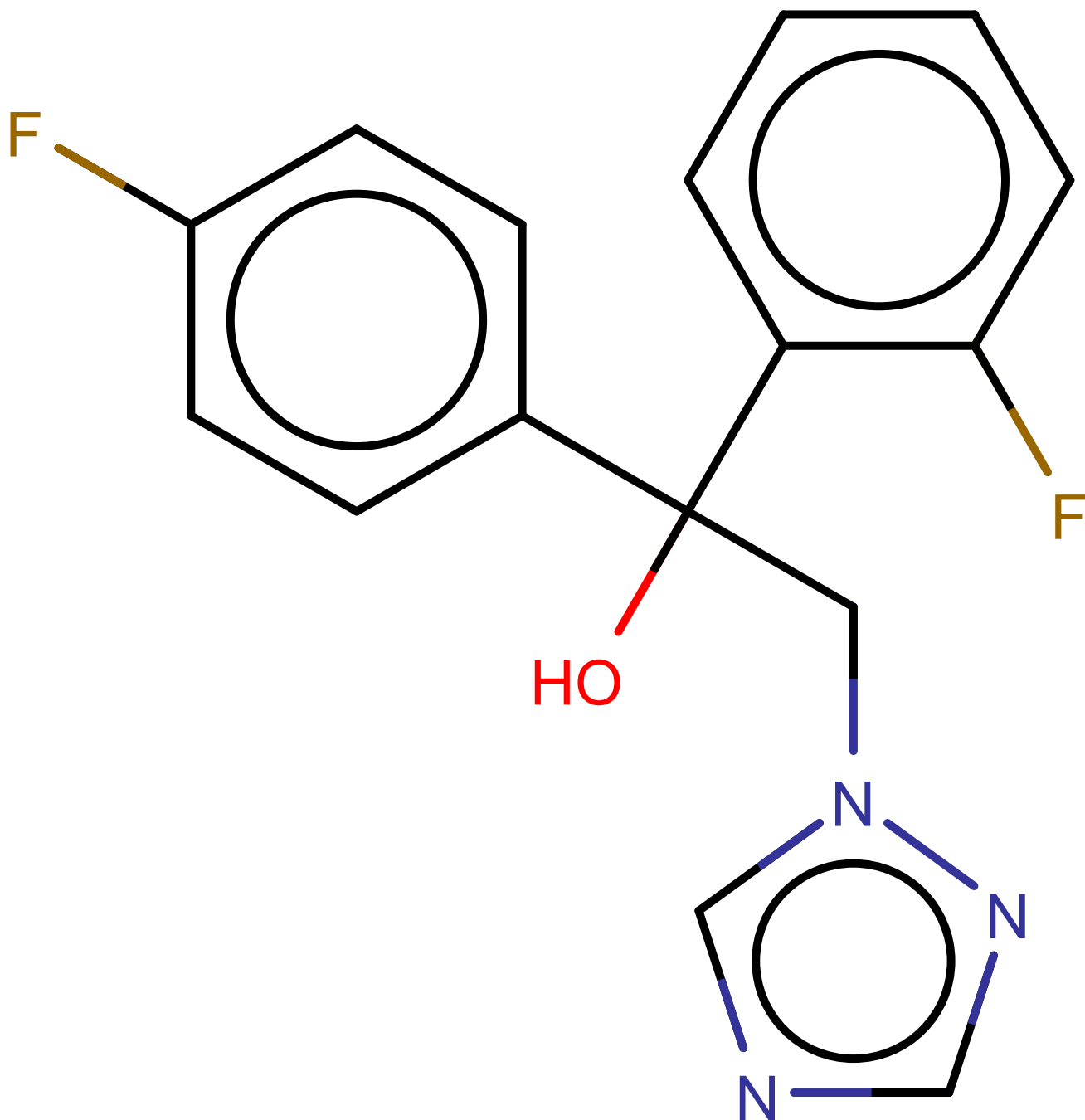

Supplement: Supplementary file 1 [file toxics-12-00425-s001.zip › Supplementary Materials/2D chemical structures/1881.pdf]

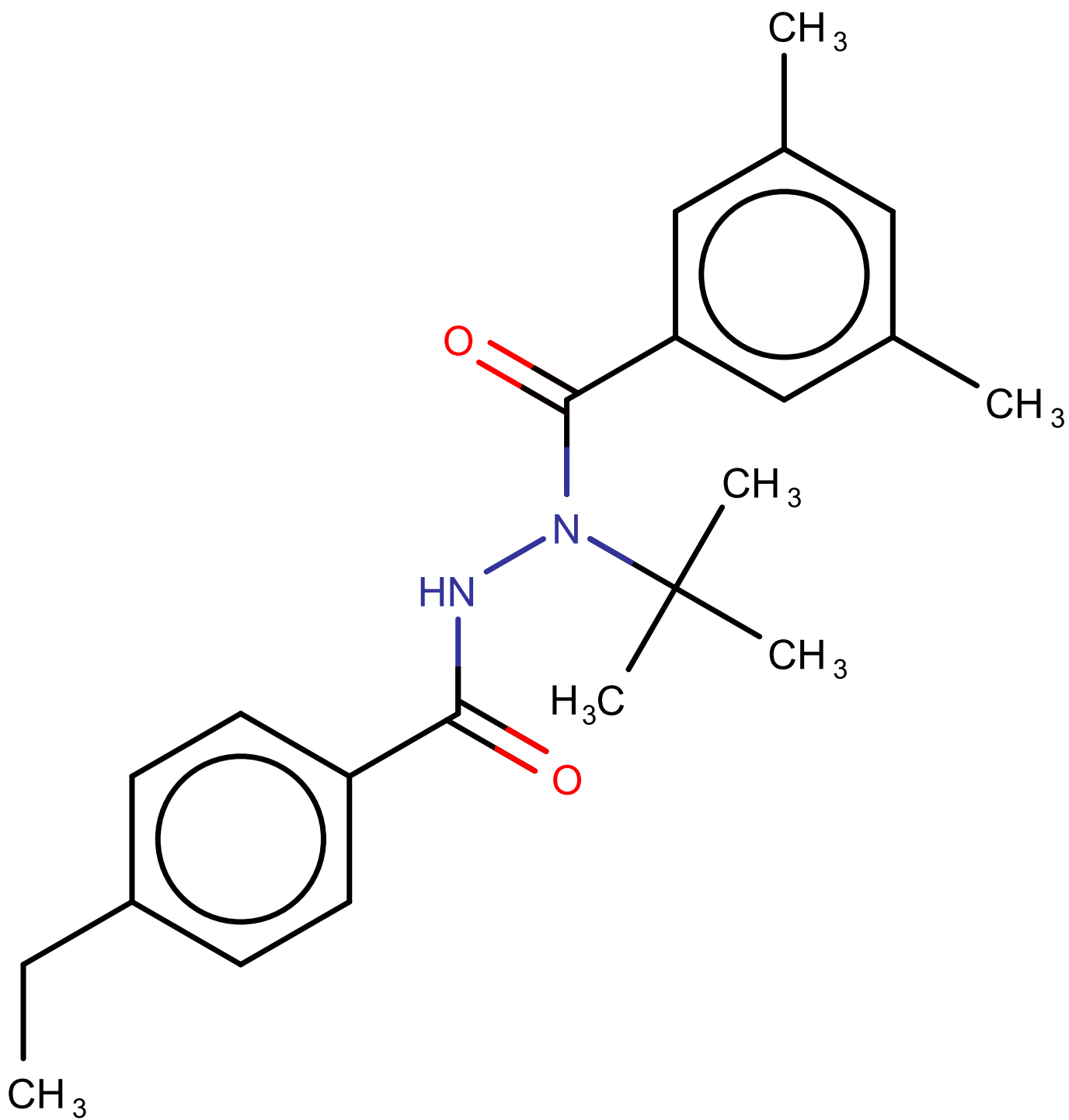

Supplement: Supplementary file 1 [file toxics-12-00425-s001.zip › Supplementary Materials/2D chemical structures/1885.pdf]

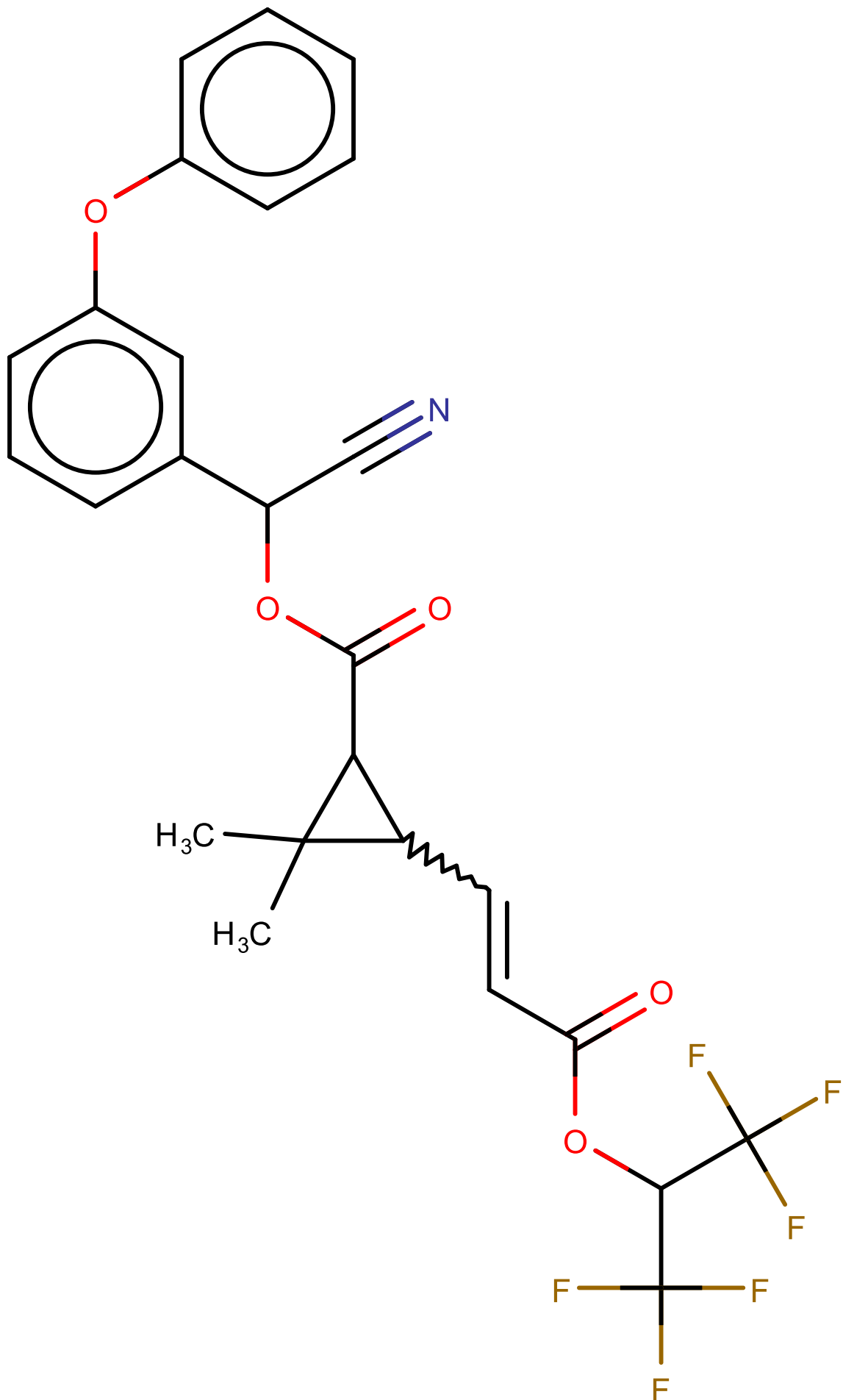

Supplement: Supplementary file 1 [file toxics-12-00425-s001.zip › Supplementary Materials/2D chemical structures/1886.pdf]

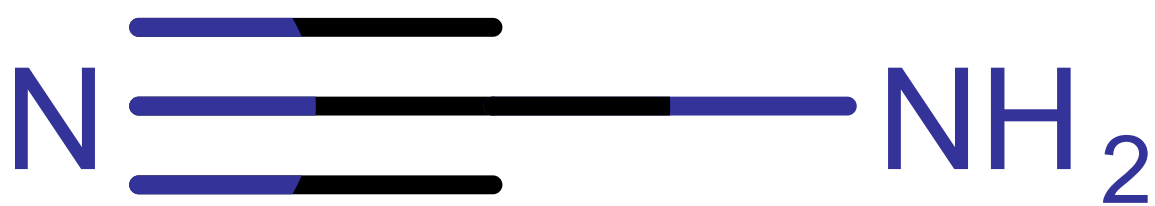

Supplement: Supplementary file 1 [file toxics-12-00425-s001.zip › Supplementary Materials/2D chemical structures/1888.pdf]

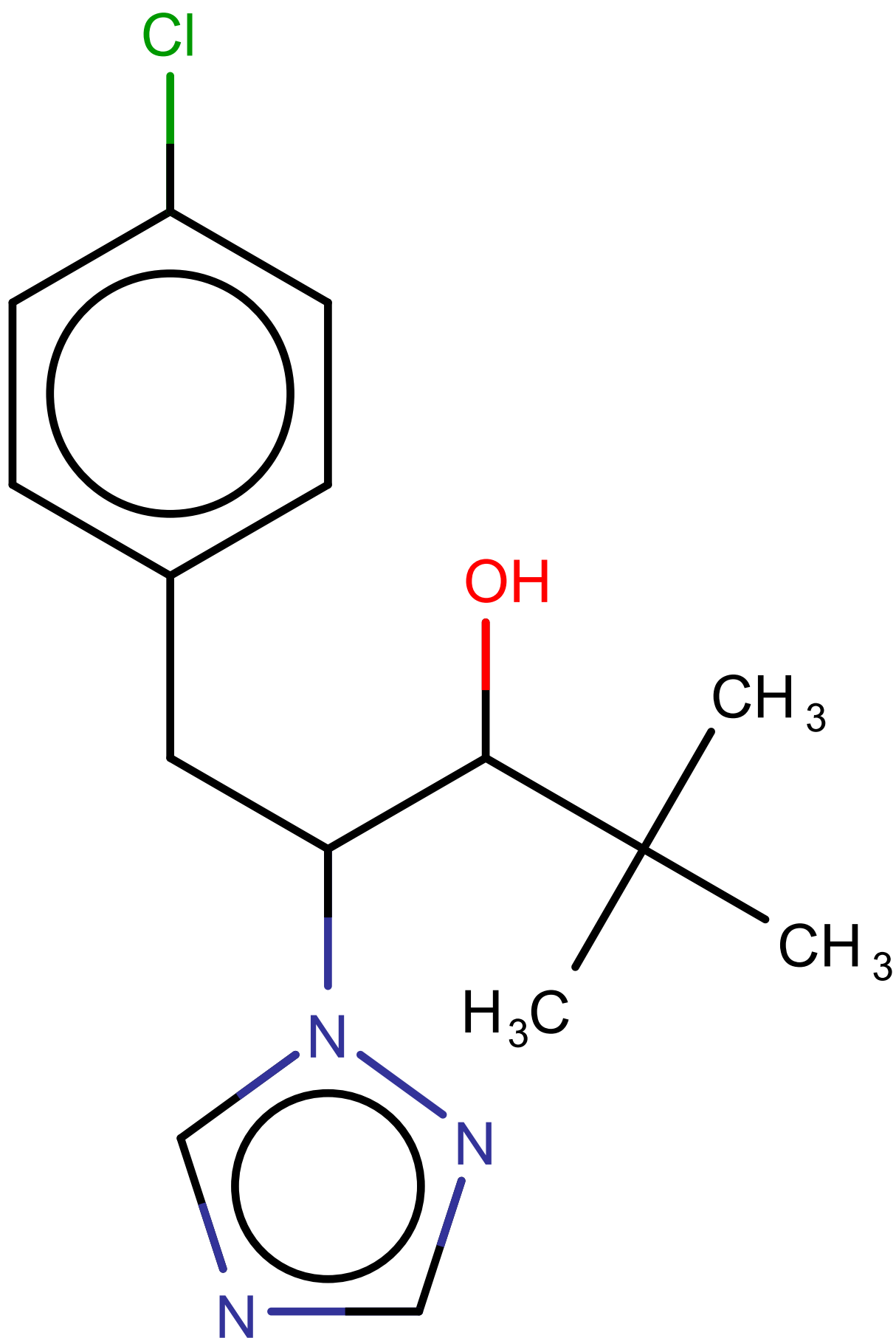

Supplement: Supplementary file 1 [file toxics-12-00425-s001.zip › Supplementary Materials/2D chemical structures/1891.pdf]

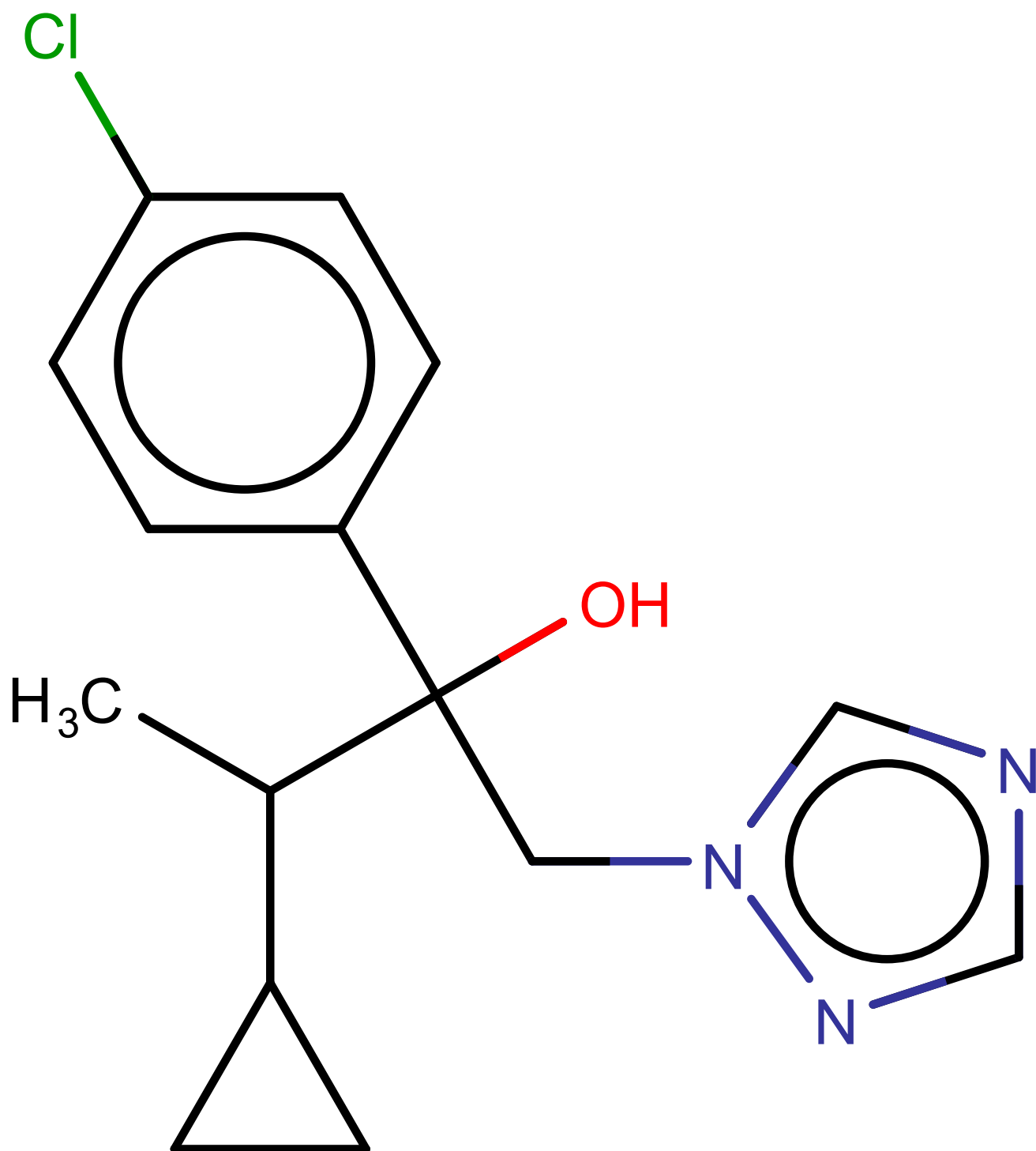

Supplement: Supplementary file 1 [file toxics-12-00425-s001.zip › Supplementary Materials/2D chemical structures/1898.pdf]

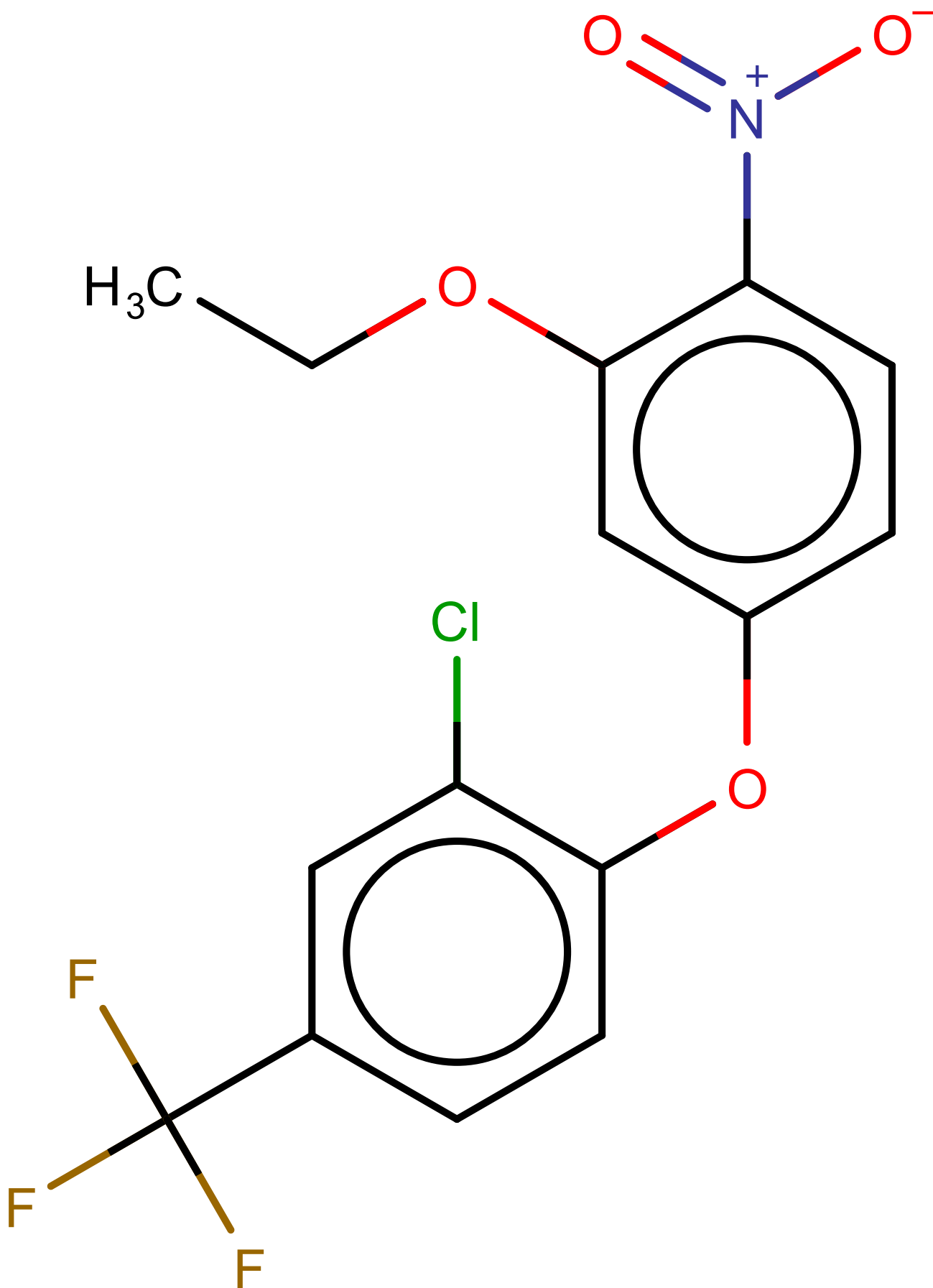

Supplement: Supplementary file 1 [file toxics-12-00425-s001.zip › Supplementary Materials/2D chemical structures/1907.pdf]

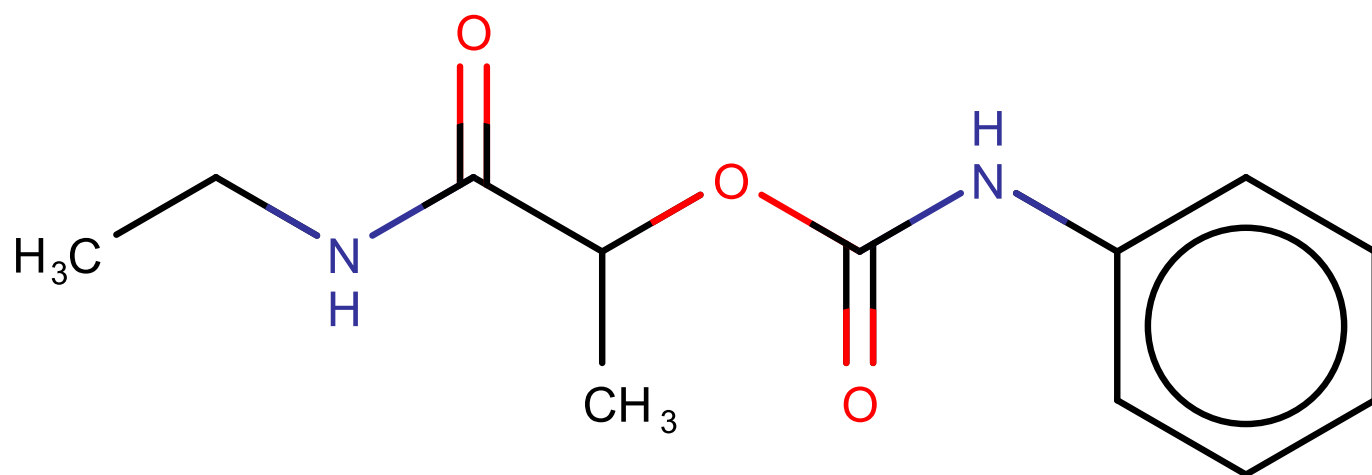

Supplement: Supplementary file 1 [file toxics-12-00425-s001.zip › Supplementary Materials/2D chemical structures/1909.pdf]

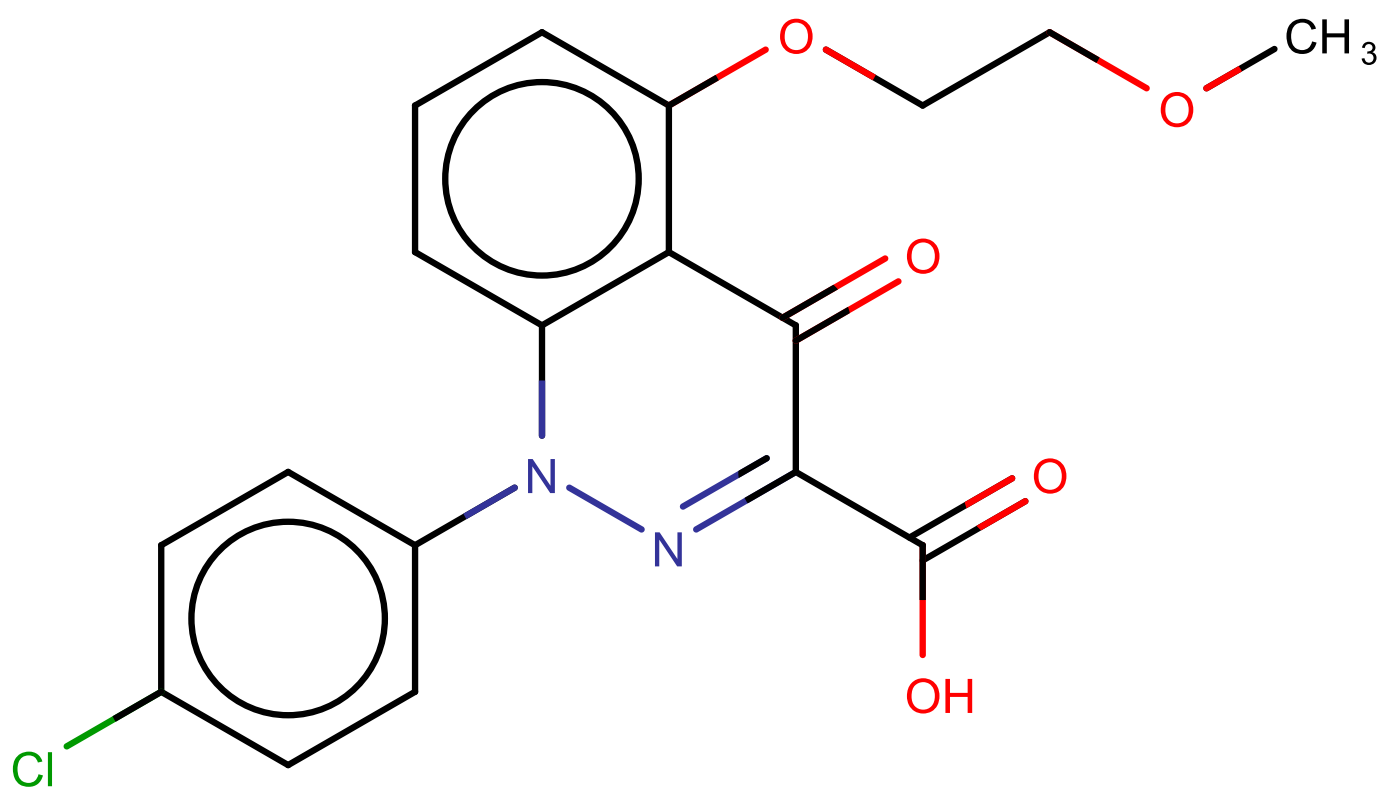

Supplement: Supplementary file 1 [file toxics-12-00425-s001.zip › Supplementary Materials/2D chemical structures/1911.pdf]

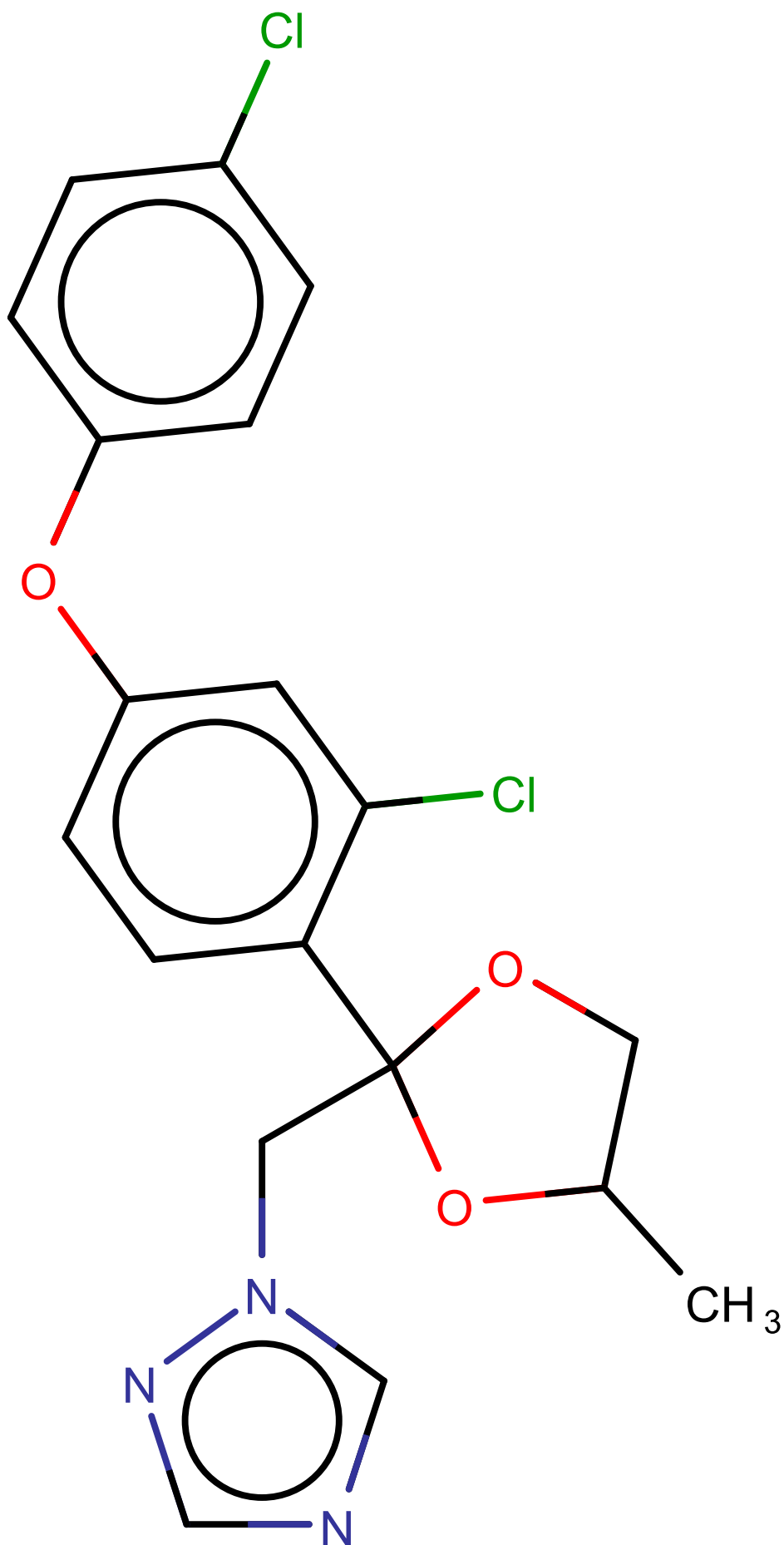

Supplement: Supplementary file 1 [file toxics-12-00425-s001.zip › Supplementary Materials/2D chemical structures/1929.pdf]

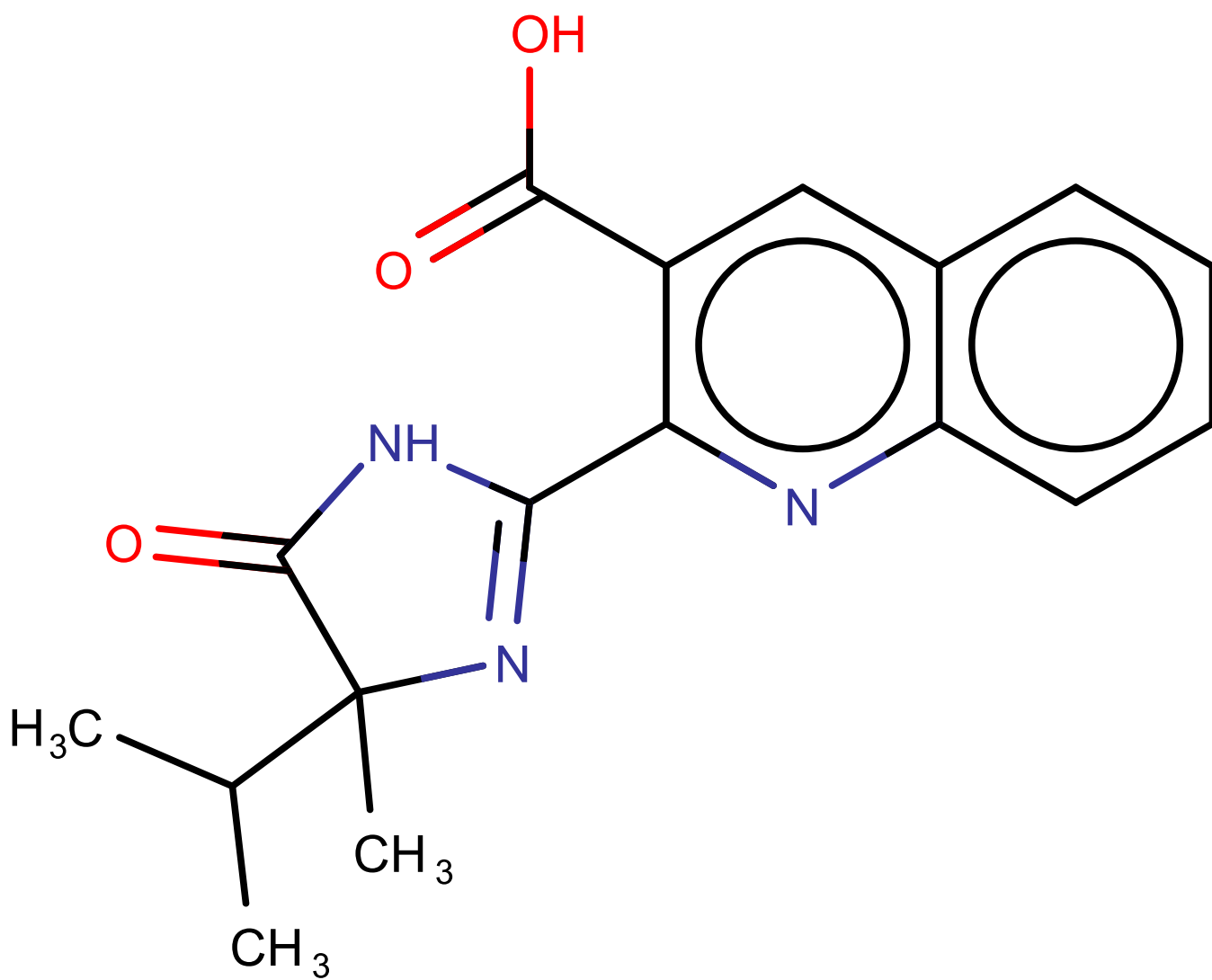

Supplement: Supplementary file 1 [file toxics-12-00425-s001.zip › Supplementary Materials/2D chemical structures/1932.pdf]

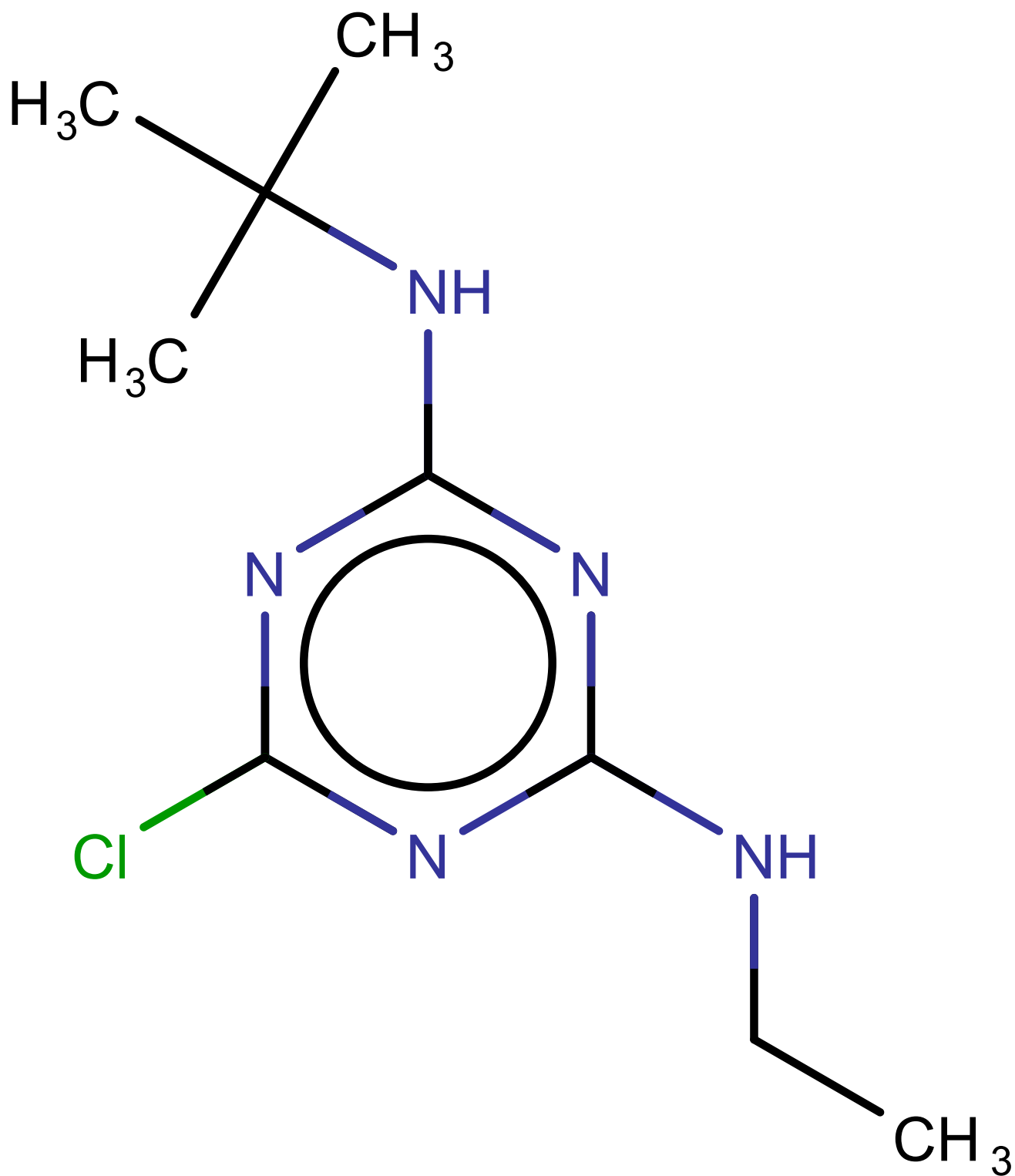

Supplement: Supplementary file 1 [file toxics-12-00425-s001.zip › Supplementary Materials/2D chemical structures/1934.pdf]

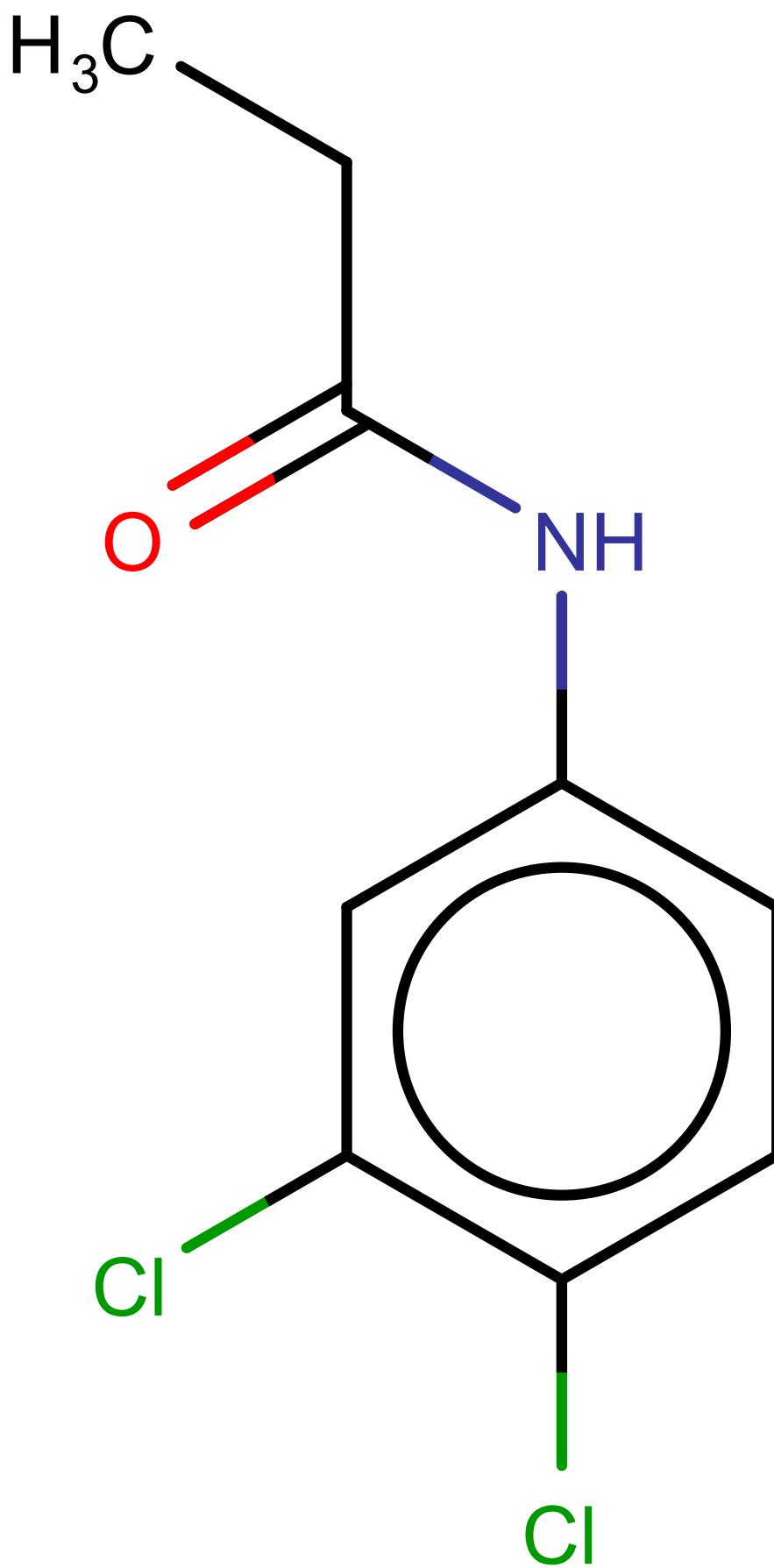

Supplement: Supplementary file 1 [file toxics-12-00425-s001.zip › Supplementary Materials/2D chemical structures/1956.pdf]

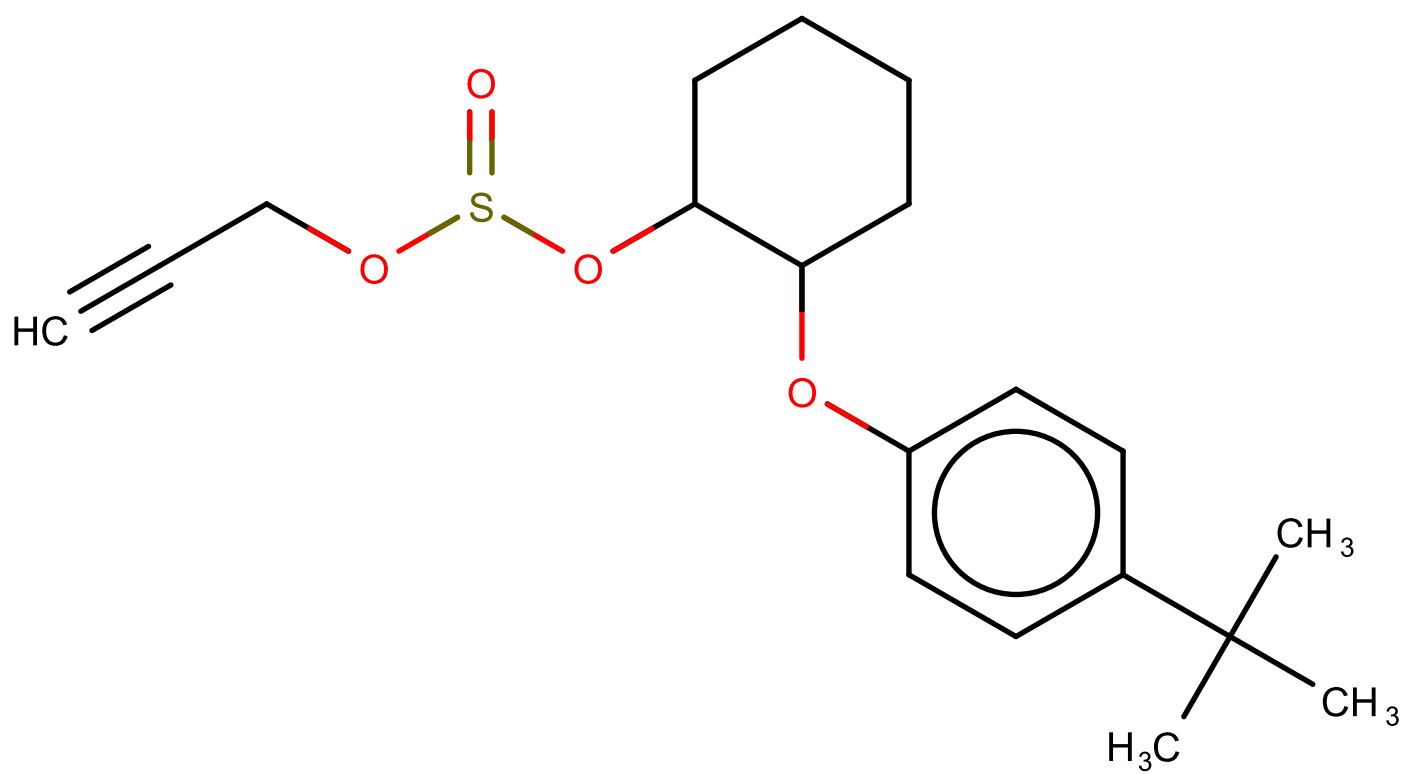

Supplement: Supplementary file 1 [file toxics-12-00425-s001.zip › Supplementary Materials/2D chemical structures/1960.pdf]

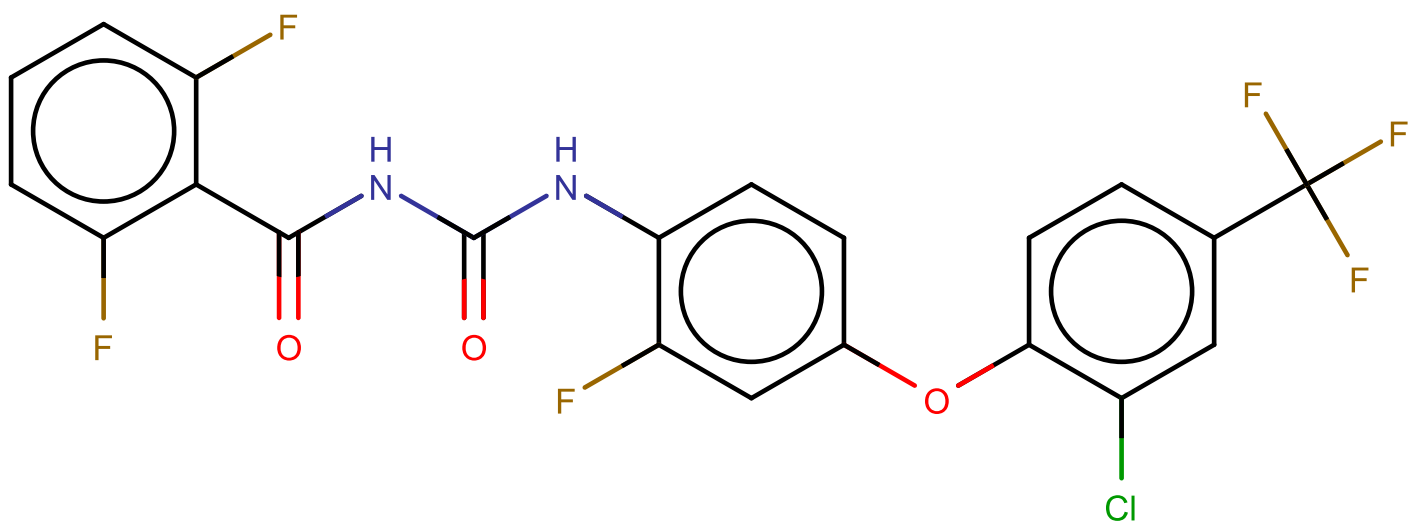

Supplement: Supplementary file 1 [file toxics-12-00425-s001.zip › Supplementary Materials/2D chemical structures/1962.pdf]

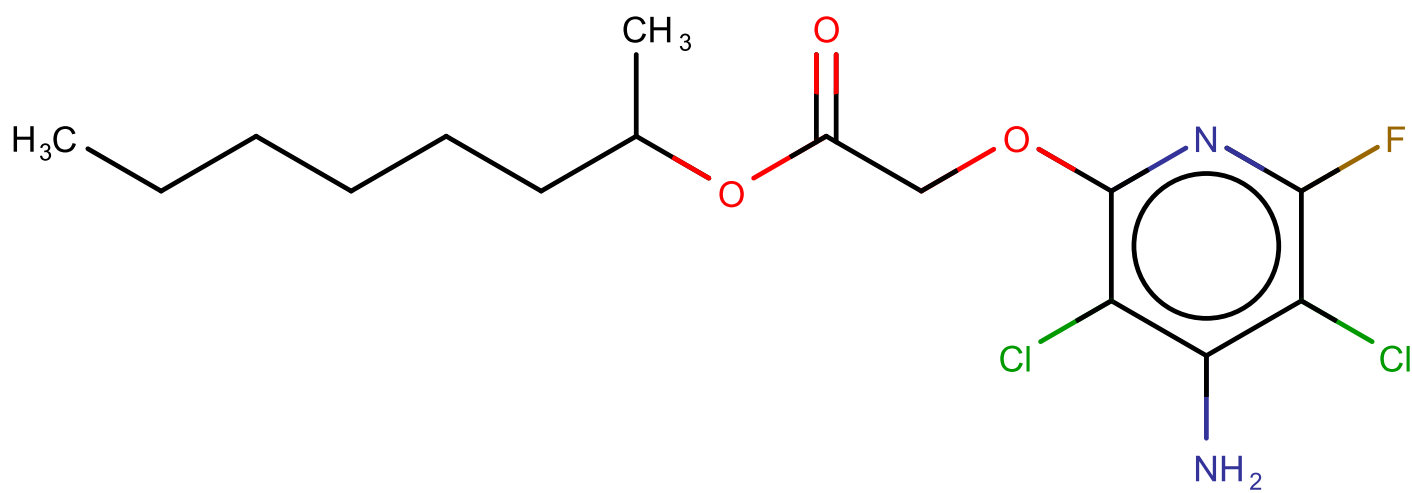

Supplement: Supplementary file 1 [file toxics-12-00425-s001.zip › Supplementary Materials/2D chemical structures/1970.pdf]

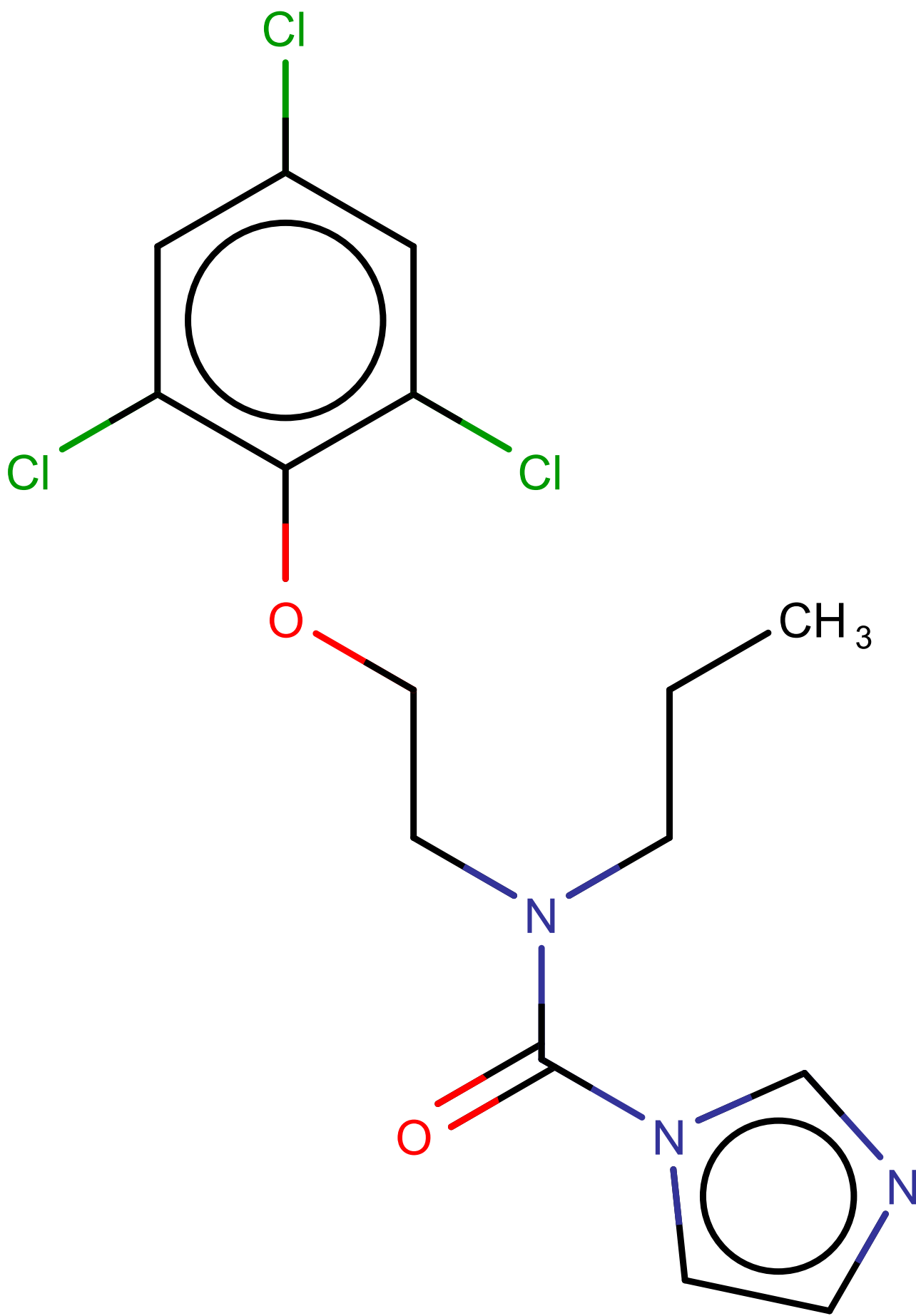

Supplement: Supplementary file 1 [file toxics-12-00425-s001.zip › Supplementary Materials/2D chemical structures/1995.pdf]
